# Chemoselective Synthesis of δ‑Amino Alcohols from Chiral 1,4-Diols Catalyzed by an NHC–Ir(III) Complex

**DOI:** 10.1021/acs.orglett.5c04592

**Published:** 2026-01-02

**Authors:** Emanuele Silvi, Aitor Bermejo-López, Sarko Jabbari, Mariell Pettersson, Magnus J. Johansson, Belén Martín-Matute

**Affiliations:** † Department of Chemistry, Organic Chemistry Unit, 7675 Stockholm University, 106 91 Stockholm, Sweden; ‡ Medicinal Chemistry *CVRM*, Discovery Sciences, 33367 Biopharmaceuticals R&D AstraZeneca, Mölndal 431 83, Gothenburg, Sweden

## Abstract

The synthesis of δ-amino alcohols from 1,4-diols via the *hydrogen borrowing* strategy by an NHC–Ir­(III) catalyst is described. The amination occurs on the primary alcohol selectively. The mild, base-free conditions effectively suppress the common second intramolecular amination to pyrrolidines. *sec*-Alcohols, even benzylic ones, remain inert toward amination and even without racemization, enabling the synthesis of chiral δ-amino alcohols from readily available chiral 1,4-diols. Late-stage functionalization (LSF) of Lenalidomide and other complex drugs is demonstrated.

## Graphical abstract

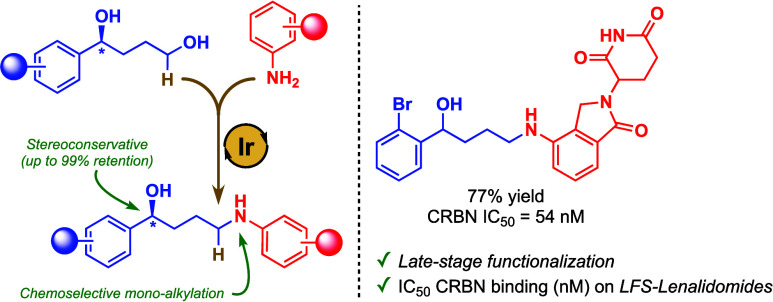

Efficient and selective formation of carbon–nitrogen (C–N) bonds is a fundamental transformation in synthetic organic chemistry. C–N bonds can be constructed through various methods, including Hofmann alkylations, Ullmann condensations, reductive aminations, Chan-Lam aminations,^1^ and Buchwald–Hartwig aminations.^2^ In recent years, amination via C–H activation has advanced remarkably,^3^ enabling challenging C–N bond construction in complex, highly functionalized, pharmaceuticals and drug intermediates via late-stage functionalization (LSF).^4^

Alcohols can serve as benign and safe alternatives to alkyl halides for the alkylation of amines via the borrowing hydrogen methodology (BHM).^5^ In this instance, water is the only byproduct, making it a reaction with high atom economy. Further, selective monoalkylation of amines, over consecutive alkylations, can be achieved under catalyst control.^6^ Various transition metal complexes—including those based on iridium, ruthenium, and first row transition metals —have been explored for this transformation.^5,7^

The BHM has been used primarily to introduce alkyl or benzylic substituents onto anilines and other relatively electron-poor amines, and even, though under harsher conditions, onto aliphatic amines.^8^ A few exceptions on complex functionalized amines, such as chiral amino acids,^9,10^ small peptides,^10^ and amino sugars,^11^ have been reported. On the other hand, BHM applied to functionalized alcohols has lagged behind. A few examples have been reported dealing with vicinal diols by the Beller,^12^ Zhang and Zhao,^13^ Oe,^14^ and Madsen groups.^15^ An alternative approach that involves BHM and hydroamination to yield γ-amino alcohols was reported by the Wang and Xiao groups.^16^

When 1,4-diols are used (Scheme 1), the vast majority of examples report double *N*-alkylations, providing the cyclized products, namely, pyrrolidines (Scheme 1a).^17^ The second alkylation proceeds readily under the required catalytic conditions. The Zhao group has reported an enantioconvergent synthesis of chiral pyrrolidines combining a chiral Ir catalyst and a chiral phosphoric acid (Scheme 1a).^18^ Restricting the reaction to monoalkylation, thus preventing the cyclization, gives access to valuable δ-amino alcohols,^19^ which are also scaffolds and precursors for biologically active compounds.^20^ A few rare examples have been reported; Zhao and co-workers achieved the synthesis of 4-(phenylamino)­butan-1-ol from 1,4-butanediol and aniline by [Cp*IrCl_2_]_2_ in moderate yields (Scheme 1b);^21^ Banerjee,^22^ Hao,^23^ and Shi^24^ reported amination of the same diol using Ni, Ru, or a CuNiAlO_*x*_ catalyst, respectively. Takacs and co-workers disclosed the single example reported using a nonsymmetrical 1,4-diol, pentane-1,4-diol, with aniline using a Ru catalyst (Scheme 1b).^25^

**Scheme 1:**
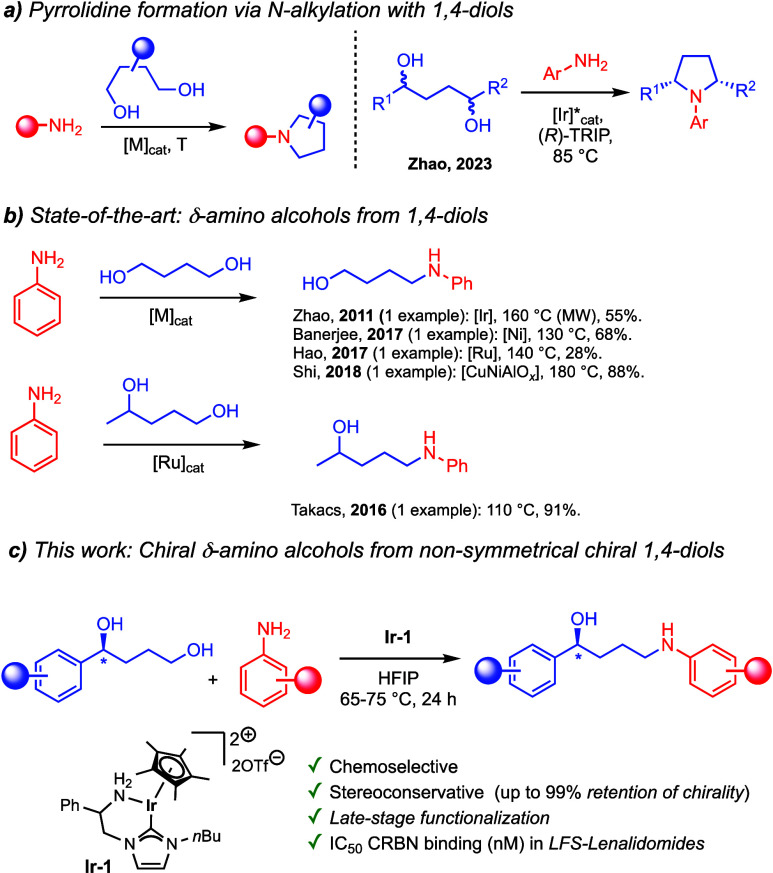
Syntheses of Pyrrolidine and δ-Amino Alcohols from 1,4-Diols^a^

a (a) From 1,4-diols to pyrrolidine via BHM. (b) Current examples on mono *N*-alkylation of aniline with symmetric and non-symmetric 1,4-diols. (c) This work.

Accessing δ-amino alcohols from 1,4-diols remains challenging due to the prevalent annulation to pyrrolidines. Stereoconservative cases in which chiral secondary alcohols are preserved under BHM catalytic conditions are unknown. Here, we address these limitations by demonstrating the versatility of complex **Ir-1** ^10,26^ for the direct monoalkylation of anilines by chiral 1,4-diols (Scheme 1c). Only the primary alcohol is selectively aminated in the presence of enantioenriched benzylic *sec*-alcohols. A particular focus on the late-stage functionalization of Lenalidomide offers a series of valuable intermediates for the synthesis of molecular glue or PROTAC compounds.

(*rac*)-1-Phenyl-1,4-butanol (**1a**) and aniline (**2a**) were selected for optimization (Table 1 and Table S2) using the NHC–Ir­(III) catalyst **Ir-1**. We specifically aimed at obtaining high selectivity toward the primary alcohol, targeting δ-amino alcohol **3aa**. Intra- or intermolecular multiple alkylations may also occur,^27^ with the former leading to pyrrolidine formation. We identified that 1,2-diphenylpyrrolidine **4aa** and amino ketone **5aa** were the major byproducts. 1,1,1,3,3,3-Hexafluoro-2-propanol (HFIP) was the optimal solvent (see Table S1). Catalyst loadings of 3 mol% gave good yields of **3aa** (Table 1, entry 1). Higher temperatures than 75 °C led to significantly larger amounts of **4aa** and **5aa** (Table 1, entry 2). Varying the **1a**/**2a** ratio from 2:1 to 1:1 (entry 3) or 0.5:1 (entry 4) led to lower yields of **3aa**. The best conditions were found with 2 equiv of **1a**, **Ir-1** (4 mol%) at 75 °C, affording **3aa** in 84% yield (Table 1, entry 5). These conditions served as the basis for exploring the substrate scope (Schemes 2 and 3). For certain substrates, the catalyst loading or reaction temperature could be further reduced (*vide infra*).

**Table 1:**
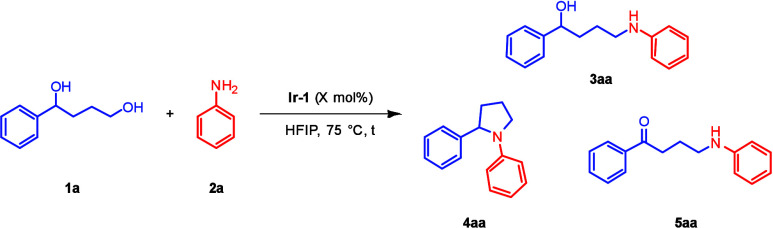
Optimization of the *N*-Alkylation **2a** with **1a** ^a^

entry	**Ir-1** (X mol%)	t (h)	**1a** (equiv)	**3aa** (%)^b^	**4aa** (%)^b^	**5aa** (%)^b^
1	3	24	2	73	3	2
2^c^	3	24	2	55	22	4
3	3	24	1	52	2	5
4^d^	3	24	0.5	55	3	5
**5**	**4**	**24**	**2**	**84**	**4**	**4**

a Unless otherwise noted: **2a** (0.2 mmol), **1a** (2 equiv), and **Ir-1** (mol% with respect to limiting reagent) in HFIP (0.4 M) under an inert atmosphere.

b Yield determined by ^1^H NMR spectroscopy using 1,1,2,2-tetrachloroethane (TCE) as internal standard.

c 100 °C.

d **1a** (0.2 mmol) and **2a** (0.4 mmol).

**Scheme 2:**
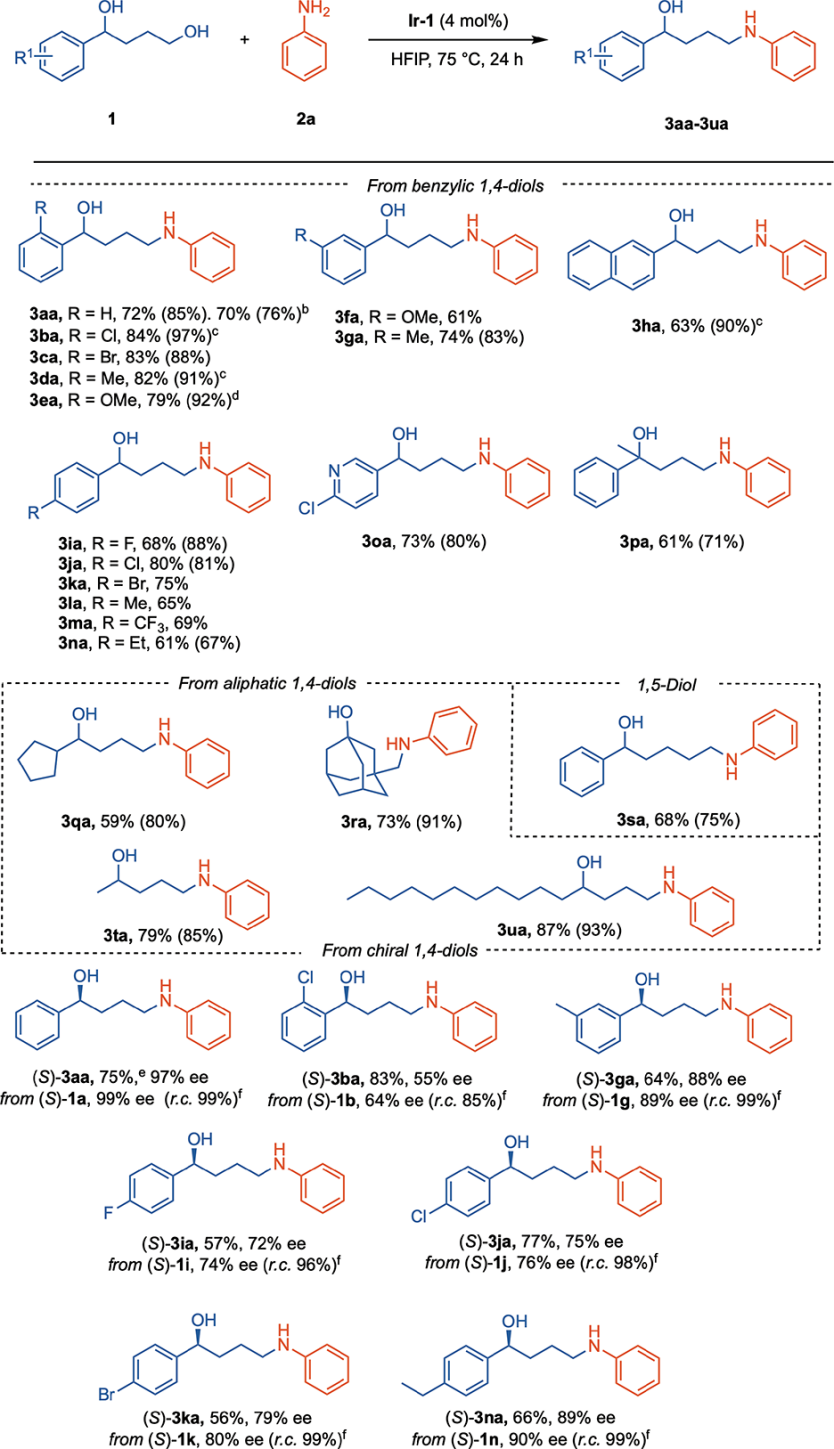
Scope of 1,4-Diols (**1**)­[Fn s2fn1]

a
**2a** (0.2 mmol), **1a**–**1u** (2 equiv), and **Ir-1** (4 mol%) in HFIP (0.4 M) under an inert atmosphere. Yields are isolated. In parentheses, yields determined by ^1^H NMR spectroscopy using TCE as internal standard are shown.
b 1 mmol **2a**.
c
**Ir-1**: 3 mol %.
d 65 °C.
e 0.1 mmol of **2a**.
f
*r.c.* = [*ee* of **3**/*ee* of **1**] × 100.

**Scheme 3:**
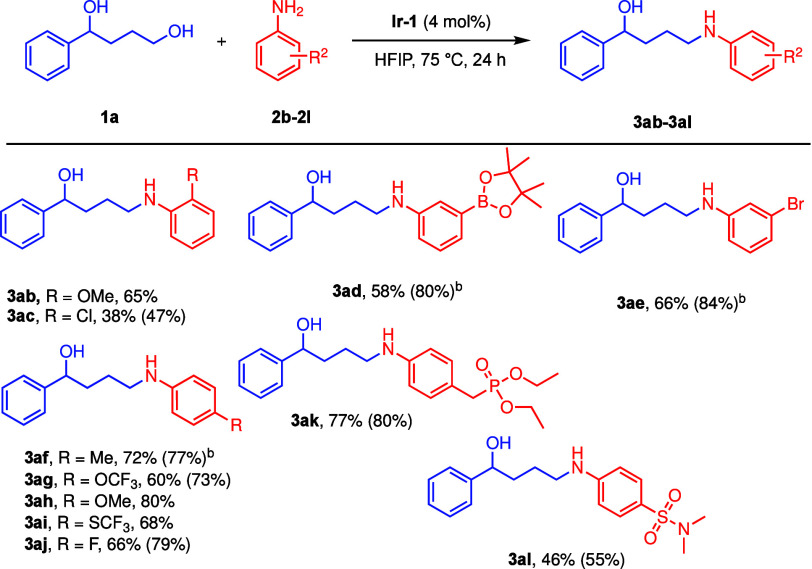
Scope of Anilines **2** ^a^

a Unless otherwise noted: **2b**–**2l** (0.2 mmol), **1a** (2 equiv), and **Ir-1** (4 mol%) in HFIP (0.4 M) under an inert atmosphere. Yields are isolated. In parentheses, yields determined by ^1^H NMR spectroscopy using TCE as internal standard are shown.
b
**Ir-1**: 3 mol%.

1,4-Diols with *ortho*-substituted phenyl groups gave excellent yields in general regardless of their electronic properties, selectively providing δ-amino alcohols **3aa**–**3ea** at 75 °C or lower temperatures (**3ea**), even with 3 mol% of catalyst (**3ba** and **3da**). *meta*-Substituted diols **1f** and **1g** provided **3fa** (R = *m*-OMe) and **3ga** (R = *m*-Me) in yields of 61% and 74%, respectively. Similarly, **3ha** was obtained in 63% isolated yield from naphthyl 1,4-diol **1h** with 3 mol% of **Ir-1**. A series of 1,4-diols with *para*-substituted phenyl rings provided δ-amino alcohols **3ia**–**3na** in good yields. Pyridine groups were tolerated (**3oa**, 73%) as well as a diol with a *tert*-benzylic alcohol (**3pa**, 61%). Aliphatic neopentyl and adamantly aliphatic diols gave good yields of **3qa** and **3ra**, respectively. Other aliphatic 1,4-diols bearing shorter (**1t**) or longer (**1u**) aliphatic chains gave the corresponding δ-amino alcohols **3ta** and **3ua** in excellent yields. 1-Phenyl-1,5-pentanediol also reacts selectively at the primary alcohol (**3sa**, 68% yield). Building on this chemoselectivity, we explored whether the reaction could proceed without erosion of the chiral center presented using enantioeriched 1,4 diols. Indeed, δ-amino alcohols (*S*)-**3aa**, (*S*)-**3ba**, (*S*)-**3ga**, (*S*)-**3ia**, (*S*)-**3ja**, (*S*)-**3ka**, and (*S*)-**3na** were obtained from the corresponding chiral diols (*S*)-**1** in good yields and with excellent retention of chirality (*r.c.*) levels in all instances above 96%, with the exception of *ortho*-substituted (*S*)-**3ba** for which racemization was observed to a larger extent (*r.c.* 85%).

The scope of the anilines was then investigated (Scheme 3). *ortho*-Substituted anilines gave moderate to low yields, giving **3ab** (*o*-OMe) and **3ac** (*o*-Cl) in 65% and 38%, respectively. *meta*-Substituted anilines, such as *m*-pinacolborane ester- and *m*-bromoaniline, afforded **3ad** and **3ae** in 58% and 66%, respectively, with 3 mol% of **Ir-1**. Good yields were obtained with *para*-substituted anilines **3af**–**3aj**, including those with phosphonate and sulfonamide substituents (**3ak** and **3al**). Aliphatic amines can be alkylated by benzylic and aliphatic alcohols by **Ir-1**.^26^ However, the higher temperature required for alkylating aliphatic amines results in competitive cyclization to pyrrolidines when they are applied to 1,4-diols.

The method was then applied to the late-stage functionalization (LSF) of marketed drugs^4a,28^ containing primary amines. This gave access to novel conjugation platforms taking advantage of the possible multifunctional properties of 1,4-diols also carrying a functionalized aromatic motif. Darunavir (**6a**), an antiretroviral drug for the treatment of HIV/AIDS with a very complex chemical architecture carrying three differentiated nitrogen functionalities, a *sec*-alcohol, a chiral acetal, and 5 stereogenic centers, was selected. To our delight, **7aa** was obtained in a very respectable 47% yield and with complete selectivity (Scheme 4). Aminoglutethimide (**6b**), a drug for the treatment of hormone related diseases and cancers, also afforded an excellent yield with complete selectivity toward the aniline functionality in the drug and the primary alcohol on the diol (**7ab**, 58% yield, Scheme 4). Next, LSF of the FDA-approved Lenalidomide (**6c**) and its C5 derivative **6d**, which are Cereblon (CRBN) analogues widely exploited for targeted protein degradation strategies, was explored. Currently, *N*-alkylated Lenalidomides are prepared via reductive aminations^29^ and nucleophilic substitutions;^30^ however, alkylation at the imide nitrogen also occurs under the reaction conditions.^31^ The method reported herein has a rather high efficiency and good functional group tolerance. The reaction between **6c** with **1a** gave **7ac** in 41% yield (Scheme 4). To extend the scope, two bromine-containing diols (**1c** and **1k**) were chosen as they provide a handle for conjugation with small molecule drugs, peptides, oligonucleotides, or lipids.^32^ Lenalidomide (**6c**) afforded **7cc** and **7kc** in 77% and 54% yield, respectively. The C5 Lenalidomide analogue (**6d**) provided similar results, giving **7ad**, **7cd**, and **7kd** in 56%, 70%, and 65% yields, respectively. On the other hand, pyrrolidine derivative **7dd** was obtained in 80% yield from *ortho*-methyl diol **1d**.

**Scheme 4:**
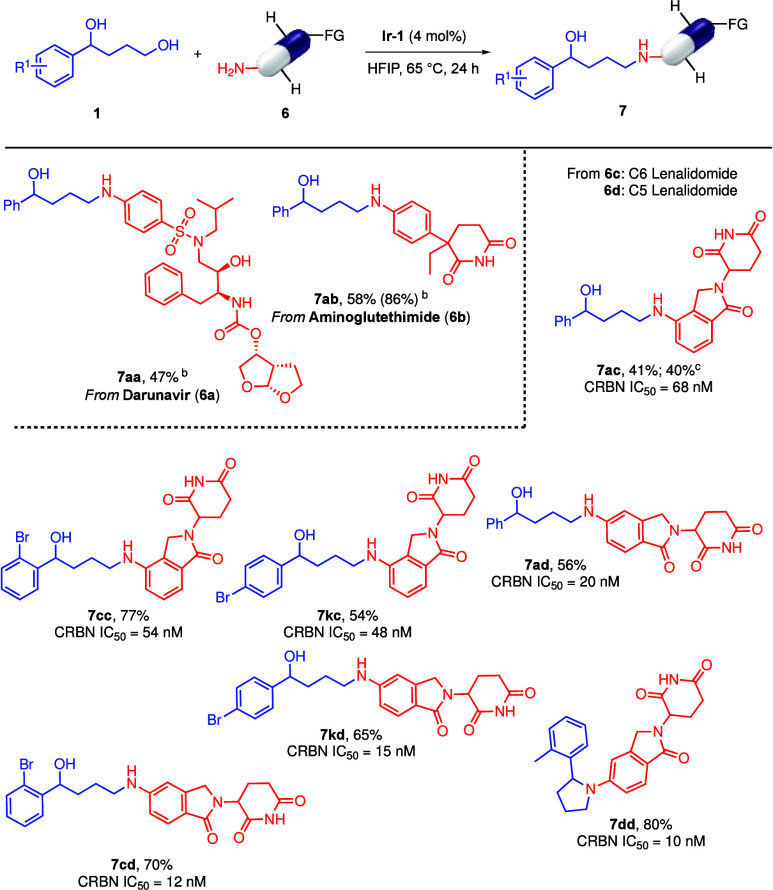
LSF of Biologically Relevant Molecules^a^

a Unless otherwise noted: **6** (0.2 mmol), **1** (2 equiv), and **Ir-1** (4 mol%) in HFIP (0.4 M) at 65 °C. Yields are isolated. In parentheses, yields determined by ^1^H NMR spectroscopy using TCE as internal standard are shown. CRBN binding was determined in a time-resolved-FRET competition assay using a Cy5-labeled CRBN ligand.
b 0.1 mmol of **6a** or **6b**.
c 75 °C.

All of the derivatives synthesized (**7ac**–**7dd**) retained nanomolar binding to CRBN (Scheme 4). Further, none of them affect cell viability in concentrations up to 100 μM in THP1 cells at a 48 h time point (Table S3), indicating that they are excellent candidates for further synthesis of molecular glue libraries or PROTAC compounds.

To understand the observed selectivity, we conducted control experiments. The reaction in the absence of the amine substrate results in full conversion of **1a** into cyclodehydration product **8a** (Scheme 5a and Figures S2–S4). Hammett studies (Figure S3) indicate intermediacy of benzylic carbocations in the formation of tetrahydrofurans **8** under these reaction conditions, i.e., in the absence of aniline (Scheme 5a).^27^ This unwanted reactivity is effectively inhibited when the reaction is performed in the presence of aniline **2a**. The excellent outcome obtained from enantioenriched/enantiopure diols (Scheme 2) further supports the absence of carbocation intermediates. From (*S*)-**1a**, (*S*)-**3aa** was formed in 75% yield with excellent chirality retention (Scheme 5b), even upon extended reaction times, highlighting the remarkable selectivity of **Ir-1** toward primary alcohols over secondary ones. In HFIP-*d*_2_, from **1a** (Scheme 5c), D was found on the aniline fragment, as well as at Cα and Cβ (20% and 48% D, respectively, Figure S7). D was not detected in recovered **1a** (Figure S9). From **1a**–***d***_**2**_ (98% D) in HFIP (Scheme 5c), the deuterium content diminished at Cα (52% D), and D was not incorporated at Cβ (see SI, Figure S8). From these control experiments, it can be concluded that (i) H/D scrambling occurs at the Ir–H/D stage,^33^ explaining D loss or incorporation at Cα, and that (ii) imine/enamine tautomerizations are involved, as D is incorporated at Cβ in the reactions run in HFIP-*d*_2_. The remaining **1a**–***d***_**2**_ in Scheme 5c, bottom maintained the original 98% D content (Figure S10).

**Scheme 5:**
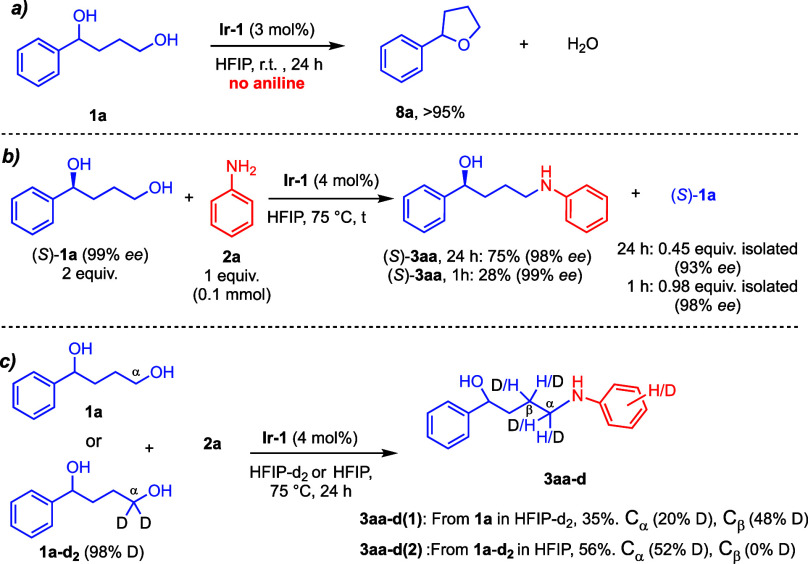
Control Experiments^a^

a Deuterium content determined on isolated product by ^1^H NMR spectroscopy.

A BHM mechanism, involving alcohol oxidation, imine formation, and imine reduction, with concomitant formation of Ir–H species is proposed (Scheme 6). As the reaction is run in HFIP, iminium species are likely intermediates.^26^ Iminium/enamine tautomerizations are involved for these aliphatic alcohol substrates, as confirmed by the deuterium-labeling studies shown in Scheme 5.

**Scheme 6:**
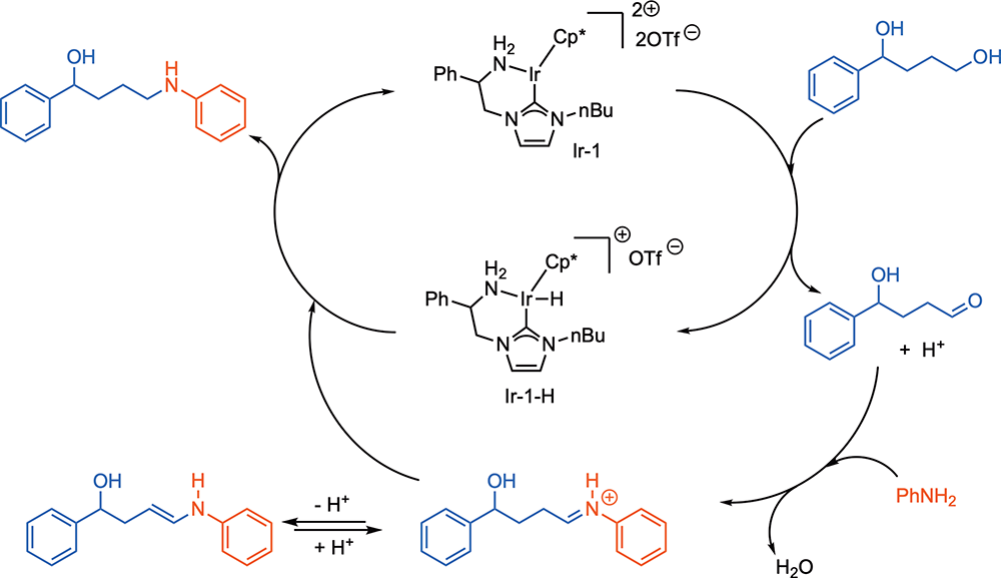
Proposed Mechanism

In conclusion, a method to access δ-amino alcohols from readily available nonsymmetrical 1,4-diols has been developed. The reaction proceeds via a *hydrogen borrowing* pathway mediated by **Ir-1**, where the dehydrative amination takes place selectively at the primary alcohol, in the presence of a *sec*-alcohol and without affecting the stereochemistry of the latter. The effectiveness and selectivity of the reaction was demonstrated with a large variety of substrates, including the LSF of Darunavir, aminoglutethimide, and C5 and C6 Lenalidomides. It is also shown that the obtained compounds bind to CRBN without affecting the THP1 cell viability up to 100 μM. Thus, they are potential candidates to afford new molecular glue libraries or PROTAC compounds, opening new opportunities in the late-stage diversification of aniline-based drugs.

## Supplementary Material



## Data Availability

The data underlying this study are available in the published article and in its Supporting Information and openly available in Zenodo at 10.5281/zenodo.17828442.

## Notes

General information; experimental procedures; supplementary tables and schemes; mechanism research; product derivation; characterization data of products; references (PDF)

## References

[1] Chen J.-Q., Li J.-H., Dong Z.-B. (2020). A Review on the Latest Progress of
Chan-Lam Coupling Reaction. Adv. Synth. Catal..

[2] Dorel R., Grugel C. P., Haydl A. M. (2019). The Buchwald–Hartwig
amination
after 25 years. Angew. Chem., Int. Ed..

[3] Singh R., Sathish E., Gupta A. K., Goyal S. (2021). 3d-Transition
Metal Catalyzed C-H to C-N bond Formation: An Update. Tetrahedron.

[4] Weis E., Johansson M. J., Martín-Matute B. (2021). Late-stage
amination of drug-like benzoic acids: Access to anilines and drug
conjugates via directed iridium-catalyzed C–H activation. Chem.—Eur. J..

[5] Reed-Berendt B. G., Latham D. E., Dambatta M. B., Morrill L. C. (2021). Borrowing Hydrogen for Organic Synthesis. ACS Cent. Sci..

[6] Templ J., Schnürch M. (2024). A guide for mono-selective *N*-methylation, *N*-ethylation, and *N*-*n*-propylation
of primary amines, amides, and sulfonamides and their applicability
in late-stage modification. Chem.-Eur. J..

[7] Irrgang T., Kempe R. (2019). 3d-Metal Catalyzed
N- and C-Alkylation
Reactions via Borrowing Hydrogen or Hydrogen Autotransfer. Chem. Rev..

[8] Podyacheva E., Afanasyev O. I., Vasilyev D. V., Chusov D. (2022). Borrowing Hydrogen
Amination Reactions: A Complex Analysis of Trends and Correlations
of the Various Reaction Parameters. ACS Catal..

[9] Yan T., Feringa B. L., Barta K. (2017). Direct *N*-Alkylation
of Unprotected Amino Acids with Alcohols. Sci.
Adv..

[10] Bermejo-López A., Raeder M., Martínez-Castro E., Martín-Matute B. (2022). Selective
and quantitative functionalization of unprotected α-amino acids
using a recyclable homogeneous catalyst. Chem..

[11] Bermejo-López A., Saavedra B., Dorst K. M., Obieta M., Maguire P., Martínez-Castro E., Widmalm G., Martín-Matute B. (2025). Selective *N*-Alkylation of Unprotected Amino Sugars by Alcohols. Application
to the Synthesis of Sugar-Based Surfactants. ChemistryEurope.

[12] Baehn S., Tillack A., Imm S., Mevius K., Michalik D., Hollmann D., Neubert L., Beller M. (2009). Ruthenium-catalyzed Selective Monoamination of Vicinal
Diols. ChemSusChem.

[13] Yang L. C., Wang Y. N., Zhang Y., Zhao Y. (2017). Acid-Assisted Ru-Catalyzed Enantioselective Amination of 1,2-Diols
through Borrowing Hydrogen. ACS Catal..

[14] Eka
Putra A., Oe Y., Ohta T. (2013). Ruthenium-Catalyzed
Enantioselective Synthesis of β-Amino Alcohols from 1,2-Diols
by “Borrowing Hydrogen”. Eur.
J. Org. Chem..

[15] Nordstrøm L. U., Madsen R. (2007). Iridium Catalysed Synthesis of Piperazines from Diols. Chem. Commun..

[16] Ma W., Zhang X., Fan J., Liu Y., Tang W., Xue D., Li C., Xiao J., Wang C. (2019). Iron-Catalyzed Anti-Markovnikov
Hydroamination and Hydroamidation
of Allylic Alcohols. J. Am. Chem. Soc..

[17] Fujita K.-I., Fujii T., Komatsubara A., Enoki Y., Yamaguchi R. (2007). An efficient synthesis of nitrogen
heterocycles by Cp*Ir-catalyzed *N*-cycloalkylation
of primary amines with diols. Heterocycles.

[18] Liu Y., Diao H., Hong G., Edward J., Zhang T., Yang G., Yang B. M., Zhao Y. (2023). Iridium-Catalyzed Enantioconvergent
Borrowing Hydrogen Annulation of Racemic 1,4-Diols with Amines. J. Am. Chem. Soc..

[19] Cristóbal C., Alonso I., Tato F., Cabrera-Afonso M. J., Adrio J., Ribagorda M. (2024). δ-Amination
of Alkyl Alcohols via Energy Transfer Photocatalysis. Org. Chem. Front..

[20] Reddy R. B., Dudhe P., Chauhan P., Sengupta S., Chelvam V. (2018). Synthesis
of tubuphenylalanine and *epi*-tubuphenylalanine via
regioselective aziridine ring opening with carbon nucleophiles followed
by hydroboration-oxidation of 1,1-substituted amino alkenes. Tetrahedron.

[21] Zhang W., Dong X., Zhao W. (2011). Microwave-Assisted
Solventless Reaction
of Iridium-Catalyzed Alkylation of Amines with Alcohols in the Absence
of Base. Org. Lett..

[22] Vellakkaran M., Singh K., Banerjee D. (2017). An Efficient
and Selective Nickel-Catalyzed
Direct *N*-Alkylation of Anilines with Alcohols. ACS Catal..

[23] Yang F.-L., Wang Y.-H., Ni Y.-F., Gao X., Song B., Zhu X., Hao X.-Q. (2017). An Efficient Homogenized
Ruthenium­(II) Pincer Complex
for *N*-Monoalkylation of Amines with Alcohols. Eur. J. Org. Chem..

[24] Wu Y., Yuan H., Shi F. (2018). Sustainable Catalytic Amination of
Diols: From Cycloamination to Monoamination. ACS Sustainable Chem. Eng..

[25] Marichev K. O., Takacs J. M. (2016). Ruthenium-Catalyzed Amination of
Secondary Alcohols
Using Borrowing Hydrogen Methodology. ACS Catal..

[26] Bermejo-López A., Li M., Dharanipragada N. V. R. A., Raeder M., Inge A. K., Himo F., Martín-Matute B. (2024). A general catalyst
for the base-free mono-*N*-alkylation of aromatic and
aliphatic amines with alcohols. Cell Rep. Phys.
Sci..

[27] Gonzalez
Miera G., Bermejo Lopez A., Martinez-Castro E., Norrby P.-O., Martin-Matute B. (2019). Nonclassical Mechanism in the Cyclodehydration
of Diols Catalyzed by a Bifunctional Iridium Complex. Chem.-Eur. J..

[28] Guillemard L., Ackermann L., Johansson M. J. (2024). Late-stage meta-C–H alkylation of pharmaceuticals
to modulate biological properties and expedite molecular optimization
in a single step. Nat. Commun..

[29] Li Q., Guo Q., Wang S., Wan S., Li Z., Zhang J., Wu X. (2022). Design and synthesis of proteolysis
targeting chimeras (PROTACs)
as an EGFR degrader based on CO-1686. Eur. J.
Med. Chem..

[30] Hu S., Yuan L., Yan H., Li Z. (2017). Design, synthesis and
biological evaluation of Lenalidomide derivatives as tumor angiogenesis
inhibitor. Bioorg. Med. Chem. Lett..

[31] Qiu X., Sun N., Kong Y., Li Y., Yang X., Jiang B. (2019). Chemoselective
synthesis of lenalidomide-based PROTAC library using alkylation reaction. Org. Lett..

[32] Buskes M. J., Blanco M.-J. (2020). Impact of cross-coupling
reactions in drug discovery and development. Molecules.

[33] Abura T., Ogo S., Watanabe Y., Fukuzumi S. (2003). Isolation
and Crystal Structure of a Water-Soluble
Iridium Hydride: A Robust and Highly Active Catalyst for Acid-Catalyzed
Transfer Hydrogenations of Carbonyl Compounds in Acidic Media. J. Am. Chem. Soc..

