# Supplementary material for: Chemoselective Synthesis of δ‑Amino Alcohols from Chiral 1,4-Diols Catalyzed by an NHC–Ir(III) Complex
Source: Org Lett. 2026 Jan 2;28(2):628–33. doi: 10.1021/acs.orglett.5c04592 (PMC12814538; doi:10.1021/acs.orglett.5c04592)
Supplement: Supplementary file 1 [file ol5c04592_si_001.pdf]

Chemoselective Synthesis of δ-Amino Alcohols from Chiral 1,4-Diols Catalyzed by an NHC-Ir(III) Complex

Emanuele Silvi, Aitor Bermejo-López, Sarko Jabbari, Mariell Pettersson, Magnus J. Johansson* and Belén Martín-Matute*

Table of Contents

1.1 General **2**

1.2 Synthesis of 1,4-diols starting materials **2**

1.2.1 Procedure A 2
1.2.2 Procedure B 7
1.2.3 Procedure C 9
1.2.4 Procedure D 12

1.3 Optimization **14**

1.3.1 Solvent screening 14
1.3.2 Byproduct evaluation 14
1.3.3 Optimization table 15

1.4 General procedure for the *N*-alkylation of anilines with 1,4-diols. **16**

1.4.1 Scope of the 1,4-diols 16
1.4.2 Scope of the anilines 26
1.4.3 Scope of *Late-stage functionalization* 30

1.5 Mechanistic investigations **35**

1.5.1 Reaction in absence of aniline (Scheme 5a), kinetic evaluation, byproduct **8** characterization 35
1.5.2 Reaction with Phenyl THF instead of 1,4-diol 37
1.5.3 Test reaction with chiral (*S*)-1,4-diol ((*S*)-**1a**) (Scheme 5b) 38
1.5.4 Control experiments with deuterated substrates or solvent (Scheme 5c) 41

1.6 Biological tests on Lenalidomide functionalized products **44**

1.7 NMR spectra of compounds **45**

1.7.1 Spectra of 1,4-diols starting material (**1**) 45
1.7.2 Spectra of *N*-alkylated anilines with 1,4-diols (**3**) 75
1.7.3 Spectra of *Late-stage functionalization* (**7aa – 7dd**) 124
1.7.4 Spectra of THF byproducts of Hammett plot (**8a – 8k**) 133

1.8 SFC chromatogram of chiral compound: *ee* determination **139**

1.8.1 SFC chromatogram of starting material 139
1.8.2 SFC chromatogram of products 147

1.9 References **154**

1.1 General

All reactions were carried out under an atmosphere of nitrogen in an oven dried round bottom flask or Biotage® microwave vial. Reagents were of analytical grade, obtained by commercial suppliers and used as purchased, unless otherwise specified. HFIP was dried by using activated molecular sieves, then filtered and distilled under reduced pressure. Thus, it was stored under an atmosphere of nitrogen in dark. Anhydrous dichloromethane and THF were obtained using a VAC solvent purification system or purchased by commercial suppliers. Flash column chromatography purifications were performed on Biotage ISOLUTE 25g, 10g, or 5g pre-loaded column, unless otherwise stated. Evaporative light scattering detector (ELSD) detection was used for non-UV active compounds. For HPLC purification preparative reverse-phase HPLC on a Kromasil C8 column (10 µm, 250x50 ID mm), using gradient elution (A: H_2O/MeCN/FA = 80/20/0.2, B: MeCN) with a flow rate of 100 mL/min over 20 minutes. NMR spectra for the characterization of compounds were recorded at room temperature on Bruker instruments: 400 MHz (^1H) and 100 MHz (^{13}C) or 500 MHz (^1H), at 126 MHz (^{13}C), 470 MHz (^{19}F), 160 MHz (^{11}B) and 202 MHz (^{31}P). Chemical shifts (δ) are reported in ppm, using the residual solvent peak in $CDCl_3$ (δ_H = 7.26 ppm and δ_C = 77.16 ppm), or DMSO-d_6 (δ_H = 2.50 ppm and δ_C = 39.52 ppm) as internal reference. Coupling constants (*J*) are given in Hz. High-resolution mass spectra (HRMS) were recorded on a Waters Ultra-Performance Liquid Chromatography (UPLC) system coupled to a Waters Synapt G2 Si quadrupole time-of-flight (Q-ToF) system with an ESI ionization source, or with a Bruker microTOF ESI-TOF spectrometer. Optical rotation was recorded on a thermostated polarimeter using sodium lamp (589 nm) and a 10 cm cell.

1.2 Synthesis of 1,4-diols starting materials

Starting material **1r** was purchased from Combi-Blocks and used without further purifications. Starting material **1t** was purchased from Sigma-Aldrich and used without further purifications.

1.2.1 Procedure A

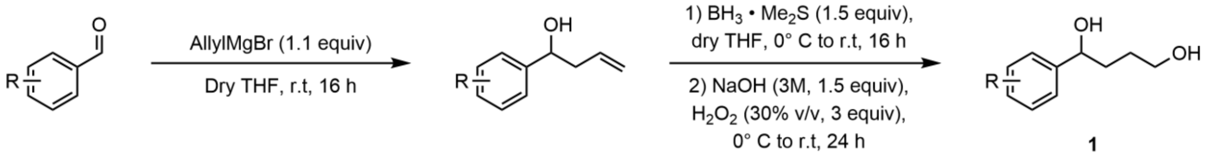

The following 1,4-diols were synthetized according to the reported procedure.[1] In an oven–dried 100 mL round bottom flask filled with nitrogen, the corresponding aldehyde was dissolved in dry THF (1.0 M) and the solution cooled down in an ice/water bath. Allyl magnesium bromide (1.0 M in THF, 1.1 equiv.) was added dropwise, and the reaction was allowed to reach room temperature while stirring overnight. Then, the mixture was diluted with Et_2O (20 mL) and a saturated solution of NH_4Cl (20 mL) was added to the reaction mixture, the organic phase was washed with Brine (3 x 20 mL) and dried over Na_2SO_4. The organic phase was filtered through a pad of celite, and the solvent evaporated under reduced pressure. The crude reaction was used without further purifications.

In an oven dried 250 mL round bottom flask filled with nitrogen, the corresponding benzylic alcohol from the previous step was dissolved in dry THF (1.0 M) and the solution cooled down in an ice/water bath. $BH_3 \cdot Me_2S$ was added dropwise (2.0 M in THF, 1.5 equiv), The reaction was then removed from the ice/water bath and let it stir overnight at room temperature. Then, the solution was cooled down in an ice/water bath and NaOH was added dropwise (3M, 1.5

equiv), followed by H_2O_2 solution (30% v/v, 3 equiv) added dropwise. During the additions the reaction was kept under nitrogen flow with an extra needle on the septum in case of over pressure. The reaction mixture was then removed from the ice bath and let stir at room temperature for 24 h. Saturated solution of NH_4Cl (20 mL) was added to the reaction mixture, and the organic phase extracted with EtOAc (3 x 20 mL). The organic phase was washed with Brine (3 x 20 mL) and dried over Na_2SO_4, filtered through a pad of celite, and the solvent evaporated under reduced pressure. Purification of the 1,4–diols was done via flash chromatographic column (DCM:MeOH 30:1) or performed on Biotage ISOLUTE SI (10–100% EtOAc in heptane, 5g, 10g, or 25 g) pre–loaded columns, unless otherwise stated.

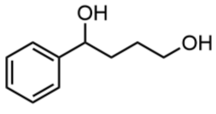

1–phenylbutane–1,4–diol (1a)
The compound **1a** was purified via automatic flash column chromatography (10–100% EtOAc in heptane, 25 g SiO_2), and obtained as a solid after freezing (3.00 g, 18.0 mmol, 60% yield over 2 steps).
^{1}H NMR (400 MHz, $CDCl_3$): δ_H = 7.38 – 7.32 (m, 4H), 7.28 (dt, *J* = 5.9, 3.1 Hz, 1H), 4.75 (t, *J* = 6.3 Hz, 1H), 3.77 – 3.64 (m, 2H), 1.88 (q, *J* = 6.9 Hz, 2H), 1.69 (ddq, *J* = 20.4, 13.8, 7.1 Hz, 2H) ppm.
^{13}C NMR (126 MHz, $CDCl_3$): δ_C = 144.8, 128.6, 127.7, 125.9, 74.6, 63.1, 36.3, 29.4 ppm.
The spectroscopic data is in agreement with those reported in the literature.[2]

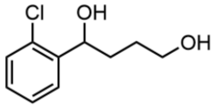

1–(2–chlorophenyl)butane–1,4–diol (1b)
The compound **1b** was purified via column chromatography (30:1 DCM:MeOH), and obtained as a colorless oil (1.52 g, 7.5 mmol, 75% yield over 2 steps).
^{1}H NMR (500 MHz, $CDCl_3$): δ_H 7.58 (d, *J* = 7.7 Hz, 1H), 7.34 – 7.27 (m, 2H), 7.19 (td, *J* = 7.7, 1.8 Hz, 1H), 5.15 (dt, *J* = 7.6, 3.8 Hz, 1H), 3.73 (dq, *J* = 24.5, 6.2 Hz, 2H), 2.42 (brs, 2H), 1.98 – 1.88 (m, 1H), 1.76 (dq, *J* = 10.3, 6.7 Hz, 3H) ppm.
^{13}C NMR (126 MHz, $CDCl_3$): δ_C = 142.2, 131.8, 129.5, 128.5, 127.2, 127.2, 70.8, 63.1, 34.8, 29.2 ppm.
The spectroscopic data is in agreement with those reported in the literature.[3]

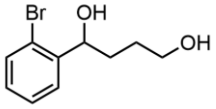

1–(2–bromophenyl)butane–1,4–diol (1c)
The compound **1c** was purified automatic flash column chromatography (10–100% EtOAc in heptane, 25 g SiO_2), and obtained as a colorless oil (1.28 g, 5.3 mmol, 52.6% yield over 2 steps).
^{1}H NMR (500 MHz, $CDCl_3$): δ_H 7.58 (dd, *J* = 7.8, 1.7 Hz, 1H), 7.51 (dd, *J* = 8.0, 1.2 Hz, 1H), 7.34 (td, *J* = 7.5, 1.2 Hz, 1H), 7.12 (ddd, *J* = 8.1, 7.4, 1.8 Hz, 1H), 5.10 (dd, *J* = 8.0, 3.5 Hz, 1H), 3.67 – 3.81 (m, 2H), 1.88 – 2.01 (m, 1H), 1.71 – 1.82 (m, 3H) ppm.
^{13}C NMR (126 MHz, $CDCl_3$): δ_C = 143.8, 132.7, 128.8, 127.8, 127.4, 121.9, 72.8, 62.8, 35.0, 29.2 ppm.
HRMS (ESI): *m/z* calc. for $C_{10}H_{12}BrO_2$ $[M - H^+]^-$: 243.0028; found: 243.0026.

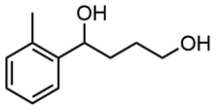

1–(o–tolyl)butane–1,4–diol (1d)

The compound **1d** was purified via automatic flash column chromatography (30:1 DCM:MeOH, 25 g SiO_2), and obtained as a white solid (0.5766 g, 3.2 mmol, 64% yield over 2 steps).

^{1}H NMR (500 MHz, $CDCl_3$): δ_H 7.52 (dd, 1H, *J*=7.6, 1.4 Hz), 7.24 – 7.28 (m, 1H), 7.15 – 7.22 (m, 2H), 5.02 (dd, 1H, *J*=7.7, 4.1 Hz), 3.69 – 3.81 (m, 2H), 2.37 (s, 3H), 1.70 – 1.93 (m, 4H) ppm.

^{13}C NMR (126 MHz, $CDCl_3$): δ_C = 143.0, 134.4, 130.5, 127.3, 126.4, 125.2, 70.9, 63.1, 35.3, 29.7, 19.2 ppm.

The spectroscopic data is in agreement with those reported in the literature.[4]

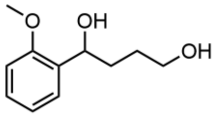

1–(2–methoxyphenyl)butane–1,4–diol (1e)

The compound **1e** was purified via automatic flash column chromatography (10–100% EtOAc in heptane, 25 g SiO_2), and obtained as a white solid (0.94 g, 4.8 mmol, 48% yield over 2 steps). M.p: 74-75°C.

^{1}H NMR (500 MHz, $CDCl_3$): δ_H 7.36 (dd, 1H, *J*=7.6, 1.7 Hz), 7.24 – 7.31 (m, 2H), 6.99 (td, 1H, *J*=7.5, 1.1 Hz), 6.91 (dd, 1H, *J*=8.2, 1.2 Hz), 4.97 (dd, 1H, *J*=7.6, 5.1 Hz), 3.88 (s, 3H), 3.67 – 3.79 (m, 2H), 1.91 (dddd, 2H, *J*=9.7, 8.6, 5.8, 2.4 Hz), 1.67 – 1.84 (m, 2H) ppm.

^{13}C NMR (126 MHz, $CDCl_3$): δ_C = 156.5, 132.5, 128.5, 126.9, 120.9, 110.6, 70.8, 63.2, 55.4, 34.3, 29.7 ppm.

HRMS (ESI): *m/z* calc. for $C_{11}H_{13}O$ $[(M - 2\ H_2O) + H]^+$: 161.0961 ; found: 161.0979.

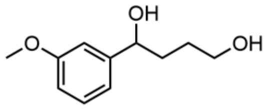

1–(3–methoxyphenyl)butane–1,4–diol (1f)

The compound **1f** was purified via automatic flash column chromatography (10–100% EtOAc in heptane, 10 g SiO_2), and obtained as a colorless oil (0.5672 g, 2.89 mmol, 58% yield over 2 steps).

^{1}H NMR (500 MHz, $CDCl_3$): δ_H = 7.22 – 7.29 (m, 1H), 6.90 – 6.95 (m, 2H), 6.81 (ddd, 1H, *J*=8.2, 2.6, 1.0 Hz), 4.71 (t, 1H, *J*=6.3 Hz), 3.81 (s, 3H), 3.68 (hept, 2H, *J*=5.4 Hz), 1.86 (td, 2H, *J*=7.4, 6.1 Hz), 1.60 – 1.77 (m, 2H) ppm.

^{13}C NMR (126 MHz, $CDCl_3$): δ_C = 159.6, 146.6, 129.3, 118.2, 112.6, 111.4, 73.9, 62.4, 55.2, 36.2, 29.0 ppm.

HRMS (ESI): *m/z* calc. for $C_{11}H_{15}O_3$ $[M - H^+]^-$: 195.1027; found: 195.1027.

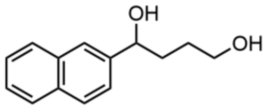

1–(naphthalen–2–yl)butane–1,4–diol (1h)

The compound **1h** was purified via column chromatography (30:1 DCM:MeOH), and obtained as a white solid (0.7834 g, 3.6 mmol, 72.4% yield over 2 steps).
^{1}H NMR (500 MHz, $CDCl_3$): δ_H 7.73 – 7.83 (m, 3H), 7.71 (d, 1H, *J*=1.6 Hz), 7.42 – 7.48 (m, 2H), 7.40 (dd, 1H, *J*=8.5, 1.7 Hz), 4.75 (t, 1H, *J*=6.3 Hz), 3.99 – 4.20 (brm, 1H), 3.49 – 3.62 (m, 2H), 3.46 (brs, 1H), 1.84 (q, 2H, *J*=7.0 Hz), 1.60 (ttd, 2H, *J*=14.2, 7.3, 5.6 Hz) ppm
^{13}C NMR (126 MHz, $CDCl_3$): δ_C = 142.2, 133.3, 132.9, 128.2, 128.0, 127.7, 126.1, 125.8, 124.5, 124.2, 74.3, 62.6, 36.2, 29.1 ppm.
The spectroscopic data is in agreement with those reported in the literature.[5]

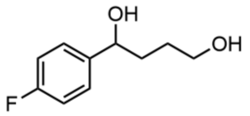

1–(4–fluorophenyl)butane–1,4–diol (1i)
The compound **1i** was purified via column chromatography (30:1 DCM:MeOH), and obtained as a colorless solid (1.00 g, 5.5 mmol, 55% yield over 2 steps).
^{1}H NMR (500 MHz, $CDCl_3$): δ_H 7.26 – 7.31 (m, 2H), 6.96 – 7.04 (m, 2H), 4.67 (dd, 1H, *J*=7.3, 5.1 Hz), 3.57 – 3.70 (m, 2H), 1.77 – 1.86 (m, 2H), 1.55 – 1.71 (m, 2H) ppm.
^{13}C NMR (126 MHz, $CDCl_3$): δ_C = 163.1, 161.2, 140.58 (d, *J*=3.1 Hz), 127.51 (d, *J*=8.1 Hz), 115.29 (d, *J*=21.3 Hz), 73.70 (d, *J*=2.8 Hz), 62.75 (d, *J*=4.0 Hz), 36.6, 29.14 (d, *J*=1.5 Hz) ppm.
^{19}F NMR (470 MHz, $CDCl_3$): δ_F = –115.4 ppm
The spectroscopic data is in agreement with those reported in the literature.[6]

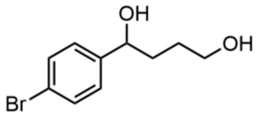

1–(4–bromophenyl)butane–1,4–diol (1k)
The compound **1k** was purified via column chromatography (30:1 DCM:MeOH), and obtained as a white solid (0.91 g, 3.7 mmol, 37% yield over 2 steps).
^{1}H NMR (500 MHz, $CDCl_3$): δ_H 7.44 – 7.50 (m, 2H), 7.21 – 7.27 (m, 2H), 4.71 (dd, 1H, *J*=7.1, 5.4 Hz), 3.64 – 3.76 (m, 2H), 1.77 – 1.90 (m, H), 1.59 – 1.76 (m, 2H) ppm.
^{13}C NMR (126 MHz, $CDCl_3$): δ_C = ^{13}C NMR (126 MHz, $CDCl_3$) δ 143.9, 131.7, 127.7, 121.3, 73.9, 63.0, 36.5, 29.1 ppm.
The spectroscopic data is in agreement with those reported in the literature.[7]

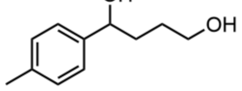

1–(p–tolyl)butane–1,4–diol (1l)
The compound **1l** was purified via column chromatography (30:1 DCM:MeOH), and obtained as a white solid (1.21 g, 6.7 mmol, 67% yield over 2 steps).
^{1}H NMR (500 MHz, $CDCl_3$): δ_H = 7.25 (d, 2H, *J*=8.2 Hz), 7.16 (d, 2H, *J*=7.7 Hz), 4.67 – 4.74 (m, 1H), 3.70 (hept, 2H, *J*=5.3 Hz), 2.34 (s, 3H), 1.79 – 1.93 (m, 2H), 1.59 – 1.77 (m, 2H) ppm.
^{13}C NMR (126 MHz, $CDCl_3$): δ_C = ^{13}C NMR (126 MHz, $CDCl_3$) δ 141.9, 137.4, 129.3, 125.9, 74.5, 63.1, 36.2, 29.4, 21.3 ppm.
The spectroscopic data is in agreement with those reported in the literature.[7]

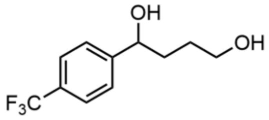

1–(4–(trifluoromethyl)phenyl)butane–1,4–diol (1m)
The compound **1m** was purified via automatic flash column chromatography (10–100% EtOAc in heptane, 10 g SiO_2), and obtained as a colorless oil (307.3 mg, 1.31 mmol, 26% yield over 2 steps).
^{1}H NMR (500 MHz, $CDCl_3$): δ_H = 7.42 (d, 2H, *J*=8.1 Hz), 7.27 (d, 2H, *J*=8.1 Hz), 4.57 (dd, 1H, *J*=8.0, 4.4 Hz), 4.17 (brs, 1H), 3.40 – 3.54 (m, 2H), 3.25 (brs, 1H), 1.58 – 1.74 (m, 2H), 1.43 – 1.57 (m, 2H) ppm.
^{13}C NMR (126 MHz, $CDCl_3$): δ_C = 148.8, 129.6 (q, *J*=32.3 Hz), 127.5, 125.4 (q, *J*=3.6 Hz), 122.12 (d, *J*=271.9 Hz), 73.6, 62.6, 36.7, 28.9 ppm.
^{19}F NMR (470 MHz, $CDCl_3$): δ_F = – 62.4 ppm.
The spectroscopic data is in agreement with those reported in the literature.[7]

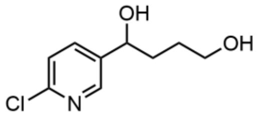

(6-chloropyridin-3-yl)butane-1,4-diol (1o)
The compound **1o** was purified via column chromatography (30:1 DCM:MeOH), and obtained as a colorless oil (0.47 g, 2.3 mmol, 47% yield over 2 steps).
^{1}H NMR (500 MHz, $CDCl_3$): δ_H = 8.25 (d, 1H, *J*=2.4 Hz), 7.64 (dd, 1H, *J*=8.2, 2.5 Hz), 7.23 – 7.28 (m, 1H), 4.71 (dd, 1H, *J*=7.8, 4.7 Hz), 4.25 (brs, 2H), 3.53 – 3.69 (m, 2H), 1.72 – 1.86 (m, 2H), 1.62 (tdd, 2H, *J*=13.2, 7.6, 1.9 Hz) ppm.
^{13}C NMR (126 MHz, $CDCl_3$): δ_C = 149.9, 147.3, 139.6, 136.9, 124.2, 70.9, 62.3, 36.5, 28.7. ppm.
HRMS (ESI): *m/z* calc. for $C_9H_{13}ClNO_2$ $[M+H]^+$: 202.0629 ; found: 202.0641.

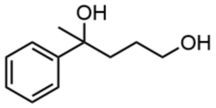

4-phenylpentane-1,4-diol (1p)
The compound **1p** was purified via automatic flash column chromatography (10–100% EtOAc in heptane, 10 g SiO_2), and obtained as a colorless oil (0.82 g, 4.56 mmol, 46% yield over 2 steps).
^{1}H NMR (500 MHz, $CDCl_3$): δ_H = 7.40 – 7.46 (m, 2H), 7.30 – 7.38 (m, 2H), 7.21 – 7.26 (m, 1H), 3.61 (hept, 2H, *J*=5.4 Hz), 2.64 (brs, 1H), 2.01 (dt, 1H, *J*=14.5, 7.3 Hz), 1.84 – 1.94 (m, 2H), 1.58 (s, 3H), 1.51 (dddd, 2H, *J*=12.6, 8.0, 6.0, 1.5 Hz) ppm.
^{13}C NMR (126 MHz, $CDCl_3$): δ_C = 148.1, 128.2, 126.5, 125.0, 74.4, 63.1, 41.3, 30.8, 27.3 ppm.
HRMS (ESI): *m/z* calc. for $C_{11}H_{13}$ $[(M - 2\ H_2O) +H]^+$: 145.1012 ; found: 145.1021.

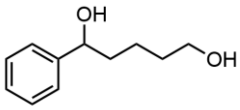

1-phenylpentane-1,5-diol (1s)

The compound **1s** was purified via automatic flash column chromatography (10–100% EtOAc in heptane, 10 g SiO_2), and obtained as a colorless oil (0.54 g, 3.0 mmol, 47% yield over 2 steps).
^{1}H NMR (500 MHz, $CDCl_3$): δ_H = 7.35 (d, 4H, *J*=3.9 Hz), 7.26 – 7.31 (m, 1H), 4.69 (ddd, 1H, *J*=7.6, 5.6, 3.2 Hz), 3.64 (td, 2H, *J*=6.4, 5.2 Hz), 1.88 (d, 1H, *J*=3.4 Hz), 1.71 – 1.87 (m, 2H), 1.45 – 1.67 (m, 4H), 1.31 – 1.44 (m, 1H), 1.28 (t, 1H, *J*=5.4 Hz) ppm.
^{13}C NMR (126 MHz, $CDCl_3$): δ_C = 144.9, 128.6, 127.6, 125.9, 74.5, 62.7, 38.8, 32.5, 22.1 ppm.
HRMS (ESI): *m/z* calc. for $C_{11}H_{13}$ [(M - 2 H_2O) +H]$^+$: 145.1012 ; found: 145.1019.

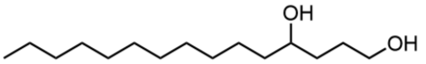

Pentadecane-1,4-diol (1u)
The compound **1u** was purified via automatic flash column chromatography (10–100% EtOAc in heptane, 10 g SiO_2), and obtained as an amorphous white solid (0.165 g, 0.67 mmol, 34% yield over 2 steps). M.p: 49–52 °C
^{1}H NMR (400 MHz, $CDCl_3$): δ_H = 3.74 – 3.59 (m, 3H), 1.75 – 1.60 (m, 3H), 1.55 – 1.38 (m, 3H), 1.26 (d, *J* = 4.8 Hz, 20H), 0.94 – 0.84 (m, 3H) ppm.
^{13}C NMR (101 MHz, $CDCl_3$): δ_C = 72.1, 63.2, 37.8, 34.5, 32.1, 29.8, 29.8, 29.8, 29.8, 29.5, 29.3, 25.9, 22.8, 14.3 ppm.
HRMS (ESI): *m/z* calc. for $C_{15}H_{31}O_2$ [M - H$^+$]$^-$: 243.2330; found:243.2329.

1.2.2 Procedure B

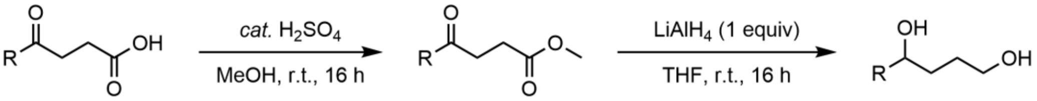

Transesterification step was performed according to the reported procedure.[8] An oven–dried 50 mL round bottom flask, 4-oxo-4-arylbutyrric acid (1 equiv.) was dissolved in MeOH (5 mL), and catalytic amount of H_2SO_4 concentrated were added to the reaction mixture, and let it stir for 16 hours at room temperature under air. Water (20 mL) was added to the reaction mixture and the emulsion extracted with Et_2O (3 x 20 mL), and the combined organic extracts were dried over sodium sulfate. The mixture was filtered, and the solvent was removed under vacuum. Purification via flash column chromatography (10–100% EtOAc in heptane, 5 g SiO_2), provided the methyl ester, used for the next step.
In an oven–dried 50 mL round bottom flask filled with nitrogen, a solution 1M of $LiAlH_4$ was added (1 equiv.) to dry THF (1 mL), then a solution of methyl ester from the previous step (1 equiv.) in dry THF (2 mL) was carefully added dropwise, and the reaction mixture was let stir for 16 h at room temperature. Then, the reaction mixture was cooled in an ice bath. Saturated solution of NH_4Cl (10 mL) was added dropwise, then extracted with EtOAc (3 x 20 mL) and the combined organic extracts were dried over sodium sulfate. The mixture was filtered, and the solvent was removed under vacuum, provided the product without further purification, unless otherwise specified.

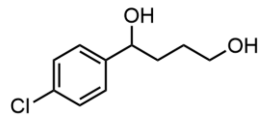

1–(4–chlorophenyl)butane–1,4–diol (1j)
The compound **1j** was purified via column chromatography (30:1 DCM:MeOH), and obtained as a colorless oil (51 mg, 0.25 mmol, 37% yield over 2 steps).
^{1}H NMR (500 MHz, $CDCl_3$): δ_H 7.34 – 7.25 (m, 4H), 4.68 (dd, J = 7.3, 5.2 Hz, 1H), 3.66 (qt, J = 10.8, 5.6 Hz, 2H), 1.88 – 1.76 (m, 2H), 1.73 – 1.58 (m, 2H) ppm.
^{13}C NMR (126 MHz, $CDCl_3$): δ_C = ^{13}C NMR (126 MHz, $CDCl_3$) δ 143.3, 133.1, 128.6, 127.3, 73.7, 62.8, 36.6, 29.1 ppm.
HRMS (ESI): *m/z* calc. for $C_{10}H_{12}ClO_2$ $[M - H^+]^-$: 199.0526 ; found: 199.0531.

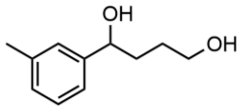

1-(m-tolyl)butane-1,4-diol (1g)
The compound **1g** was obtained as a white amorphous solid (96 mg, 0.53 mmol, 82% yield over 2 steps). M.p.: 61-64°C
^{1}H NMR (500 MHz, $CDCl_3$): δ_H = 7.23 (t, J = 7.5 Hz, 1H), 7.18 – 7.16 (m, 1H), 7.13 (dd, J = 7.8, 1.8 Hz, 1H), 7.08 (ddt, J = 7.4, 1.8, 1.0 Hz, 1H), 4.68 (t, J = 6.3 Hz, 1H), 3.73 – 3.62 (m, 2H), 2.35 (s, 3H), 1.90 – 1.81 (m, 2H), 1.76 – 1.59 (m, 2H) ppm.
^{13}C NMR (126 MHz, $CDCl_3$): δ_C = 144.8, 138.3, 128.5, 128.4, 126.6, 123.0, 74.6, 63.0, 36.3, 29.4, 21.6. ppm.
HRMS (ESI): *m/z* calc. for $C_{11}H_{15}O_2$ $[M - H^+]^-$: 179.1078; found: 179.1078.

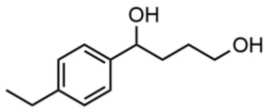

1-(4-ethylphenyl)butane-1,4-diol (1n)
The compound **1n** was obtained as a colorless oil (131 mg, 0.68 mmol, 83% yield over 2 steps).
^{1}H NMR (500 MHz, $CDCl_3$): δ_H = 7.25 – 7.30 (m, 2H), 7.15 – 7.21 (m, 2H), 4.71 (dd, 1H, J=7.2, 5.6 Hz), 3.68 (hept, 2H, J=5.4 Hz), 2.64 (q, 2H, J=7.6 Hz), 2.34 (brs, 1H), 1.97 (brs, 1H), 1.80 – 1.92 (m, 2H), 1.59 – 1.77 (m, 2H), 1.23 (t, 3H, J=7.6 Hz) ppm.
^{13}C NMR (126 MHz, $CDCl_3$): δ_C = 143.6, 142.1, 128.0, 125.9, 74.3, 62.8, 36.3, 29.4, 28.6, 15.7 ppm.
HRMS (ESI): *m/z* calc. for $C_{12}H_{15}$ $[(M - 2\ H_2O) + H]^+$: 159.1168 ; found: 159.1173.

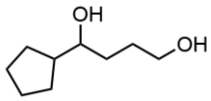

1-cyclopentylbutane-1,4-diol (1q)
The compound **1q** was purified via automatic flash column chromatography (10–100% EtOAc in heptane, 10 g SiO_2, ELSD was used to detect the product), and obtained as a colorless oil (0.30 g, 1.9 mmol, 74% yield over 2 steps).
^{1}H NMR (500 MHz, $CDCl_3$): δ_H = 3.72 – 3.56 (m, 2H), 3.40 (ddd, J = 9.2, 7.5, 2.1 Hz, 1H), 3.17 – 2.64 (brs, 2H), 1.92 – 1.82 (m, 1H), 1.82 – 1.74 (m, 1H), 1.74 – 1.49 (m, 8H), 1.44 (dddd, J = 13.8, 9.1, 6.9, 4.7 Hz, 1H), 1.33 (dq, J = 12.1, 8.0 Hz, 1H), 1.23 – 1.11 (m, 1H) ppm.
^{13}C NMR (126 MHz, $CDCl_3$): δ_C = ^{13}C NMR (126 MHz, $CDCl_3$) δ 76.1, 63.0, 46.6, 33.3, 29.4, 29.3, 29.0, 25.8, 25.7 ppm.

HRMS (ESI): *m/z* calc. for $C_9H_{18}NaO_2$ $[M+Na]^+$: 181.1199 ; found: 181.1188.

1.2.3 Procedure C

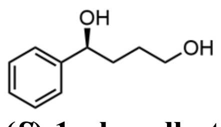

(*S*)-1-phenylbutane-1,4-diol [(*S*)-1a]
The starting material (*S*)-**1a** was obtained by chiral separation from the racemic mixture of **1a**. Shimadzu SFC Prep equipped with a column Lux C4 (250 x 30, particle size 3 μm) was used with 23% IPA/DEA 100/20mM in CO_2, 120 bar. The enantiomeric excess was determined with a Lux C4 column (150 x 4.6, particle size 3 μm) and a mobile phase of 30% IPA/DEA 100/20mM in CO_2 120 bar, 3.5 ml/min, λ = 257 nm, enantiomer 1 t_r = 1.016 min. (99.2% *ee*), enantiomer 2 t_r = 1.166 min. (97.6% *ee*). The enantiomer 1 was the one used for the control experiments. Measurement of optical rotation allowed the stereochemistry assignment.
$[\alpha]^{20}_D$: –31 (c 0.97, MeOH), in accordance with literature.[9]

Chiral strting material (*S*)-**1b**, (*S*)-**1g**, (*S*)-**1i**, (*S*)-**1j**, (*S*)-**1k**, and (*S*)-**1n** were synthetized according the reported procedure.[10]

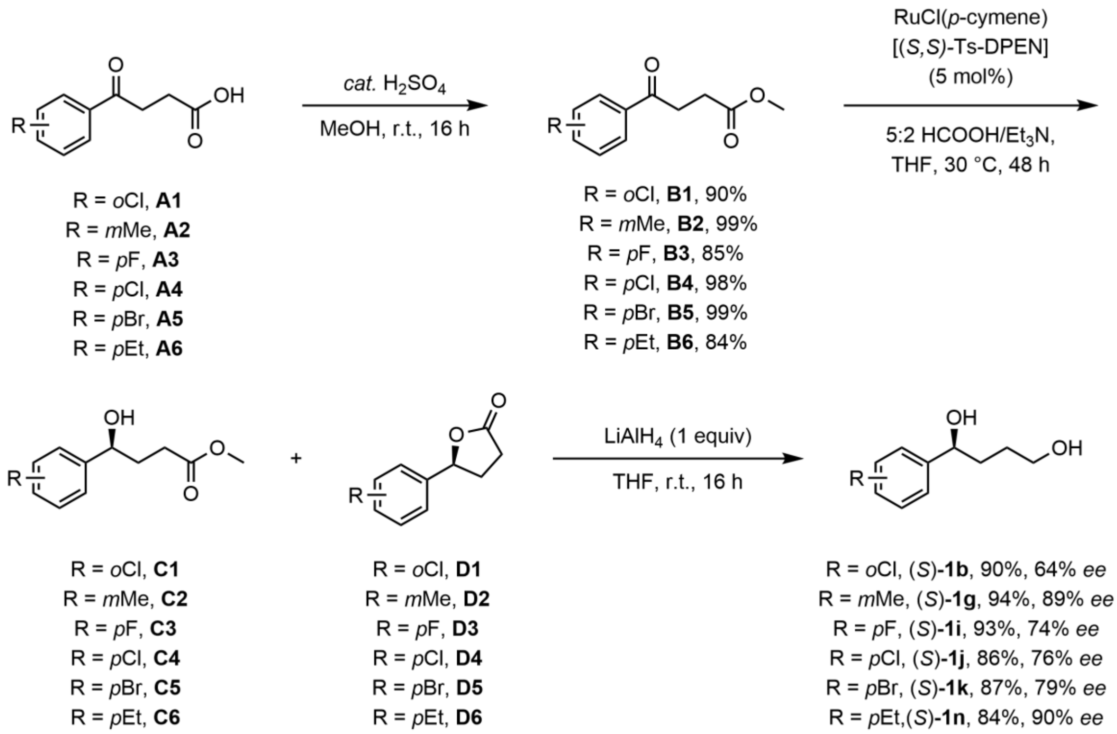

C1 + D1 = 55%
C2 + D2 = 55%
C3 + D3 = 75%
C4 + D4 = 76%
C5 + D5 = 73%
C6 + D6 = 64%

Transesterification step was performed according to the reported procedure.[8] An oven–dried 50 mL round bottom flask, 4-oxo-4-arylbutyrric acid was dissolved in MeOH (5 mL), and catalytic amount of H_2SO_4 concentrated were added to the reaction mixture, and let it stir for 16 hours at room temperature under air. Water (20 mL) was added to the reaction mixture and the emulsion extracted with Et_2O (3 x 20 mL), and the combined organic extracts were dried over sodium sulfate. The mixture was filtered, and the solvent was removed under vacuum. Purification via flash column chromatography (10–100% EtOAc in heptane, 5 g SiO_2), provided the methyl ester **B**, used for the next step.
In an oven–dried 30 mL microwave vial filled with Argon, ester **B** (1 mmol) dissolved in THF (2 mL) was added to a solution of RuCl(*p*-cymene)[(*S,S*)-Ts-DPEN (5 mol%) in 5 : 2 formic acid / triethylamine (2 mL) under Argon and the reaction mixture was let stir at 30 °C for 48 h. Over pressure was released with a needle at occurrence. After completion the reaction was quenched with saturated $NaHCO_3$ solution (15 mL) and extracted with DCM (3 x 20 mL). The combined organic extracts were dried over sodium sulfate. The mixture was filtered, the solvent was removed under vacuum, and the residue was purified via flash column chromatography (10–100% EtOAc in heptane, 5 g SiO_2), providing a non-separable mixture of alcohol **C** and lactone **D**.
In an oven dried 50 mL round-bottom flask, $LiAlH_4$ (0.5 equiv., 0.28 mL, 1M in THF) was dissolved in THF (1 mL), and cooled down with water/ice bath. A mixture of alcohol **C** and lactone **D** (approximately 0.5 mmol) was dissolved in THF (2 mL) and added dropwise to the $LiAlH_4$ solution, and the reaction mixture was let stir for 16 h at room temperature. After completion, the reaction mixture was cooled down with water/ice bath, saturated solution of NH_4Cl (10 mL) was added dropwise, then extracted with EtOAc (3 x 20 mL) and the combined organic extracts were dried over sodium sulfate. The mixture was filtered, and the solvent was removed under vacuum. The reaction crude was purified via flash column chromatography (10–100% EtOAc in heptane, 5 g SiO_2), providing the desired products.
For product (*S*)-**1k**, the reduction with $LiAlH_4$ was done at -20 °C instead of room temperature. The yields of each step are shown in the scheme above.

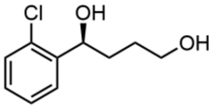

(*S*)-1-(2-chlorophenyl)butane-1,4-diol [(*S*)-1b]
The compound **(*S*)-1b** was obtained as a colorless oil (81.8 mg, 64.3% *ee*)
^{1}H NMR (500 MHz, $CDCl_3$): δ_H = 7.56 – 7.62 (m, 1H), 7.27 – 7.34 (m, 2H), 7.18 – 7.23 (m, 1H), 5.17 (ddd, 1H, *J*=7.7, 3.7, 1.4 Hz), 3.67 – 3.81 (m, 2H), 1.85 – 2.02 (m, 1H), 1.71 – 1.84 (m, 3H) ppm.
^{13}C NMR (126 MHz, $CDCl_3$): δ_C = 142.2, 131.8, 129.5, 128.5, 127.2, 127.2, 70.8, 63.1, 34.8, 29.2 ppm.
HRMS (ESI): *m/z* calc. for $C_{10}H_{12}ClO_2$ $[M-H^+]^-$: 199.0531; found: 199.0533.
The enantiomeric excess of the starting materials **(*S*)-1b** was determined with a Lux i-A3 (IG) column (150 x 4.6, particle size 3 μm) and a mobile phase of 15% EtOH/NH_3, 100/20mM in CO_2 120 bar, 3.5 ml/min, λ = 210 nm, 40 °C. Enantiomer 2 for **(*S*)-1b**, t_r = 2.188 min. (64.3% *ee*).
$[\alpha]^{20}_D$: –18.8 (c 0.1, $CHCl_3$).

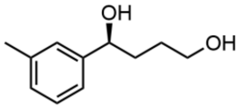

(*S*)-1-(m-tolyl)butane-1,4-diol [(*S*)-1g]

The compound **(*S*)-1g** was obtained as a colorless oil (91.8 mg, 89% *ee*)
^{1}H NMR (500 MHz, $CDCl_3$): δ_H = 7.23 (d, *J* = 7.5 Hz, 1H), 7.20 – 7.12 (m, 2H), 7.09 (d, *J* = 7.4 Hz, 1H), 4.71 (t, *J* = 6.4 Hz, 1H), 3.76 – 3.65 (m, 2H), 2.36 (s, 3H), 1.92 – 1.83 (m, 2H), 1.78 – 1.61 (m, 2H) ppm.
^{13}C NMR (126 MHz, $CDCl_3$): δ_C = 144.8, 138.3, 128.6, 128.5, 126.6, 123.0, 74.7, 63.1, 36.3, 29.4, 21.6 ppm.
The spectroscopic data is in agreement with those reported in the literature.[11]
The enantiomeric excess of the starting materials **(*S*)-1g** was determined with a Lux C2 column (150 x 4.6, particle size 3 μm) and a mobile phase of 20% EtOH/NH_3, 100/20mM in CO_2 120 bar, 3.5 ml/min, λ = 210 nm, 40 °C. Enantiomer 1 for **(*S*)-1g**, t_r = 1.473 min. (89.1% *ee*).
$[\alpha]^{20}_D$: –33.9 (c 0.33, $CHCl_3$).

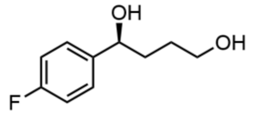

(*S*)-1-(4-fluorophenyl)butane-1,4-diol [(*S*)-1i]
The compound **(*S*)-1i** was obtained as a colorless oil (128 mg, 74% *ee*)
^{1}H NMR (500 MHz, $CDCl_3$): δ_H = 7.38 – 7.29 (m, 2H), 7.08 – 6.99 (m, 2H), 4.73 (t, *J* = 6.3 Hz, 1H), 3.76 – 3.65 (m, 2H), 1.90 – 1.81 (m, 2H), 1.77 – 1.59 (m, 2H) ppm.
^{13}C NMR (126 MHz, $CDCl_3$): δ_C = 163.3, 161.3, 140.6 (d, *J* = 3.2 Hz), 127.6 (d, *J* = 8.2 Hz), 115.4 (d, *J* = 21.2 Hz), 73.9, 63.0, 36.5, 29.2 ppm.
^{19}F NMR (470 MHz, $CDCl_3$): δ_F = –115.2 ppm.
The spectroscopic data is in agreement with those reported in the literature.[11]
The enantiomeric excess of the starting materials **(*S*)-1i** was determined with a Lux C2 column (150 x 4.6, particle size 3 μm) and a mobile phase of 15% IPA/NH_3, 100/20mM in CO_2 120 bar, 3.5 ml/min, λ = 210 nm, 40 °C. Enantiomer 1 for **(*S*)-1i** t_r = 1.812 min. (74% *ee*).
$[\alpha]^{20}_D$: –35.3 (c 1.00, $CHCl_3$).

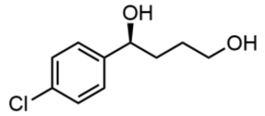

(*S*)-1-(4-chlorophenyl)butane-1,4-diol [(*S*)-1j]
The compound **(*S*)-1j** was obtained as a colorless oil (131 mg, 76% *ee*)
^{1}H NMR (500 MHz, $CDCl_3$): δ_H = 7.34 – 7.28 (m, 4H), 4.73 (dd, *J* = 7.1, 5.5 Hz, 1H), 3.76 – 3.65 (m, 2H), 1.91 – 1.77 (m, 2H), 1.76 – 1.59 (m, 2H) ppm.
^{13}C NMR (126 MHz, $CDCl_3$): δ_C = 143.3, 133.3, 128.7, 127.3, 73.8, 63.0, 36.5, 29.1 ppm.
The spectroscopic data is in agreement with those reported in the literature.[12]
The enantiomeric excess of the starting materials **(*S*)-1j** was determined with a Lux C2 column (150 x 4.6, particle size 3 μm) and a mobile phase of 20% IPA/NH_3, 100/20mM in CO_2 120 bar, 3.5 ml/min, λ = 210 nm, 40 °C. Enantiomer 1 for **(*S*)-1j** t_r = 1.871 min. (76% *ee*).
$[\alpha]^{20}_D$: –36.6 (c 1.00, $CHCl_3$).

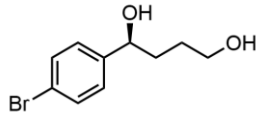

(*S*)-1-(4-bromophenyl)butane-1,4-diol [(*S*)-1k]
The compound **(*S*)-1k** was obtained as a colorless oil (131 mg, 79% *ee*)
^{1}H NMR (500 MHz, $CDCl_3$): δ_H = 7.50 – 7.43 (m, 2H), 7.25 – 7.22 (m, 2H), 4.70 (dd, *J* = 7.2, 5.4 Hz, 1H), 3.69 (hept, *J* = 5.4 Hz, 2H), 1.89 – 1.76 (m, 2H), 1.74 – 1.61 (m, 2H). ppm.
^{13}C NMR (126 MHz, $CDCl_3$): δ_C = 143.9, 131.6, 127.7, 121.3, 73.8, 62.9, 36.5, 29.1 ppm.

The spectroscopic data is in agreement with those reported in the literature.[13]
The enantiomeric excess of the starting materials **(*S*)-1k** was determined with a Chiralopak IF column (150 x 3, particle size 3 μm) and a mobile phase of 10% MeOH, 100/20mM in CO_2 120 bar, 0.8 ml/min, λ = 210 nm, 40 °C. Enantiomer 1 for **(*S*)-1k** t_r = 9.817 min. (80% *ee*).
$[\alpha]^{20}_D$: –35.3 (c 1.00, $CHCl_3$).

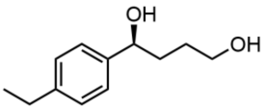

(*S*)-1-(4-ethylphenyl)butane-1,4-diol [(*S*)-1n]
The compound **(*S*)-1n** was obtained as a colorless oil (100 mg, 89.9% *ee*)
^{1}H NMR (500 MHz, $CDCl_3$): δ_H = 7.25 – 7.3 (m, 2H), 7.15 – 7.21 (m, 2H), 4.71 (dd, 1H, *J*=7.2, 5.5 Hz), 3.68 (hept, 2H, *J*=5.3 Hz), 2.64 (q, 2H, *J*=7.6 Hz), 2.35 (brs, 1H), 1.98 (brs, 1H), 1.8 – 1.93 (m, 2H), 1.59 – 1.77 (m, 2H), 1.23 (t, 3H, *J*=7.6 Hz) ppm.
^{13}C NMR (126 MHz, $CDCl_3$): δ_C = 143.8, 142.1, 128.1, 126.0, 74.5, 62.1, 36.2, 29.5, 28.6, 15.7 ppm.
HRMS (ESI): *m/z* calc. for $C_{12}H_{17}O_2$ $[M - H^+]^-$: 193.1234; found: 193.1235.
The enantiomeric excess of the starting materials **(*S*)-1n** was determined with a Lux C4 column (150 x 4.6, particle size 3 μm) and a mobile phase of 20% IPA/NH_3, 100/20mM in CO_2 120 bar, 3.5 ml/min, λ = 210 nm, 40 °C. Enantiomer 1 for **(*S*)-1n** t_r = 1.812 min. (89.9% *ee*).
$[\alpha]^{20}_D$: –44.1 (c 0.76, $CHCl_3$).

1.2.4 Procedure D

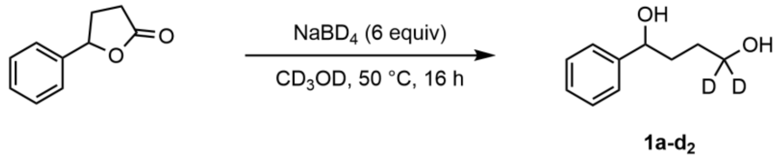

The compound **1a–d₂** was synthetized according to the following procedure. A two–neck oven–dried 100 mL round bottom flask was filled with nitrogen, deuterated methanol was added (CD_3OD, 12 mL) and the reaction flask was cooled down in an ice/water bath. Then $NaBD_4$ (1.084 g, 25.9 mmol, 6 equiv.) were carefully added to the cooled solution portion wise, keeping a constant nitrogen flow. A solution of 5–phenyldihydrofuran–2(3*H*)–one (700 mg, 4.32 mmol) in CD_3OD (8 mL, 0.2 M) was added dropwise, keeping the ice bath under the reaction flask and the nitrogen flow. After the addition the ice bath was removed, a condenser was put above the two–neck round bottom flask and the reaction mixture was heated to 50° C and let stir for 16 h. Then, the reaction mixture was cooled down first to room temperature, then was put in an ice bath. Saturated solution of NH_4Cl (10 mL) was added dropwise, then extracted with EtOAc (3 x 20 mL) and the combined organic extracts were dried over sodium sulfate. The mixture was filtered, and the solvent was removed under vacuum. Purification via flash column chromatography (10–100% EtOAc in heptane, 10 g SiO_2), provided the product **1a–d₂** (589.2 mg, 81% yield), >98% D incorporation.

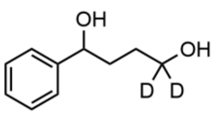

1-phenylbutane-4,4-d₂-1,4-diol (1a–d₂)
^{1}H NMR (500 MHz, $CDCl_3$): δ_H = 7.31 – 7.39 (m, 4H), 7.24 – 7.30 (m, 1H), 4.74 (t, 1H, *J*=6.3 Hz), 2.51 (brs, 1H), 1.98 (brs, 1H), 1.83 – 1.91 (m, 2H), 1.67 (qt, 2H, *J*=14.2, 7.2 Hz) ppm.

^{13}C NMR (126 MHz, $CDCl_3$): δ_C = 144.9, 128.6, 127.6, 125.9, 74.5, 62.21 (p, *J*=21.6 Hz), 36.4, 29.1 ppm. $CDCl_3$.
HRMS (ESI): *m/z* calc. for $C_{10}H_{12}D_2NaO_2$ $[M+Na]^+$: 191.1012 ; found: 191.1091.

1.3 Optimization

1.3.1 Solvent screening

Solvent screening was performed for the iridium catalyzed *N*-alkylation reaction. HFIP resulted to be the optimal one, as seen from Table S1.

Table S1: Solvent screening for the iridium catalyzed *N*-alkylation reaction.

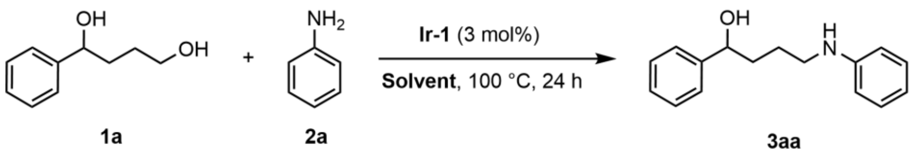

entry	**Solvent**	**3aa** (%) [a]
1	Toluene	-
2	THF	-
3	TFE	15
4	*i*-PrOH	13
5	H_2O	37
6	HFIP	49

The reaction was conducted in a pressure tube where the active catalyst is deposited and dried on its surface; 0.2 mmol of **2a** and 2 equiv. of **1a** were used in 0.5 mL of solvent. Reaction conditions. [a] Yield determined by ^{1}H NMR spectroscopy using 1,1,2,2-tetrachloroethane (TCE) as internal standard.

1.3.2 Byproduct evaluation

In the following Figure S1 a representative example of the NMR yield evaluation during the reaction profile using 1,1,2,2–tetrachloroethane (TCE) as internal standard (in yellow). Thus, it was possible to perform a reaction profile and carry on the optimization of the reaction.

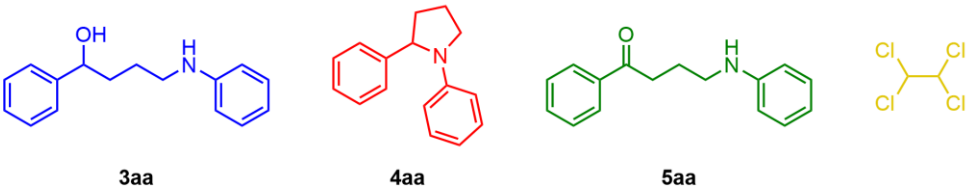

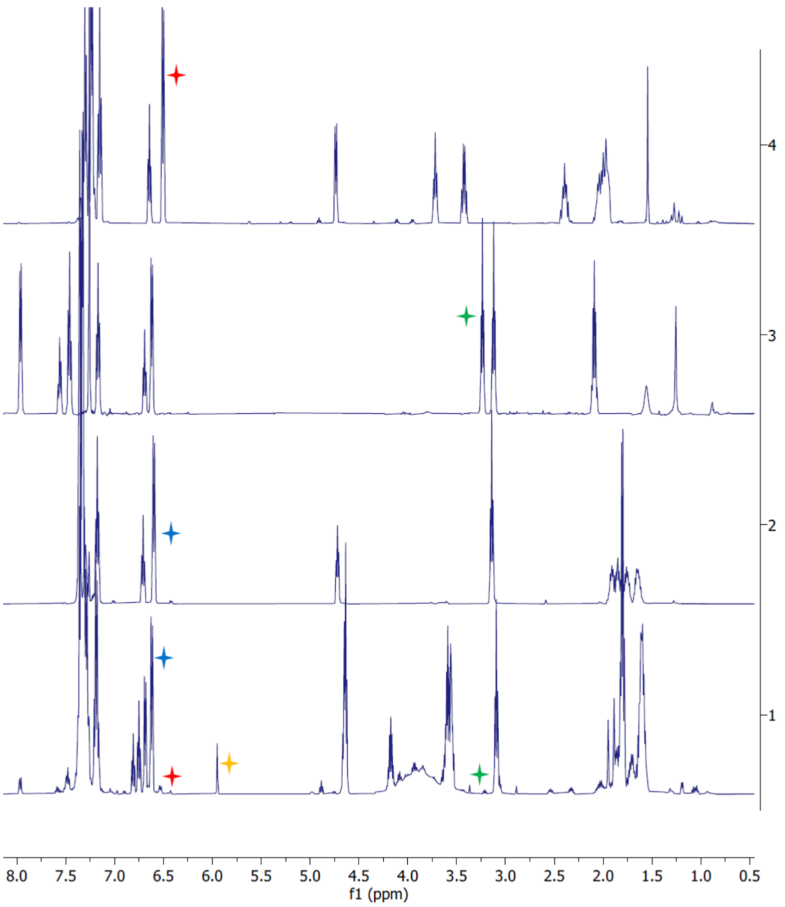

Figure S1: NMR evaluation of the *N*–alkylation of aniline with 1,4–diol (**1a**). [4]: ^{1}H NMR of the isolated byproduct **4aa** used for comparison. [3]: ^{1}H NMR of the isolated ketone **5aa** used for comparison. [2]: ^{1}H NMR of the desired product **3aa** used for comparison. [1]: Example of a crude NMR. The product is already present, and the reaction is *ongoing*. Traces of side products **4aa** and **5aa** can be identified.

1.3.3 Optimization table

Table S2: Full optimization for the *N*-alkylation of aniline **2a** with model 1,4-diol **1a**.[a]

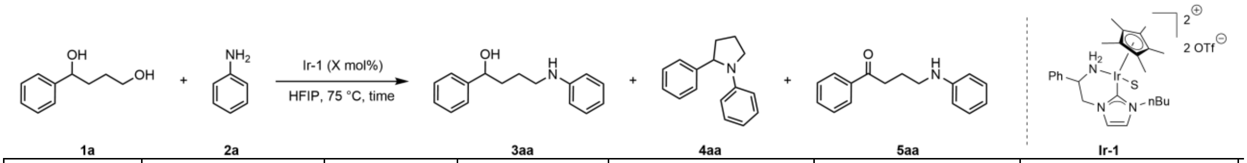

Entry	Cat (X mol%)	time (h)	1a (equiv.)	3aa yield (%)^b	4aa yield (%)^b	5aa yield (%)^b
1	2	24	2	38	1	4
2	2.5	24	2	68	2	3
3	3	24	2	73	3	2
4 ^{c}	3	24	2	55	22	4
5	3	48	2	76	8	8
6	3	24	1	52	2	5
7 ^{d}	3	24	0.5	55	3	5
8	3	24	3	77	3	2
**9**	**4**	**24**	**2**	**84**	**4**	**4**
10	4	24	3	86	5	5

Unless otherwise stated: 0.2 mmol of **2a** were use in dry and distilled HFIP (0.4 M) under inert atmosphere. **Ir-1** (mol% with respect to limiting reagent). ^{b}Yields determined by NMR spectroscopy with TCE internal standard. ^{c}100 °C instead of 75 °C. ^{d} The reaction was done with **1a** (0.2 mmol) and **2a** (0.4 mmol).

1.4 General procedure for the *N*-alkylation of anilines with 1,4-diols.

Iridium pre–catalyst iridium(III) chloride[14] was activated according to the following procedure.[15] In an oven dried 4 mL vial was covered with aluminum foil, then was charged with the iridium pre–catalyst (5.13 mg, 0.008 mmol, 1 equiv), silver salt AgOTf (4.11 mg, 0.016 mmol, 2 equiv) and dry DCM (0.5 mL). The suspension was left stirring for 30 minutes at room temperature. The mixture was then filtered off a pad of Celite and transferred to an oven dried pressure tube. The solvent was evaporated under reduced pressure and the activated catalyst was used *in–situ* under inert atmosphere.

The pressure tube impregnated with the activated catalyst (0.008 mmol, 4 mol%) closed with a septum under inert atmosphere was loaded with dry HFIP (0.5 mL, 0.4 M) and the corresponding aniline (0.2 mmol, 1 equiv.) and let stir for 5 minutes. The corresponding 1,4–diol (0.4 mmol, 2 equiv.) was added to the reaction mixture, the pressure tube was sealed under inert atmosphere and put in a pre–heated oil bath at 75 °C for 24 h. The pressure tube was then removed from the heating bath and let cool down, opened and the solvent was removed under reduced pressure. The yield was quantified by ^{1}H NMR spectroscopy using 1,1,2,2–tetrachloroethane (TCE) as internal standard, and the purification was performed by automatic flash column chromatography (0–100% EtOAc in heptane, 5 g SiO_2) or by preparative HPLC (20-100% MeCN in H_2O), giving the isolated yield. When preparative HPLC was used, the crude was dissolved prior in DMSO and let stir 2 hours in presence of excess of metal scavenger, then filtered before purification. Purification of δ-amino alcohols was proven to be challenging due to tailing during column chromatography.

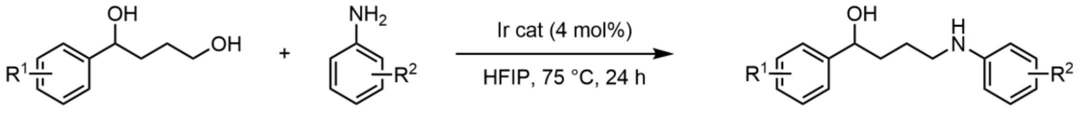

1.4.1 Scope of the 1,4-diols

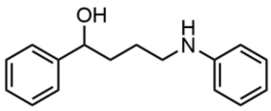

1–phenyl–4–(phenylamino)butan–1–ol (3aa)

The general procedure was applied using aniline (0.2 mmol) and 1–phenylbutane–1,4–diol (0.4 mmol, 2 equiv). Purification by automatic flash column chromatography (0–100% EtOAc in heptane, 5 g SiO_2), afforded the product **3aa** as colorless oil (34.7 mg, 72% yield).

^{1}H NMR (400 MHz, $CDCl_3$): δ_H = 7.38 – 7.27 (m, 5H), 7.22 – 7.12 (m, 2H), 6.70 (tt, *J* = 7.3, 1.1 Hz, 1H), 6.63 – 6.55 (m, 2H), 4.73 (dd, *J* = 7.5, 5.4 Hz, 1H), 3.14 (t, *J* = 6.9 Hz, 2H), 1.99 – 1.59 (m, 4H) ppm.

^{13}C NMR (126 MHz, $CDCl_3$): δ_C = 148.3, 144.7, 129.3, 128.5, 127.6, 125.9, 117.4, 113.0, 74.2, 44.0, 36.6, 25.8 ppm.

HRMS (ESI): *m/z* calc. for $C_{16}H_{20}NO$ $[M+H]^+$: 242.1539 ; found: 242.1541.

The general procedure was applied for the synthesis of **3aa** at 1 mmol scale.

In a pressure tube impregnated with the activated catalyst (0.04 mmol, 4 mol%) under inert atmosphere, aniline (1 mmol) and 1–phenylbutane–1,4–diol (2 mmol, 2 equiv) were dissolved in HFIP (2.5 mL, 0.4 M). The product **3aa** (168.0 mg, 70% yield) was purified by automatic flash column chromatography (0–100% EtOAc in heptane, 10 g SiO_2).

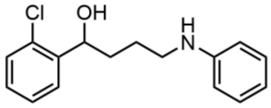

1–(2–chlorophenyl)–4–(phenylamino)butan–1–ol (3ba)
The general procedure was applied using aniline (0.2 mmol) and 1–(2–chlorophenyl)butane–1,4–diol (0.4 mmol, 2 equiv), with 3 mol% of **Ir-1** catalyst instead of 4 mol%. Purification by automatic flash column chromatography (0–100% EtOAc in heptane, 5 g SiO_2), afforded the product **3ba** as brown oil (46.7 mg, 84% yield).
^{1}H NMR (500 MHz, $CDCl_3$): δ_H = 7.57 (dd, J = 7.8, 1.7 Hz, 1H), 7.37 – 7.27 (m, 2H), 7.24 – 7.14 (m, 3H), 6.71 (td, J = 7.2, 1.3 Hz, 1H), 6.65 – 6.59 (m, 2H), 5.16 (dd, J = 7.4, 4.2 Hz, 1H), 3.38 – 2.75 (m, 4H), 1.96 – 1.70 (m, 4H) ppm.
^{13}C NMR (126 MHz, $CDCl_3$): δ_C = 148.3, 142.0, 131.8, 129.4, 129.3, 128.5, 127.2, 127.1, 117.5, 113.1, 70.4, 44.0, 35.2, 25.9 ppm.
HRMS (ESI): *m/z* calc. for $C_{16}H_{19}ClNO$ $[M+H]^+$: 276.1150; found: 276.1164.

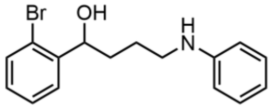

1-(2-bromophenyl)-4-(phenylamino)butan-1-ol (3ca)
The general procedure was applied using aniline (0.2 mmol) and 1–(2–bromophenyl)butane–1,4–diol (0.4 mmol, 2 equiv). Purification by automatic flash column chromatography (0–100% EtOAc in heptane, 5 g SiO_2), afforded the product **3ca** as brown oil (53.3 mg, 83% yield).
^{1}H NMR (500 MHz, $CDCl_3$): δ_H = 7.48 – 7.59 (m, 2H), 7.34 (td, 1H, J=7.5, 1.2 Hz), 7.16 – 7.21 (m, 2H), 7.13 (td, 1H, J=7.6, 1.7 Hz), 6.73 (tt, 1H, J=7.3, 1.1 Hz), 6.61 – 6.67 (m, 2H), 5.06 – 5.14 (m, 1H), 3.12 – 3.25 (m, 2H), 3.04 (brs, 1H), 1.73 – 1.97 (m, 4H) ppm.
^{13}C NMR (126 MHz, $CDCl_3$): δ_C = 148.1, 143.7, 132.8, 129.4, 129.0, 127.9, 127.4, 122.0, 117.9, 113.4, 72.7, 44.2, 35.3, 25.9 ppm.
HRMS (ESI): *m/z* calc. for $C_{16}H_{19}BrNO$ $[M+H]^+$: 320.0645; found: 320.0652.

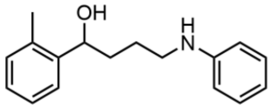

4–(phenylamino)–1–(o–tolyl)butan–1–ol (3da)
The general procedure was applied using aniline (0.2 mmol) and 1–(o–tolyl)butane–1,4–diol (0.4 mmol, 2 equiv), with 3 mol% of **Ir-1** catalyst instead of 4 mol%. Purification by automatic flash column chromatography (0–100% EtOAc in heptane, 5 g SiO_2), afforded the product **3da** obtained as pale brown amorphous solid (41.9 mg, 82% yield). M.p:71-76°C.
^{1}H NMR (500 MHz, $CDCl_3$): δ_H = 7.51 (dd, 1H, J=7.7, 1.5 Hz), 7.25 – 7.30 (m, 1H), 7.17 – 7.24 (m, 4H), 6.74 (tt, 1H, J=7.3, 1.1 Hz), 6.64 (dq, 2H, J=6.9, 1.5 Hz), 4.99 (dd, 1H, J=7.5, 4.4 Hz), 3.14 – 3.24 (m, 2H), 2.37 (s, 3H), 1.69 – 1.94 (m, 4H) ppm.
^{13}C NMR (126 MHz, $CDCl_3$): δ_C = 148.3, 142.8, 134.4, 130.5, 129.3, 127.3, 126.4, 125.2, 117.4, 112.9, 70.5, 44.1, 35.6, 26.1, 19.1 ppm.
HRMS (ESI): *m/z* calc. for $C_{17}H_{22}NO$ $[M+H]^+$: 256.1696; found: 256.1708.

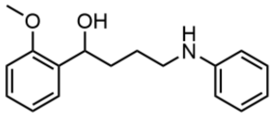

1–(2–methoxyphenyl)–4–(phenylamino)butan–1–ol (3ea)

The general procedure was applied using aniline (0.2 mmol) and 1–(2–methoxyphenyl)butane–1,4–diol (0.4 mmol, 2 equiv). The reaction temperature was 65 °C. Purification by automatic flash column chromatography (0–100% EtOAc in heptane, 5 g SiO_2), afforded the product **3ea** as brown oil (43.1 mg, 79% yield).
^{1}H NMR (500 MHz, $CDCl_3$): δ_H = 7.32 (dd, 1H, *J*=7.5, 1.7 Hz), 7.22 – 7.27 (m, 1H), 7.12 – 7.19 (m, 2H), 6.96 (td, 1H, *J*=7.5, 1.1 Hz), 6.89 (dd, 1H, *J*=8.2, 1.0 Hz), 6.68 (tt, 1H, *J*=7.3, 1.1 Hz), 6.57 – 6.62 (m, 2H), 4.92 (dd, 1H, *J*=7.7, 5.4 Hz), 3.85 (s, 3H), 3.16 (t, 2H, *J*=6.9 Hz), 1.75 – 1.98 (m, 3H), 1.69 (ddq, 1H, *J*=13.4, 9.3, 6.6 Hz) ppm.
^{13}C NMR (126 MHz, $CDCl_3$): δ_C = 156.5, 148.5, 132.4, 129.3, 128.4, 126.9, 120.9, 117.2, 112.9, 110.6, 70.5, 55.3, 44.0, 34.8, 26.1 ppm.
HRMS (ESI): *m/z* calc. for $C_{17}H_{22}NO_2$ $[M+H]^+$: 272.1645; found: 272.1644.

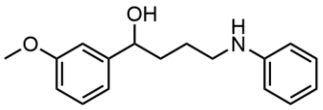

1–(3–methoxyphenyl)–4–(phenylamino)butan–1–ol (3fa)
The general procedure was applied using aniline (0.2 mmol) and 1–(3–methoxyphenyl)butane–1,4–diol (0.4 mmol, 2 equiv). Purification by automatic flash column chromatography (0–100% EtOAc in heptane, 5 g SiO_2), afforded the product **3fa** as pink oil (33 mg, 61% yield).
^{1}H NMR (500 MHz, $CDCl_3$): δ_H = 7.23 – 7.31 (m, 1H), 7.12 – 7.21 (m, 2H), 6.90 – 6.95 (m, 2H), 6.83 (ddd, 1H, *J*=8.3, 2.5, 1.1 Hz), 6.71 (tt, 1H, *J*=7.3, 1.1 Hz), 6.55 – 6.63 (m, 2H), 4.68 (dd, 1H, *J*=7.5, 5.4 Hz), 3.82 (s, 3H), 3.13 (t, 2H, *J*=6.9 Hz), 1.58 – 1.95 (m, 4H) ppm.
^{13}C NMR (126 MHz, $CDCl_3$): δ_C = 159.8, 148.3, 146.4, 129.6, 129.2, 118.2, 117.4, 113.0, 112.9, 111.4, 74.2, 55.2, 43.9, 36.6, 25.9 ppm.
HRMS (ESI): *m/z* calc. for $C_{17}H_{22}NO_2$ $[M+H]^+$: 272.1645; found: 272.1644.

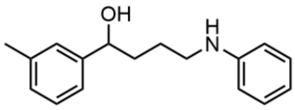

1–(3–methylphenyl)–4–(phenylamino)butan–1–ol (3ga)
The general procedure was applied using aniline (0.2 mmol) and 1–(3–methylphenyl)butane–1,4–diol (0.4 mmol, 2 equiv). Purification by automatic flash column chromatography (0–100% EtOAc in heptane, 5 g SiO_2), afforded the product **3ga** obtained as brown oil (37.9 mg, 74% yield).
^{1}H NMR (500 MHz, $CDCl_3$): δ_H = 7.28 (t, *J* = 7.5 Hz, 1H), 7.23 – 7.12 (m, 5H), 6.75 (tt, *J* = 7.3, 1.1 Hz, 1H), 6.69 – 6.61 (m, 2H), 4.70 (dd, *J* = 7.6, 5.3 Hz, 1H), 3.17 (t, *J* = 6.9 Hz, 2H), 2.40 (s, 3H), 1.97 – 1.74 (m, 3H), 1.72 – 1.62 (m, 1H). ppm.
^{13}C NMR (126 MHz, $CDCl_3$): δ_C = 148.2, 144.6, 138.3, 129.4, 128.5, 128.5, 126.7, 123.0, 117.7, 113.2, 74.4, 44.2, 36.7, 26.0, 21.6 ppm.
HRMS (ESI): *m/z* calc. for $C_{17}H_{22}NO$ $[M+H]^+$: 256.1696; found: 256.1690.

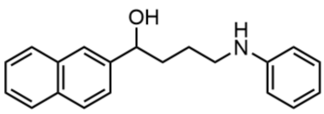

1–(naphthalen–2–yl)–4–(phenylamino)butan–1–ol (3ha)
The general procedure was applied using aniline (0.2 mmol) and 1–(naphthalen–2–yl)butane–1,4–diol (0.4 mmol, 2 equiv), with 3 mol% of **Ir-1** catalyst instead of 4 mol%. Purification by automatic flash column chromatography (0–100% EtOAc in heptane, 5 g SiO_2), afforded the product **3ha** as pale yellow oil (37 mg, 63% yield).

^{1}H NMR (500 MHz, $CDCl_3$): δ_H = 7.79 – 7.88 (m, 3H), 7.78 (d, 1H, *J*=1.7 Hz), 7.45 – 7.54 (m, 3H), 7.14 – 7.22 (m, 2H), 6.72 (tt, 1H, *J*=7.2, 1.1 Hz), 6.56 – 6.63 (m, 2H), 4.87 (dd, 1H, *J*=7.4, 5.6 Hz), 3.14 (t, 2H, *J*=6.9 Hz), 1.87 – 2.04 (m, 2H), 1.72 – 1.82 (m, 1H), 1.65 (ddq, 1H, *J*=13.3, 9.6, 6.7 Hz) ppm.
^{13}C NMR (126 MHz, $CDCl_3$): δ_C = 148.4, 142.0, 133.4, 133.1, 129.4, 128.5, 128.0, 127.8, 126.3, 126.0, 124.7, 124.1, 117.5, 113.0, 74.5, 44.1, 36.6, 26.0 ppm.
HRMS (ESI): *m/z* calc. for $C_{20}H_{22}NO$ $[M+H]^+$: 292.1696; found: 292.1697.

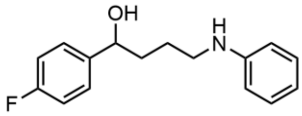

1–(4–fluorophenyl)–4–(phenylamino)butan–1–ol (3ia)
The general procedure was applied using aniline (0.2 mmol) and 1–(4–fluorophenyl)butane–1,4–diol (0.4 mmol, 2 equiv). Purification by automatic flash column chromatography (0–100% EtOAc in heptane, 5 g SiO_2), afforded the product **3ia** as colorless oil (35.5 mg, 68% yield).
^{1}H NMR (500 MHz, $CDCl_3$): δ_H = 7.28 – 7.36 (m, 2H), 7.13 – 7.21 (m, 2H), 6.99 – 7.08 (m, 2H), 6.70 (tt, 1H, *J*=7.3, 1.1 Hz), 6.56 – 6.63 (m, 2H), 4.72 (dd, 1H, *J*=7.6, 5.3 Hz), 3.15 (t, 2H, *J*=6.8 Hz), 1.69 – 1.94 (m, 3H), 1.58 – 1.68 (m, 1H) ppm.
^{13}C NMR (126 MHz, $CDCl_3$): δ_C = 162.21 (d, *J*=245.6 Hz), 148.2, 140.37 (d, *J*=3.0 Hz), 129.3, 127.52 (d, *J*=8.0 Hz), 117.6, 115.33 (d, *J*=21.4 Hz), 113.0, 73.7, 44.0, 36.8, 25.9 ppm.
^{19}F NMR (470 MHz, $CDCl_3$): δ_F = –115.0 ppm
HRMS (ESI): *m/z* calc. for $C_{16}H_{19}FNO$ $[M+H]^+$: 260.1445; found: 260.1445.

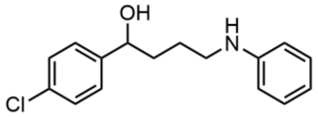

1–(4–chlorophenyl)–4–(phenylamino)butan–1–ol (3ja)
The general procedure was applied using aniline (0.2 mmol) and 1–(4–chlorophenyl)butane–1,4–diol (0.4 mmol, 2 equiv). Purification by automatic flash column chromatography (0–100% EtOAc in heptane, 5 g SiO_2), afforded the product **3ja** as brown oil (44.7 mg, 80% yield).
^{1}H NMR (500 MHz, $CDCl_3$): δ_H = 7.36 – 7.33 (m, 2H), 7.32 – 7.28 (m, 2H), 7.22 – 7.18 (m, 2H), 6.74 (tt, *J* = 7.3, 1.1 Hz, 1H), 6.66 – 6.60 (m, 2H), 4.73 (dd, *J* = 7.6, 5.2 Hz, 1H), 3.17 (t, *J* = 6.8 Hz, 2H), 1.94 – 1.72 (m, 3H), 1.71 – 1.62 (m, 1H) ppm.
^{13}C NMR (126 MHz, $CDCl_3$): δ_C = 148.2, 143.2, 133.4, 129.4, 128.8, 127.4, 117.8, 113.2, 73.7, 44.2, 36.9, 25.9 ppm.
HRMS (ESI): *m/z* calc. for $C_{16}H_{17}ClNO$ $[M-H^+]^-$: 274.0999; found: 274.0990.

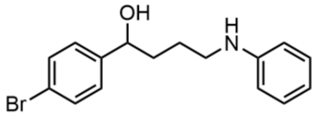

1–(4–bromophenyl)–4–(phenylamino)butan–1–ol (3ka)
The general procedure was applied using aniline (0.2 mmol) and 1–(4–bromophenyl)butane–1,4–diol (0.4 mmol, 2 equiv). Purification by automatic flash column chromatography (0–100% EtOAc in heptane, 5 g SiO_2), afforded the product **3ka** as yellow oil (47.8 mg, 75% yield).
^{1}H NMR (500 MHz, $CDCl_3$): δ_H = 7.51 – 7.44 (m, 2H), 7.26 – 7.20 (m, 2H), 7.21 – 7.13 (m, 2H), 6.71 (tt, *J* = 7.3, 1.1 Hz, 1H), 6.63 – 6.56 (m, 2H), 4.70 (dd, *J* = 7.6, 5.2 Hz, 1H), 3.15 (t, *J* = 6.8 Hz, 2H), 1.92 – 1.69 (m, 3H), 1.65 (ddt, *J* = 13.2, 9.9, 6.4 Hz, 1H) ppm.

^{13}C NMR (126 MHz, $CDCl_3$): δ_C = 148.1, 143.7, 131.8, 129.4, 127.7, 121.5, 117.9, 113.3, 73.8, 44.3, 36.9, 25.9 ppm.
HRMS (ESI): *m/z* calc. for $C_{16}H_{19}BrNO$ $[M+H]^+$: 320.0645; found: 320.0661.

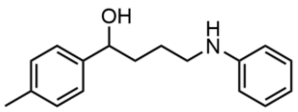

4–(phenylamino)–1–(p–tolyl)butan–1–ol (3la)
The general procedure was applied using aniline (0.2 mmol) and 1–(p–tolyl)butane–1,4–diol (0.4 mmol, 2 equiv). Purification by automatic flash column chromatography (0–100% EtOAc in heptane, 5 g SiO_2), afforded the product **3la** as brown amorphous solid (33.4 mg, 65% yield). M.p.: 58-62 °C.
^{1}H NMR (500 MHz, $CDCl_3$): δ_H = 7.24 (d, 2H, *J*=8.1 Hz), 7.12 – 7.20 (m, 4H), 6.69 (tt, 1H, *J*=7.3, 1.1 Hz), 6.56 – 6.64 (m, 2H), 4.69 (dd, 1H, *J*=7.5, 5.5 Hz), 3.14 (t, 2H, *J*=6.9 Hz), 2.35 (s, 3H), 1.69 – 1.97 (m, 3H), 1.64 (ddt, 1H, *J*=13.2, 10.2, 6.2 Hz) ppm.
^{13}C NMR (126 MHz, $CDCl_3$): δ_C = 148.4, 141.6, 137.4, 129.3, 129.2, 125.9, 117.4, 112.9, 74.2, 44.0, 36.6, 26.0, 21.2 ppm.
HRMS (ESI): *m/z* calc. for $C_{17}H_{22}NO$ $[M+H]^+$: 256.1696; found: 256.1708.

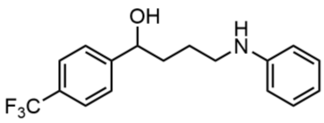

4–(phenylamino)–1–(4–(trifluoromethyl)phenyl)butan–1–ol (3ma)
The general procedure was applied using aniline (0.2 mmol) and 1–(4–(trifluoromethyl)phenyl)butane–1,4–diol (0.4 mmol, 2 equiv). Purification by automatic flash column chromatography (0–100% EtOAc in heptane, 5 g SiO_2), afforded the product **3ma** as brown oil (42.8 mg, 69% yield).
^{1}H NMR (500 MHz, $CDCl_3$): δ_H = 7.63 (d, 2H, *J*=8.1 Hz), 7.47 (d, 2H, *J*=8.0 Hz), 7.17 – 7.25 (m, 2H), 6.76 (tt, 1H, *J*=7.3, 1.1 Hz), 6.60 – 6.66 (m, 2H), 4.78 (dd, 1H, *J*=7.4, 5.3 Hz), 3.17 (t, 2H, *J*=6.8 Hz), 1.82 – 1.94 (m, 2H), 1.63 – 1.82 (m, 2H) ppm.
^{13}C NMR (126 MHz, $CDCl_3$): δ_C = 148.6, 148.2, 129.7, 129.3, 125.44 (q, *J*=3.9 Hz), 124.2 (q, *J*=272.0 Hz), 117.8, 113.2, 73.6, 44.0, 36.9, 25.8 ppm.
^{19}F NMR (470 MHz, $CDCl_3$): δ_F = –62.4 ppm
HRMS (ESI): *m/z* calc. for $C_{17}H_{19}F_3NO$ $[M+H]^+$: 310.1413; found: 310.1433.

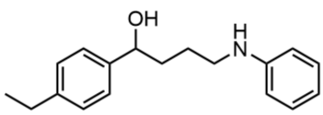

1-(4-ethylphenyl)-4-(phenylamino)butan-1-ol (3na)
The general procedure was applied using aniline (0.2 mmol) and 1-(4-ethylphenyl)butane-1,4-diol (0.4 mmol, 2 equiv). Purification by automatic flash column chromatography (0–100% EtOAc in heptane, 5 g SiO_2), afforded the product **3na** as yellow oil (33.0 mg, 61% yield).
^{1}H NMR (500 MHz, $CDCl_3$): δ_H = 7.27 (d, *J* = 7.2 Hz, 2H), 7.22 – 7.12 (m, 4H), 6.69 (dd, *J* = 7.9, 6.8 Hz, 1H), 6.61 – 6.56 (m, 2H), 4.70 (dd, *J* = 7.5, 5.5 Hz, 1H), 3.14 (t, *J* = 6.9 Hz, 2H), 2.65 (q, *J* = 7.6 Hz, 2H), 1.97 – 1.70 (m, 3H), 1.64 (ddq, *J* = 13.2, 9.9, 6.5 Hz, 1H), 1.24 (t, *J* = 7.6 Hz, 3H) ppm.
^{13}C NMR (126 MHz, $CDCl_3$): δ_C = 148.5, 143.9, 141.9, 129.3, 128.1, 126.0, 117.4, 113.0, 74.3, 44.0, 36.6, 28.7, 26.1, 15.7 ppm.
HRMS (ESI): *m/z* calc. for $C_{18}H_{22}N$ $[(M - H_2O) +H]^+$: 252.1747; found: 252.1757.

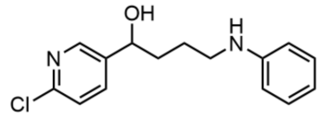

1-(6-chloropyridin-3-yl)-4-(phenylamino)butan-1-ol (3oa)
The general procedure was applied using aniline (0.2 mmol) and (6-chloropyridin-3-yl)butane-1,4-diol (0.4 mmol, 2 equiv). Purification by automatic flash column chromatography (0–100% EtOAc in heptane, 5 g SiO_2), afforded the product **3oa** as pale brown oil (40.3 mg, 73% yield).
^{1}H NMR (500 MHz, $CDCl_3$): δ_H = 8.34 (d, 1H, *J*=2.5 Hz), 7.67 (dd, 1H, *J*=8.2, 2.5 Hz), 7.31 (d, 1H, *J*=8.2 Hz), 7.18 (tt, 2H, *J*=7.4, 1.1 Hz), 6.73 (td, 1H, *J*=7.3, 1.0 Hz), 6.58 – 6.64 (m, 2H), 4.78 (dd, 1H, *J*=7.7, 5.1 Hz), 3.17 (t, 2H, *J*=6.7 Hz), 1.63 – 1.95 (m, 4H) ppm.
^{13}C NMR (126 MHz, $CDCl_3$): δ_C = 150.7, 148.1, 147.7, 139.0, 136.6, 129.4, 124.3, 118.1, 113.3, 71.4, 44.1, 37.0, 25.9 ppm.
HRMS (ESI): *m/z* calc. for $C_{15}H_{18}ClN_2O$ $[M+H]^+$: 277.1102; found: 277.1097.

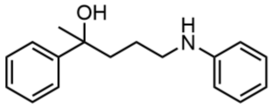

2-phenyl-5-(phenylamino)pentan-2-ol (3pa)
The general procedure was applied using aniline (0.2 mmol) and 4-phenylpentane-1,4-diol (0.4 mmol, 2 equiv). Purification by automatic flash column chromatography (0–100% EtOAc in heptane, 5 g SiO_2), afforded the product **3pa** as dark yellow oil (31.8 mg, 61% yield).
^{1}H NMR (500 MHz, $CDCl_3$): δ_H = 7.41 – 7.48 (m, 2H), 7.32 – 7.37 (m, 2H), 7.22 – 7.27 (m, 1H), 7.11 – 7.20 (m, 2H), 6.69 (tt, 1H, *J*=7.3, 1.1 Hz), 6.53 – 6.59 (m, 2H), 3.06 (td, 2H, *J*=6.9, 2.1 Hz), 1.86 – 2.03 (m, 2H), 1.44 – 1.66 (m, 6H) ppm.
^{13}C NMR (126 MHz, $CDCl_3$): δ_C = 148.4, 147.8, 129.3, 128.3, 126.7, 124.9, 117.6, 113.1, 74.5, 44.4, 41.8, 30.7, 24.4 ppm.
HRMS (ESI): *m/z* calc. for $C_{17}H_{20}N$ $[(M - H_2O) +H]^+$: 238.1590; found: 238.1603.

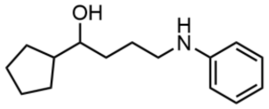

1-cyclopentyl-4-(phenylamino)butan-1-ol (3qa)
The general procedure was applied using aniline (0.2 mmol) and 1-cyclopentylbutane-1,4-diol (0.4 mmol, 2 equiv). Purification by automatic flash column chromatography (0–100% EtOAc in heptane, 5 g SiO_2), afforded the product **3qa** as white amorphous solid (27.5 mg, 59% yield). M.p.: 70-71 °C.
^{1}H NMR (500 MHz, $CDCl_3$): δ_H = 7.15 – 7.23 (m, 2H), 6.75 (t, 1H, *J*=7.3 Hz), 6.67 – 6.71 (m, 2H), 3.44 (ddd, 1H, *J*=9.4, 7.4, 2.9 Hz), 3.17 (qt, 2H, *J*=12.0, 6.8 Hz), 1.43 – 1.94 (m, 13H), 1.34 (dq, *J* = 12.1, 8.0 Hz, 1H), 1.14 – 1.27 (m, 1H) ppm.
^{13}C NMR (126 MHz, $CDCl_3$): δ_C = 148.4, 129.4, 117.6, 113.2, 75.9, 46.7, 44.5, 33.9, 29.3, 28.7, 26.1, 25.9, 25.7 ppm.
HRMS (ESI): *m/z* calc. for $C_{15}H_{24}NO$ $[M+H]^+$: 234.1852; found: 234.1859.

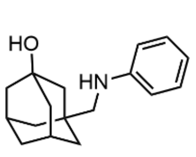

(1s,3r,5R,7S)-3-((phenylamino)methyl)adamantan-1-ol (3ra)

The general procedure was applied using aniline (0.2 mmol) and (1s,3r,5R,7S)-3-(hydroxymethyl)adamantan-1-ol (0.4 mmol, 2 equiv). Purification by preparative HPLC (20-100% MeCN in H_2O), afforded the product **3ra** as colorless oil (37.5 mg, 73% yield).
^{1}H NMR (500 MHz, $CDCl_3$): δ_H = 7.13 – 7.19 (m, 2H), 6.61 – 6.71 (m, 3H), 2.90 (s, 2H), 2.24 (p, 2H, *J*=3.1 Hz), 1.64 – 1.75 (m, 4H), 1.46 – 1.61 (m, 8H) ppm.
^{13}C NMR (126 MHz, $CDCl_3$): δ_C = 148.9, 129.4, 117.3, 113.0, 69.0, 55.6, 48.4, 44.9, 39.5, 38.0, 35.7, 30.6 ppm.
HRMS (ESI): *m/z* calc. for $C_{17}H_{24}NO$ $[M+H]^+$: 258.1852; found: 258.1862.

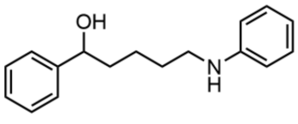

1-phenyl-5-(phenylamino)pentan-1-ol (3sa)
The general procedure was applied using aniline (0.2 mmol) and 1-phenylpentane-1,5-diol (0.4 mmol, 2 equiv). Purification by automatic flash column chromatography (0–100% EtOAc in heptane, 5 g SiO_2), afforded the product **3sa** as brown oil (34.7 mg, 68% yield)
^{1}H NMR (500 MHz, $CDCl_3$): δ_H = 7.27 – 7.40 (m, 5H), 7.14 – 7.22 (m, 2H), 6.70 (t, 1H, *J*=7.3 Hz), 6.56 – 6.62 (m, 2H), 4.68 (dd, 1H, *J*=7.5, 5.7 Hz), 3.10 (t, 2H, *J*=7.0 Hz), 1.71 – 1.91 (m, 2H), 1.61 – 1.69 (m, 2H), 1.35 – 1.60 (m, 2H). ppm.
^{13}C NMR (126 MHz, $CDCl_3$): δ_C = 148.5, 144.8, 129.3, 128.6, 127.7, 126.0, 117.3, 112.8, 74.6, 43.9, 38.9, 29.5, 23.5 ppm.
HRMS (ESI): *m/z* calc. for $C_{17}H_{20}N$ $[(M - H_2O) +H]^+$: 238.1590; found: 238.1611.

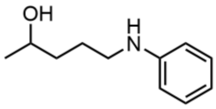

5-(phenylamino)pentan-2-ol (3ta)
The general procedure was applied using aniline (0.2 mmol) and pentane-1,4-diol (0.4 mmol, 2 equiv). Purification by automatic flash column chromatography (0–100% EtOAc in heptane, 5 g SiO_2), afforded the product **3ta** as pale brown oil (28.3 mg, 79% yield).
^{1}H NMR (400 MHz, $CDCl_3$): δ_H = 7.23 – 7.13 (m, 2H), 6.71 (tt, *J* = 7.3, 1.1 Hz, 1H), 6.62 (dd, *J* = 8.5, 1.2 Hz, 2H), 3.84 (h, *J* = 6.2 Hz, 1H), 3.14 (t, *J* = 6.9 Hz, 2H), 2.67 (brs, 2H), 1.84 – 1.60 (m, 2H), 1.63 – 1.49 (m, 2H), 1.22 (d, *J* = 6.2 Hz, 3H) ppm.
^{13}C NMR (101 MHz, $CDCl_3$): δ_C = 148.4, 129.4, 117.5, 113.0, 67.9, 44.2, 36.9, 26.0, 23.8 ppm.
The spectroscopic data is in agreement with those reported in the literature.[16]

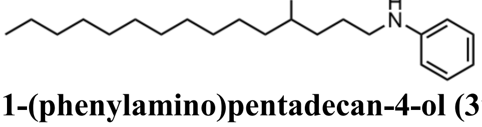

1-(phenylamino)pentadecan-4-ol (3ua)
The general procedure was applied using aniline (0.2 mmol) and pentane-1,4-diol (0.4 mmol, 2 equiv). Purification by automatic flash column chromatography (0–100% EtOAc in heptane, 5 g SiO_2), afforded the product **3ta** as white amorphous solid (55.6 mg, 87% yield). M.p.: 55-56 °C.
^{1}H NMR (400 MHz, $CDCl_3$): δ_H = 7.18 (t, *J* = 7.7 Hz, 2H), 6.70 (t, *J* = 7.3 Hz, 1H), 6.62 (d, *J* = 8.0 Hz, 2H), 3.63 (tt, *J* = 8.5, 4.2 Hz, 1H), 3.15 (t, *J* = 6.9 Hz, 2H), 1.84 – 1.38 (m, 6H), 1.28 (d, *J* = 7.7 Hz, 19H), 0.89 (t, *J* = 6.7 Hz, 3H). ppm.

^{13}C NMR (101 MHz, $CDCl_3$): δ_C = 148.5, 129.4, 117.5, 113.0, 71.9, 44.3, 37.8, 35.1, 32.1, 29.8, 29.8, 29.8, 29.5, 26.0, 25.8, 22.8, 14.3 ppm.
HRMS (ESI): *m/z* calc. for $C_{21}H_{37}NO$ [M +H]$^+$: 320.2947; found: 320.2948.

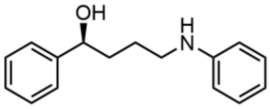

(*S*)-1-phenyl-4-(phenylamino)butan-1-ol [(*S*)-3aa]
The general procedure was applied using aniline (0.1 mmol) and (*S*)-1-phenylbutane-1,4-diol (0.2 mmol, 2 equiv). Purification by automatic flash column chromatography (0–100% EtOAc in heptane, 5 g SiO_2), afforded the product **(*S*)-3aa** as colorless oil (18.1 mg, 75% yield, 97% *ee*).
^{1}H NMR (500 MHz, $CDCl_3$): δ_H = 7.35 (d, 4H, *J*=4.4 Hz), 7.28 (ddd, 1H, *J*=8.6, 5.1, 3.8 Hz), 7.13 – 7.23 (m, 2H), 6.76 (tt, 1H, *J*=7.3, 1.1 Hz), 6.65 – 6.71 (m, 2H), 4.72 (dd, 1H, *J*=7.6, 5.1 Hz), 3.16 (td, 2H, *J*=6.8, 1.0 Hz), 1.74 – 1.97 (m, 3H), 1.68 (dddd, 1H, *J*=15.3, 13.1, 5.5, 4.1 Hz) ppm.
^{13}C NMR (126 MHz, $CDCl_3$): δ_C = 147.3, 144.6, 129.6, 128.7, 127.8, 126.0, 118.6, 114.0, 74.4, 45.0, 36.7, 25.8 ppm.
HRMS (ESI): *m/z* calc. for $C_{16}H_{20}NO$ [M+H]$^+$: 242.1539; found: 242.1540.
The enantiopurity of the products (*S*)-**3aa** were determined with Shimadzu SFC Prep equipped with Chiralpak IN column (150 x 4.6, particle size 3 μm) and a mobile phase of 20% MeOH/NH3 100/20mM in CO_2 120 bar, 3.5 ml/min, λ = 240 nm, 40 °C. Enantiomer 2 t_r = 1.882 min.
$[\alpha]^{20}_D$: –21.8 (c 0.26, $CHCl_3$).

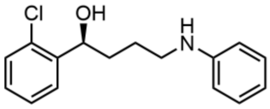

(*S*)-1-(2-chlorophenyl)-4-(phenylamino)butan-1-ol [(*S*)-3ba]
The general procedure was applied using aniline (0.2 mmol) and (*S*)-1-(2-chlorophenyl)butane-1,4-diol (0.4 mmol, 2 equiv). Purification by automatic flash column chromatography (0–100% EtOAc in heptane, 5 g SiO_2), afforded the product **(*S*)-3ba** as colorless oil (45.7 mg, 83% yield, 55% *ee*).
^{1}H NMR (500 MHz, $CDCl_3$): δ_H = 7.57 (dd, 1H, *J*=7.8, 1.8 Hz), 7.27 – 7.40 (m, 2H), 7.13 – 7.24 (m, 3H), 6.70 (tt, 1H, *J*=7.3, 1.1 Hz), 6.58 – 6.65 (m, 2H), 5.18 (dd, 1H, *J*=7.4, 4.2 Hz), 3.12 – 3.25 (m, 2H), 1.72 – 1.97 (m, 4H) ppm.
^{13}C NMR (126 MHz, $CDCl_3$): δ_C = 148.4, 142.1, 131.9, 129.6, 129.4, 128.6, 127.3, 127.2, 117.6, 113.2, 70.6, 44.1, 35.3, 26.0 ppm.
HRMS (ESI): *m/z* calc. for $C_{16}H_{19}ClNO$ [M+H]$^+$: 276.1150; found: 276.1147.
The enantiomeric excess of the product **(*S*)-3ba** was determined with a Chiralpak IA column (150 x 4.6, particle size 3 μm) and a mobile phase of 25% EtOH/NH_3, 100/20mM in CO_2 120 bar, 3.5 ml/min, λ = 210 nm, 40 °C. Enantiomer 2 for (*S*)-**3ba**, t_r = 2.019 min.
$[\alpha]^{20}_D$: –34.7 (c 1.00, $CHCl_3$).

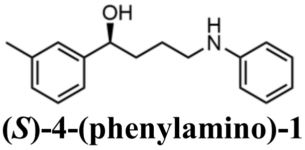

(*S*)-4-(phenylamino)-1-(m-tolyl)butan-1-ol [(*S*)-3ga]

The general procedure was applied using aniline (0.2 mmol) and (*S*)-1-(m-tolyl)butane-1,4-diol (0.4 mmol, 2 equiv). Purification by automatic flash column chromatography (0–100% EtOAc in heptane, 5 g SiO_2), afforded the product **(*S*)-3ga** as brown oil (32.9 mg, 64% yield, 88% *ee*).
^{1}H NMR (500 MHz, $CDCl_3$): δ_H = 7.25 (t, *J* = 7.5 Hz, 1H), 7.21 – 7.08 (m, 5H), 6.72 (tt, *J* = 7.3, 1.1 Hz, 1H), 6.65 – 6.57 (m, 2H), 4.68 (dd, *J* = 7.6, 5.3 Hz, 1H), 3.15 (t, *J* = 6.9 Hz, 2H), 2.39 – 2.36 (m, 3H), 1.96 – 1.71 (m, 3H), 1.70 – 1.59 (m, 1H) ppm.
^{13}C NMR (126 MHz, $CDCl_3$): δ_C = 148.3, 144.7, 138.3, 129.4, 128.5, 128.5, 126.7, 123.0, 117.6, 113.2, 74.5, 44.2, 36.7, 26.0, 21.6. ppm.
HRMS (ESI): *m/z* calc. for $C_{17}H_{22}NO$ $[M+H]^+$: 256.1701; found: 256.1700.
The enantiomeric excess of the product **(*S*)-3ga** was determined with a Shimadzu SFC Prep equipped with Lux C3 (OJ) column (150 x 4.6, particle size 3 µm) and a mobile phase of 25% NH_3/EtOH (20mM), 100/20mM in CO_2 120 bar, 3.5 ml/min, λ = 210 nm, 40 °C. Enantiomer 1 for (*S*)-**3ga**, t_r = 2.484 min.
$[\alpha]^{20}_D$: –13.4 (c 0.57, $CHCl_3$).

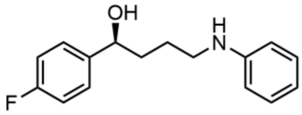

(*S*)-1-(4-fluorophenyl)-4-(phenylamino)butan-1-ol [(*S*)-3ia]
The general procedure was applied using aniline (0.2 mmol) and (*S*)-1-(4-fluorophenyl)butan-1-ol (0.4 mmol, 2 equiv). Purification by automatic flash column chromatography (0–100% EtOAc in heptane, 5 g SiO_2), afforded the product **(*S*)-3ia** as colorless oil (29.7 mg, 57% yield, 72% *ee*).
^{1}H NMR (500 MHz, $CDCl_3$): δ_H = 7.34 – 7.29 (m, 2H), 7.21 – 7.14 (m, 2H), 7.08 – 6.99 (m, 2H), 6.71 (t, *J* = 7.3 Hz, 1H), 6.60 (d, *J* = 7.5 Hz, 2H), 4.71 (dd, *J* = 7.6, 5.3 Hz, 1H), 3.15 (t, *J* = 6.8 Hz, 2H), 1.95 – 1.68 (m, 3H), 1.69 – 1.57 (m, 1H) ppm.
^{13}C NMR (126 MHz, $CDCl_3$): δ_C = 163.3, 161.4, 148.3, 140.5, 140.4, 129.4, 127.6, 127.6, 117.7, 115.5, 115.4, 113.2, 73.8, 44.2, 36.9, 26.0 ppm.
^{19}F NMR (470 MHz, $CDCl_3$): δ_F = –114.97 (ddd, *J* = 14.3, 9.0, 5.4 Hz).
HRMS (ESI): *m/z* calc. for $C_{16}H_{19}FNO$ $[M+H]^+$: 260.1451; found: 260.1451.
The enantiomeric excess of the product **(*S*)-3ia** was determined with a Shimadzu SFC Prep equipped with Chiralpak IB-N column (150 x 4.6, particle size 3 µm) and a mobile phase of 25% NH_3/iPrOH (20mM), 100/20mM in CO_2 120 bar, 3.5 ml/min, λ = 210 nm, 40 °C. Enantiomer 1 for (*S*)-**3ia**, t_r = 2.289 min.
$[\alpha]^{20}_D$: –14.0 (c 0.70, $CHCl_3$).

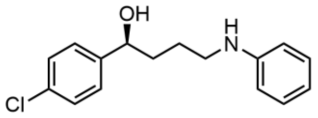

(*S*)-1-(4-chlorophenyl)-4-(phenylamino)butan-1-ol [(*S*)-3ja]
The general procedure was applied using aniline (0.2 mmol) and (*S*)-1-(4-chlorophenyl)butan-1-ol (0.4 mmol, 2 equiv). Purification by automatic flash column chromatography (0–100% EtOAc in heptane, 5 g SiO_2), afforded the product **(*S*)-3ja** as brown oil (42.6 mg, 77% yield, 75% *ee*).
^{1}H NMR (500 MHz, $CDCl_3$): δ_H = 7.36 – 7.32 (m, 2H), 7.31 – 7.27 (m, 2H), 7.23 – 7.18 (m, 2H), 6.76 (tt, *J* = 7.3, 1.1 Hz, 1H), 6.68 – 6.61 (m, 2H), 4.71 (dd, *J* = 7.6, 5.2 Hz, 1H), 3.16 (t, *J* = 6.8 Hz, 2H), 1.94 – 1.72 (m, 3H), 1.71 – 1.61 (m, 1H) ppm.
^{13}C NMR (126 MHz, $CDCl_3$): δ_C = 148.0, 143.2, 133.4, 129.4, 128.8, 127.4, 118.0, 113.4, 73.7, 44.3, 36.8, 25.9 ppm.

HRMS (ESI): *m/z* calc. for $C_{16}H_{19}ClNO$ $[M+H]^+$: 276.1155; found: 276.1157.
The enantiomeric excess of the product **(*S*)-3ja** was determined with a Shimadzu SFC Prep equipped with Chiralpak IF column (150 x 4.6, particle size 3 μm) and a mobile phase of 5-40% NH_3/(MeOH:MeCN 15:85) (20mM), 100/20mM in CO_2 120 bar, 3.5 ml/min, λ = 210 nm, 40 °C. Enantiomer 1 for (*S*)-**3ia**, t_r = 5.608 min.
$[\alpha]^{20}_D$: –13.9 (c 0.97, $CHCl_3$).

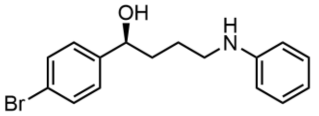

(*S*)-1-(4-bromophenyl)-4-(phenylamino)butan-1-ol [(*S*)-3ka]
The general procedure was applied using aniline (0.2 mmol) and (*S*)-1-(4-bromophenyl)butan-1-ol (0.4 mmol, 2 equiv). Purification by automatic flash column chromatography (0–100% EtOAc in heptane, 5 g SiO_2), afforded the product **(*S*)-3ka** as yellow oil (35.8 mg, 56% yield, 79% *ee*).
^{1}H NMR (400 MHz, $CDCl_3$): δ_H = 7.47 (d, *J* = 8.2 Hz, 2H), 7.24 – 7.14 (m, 4H), 6.71 (td, *J* = 7.3, 1.2 Hz, 1H), 6.60 (d, *J* = 8.0 Hz, 2H), 4.68 (dd, *J* = 7.4, 5.3 Hz, 1H), 3.13 (t, *J* = 6.7 Hz, 2H), 2.94 (brs, 2H), 1.92 – 1.56 (m, 4H) ppm.
^{13}C NMR (101 MHz, $CDCl_3$): δ_C = 148.3, 143.7, 131.7, 129.4, 127.7, 121.4, 117.7, 113.1, 73.7, 44.1, 36.8, 25.9 ppm.
HRMS (ESI): *m/z* calc. for $C_{16}H_{19}BrNO$ $[M+H]^+$: 320.0650; found: 320.0654.
The enantiomeric excess of the product **(*S*)-3ka** was determined with a Shimadzu SFC Prep equipped with Chiralpak IF column (150 x 3, particle size 3 μm) and a mobile phase of 40% MeOH, 100/20mM in CO_2 120 bar, 0.8 ml/min, λ = 210 nm, 40 °C. Enantiomer 1 for (*S*)-**3ka**, t_r = 6.494 min.
$[\alpha]^{20}_D$: –13.9 (c 1.00, $CHCl_3$).

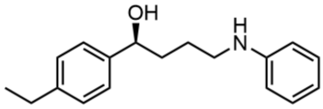

(*S*)-1-(4-ethylphenyl)-4-(phenylamino)butan-1-ol [(*S*)-3na]
The general procedure was applied using aniline (0.2 mmol) and (*S*)-1-(4-ethylphenyl)butane-1,4-diol (0.4 mmol, 2 equiv). Purification by automatic flash column chromatography (0–100% EtOAc in heptane, 5 g SiO_2), afforded the product **(*S*)-3na** as yellow oil (35.3 mg, 66% yield, 89% *ee*).
^{1}H NMR (500 MHz, $CDCl_3$): δ_H = 7.24 – 7.31 (m, 2H), 7.10 – 7.24 (m, 4H), 6.71 (tt, 1H, *J*=7.3, 1.1 Hz), 6.57 – 6.64 (m, 2H), 4.69 (dd, 1H, *J*=7.6, 5.4 Hz), 3.14 (t, 2H, *J*=6.9 Hz), 2.66 (q, 2H, *J*=7.6 Hz), 1.71 – 1.97 (m, 3H), 1.58 – 1.69 (m, 1H), 1.25 (t, 3H, *J*=7.6 Hz) ppm.
^{13}C NMR (126 MHz, $CDCl_3$): δ_C = 148.3, 143.9, 141.9, 129.3, 128.1, 126.0, 117.6, 113.1, 74.3, 44.2, 36.6, 28.7, 26.0, 15.7 ppm.
HRMS (ESI): *m/z* calc. for $C_{18}H_{24}NO$ $[M+H]^+$: 270.1852; found: 270.1854.
The enantiomeric excess of the product **(*S*)-3na** was determined with a Chiralpak IA column (150 x 4.6, particle size 3 μm) and a mobile phase of 30% EtOH/NH_3, 100/20mM in CO_2 120 bar, 3.5 ml/min, λ = 210 nm, 40 °C. Enantiomer 2 for (*S*)-**3na**, t_r = 2.014 min.
$[\alpha]^{20}_D$: –14.6 (c 0.70, $CHCl_3$).

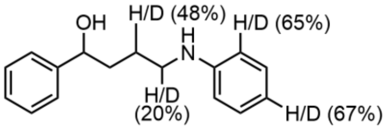

1–phenyl–4–(phenylamino)butan–3–*D*–1–ol [3aa–d(1)]
The general procedure was applied using aniline (0.2 mmol), 1–phenylbutane–1,4–diol **1a** (0.4 mmol, 2 equiv) and the reaction was conducted in HFIP-d_2. The product **3aa–d(1)** (16.8 mg, 35% yield) was purified by automatic flash column chromatography (0–100% EtOAc in heptane, 5 g SiO_2).
^{1}H NMR (500 MHz, $CDCl_3$): δ_H = 7.32 – 7.38 (m, 4H), 7.26 – 7.32 (m, 1H), 7.16 – 7.20 (m, 2H), 6.73 (td, 0.34 H, *J*=7.3, 1.0 Hz, 66% deuterium incorporation), 6.64 (dd, 0.72 H, *J*=8.3, 1.1 Hz, 64% deuterium incorporation), 4.73 (dd, 1H, *J*=7.6, 5.4 Hz), 3.10 – 3.19 (m, 1.59 H, 20% deuterium incorporation), 1.80 – 1.97 (m, 2H), 1.61 – 1.80 (m, 1H) ppm.
^{13}C NMR (126 MHz, $CDCl_3$): δ_C = 147.9 (t, *J*=7.8 Hz), 144.7, 129.3 (t, *J*=13.6 Hz), 128.7, 127.8, 126.0, 117.5 – 118.0 (m), 112.6 – 113.8 (m), 74.4, 43.5 – 44.7 (m), 36.4 – 36.9 (m), 24.9 – 26.0 (m) ppm.
HRMS (ESI): *m/z* calc. for $C_{16}H_{20}NO$ $[M +H]^+$: 242.1539; found: 242.1535 (2.9% relative abundance); *m/z* calc. for $C_{16}H_{19}DNO$ $[(M -H +D)+H]^+$: 243.1602; found: 243.1598 (22.1% relative abundance). *m/z* calc. for $C_{16}H_{18}D_2NO$ $[(M -2H + 2D)+H]^+$: 244.1665; found: 244.1660 (65.8% relative abundance). *m/z* calc. for $C_{16}H_{17}D_3NO$ $[(M -3H + 3D)+H]^+$: 245.1728; found: 245.1721 (100% relative abundance). *m/z* calc. for $C_{16}H_{16}D_4NO$ $[(M -4H + 4D)+H]^+$: 246.1790; found: 246.1783 (85.5% relative abundance). *m/z* calc. for $C_{16}H_{15}D_5NO$ $[(M -5H + 5D)+H]^+$: 247.1853; found: 247.1847 (44.5% relative abundance). *m/z* calc. for $C_{16}H_{14}D_6NO$ $[(M -6H + 6D)+H]^+$: 248.1916; found: 248.1910 (10.4% relative abundance).

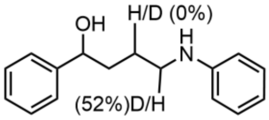

1–phenyl–4–(phenylamino)butan–3–*D*–1–ol [3aa–d(2)]
The general procedure was applied using aniline (0.2 mmol), 1-phenylbutane-4,4-d_2-1,4-diol **1a-d₂** (0.4 mmol, 2 equiv) and the reaction was conducted in HFIP. The product **3aa–d(2)** (27.2 mg, 56% yield) was purified by automatic flash column chromatography (0–100% EtOAc in heptane, 5 g SiO_2).
^{1}H NMR (500 MHz, $CDCl_3$): δ_H = 7.40 – 7.27 (m, 5H), 7.21 – 7.15 (m, 2H), 6.71 (t, *J* = 7.3 Hz, 1H), 6.60 (d, *J* = 8.0 Hz, 2H), 4.71 (dd, *J* = 7.5, 5.4 Hz, 1H), 3.17 – 3.08 (m, 0.95H, 52% deuterium incorporation), 1.95 – 1.71 (m, 3H), 1.63 (ddt, *J* = 13.2, 9.6, 6.3 Hz, 1H) ppm.
^{13}C NMR (126 MHz, $CDCl_3$): δ_C = 148.4, 144.7, 129.3, 128.6, 127.8, 126.0, 117.5, 113.0, 77.4, 77.2, 76.9, 74.4, 44.1, 43.9, 43.7, 43.5, 36.7, 25.9 ppm.
HRMS (ESI): *m/z* calc. for $C_{16}H_{19}N$ $[(M - OH) +H]^+$: 225.1512; found: 225.1491 (100% relative abundance). *m/z* calc. for $C_{16}H_{18}DN$ $[((M -H +D) - OH) +H]^+$: 226.1575; found: 226.1555 (38% relative abundance). *m/z* calc. for $C_{16}H_{17}D_2N$ $[((M -H +D) - OH) +H]^+$: 227.1638; found: 227.1591 (4% relative abundance).

1.4.2 Scope of the anilines

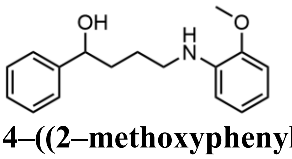

4–((2–methoxyphenyl)amino)–1–phenylbutan–1–ol (3ab)
The general procedure was applied using 2–methoxyaniline (0.2 mmol) and 1–phenylbutane–1,4–diol (0.4 mmol, 2 equiv). Purification by automatic flash column chromatography (0–100% EtOAc in heptane, 5 g SiO_2), afforded the product **3ab** as yellow oil (35.0 mg, 65% yield).
^{1}H NMR (500 MHz, $CDCl_3$): δ_H = 7.26 (d, 4H, J=4.4 Hz), 7.19 (h, 1H, J=4.0 Hz), 6.77 (td, 1H, J=7.6, 1.4 Hz), 6.67 (dd, 1H, J=7.9, 1.4 Hz), 6.57 (td, 1H, J=7.7, 1.5 Hz), 6.50 (dd, 1H, J=7.9, 1.5 Hz), 4.61 (dd, 1H, J=7.5, 5.4 Hz), 3.73 (s, 3H), 3.05 (t, 2H, J=6.9 Hz), 1.63 – 1.88 (m, 3H), 1.57 (tdd, 1H, J=13.1, 8.1, 5.5 Hz) ppm.
^{13}C NMR (126 MHz, $CDCl_3$): δ_C = 147.0, 144.8, 138.3, 128.6, 127.7, 126.0, 121.4, 116.6, 110.1, 109.5, 74.4, 55.5, 43.8, 36.8, 26.0 ppm.
HRMS (ESI): *m/z* calc. for $C_{17}H_{22}NO_2$ $[M+H]^+$: 272.1645; found: 272.1644.

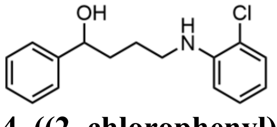

4–((2–chlorophenyl)amino)–1–phenylbutan–1–ol (3ac)
The general procedure was applied using 2–chloroaniline (0.2 mmol) and 1–phenylbutane–1,4–diol (0.4 mmol, 2 equiv). Purification by automatic flash column chromatography (0–100% EtOAc in heptane, 5 g SiO_2), afforded the product **3ac** as colorless oil (21 mg, 38% yield).
^{1}H NMR (500 MHz, $CDCl_3$): δ_H = 7.36 (d, 4H, J=4.4 Hz), 7.29 (ddd, 1H, J=8.7, 4.9, 4.0 Hz), 7.23 (dd, 1H, J=7.8, 1.5 Hz), 7.12 (ddd, 1H, J=8.1, 7.3, 1.5 Hz), 6.57 – 6.65 (m, 2H), 4.74 (dd, 1H, J=7.5, 5.2 Hz), 4.30 (brs, 1H), 3.20 (d, 2H, J=7.4 Hz), 1.75 – 1.99 (m, 4H), 1.63 – 1.75 (m, 1H) ppm.
^{13}C NMR (126 MHz, $CDCl_3$): δ_C = 144.6, 144.2, 129.2, 128.7, 127.9, 127.9, 126.0, 119.2, 117.1, 111.3, 74.5, 43.7, 36.6, 25.8 ppm.
HRMS (ESI): *m/z* calc. for $C_{16}H_{19}ClNO$ $[M+H]^+$: 276.1150; found: 276.1145.

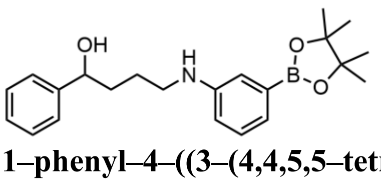

1–phenyl–4–((3–(4,4,5,5–tetramethyl–1,3,2–dioxaborolan–2–yl)phenyl)amino)butan–1–ol (3ad)
The general procedure was applied using 3–(4,4,5,5–tetramethyl–1,3,2–dioxaborolan–2–yl)aniline (0.2 mmol) and 1–phenylbutane–1,4–diol (0.4 mmol, 2 equiv), with 3 mol% of **Ir-1** catalyst instead of 4 mol%. Purification by automatic flash column chromatography (0–100% EtOAc in heptane, 5 g SiO_2), afforded the product **3ad** as orange oil (42.8 mg, 58% yield).
^{1}H NMR (500 MHz, $CDCl_3$): δ_H = 7.35 – 7.41 (m, 4H), 7.29 – 7.34 (m, 1H), 7.17 – 7.26 (m, 2H), 7.09 (d, 1H, J=2.6 Hz), 6.73 (ddd, 1H, J=7.5, 2.6, 1.8 Hz), 4.72 (dd, 1H, J=7.6, 5.4 Hz), 3.18 (t, 2H, J=6.9 Hz), 1.78 – 1.94 (m, 2H), 1.68 – 1.78 (m, 1H), 1.63 (dtt, J = 13.2, 9.7, 6.2 Hz, 1H), 1.37 (s, 12H) ppm.
^{13}C NMR (126 MHz, $CDCl_3$): δ_C = 147.8, 144.7, 128.7, 128.5, 127.6, 125.9, 123.9, 119.2, 115.8, 83.7, 74.2, 44.0, 36.7, 25.9, 24.9 ppm. The carbon attached to boron was not observed, likely due to quadrupolar relaxation.
^{11}B NMR (160 MHz, $CDCl_3$): δ_B = 30.1 ppm.

HRMS (ESI): *m/z* calc. for $C_{22}H_{31}BNO_3$ $[M+H]^+$: 368.2392; found: 368.2410.

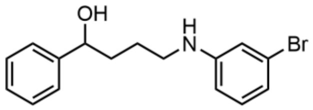

4–((3–bromophenyl)amino)–1–phenylbutan–1–ol (3ae)
The general procedure was applied using 3–bromoaniline (0.2 mmol) and 1–phenylbutane–1,4–diol (0.4 mmol, 2 equiv), with 3 mol% of **Ir-1** catalyst instead of 4 mol%. Purification by automatic flash column chromatography (0–100% EtOAc in heptane, 5 g SiO_2), afforded the product **3ae** as colorless oil (42.3 mg, 66% yield).
^{1}H NMR (500 MHz, $CDCl_3$): δ_H = 7.32 – 7.39 (m, 4H), 7.28 – 7.32 (m, 1H), 6.99 (t, 1H, *J*=8.0 Hz), 6.79 (ddd, 1H, *J*=7.8, 1.8, 0.9 Hz), 6.70 (t, 1H, *J*=2.1 Hz), 6.47 (ddd, 1H, *J*=8.3, 2.3, 0.9 Hz), 4.71 (dd, 1H, *J*=7.6, 5.4 Hz), 3.10 (t, 2H, *J*=6.9 Hz), 1.56 – 1.95 (m, 4H) ppm.
^{13}C NMR (126 MHz, $CDCl_3$): δ_C = 149.6, 144.4, 130.4, 128.6, 127.8, 125.8, 123.3, 119.9, 115.1, 111.5, 74.3, 43.6, 36.4, 25.6 ppm.
HRMS (ESI): *m/z* calc. for $C_{16}H_{19}BrNO$ $[M+H]^+$: 320.0645; found: 320.0661.

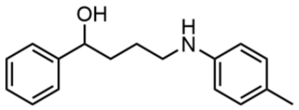

1–phenyl–4–(p–tolylamino)butan–1–ol (3af)
The general procedure was applied using p–toluidine (0.2 mmol) and 1–phenylbutane–1,4–diol (0.4 mmol, 2 equiv), with 3 mol% of **Ir-1** catalyst instead of 4 mol%. Purification by automatic flash column chromatography (0–100% EtOAc in heptane, 5 g SiO_2), afforded the product **3af** as amorphous orange solid (36.7 mg, 72% yield). M.p: 77-80 °C.
^{1}H NMR (500 MHz, $CDCl_3$): δ_H = 7.33 – 7.4 (m, 4H), 7.27 – 7.33 (m, 1H), 6.96 – 7.03 (m, 2H), 6.51 – 6.59 (m, 2H), 4.70 (dd, 1H, *J*=7.6, 5.4 Hz), 3.11 (t, 2H, *J*=6.9 Hz), 2.26 (s, 3H), 1.80 – 1.95 (m, 2H), 1.69 – 1.80 (m, 1H), 1.57 – 1.69 (m, 1H) ppm.
^{13}C NMR (126 MHz, $CDCl_3$): δ_C =146.0, 144.6, 129.7, 128.5, 127.6, 126.7, 125.9, 113.2, 74.3, 44.4, 36.7, 26.0, 20.4 ppm.
HRMS (ESI): *m/z* calc. for $C_{17}H_{22}NO$ $[M+H]^+$: 256.1696; found: 256.1691.

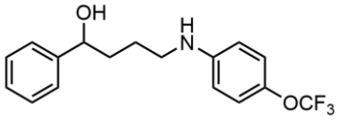

1–phenyl–4–((4–(trifluoromethoxy)phenyl)amino)butan–1–ol (3ag)
The general procedure was applied using 4–(trifluoromethoxy)aniline (0.2 mmol) and 1–phenylbutane–1,4–diol (0.4 mmol, 2 equiv). Purification by automatic flash column chromatography (0–100% EtOAc in heptane, 5 g SiO_2), afforded the product **3ag** as pale brown amorphous solid (39.1 mg, 60% yield). M.p: 50-53 °C.
^{1}H NMR (500 MHz, $CDCl_3$): δ_H = 7.35 – 7.42 (m, 4H), 7.32 (ddd, 1H, *J*=8.5, 5.5, 2.3 Hz), 7.04 (d, 2H, *J*=8.5 Hz), 6.51 – 6.58 (m, 2H), 4.74 (dd, 1H, *J*=7.6, 5.3 Hz), 3.13 (t, 2H, *J*=6.9 Hz), 1.95 – 1.70 (m, 3H), 1.64 (ddq, *J* = 13.1, 10.0, 6.5 Hz, 1H) ppm.
^{13}C NMR (126 MHz, $CDCl_3$): δ_C = 147.1, 144.5, 140.35 (q, *J*=2.0 Hz), 128.6, 127.8, 122.4, 120.7 (q, *J*=255.1 Hz), 112.9, 74.3, 44.1, 38.5, 25.7 ppm.
^{19}F NMR (470 MHz, $CDCl_3$): δ_F = –58.5 ppm
HRMS (ESI): *m/z* calc. for $C_{17}H_{19}F_3NO_2$ $[M+H]^+$: 326.1362; found: 326.1362.

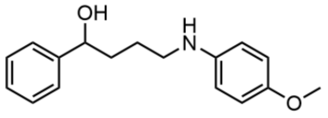

4–((4–methoxyphenyl)amino)–1–phenylbutan–1–ol (3ah)
The general procedure was applied using 4–methoxyaniline (0.2 mmol) and 1–phenylbutane–1,4–diol (0.4 mmol, 2 equiv). Purification by automatic flash column chromatography (0–100% EtOAc in heptane, 5 g SiO_2), afforded the product **3ah** as brown amorphous solid (43.5 mg, 80% yield). M.p: 91-93 °C.
^{1}H NMR (500 MHz, $CDCl_3$): δ_H = 7.26 – 7.38 (m, 5H), 6.78 (d, 2H, *J*=8.0 Hz), 6.58 (d, 2H, *J*=9.1 Hz), 4.69 (t, 1H, *J*=6.5 Hz), 3.73 – 3.76 (m, 3H), 3.08 (t, 2H, *J*=6.8 Hz), 1.78 – 1.95 (m, 2H), 1.56 – 1.78 (m, 2H) ppm.
^{13}C NMR (126 MHz, $CDCl_3$): δ_C = 152.3, 144.7, 142.5, 128.5, 127.6, 125.9, 114.9, 114.5, 74.2, 55.8, 45.1, 36.8, 26.1 ppm.
HRMS (ESI): *m/z* calc. for $C_{17}H_{22}NO_2$ $[M+H]^+$: 272.1645; found: 272.1644.

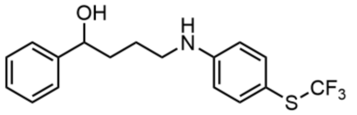

1–phenyl–4–((4–((trifluoromethyl)thio)phenyl)amino)butan–1–ol (3ai)
The general procedure was applied using 4–((trifluoromethyl)thio)aniline (0.2 mmol) and 1–phenylbutane–1,4–diol (0.4 mmol, 2 equiv). Purification by automatic flash column chromatography (0–100% EtOAc in heptane, 5 g SiO_2), afforded the product **3ai** as colorless oil (46.4 mg, 68% yield).
^{1}H NMR (500 MHz, $CDCl_3$): δ_H = 7.39 – 7.44 (m, 2H), 7.33 – 7.39 (m, 4H), 7.28 – 7.33 (m, 1H), 6.50 – 6.57 (m, 2H), 4.72 (dd, 1H, *J*=7.6, 5.2 Hz), 4.03 (brs, 1H), 3.14 (t, 2H, *J*=6.8 Hz), 2.07 (brs, 1H), 1.71 – 1.95 (m, 3H), 1.65 (dddd, 1H, *J*=13.3, 9.7, 5.8, 1.6 Hz) ppm.
^{13}C NMR (126 MHz, $CDCl_3$): δ_C = 150.4, 144.4, 138.2, 129.8 (q, *J*=308.5 Hz), 128.6, 127.8, 125.8, 112.9, 109.3 (d, *J*=2.2 Hz), 74.3, 43.4, 36.3, 25.5 ppm.
^{19}F NMR (470 MHz, $CDCl_3$): δ_F = –44.7 ppm
HRMS (ESI): *m/z* calc. for $C_{17}H_{19}F_3NOS$ $[M+H]^+$: 342.1134; found: 342.1146.

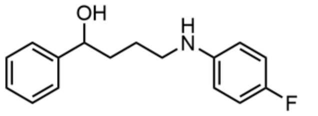

4–((4–fluorophenyl)amino)–1–phenylbutan–1–ol (3aj)
The general procedure was applied using 4–fluoroaniline (0.2 mmol) and 1–phenylbutane–1,4–diol (0.4 mmol, 2 equiv). Purification by automatic flash column chromatography (0–100% EtOAc in heptane, 5 g SiO_2), afforded the product **3aj** as brown oil (34.1 mg, 66% yield).
^{1}H NMR (500 MHz, $CDCl_3$): δ_H = 7.36 (d, 4H, *J*=4.8 Hz), 7.26 – 7.33 (m, 1H), 6.83 – 6.91 (m, 2H), 6.48 – 6.56 (m, 2H), 4.73 (dd, 1H, *J*=7.6, 5.4 Hz), 3.10 (t, 2H, *J*=6.9 Hz), 1.96 – 1.80 (m, 2H), 1.80 – 1.70 (m, 1H), 1.69 – 1.59 (m, 1H) ppm.
^{13}C NMR (126 MHz, $CDCl_3$): δ_C = 157.0, 155.1, 144.67 (d, *J*=2.7 Hz), 128.7, 127.9, 126.0, 115.78 (d, *J*=22.3 Hz), 113.95 (d, *J*=7.4 Hz), 74.5, 44.9, 36.8, 26.0 ppm.
^{19}F NMR (470 MHz, $CDCl_3$): δ_F = –128.2 ppm
HRMS (ESI): *m/z* calc. for $C_{16}H_{19}FNO$ $[M+H]^+$: 260.1445; found: 260.1445.

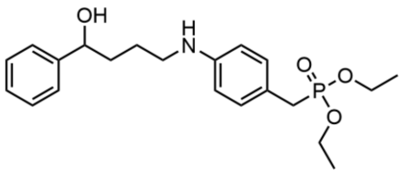

diethyl (4–((4–hydroxy–4–phenylbutyl)amino)benzyl)phosphonate (3ak)
The general procedure was applied using diethyl (4–aminobenzyl)phosphonate (0.2 mmol) and 1–phenylbutane–1,4–diol (0.4 mmol, 2 equiv). Purification by automatic flash column chromatography (0–100% EtOAc in heptane, 5 g SiO_2), afforded the product **3ak** as orange oil (60.2 mg, 77% yield).
^{1}H NMR (500 MHz, $CDCl_3$): δ_H = 7.34 (d, J = 4.4 Hz, 4H), 7.29 – 7.23 (m, 1H), 7.06 (dd, J = 8.6, 2.6 Hz, 2H), 6.52 (d, J = 8.1 Hz, 2H), 4.70 (dd, J = 7.6, 5.3 Hz, 1H), 4.04 – 3.90 (m, 4H), 3.10 (t, J = 6.9 Hz, 2H), 3.01 (d, J = 20.8 Hz, 2H), 1.93 – 1.68 (m, 3H), 1.62 (ddq, J = 13.1, 9.8, 6.5 Hz, 1H), 1.22 (t, J = 7.1 Hz, 6H) ppm.
^{13}C NMR (126 MHz, $CDCl_3$): δ_C = 147.3 (d, J=3.1 Hz), 144.8, 130.6 (d, J=6.4 Hz), 128.6, 127.7, 126.0, 119.7 (d, J=9.5 Hz), 113.2 (d, J=2.9 Hz), 74.3, 62.2 (d, J=6.9 Hz), 44.2, 36.8, 32.7 (d, J=138.9 Hz), 26.0, 16.5 (d, J=5.9 Hz) ppm.
^{31}P NMR (202 MHz, $CDCl_3$): δ_P = 27.34 ppm.
HRMS (ESI): *m/z* calc. for $C_{21}H_{31}NO_4P$ $[M+H]^+$: 392.1985; found: 392.1972.

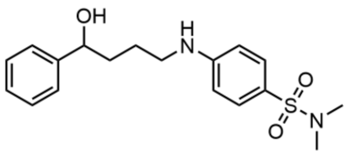

4–amino–N,N–dimethylbenzenesulfonamide (3al)
The general procedure was applied using aniline (0.2 mmol) and 1–phenylbutane–1,4–diol (0.4 mmol, 2 equiv). Purification by automatic flash column chromatography (0–100% EtOAc in heptane, 5 g SiO_2), afforded the product **3al** as colorless oil (32.3 mg, 46% yield).
^{1}H NMR (500 MHz, $CDCl_3$): δ_H = 7.57 – 7.51 (m, 2H), 7.40 – 7.33 (m, 4H), 7.30 (ddd, J = 8.6, 3.6, 2.5 Hz, 1H), 6.60 – 6.54 (m, 2H), 4.74 (dd, J = 7.5, 5.0 Hz, 1H), 4.25 (s, 1H), 3.19 (t, J = 6.7 Hz, 2H), 2.65 (s, 6H), 1.97 – 1.75 (m, 4H), 1.74 – 1.63 (m, 1H) ppm.
^{13}C NMR (126 MHz, $CDCl_3$): δ_C = 151.8, 144.5, 130.0, 128.7, 127.9, 125.9, 121.7, 111.6, 74.3, 43.3, 38.2, 36.4, 25.6 ppm.
HRMS (ESI): *m/z* calc. for $C_{18}H_{25}N_2O_3S$ $[M+H]^+$: 349.1580; found: 349.1587.

1.4.3 Scope of *Late-stage functionalization*

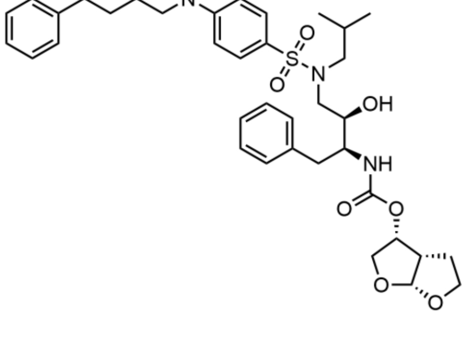

(3R,3aS,6aR)-hexahydrofuro[2,3-b]furan-3-yl-((2S,3R)-3-hydroxy-4-((4-((4-hydroxy-4-phenylbutyl)amino)-N-isobutylphenyl)sulfonamido)-1-phenylbutan-2-yl)carbamate (7aa)

The general procedure was applied using Darunavir (0.1 mmol) and 1–phenylbutane–1,4–diol **1a** (0.2 mmol, 2 equiv), the reaction was carried out at 65 °C instead of 75 °C.
Purification by preparative HPLC (20-100% MeCN in H_2O, basic buffer), afforded the product **7aa** as white amorphous solid (32.5 mg, 47% yield). M.p.: 71-74 °C.

^{1}H NMR (500 MHz, DMSO-d_6): δ_H = 7.42 (d, *J* = 8.8 Hz, 2H), 7.35 – 7.28 (m, 4H), 7.26 (d, *J* = 9.4 Hz, 1H), 7.22 (d, *J* = 4.4 Hz, 5H), 7.14 (dt, *J* = 8.8, 4.2 Hz, 1H), 6.63 – 6.56 (m, 2H), 6.52 (t, *J* = 5.4 Hz, 1H), 5.51 (d, *J* = 5.2 Hz, 1H), 5.17 (d, *J* = 4.4 Hz, 1H), 4.98 (d, *J* = 6.4 Hz, 1H), 4.85 (dt, *J* = 8.1, 5.7 Hz, 1H), 4.55 (q, *J* = 5.6 Hz, 1H), 3.86 (dd, *J* = 9.5, 6.0 Hz, 1H), 3.73 (td, *J* = 8.2, 1.9 Hz, 1H), 3.61 (ddt, *J* = 18.4, 14.4, 7.2 Hz, 4H), 3.07 – 3.01 (m, 3H), 2.94 (dd, *J* = 13.5, 8.6 Hz, 1H), 2.78 (q, *J* = 7.9 Hz, 1H), 2.69 – 2.59 (m, 3H), 2.37 (p, *J* = 1.9 Hz, 1H), 1.95 (dt, *J* = 13.9, 6.8 Hz, 1H), 1.64 (ddq, *J* = 29.4, 14.5, 6.4 Hz, 3H), 1.51 (dt, *J* = 14.8, 7.0 Hz, 1H), 1.45 – 1.33 (m, 1H), 1.29 – 1.22 (m, 1H), 0.83 (dd, *J* = 31.1, 6.6 Hz, 6H).

^{13}C NMR (126 MHz, DMSO-d_6): δ_C = 155.2, 152.2, 146.3, 139.5, 129.2, 128.9, 127.9, 127.9, 126.6, 125.8, 125.7, 123.0, 110.7, 108.8, 72.8, 72.3, 72.0, 70.4, 68.9, 57.4, 55.9, 52.8, 45.1, 42.3, 36.7, 35.2, 26.4, 25.6, 24.9, 20.1 ppm.

HRMS (ESI): *m/z* calc. for $C_{37}H_{50}N_3O_8S$ $[M+H]^+$: 696.3318; found: 696.3317.

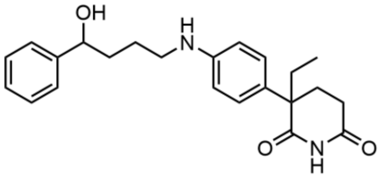

3-ethyl-3-(4-((4-hydroxy-4-phenylbutyl)amino)phenyl)piperidine-2,6-dione (7ab)

The general procedure was applied using Aminoglutethimide (0.1 mmol) and 1–phenylbutane–1,4–diol **1a** (0.2 mmol, 2 equiv), the reaction was carried out at 65 °C instead of 75 °C. Purification by preparative HPLC (20-100% MeCN in H_2O, acid buffer), afforded the product **7ab** as yellow oil (22.0 mg, 58% yield).

^{1}H NMR (500 MHz, DMSO-d_6): δ_H = 10.70 (s, 1H), 7.27–7.35 (m, 4H), 7.18–7.22 (m, 1H), 6.95 (d, *J* = 8.8 Hz, 2H), 6.51 (d, *J* = 8.8 Hz, 2H), 5.56 (t, *J* = 5.6 Hz, 1H), 5.14 (d, *J* = 4.4 Hz, 1H), 4.49–4.57 (m, 1H), 2.95 (h, *J* = 5.8 Hz, 2H), 2.37–2.45 (m, 1H), 2.24 (ddd, *J* = 13.3, 4.5, 2.5 Hz, 1H), 2.01–2.19 (m, 2H), 1.53–1.84 (m, 5H), 1.48 (dtd, *J* = 13.1, 6.8, 1.8 Hz, 1H), 0.73 (t, *J* = 7.4 Hz, 3H) ppm.

^{13}C NMR (126 MHz, DMSO-d_6): δ_C = 176.2, 172.9, 147.9, 146.3, 127.9, 126.7, 126.5, 125.8, 125.6, 111.9, 72.1, 49.2, 42.8, 36.9, 32.3, 29.2, 25.9, 25.2, 8.9. ppm.

HRMS (ESI): *m/z* calc. For $C_{23}H_{27}N_2O_3$ $[M-H^+]^-$: 379.2022; found: 379.2034

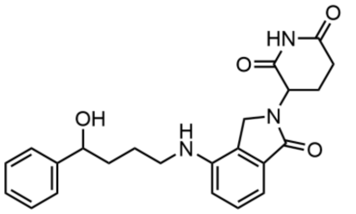

3-(4-((4-hydroxy-4-phenylbutyl)amino)-1-oxoisoindolin-2-yl)piperidine-2,6-dione (7ac)

The general procedure was applied using Lenalidomide **6c** (0.2 mmol) and 1–phenylbutane–1,4–diol **1a** (0.4 mmol, 2 equiv), the reaction was carried out at 65 °C instead of 75 °C.

Purification by preparative HPLC (20-100% MeCN in H_2O, acid buffer), afforded the product **7ac** as white amorphous solid (33.6 mg, 41% yield). M.p.: 167-169 °C.
^{1}H NMR (500 MHz, DMSO-d$_6$): δ_H = 10.99 (s, 1H), 7.16 – 7.36 (m, 6H), 6.91 (dd, 1H, *J*=7.5, 0.7 Hz), 6.71 (d, 1H, *J*=8.0 Hz), 5.58 (t, 1H, *J*=5.5 Hz), 5.18 (d, 1H, *J*=4.3 Hz), 5.10 (dd, 1H, *J*=13.3, 5.1 Hz), 4.52 – 4.59 (m, 1H), 4.20 (d, 1H, *J*=17.2 Hz), 4.10 (d, 1H, *J*=17.1 Hz), 3.11 (q, 2H, *J*=6.5 Hz), 2.92 (ddd, 1H, *J*=17.3, 13.6, 5.4 Hz), 2.57 – 2.65 (m, 1H), 2.28 (qd, 1H, *J*=13.3, 4.5 Hz), 2.02 (dtd, 1H, *J*=12.7, 5.4, 2.3 Hz), 1.60 – 1.75 (m, 3H), 1.48 – 1.59 (m, 1H) ppm.
^{13}C NMR (126 MHz, DMSO-d$_6$): δ_C = 172.9, 171.2, 168.9, 146.3, 143.7, 132.0, 129.2, 127.9, 126.6, 126.4, 125.8, 111.7, 109.8, 72.1, 51.5, 45.7, 42.8, 36.8, 31.2, 25.0, 22.8 ppm.
HRMS (ESI): *m/z* calc. for $C_{23}H_{24}N_3O_3$ [(M – H_2O) + H]$^+$: 390.1812; found: 390.1825.

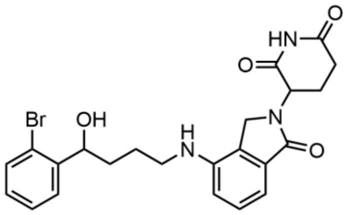

3-(4-((4-(2-bromophenyl)-4-hydroxybutyl)amino)-1-oxoisoindolin-2-yl)piperidine-2,6-dione (7cc)

The general procedure was applied using Lenalidomide **6c** (0.2 mmol) and 1–(2–bromophenyl)butane–1,4–diol **1c** (0.4 mmol, 2 equiv), the reaction was carried out at 65 °C instead of 75 °C. Purification by preparative HPLC (20-100% MeCN in H_2O, acid buffer), afforded the product **7cc** as yellow amorphous solid (75.1 mg, 77% yield). M.p.: 110-113 °C.
^{1}H NMR (500 MHz, DMSO-d$_6$): δ_H = 11.00 (s, 1H), 7.60 – 7.51 (m, 2H), 7.39 (td, *J* = 7.6, 1.3 Hz, 1H), 7.27 (t, *J* = 7.7 Hz, 1H), 7.17 (td, *J* = 7.6, 1.8 Hz, 1H), 6.94 – 6.89 (m, 1H), 6.73 (dt, *J* = 8.1, 1.1 Hz, 1H), 5.59 (t, *J* = 5.6 Hz, 1H), 5.44 (d, *J* = 4.5 Hz, 1H), 5.11 (dd, *J* = 13.3, 5.1 Hz, 1H), 4.86 (p, *J* = 3.4 Hz, 1H), 4.22 (d, *J* = 17.1 Hz, 1H), 4.12 (d, *J* = 17.1 Hz, 1H), 3.14 (dh, *J* = 12.9, 6.6 Hz, 2H), 2.92 (ddd, *J* = 17.3, 13.6, 5.4 Hz, 1H), 2.61 (ddd, *J* = 17.3, 4.5, 2.3 Hz, 1H), 2.29 (qd, *J* = 13.2, 4.5 Hz, 1H), 2.02 (dtt, *J* = 14.4, 6.2, 3.1 Hz, 1H), 1.78 – 1.56 (m, 4H) ppm.
^{13}C NMR (126 MHz, DMSO-d$_6$): δ_C = 172.9, 171.2, 168.9, 145.0, 143.7, 132.1, 132.0, 129.2, 128.6, 127.8, 127.7, 126.5, 121.1, 111.7, 109.9, 70.8 (d, *J*=2.1 Hz), 51.4, 45.7, 42.7, 35.3 (d, *J*=2.2 Hz), 31.2, 25.0 (d, *J*=2.8 Hz), 22.8 ppm
HRMS (ESI): *m/z* calc. for $C_{23}H_{25}BrN_3O_4$ [M+H]$^+$: 486.1023; found: 486.1033.

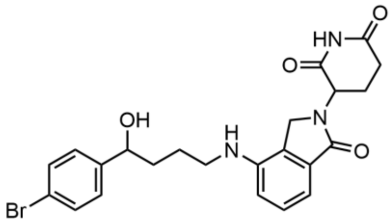

3-(4-((4-(4-bromophenyl)-4-hydroxybutyl)amino)-1-oxoisoindolin-2-yl)piperidine-2,6-dione (7kc)

The general procedure was applied using Lenalidomide **6c** (0.2 mmol) and 1–(4–bromophenyl)butane–1,4–diol **1k** (0.4 mmol, 2 equiv), the reaction was carried out at 65 °C instead of 75 °C. Purification by preparative HPLC (20-100% MeCN in H_2O, acid buffer), afforded the product **7kc** as yellow amorphous solid (53 mg, 54% yield). M.p.: 102-104 °C.

^{1}H NMR (500 MHz, DMSO-d$_6$): δ_H = 11.00 (s, 1H), 7.53 – 7.46 (m, 2H), 7.33 – 7.23 (m, 3H), 6.91 (dd, *J* = 7.4, 0.8 Hz, 1H), 6.71 (d, *J* = 8.0 Hz, 1H), 5.57 (t, *J* = 5.6 Hz, 1H), 5.29 (d, *J* = 4.4 Hz, 1H), 5.10 (dd, *J* = 13.3, 5.1 Hz, 1H), 4.56 (q, *J* = 6.0 Hz, 1H), 4.20 (d, *J* = 17.1 Hz, 1H), 4.10 (d, *J* = 17.1 Hz, 1H), 3.11 (q, *J* = 6.6 Hz, 2H), 2.92 (ddd, *J* = 17.3, 13.6, 5.4 Hz, 1H), 2.61 (ddd, *J* = 17.3, 4.4, 2.4 Hz, 1H), 2.29 (qd, *J* = 13.2, 4.5 Hz, 1H), 2.02 (dtt, *J* = 13.3, 5.0, 2.6 Hz, 1H), 1.71 – 1.49 (m, 4H) ppm
^{13}C NMR (126 MHz, DMSO-d$_6$): δ_C = 172.9, 171.3, 168.9, 145.7, 143.7, 132.0, 130.8, 129.2, 128.1, 126.4, 119.5, 111.7, 109.9, 71.4 (d, *J*=1.8 Hz), 51.5, 45.7, 42.7, 36.6, 31.2, 24.8, 22.8 ppm.
HRMS (ESI): *m/z* calc. for $C_{23}H_{25}BrN_3O_4$ $[M+H]^+$: 486.1023; found: 486.1030.

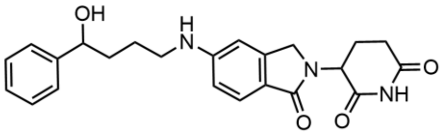

3-(5-((4-hydroxy-4-phenylbutyl)amino)-1-oxoisoindolin-2-yl)piperidine-2,6-dione (7ad)

The general procedure was applied using C5 Lenalidomide **6d** (0.2 mmol) and 1–phenylbutane–1,4–diol **1a** (0.4 mmol, 2 equiv), the reaction was carried out at 65 °C instead of 75 °C. Purification by preparative HPLC (20-100% MeCN in H_2O, acid buffer), afforded the product **7ad** as beige amorphous solid (45.8 mg, 56% yield). M.p.: 168-172 °C.
^{1}H NMR (500 MHz, DMSO-d$_6$): δ_H = 10.92 (s, 1H), 7.36 (d, *J* = 8.4 Hz, 1H), 7.35 – 7.28 (m, 4H), 7.18 – 7.25 (m, 1H), 6.63 (dd, 1H, *J*=8.4, 2.0 Hz), 6.58 (d, 1H, *J*=1.9 Hz), 6.34 (t, 1H, *J*=5.5 Hz), 5.18 (d, 1H, *J*=4.4 Hz), 5.01 (dd, 1H, *J*=13.3, 5.1 Hz), 4.56 (dt, 1H, *J*=7.3, 4.8 Hz), 4.25 (d, 1H, *J*=16.6 Hz), 4.12 (d, 1H, *J*=16.6 Hz), 3.07 (q, 2H, *J*=5.9 Hz), 2.89 (ddd, 1H, *J*=17.2, 13.6, 5.4 Hz), 2.52 – 2.62 (m, 1H), 2.33 (qd, 1H, *J*=13.3, 4.5 Hz), 1.94 (ddq, 1H, *J*=10.4, 5.4, 2.7 Hz), 1.58 – 1.76 (m, 3H), 1.46 – 1.58 (m, 1H) ppm.
^{13}C NMR (126 MHz, DMSO-d$_6$): δ_C = 173.0, 171.4, 168.7, 152.3, 146.3, 144.7, 127.9, 126.6, 125.8, 123.9, 118.6, 112.4, 104.1, 72.0, 51.3, 46.7, 42.6, 36.8, 31.2, 25.0, 22.7 ppm.
HRMS (ESI): *m/z* calc. for $C_{23}H_{26}N_3O_4$ $[M+H]^+$: 408.1918; found: 408.1923.

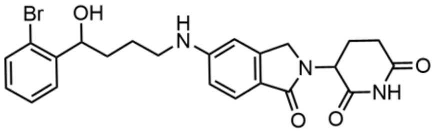

3-(5-((4-(2-bromophenyl)-4-hydroxybutyl)amino)-1-oxoisoindolin-2-yl)piperidine-2,6-dione (7cd)

The general procedure was applied using C5 Lenalidomide **6d** (0.2 mmol) and 1–(2–bromophenyl)butane–1,4–diol **1c** (0.4 mmol, 2 equiv), the reaction was carried out at 65 °C instead of 75 °C. Purification by preparative HPLC (20-100% MeCN in H_2O, acid buffer), afforded the product **7cd** as yellow amorphous solid (67.7 mg, 70% yield). M.p.: 105-108 °C.
^{1}H NMR (500 MHz, DMSO-d$_6$): δ_H = 10.92 (s, 1H), 7.56 (ddd, *J* = 19.4, 7.9, 1.5 Hz, 2H), 7.42 – 7.34 (m, 2H), 7.18 (td, *J* = 7.6, 1.8 Hz, 1H), 6.63 (dd, *J* = 8.4, 2.0 Hz, 1H), 6.60 (d, *J* = 1.9 Hz, 1H), 6.35 (t, *J* = 5.5 Hz, 1H), 5.44 (d, *J* = 4.5 Hz, 1H), 5.01 (dd, *J* = 13.3, 5.1 Hz, 1H), 4.86 (dt, *J* = 7.7, 3.8 Hz, 1H), 4.26 (d, *J* = 16.6 Hz, 1H), 4.13 (d, *J* = 16.6 Hz, 1H), 3.09 (qd, *J* = 6.0, 3.3 Hz, 2H), 2.89 (ddd, *J* = 17.2, 13.6, 5.4 Hz, 1H), 2.58 (ddd, *J* = 17.3, 4.5, 2.4 Hz, 1H), 2.33 (qd, *J* = 13.3, 4.5 Hz, 1H), 1.94 (dtd, *J* = 12.6, 5.2, 2.2 Hz, 1H), 1.72 (ttd, *J* = 9.5, 6.9, 3.2 Hz, 2H), 1.67 – 1.55 (m, 2H) ppm.

^{13}C NMR (126 MHz, DMSO-d$_6$): δ_C = 173.0, 171.4, 168.7, 152.3, 145.0, 144.5, 132.1, 128.7, 127.8, 127.7, 123.9, 121.1, 118.6, 112.4, 104.1, 70.8, 51.3, 46.8, 42.5, 40.1, 40.0, 39.9, 39.9, 39.8, 39.7, 39.6, 39.5, 39.4, 39.4, 39.2, 39.0, 35.3, 31.3, 24.9, 22.7 ppm.
HRMS (ESI): *m/z* calc. for $C_{23}H_{25}BrN_3O_4$ [M+H]$^+$: 486.1023; found: 486.1032.

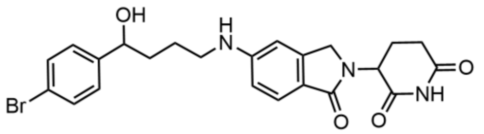

3-(5-((4-(4-bromophenyl)-4-hydroxybutyl)amino)-1-oxoisoindolin-2-yl)piperidine-2,6-dione (7kd)
The general procedure was applied using C5 Lenalidomide **6d** (0.2 mmol) and 1–(4–bromophenyl)butane–1,4–diol **1k** (0.4 mmol, 2 equiv), the reaction was carried out at 65 °C instead of 75 °C. Purification by preparative HPLC (20-100% MeCN in H_2O, acid buffer), afforded the product **7kd** as white amorphous solid (63.7 mg, 65% yield). M.p.: 147-149 °C.
^{1}H NMR (500 MHz, DMSO-d$_6$): δ_H = 10.92 (s, 1H), 7.47 – 7.53 (m, 2H), 7.37 (d, 1H, *J*=8.4 Hz), 7.27 – 7.34 (m, 2H), 6.66 – 6.55 (m, 2H), 6.34 (t, 1H, *J*=5.5 Hz), 5.29 (brs, 1H), 5.01 (dd, 1H, *J*=13.3, 5.1 Hz), 4.56 (t, 1H, *J*=6.2 Hz), 4.25 (d, 1H, *J*=16.6 Hz), 4.13 (d, 1H, *J*=16.6 Hz), 3.06 (q, 2H, *J*=6.5 Hz), 2.89 (ddd, 1H, *J*=17.2, 13.6, 5.4 Hz), 2.52 – 2.62 (m, 1H), 2.33 (qd, 1H, *J*=13.2, 4.5 Hz), 1.94 (dtd, 1H, *J*=12.8, 5.2, 2.3 Hz), 1.58 – 1.70 (m, 3H), 1.48 – 1.56 (m, 1H) ppm.
^{13}C NMR (126 MHz, DMSO-d$_6$): δ_C = 173.0, 171.4, 168.6, 152.3, 145.7, 144.5, 130.8, 128.1, 123.9, 119.5, 118.6, 112.4, 104.7, 71.3, 51.3, 46.8, 42.5, 36.6, 31.3, 24.8, 22.7 ppm
HRMS (ESI): *m/z* calc. for $C_{23}H_{25}BrN_3O_4$ [M+H]$^+$: 486.1023; found: 486.1036.

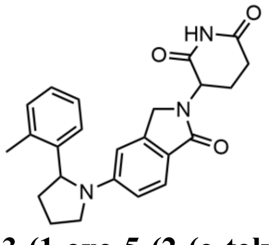

3-(1-oxo-5-(2-(o-tolyl)pyrrolidin-1-yl)isoindolin-2-yl)piperidine-2,6-dione (7dd)
The general procedure was applied using C5 Lenalidomide **6d** (0.2 mmol) and 1–(o–tolyl)butane–1,4–diol **1d** (0.4 mmol, 2 equiv), the reaction was carried out at 65 °C instead of 75 °C. Purification by preparative HPLC (20-100% MeCN in H_2O, acid buffer), afforded the product **7dd** as yellow amorphous solid (64.7 mg, 80% yield). M.p.: 167-169 °C.
^{1}H NMR (500 MHz, DMSO-d$_6$): δ_H = 10.91 (d, *J* = 4.1 Hz, 1H), 7.39 (dd, *J* = 8.5, 4.0 Hz, 1H), 7.21 (d, *J* = 7.4 Hz, 1H), 7.12 (td, *J* = 7.4, 1.4 Hz, 1H), 7.04 (td, *J* = 7.5, 1.4 Hz, 1H), 6.85 (ddd, *J* = 7.7, 3.9, 1.4 Hz, 1H), 6.49 (d, *J* = 5.8 Hz, 1H), 6.41 (t, *J* = 9.4 Hz, 1H), 5.00 (dt, *J* = 13.3, 5.2 Hz, 2H), 4.23 (t, *J* = 16.0 Hz, 1H), 4.10 (dd, *J* = 16.7, 14.7 Hz, 1H), 3.82 – 3.73 (m, 1H), 3.47 (tdd, *J* = 9.9, 6.8, 3.9 Hz, 1H), 2.88 (ddd, *J* = 18.1, 13.6, 5.5 Hz, 1H), 2.56 (dq, *J* = 17.1, 3.7 Hz, 1H), 2.42 (s, 4H), 2.30 (qdd, *J* = 13.2, 8.4, 4.5 Hz, 1H), 2.02 (dtt, *J* = 11.8, 6.6, 3.2 Hz, 1H), 1.92 (qp, *J* = 5.5, 3.9 Hz, 2H), 1.81 – 1.73 (m, 1H) ppm.
^{13}C NMR (126 MHz, DMSO-d$_6$): δ_C = 172.9, 171.4, 168.7, 149.2, 144.0, 140.8 (d, *J*=2.5 Hz), 134.4, 130.7, 126.6, 125.8, 124.7, 123.8, 118.7 (d, *J*=2.0 Hz), 112.3 (d, *J*=2.9 Hz), 105.5 (d, *J*=4.4 Hz), 59.6 (d, *J*=3.3 Hz), 51.3 (d, *J*=7.3 Hz), 48.9, 46.8 (d, *J*=11.0 Hz), 33.2, 31.3, 22.6 (d, *J*=3.4 Hz), 22.3 (d, *J*=2.0 Hz), 18.9 ppm.
HRMS (ESI): *m/z* calc. for $C_{24}H_{26}N_3O_3$ [M+H]$^+$: 404.1969; found: 404.1975.

1.5 Mechanistic investigations

1.5.1 Reaction in absence of aniline (Scheme 5a), kinetic evaluation, byproduct **8** characterization

The reaction was performed according to the general procedure for *N*–alkylation in an oven dried pressure tube with the activated catalyst deposited and dried on the surface, without addition of the aniline **1a** at room temperature. The reaction proceeds to full conversion via cyclodehydration to byproduct **8a**, as shown in Figure S2.

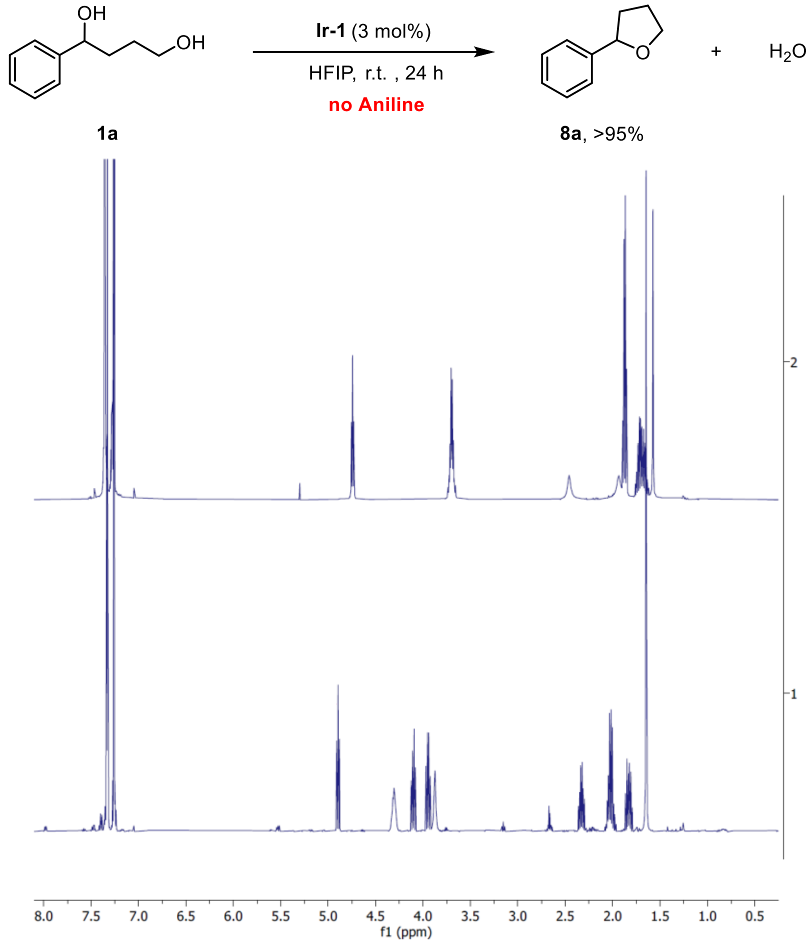

Figure S2. Cyclodehydration of 1,4–diol **1a**. Starting material at top, in comparison with the reaction crude at bottom. Full conversion to **8a**.

The cyclodehydration reaction was studied via Hammett plot evaluation. Few substrates shown in Figure S3 were used in the evaluation with parallel and independent reactions. The evaluation was performed according to the following procedure. An oven dried NMR tube impregnated with the activate catalyst (0.002 mmol, 1 mol%) was loaded with appropriate 1,4–diol (0.2 mmol) and dry HFIP (0.5 mL, 0.4 M). Kinetic evaluation was made with an *in situ* no–D NMR[17] monitoring for the conversion up to 10%, acquiring the spectrum every 2, 4 or 6 minutes depending on the substrate. The initial rates were used to build the Hammett plot seen in Figure 7.3.

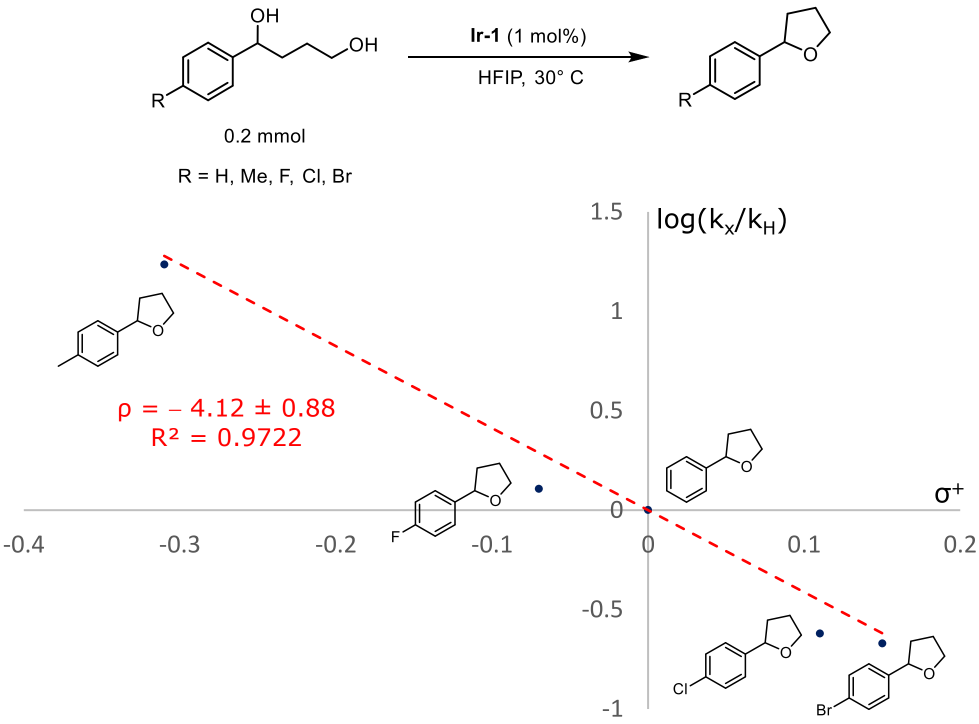

Figure S3. Hammett plot study using *para*–substituted 1,4–diols.

A ρ value of –4.12 is indicative of an S_N1 type of mechanism, where the iridium complex is acting as a Lewis acid.

As per confirmation, side products **8a-8k** were synthetized from the corresponding 1,4-diol according the following procedure.

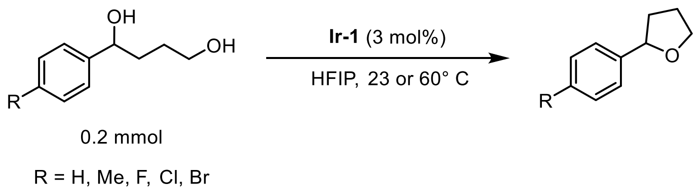

The pressure tube impregnated with the activated catalyst (0.006 mmol, 3 mol%) under air was loaded with dry HFIP (0.5 mL, 0.4 M) and the corresponding 1,4–diol (0.2 mmol, 1 equiv.) was added to the reaction mixture, the pressure tube was sealed and let stir at room temperature or put in a pre–heated oil bath at 60 °C for 18 h. The pressure tube was then removed from the heating bath and let cool down, opened and the solvent was removed under reduced pressure. The reaction crude was filtered through a pad of silica giving the desired THF byproduct.

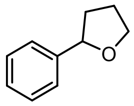

2-phenyltetrahydrofuran (8a)

Colorless oil, 28mg, 95% yield.

^{1}H NMR (400 MHz, $CDCl_3$): δ_H = 7.39 – 7.33 (m, 4H), 7.32 – 7.25 (m, 1H), 4.93 (t, *J* = 7.2 Hz, 1H), 4.13 (dt, *J* = 8.0, 6.8 Hz, 1H), 3.97 (td, *J* = 7.9, 6.4 Hz, 1H), 2.36 (dtd, *J* = 12.4, 7.1, 5.4 Hz, 1H), 2.04 (dddd, *J* = 14.8, 7.4, 5.9, 3.3 Hz, 2H), 1.84 (ddt, *J* = 12.1, 8.6, 7.5 Hz, 1H) ppm.

^{13}C NMR (101 MHz, $CDCl_3$): δ_C = 143.6, 128.4, 127.2, 125.7, 80.8, 68.8, 34.7, 26.2 ppm.

The spectroscopic data is in agreement with those reported in the literature.[18]

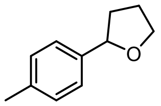

2-(p-tolyl)tetrahydrofuran (8l)
Colorless oil, 31 mg, 95% yield.
^{1}H NMR (400 MHz, $CDCl_3$): δ_H = 7.24 (d, J = 8.0 Hz, 2H), 7.15 (d, J = 7.9 Hz, 2H), 4.87 (t, J = 7.2 Hz, 1H), 4.09 (dt, J = 8.1, 6.7 Hz, 1H), 3.93 (td, J = 7.9, 6.3 Hz, 1H), 2.35 (s, 3H), 2.33 – 2.26 (m, 1H), 2.09 – 1.92 (m, 2H), 1.80 (ddt, J = 12.1, 8.6, 7.5 Hz, 1H) ppm.
^{13}C NMR (101 MHz, $CDCl_3$): δ_C = 140.5, 136.8, 129.1, 125.7, 80.7, 68.7, 34.7, 26.2, 21.2 ppm.
The spectroscopic data is in agreement with those reported in the literature. [18]

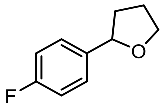

2-(4-fluorophenyl)tetrahydrofuran (8i)
Colorless oil, 30.7 mg, 92% yield.
^{1}H NMR (400 MHz, $CDCl_3$): δ_H = 7.30 (dd, J = 8.4, 5.5 Hz, 2H), 7.01 (t, J = 8.7 Hz, 2H), 4.85 (t, J = 7.2 Hz, 1H), 4.09 (q, J = 7.1 Hz, 1H), 3.92 (q, J = 7.4 Hz, 1H), 2.37 – 2.24 (m, 1H), 2.08 – 1.92 (m, 2H), 1.76 (dq, J = 12.1, 7.9 Hz, 1H) ppm.
^{13}C NMR (101 MHz, $CDCl_3$): δ_C = 162.13 (d, J = 244.5 Hz), 139.21 (d, J = 3.2 Hz), 127.38 (d, J = 8.0 Hz), 115.19 (d, J = 21.3 Hz), 80.3, 68.7, 34.8, 26.1 ppm.
^{19}F NMR (377 MHz, $CDCl_3$): δ_F = –115.9 ppm
The spectroscopic data is in agreement with those reported in the literature.[18]

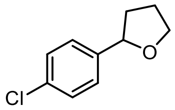

2-(4-chlorophenyl)tetrahydrofuran (8j)
Reaction done at 60 °C. Colorless oil, 35 mg, 95% yield.
^{1}H NMR (400 MHz, $CDCl_3$): δ_H = 7.37 – 7.26 (m, 4H), 4.88 (t, J = 7.2 Hz, 1H), 4.17 – 4.06 (m, 1H), 3.95 (dt, J = 8.3, 6.9 Hz, 1H), 2.41 – 2.26 (m, 1H), 2.09 – 1.95 (m, 2H), 1.83 – 1.72 (m, 1H) ppm.
^{13}C NMR (101 MHz, $CDCl_3$): δ_C = 142.0, 132.7, 128.4, 127.0, 80.0, 68.7, 34.7, 26.0 ppm.
The spectroscopic data is in agreement with those reported in the literature.[18]

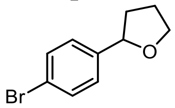

2-(4-bromophenyl)tetrahydrofuran (8k)
Reaction done at 60 °C. Colorless oil, 35.1 mg, 77% yield.
^{1}H NMR (400 MHz, $CDCl_3$): δ_H = 7.47 – 7.42 (m, 2H), 7.21 (dt, J = 7.4, 0.7 Hz, 2H), 4.84 (t, J = 7.2 Hz, 1H), 4.08 (dt, J = 8.2, 6.8 Hz, 1H), 3.93 (dt, J = 8.3, 6.9 Hz, 1H), 2.38 – 2.25 (m, 1H), 2.07 – 1.92 (m, 2H), 1.81 – 1.67 (m, 1H) ppm.
^{13}C NMR (101 MHz, $CDCl_3$): δ_C = 142.7, 131.5, 127.5, 120.9, 80.1, 77.2, 68.9, 34.8, 26.1 ppm.
The spectroscopic data is in agreement with those reported in the literature.[18]

1.5.2 Reaction with Phenyl THF instead of 1,4-diol

The reaction was performed according to the general procedure for the *N*–alkylation of anilines

with 1,4–diols, using 1 equivalent of Phenyl THF (**8a**) instead of the 1,4-diol, and 1 equiv of water. In the crude reaction (shown in Figure S4) are clearly visible the unreacted starting materials **8a** and **2a**, and the desired δ-amino alcohol is not detected.

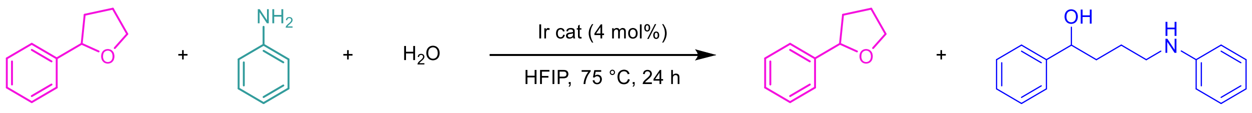

8a **2a** **8a**, 100% **3aa**, n.d.

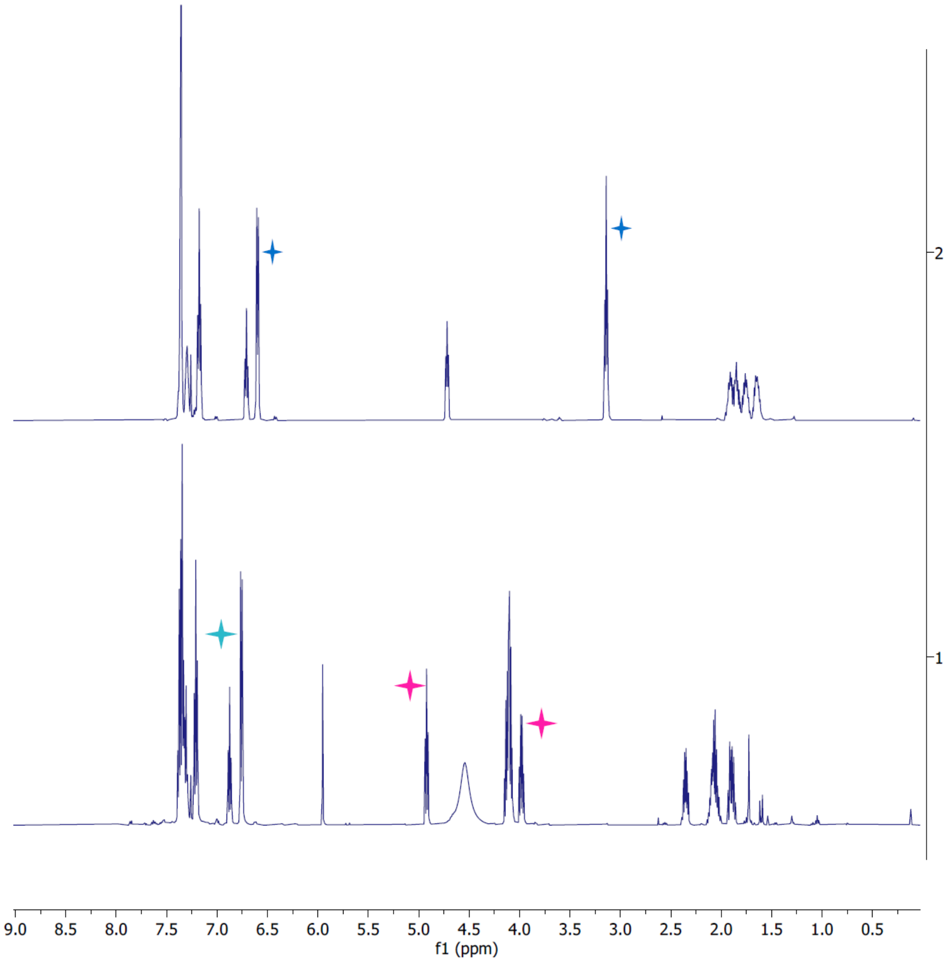

Figure S4 . Comparison between the ^{1}H NMR of the δ-amino alcohol **3aa** with the ^{1}H NMR of the crude reaction described above. Only unreacted starting material are present in the crude reaction.

1.5.3 Test reaction with chiral (*S*)-1,4-diol ((*S*)-**1a**) (Scheme 5b)

The test reaction was performed according to the general procedure for the *N*–alkylation of anilines using 0.1 mmol of aniline **2a** and 2 equivalents of the chiral 1,4–diol ((*S*)-**1a**).

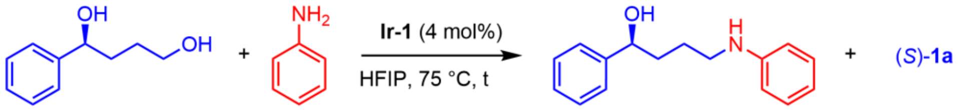

(*S*)-**1a** (99% *ee*)
2 equiv.

2a
1 equiv.
(0.1 mmol)

(*S*)-**3aa**, 24 h: 75% (98% *ee*)
(*S*)-**3aa**, 1h: 28% (99% *ee*)

24 h: 0.45 equiv. isolated
(93% *ee*)
1 h: 0.98 equiv. isolated
(98% *ee*)

In the test reaction after 24 h the product (*S*)-**3aa** has an enantiomeric excess of 98%, meanwhile in the test reaction after 1 h the product (*S*)-**3aa** has an enantiomeric excess of 99% (Figure S5).

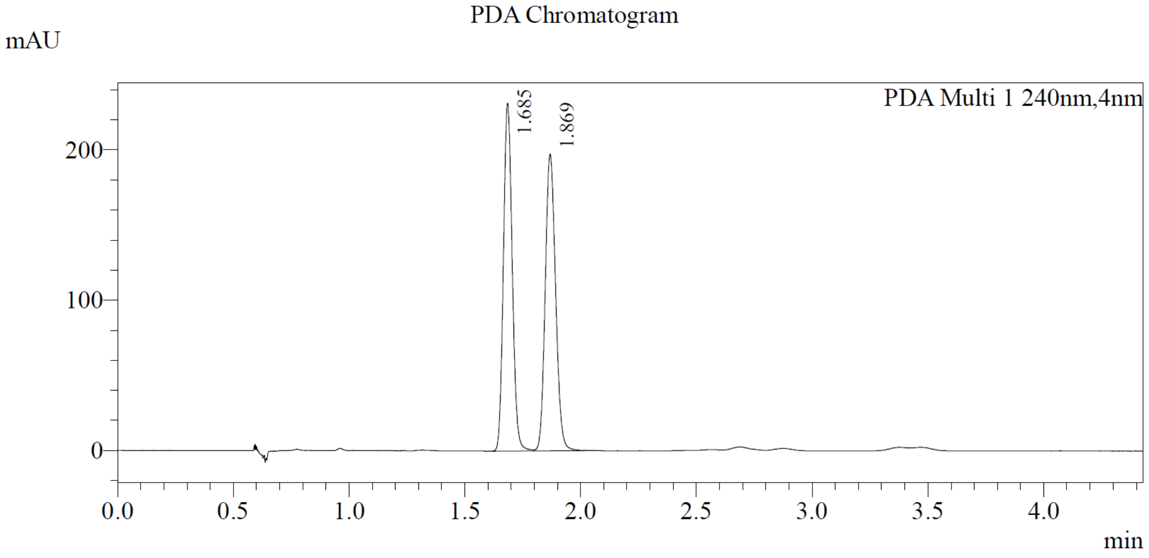

Peak Table

PDA Ch1 240nm

Peak#	Ret. Time	Area	Height	Area%
1	1.685	612420	226116	49.931
2	1.869	614118	194584	50.069

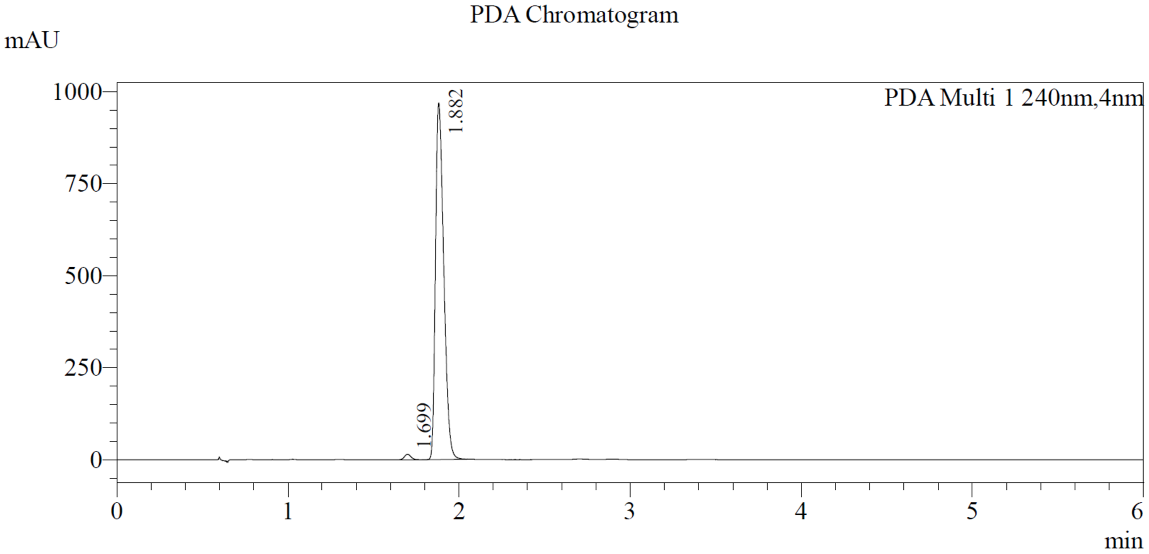

Peak Table

PDA Ch1 240nm

Peak#	Ret. Time	Area	Height	Area%
1	1.699	39453	14631	1.201
2	1.882	3246171	956784	98.799

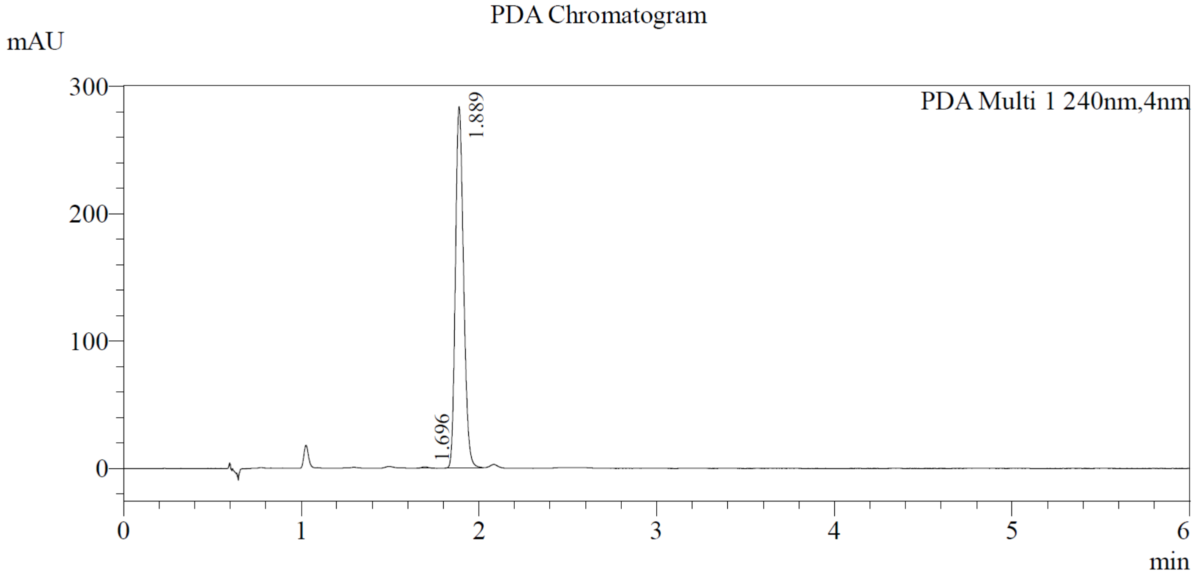

Peak Table

PDA Ch1 240nm

Peak#	Ret. Time	Area	Height	Area%
1	1.696	2887	1123	0.317
2	1.889	908266	281913	99.683

Figure S5. SFC chromatogram of the racemic mixture of product **3aa** (up), product (*S*)-**3aa** after 24 h reaction (middle), product (*S*)-**3aa** after 1h reaction (down).

The enantiomeric excess of the recovered starting material (*S*)-**1a** was determined on a Shimadzu SFC Prep equipped with a Lux C4 column (150 x 4.6, particle size 3 µm) and a mobile phase of 30% IPA/DEA 100/20mM in CO_2 120 bar, 3.5 ml/min, λ = 220 nm. The recovered starting material after 24 h an enantiomeric excess of 93%, whereas just after 1 h the sample an enantiomeric excess of 98.8%, as seen in Figure S6.

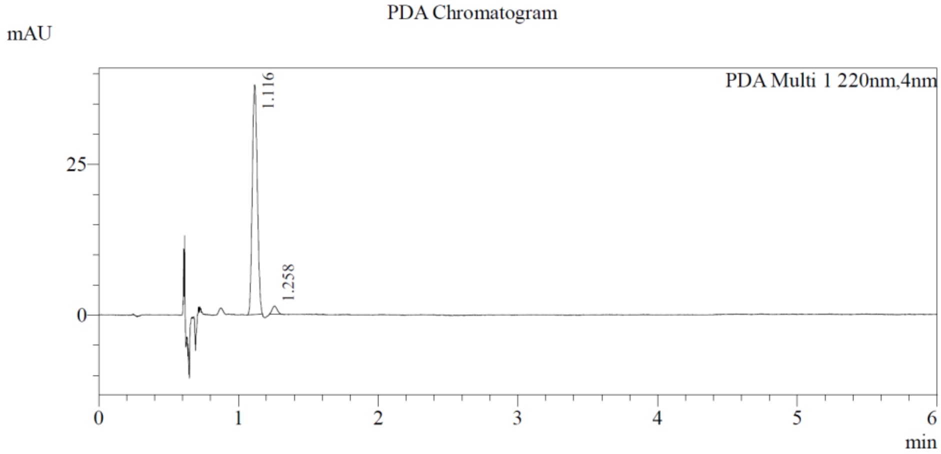

Peak Table

PDA Ch1 220nm

Peak#	Ret. Time	Area	Height	Area%
1	1.116	95164	37021	96.500
2	1.258	3451	1317	3.500

93% ee

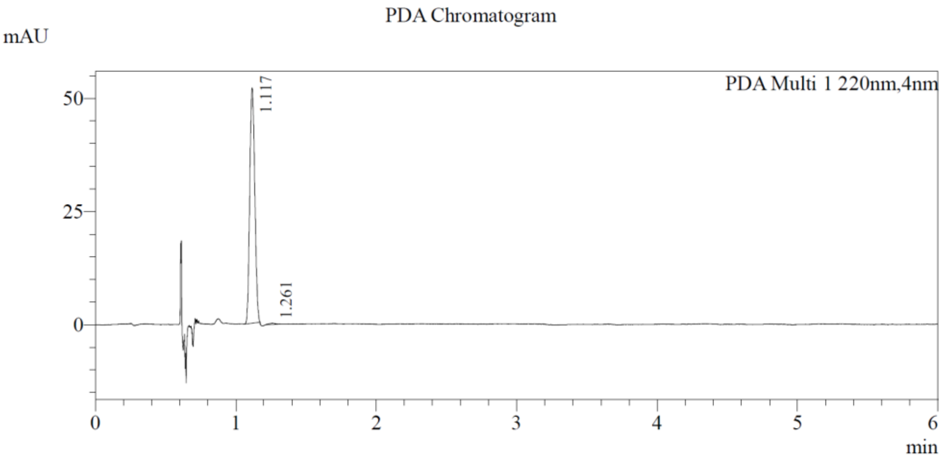

Peak Table

PDA Ch1 220nm

Peak#	Ret. Time	Area	Height	Area%
1	1.117	129552	50244	99.422
2	1.261	753	251	0.578

98% ee

Figure S6. SFC chromatogram of the recovered chiral diol (*S*)-**1a** after 24 h (up) and after 1 h (down), from Scheme 5b.

1.5.4 Control experiments with deuterated substrates or solvent (Scheme 5c)

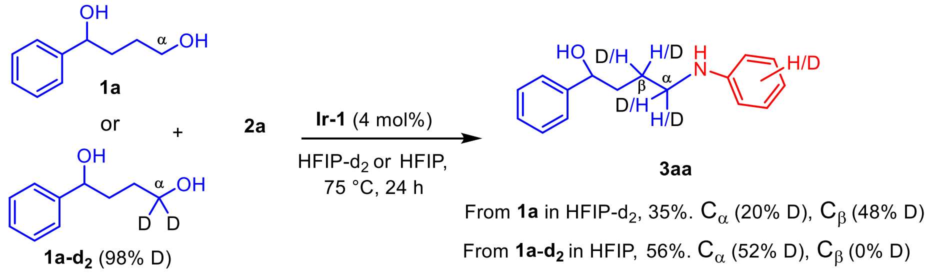

The reaction was performed according to the general procedure for the *N*–alkylation of anilines with 1,4–diols. Parallel and independent reactions containing the diol **1a** in HFIP-d2 and **1a–d2** in HFIP respectively, were tested under standard conditions reported. In Figure S7, the NMR of the products **3aa** from **1a** in HFIP-d2. Clear deuterium incorporation in Cα- and Cβ positions are visible from ^{1}H NMR, along deuteration in the *ortho*- and *para*-positions of the aniline side. In Figure S8, the NMR of the products **3aa** from **1a-d2** in HFIP. In the ^{1}H NMR, proton incorporation in Cα- position is detected. In Figure S9, the ^{1}H NMR of the recovered diol **1a**, from the reaction in HFIP-d2, no deuterium incorporation is visible in Cα- position. In Figure S10, the ^{1}H NMR of the recovered deuterated diol **1a-d2**, from the reaction in HFIP. Deuterium incorporation in Cα- position remains the original 98% D.

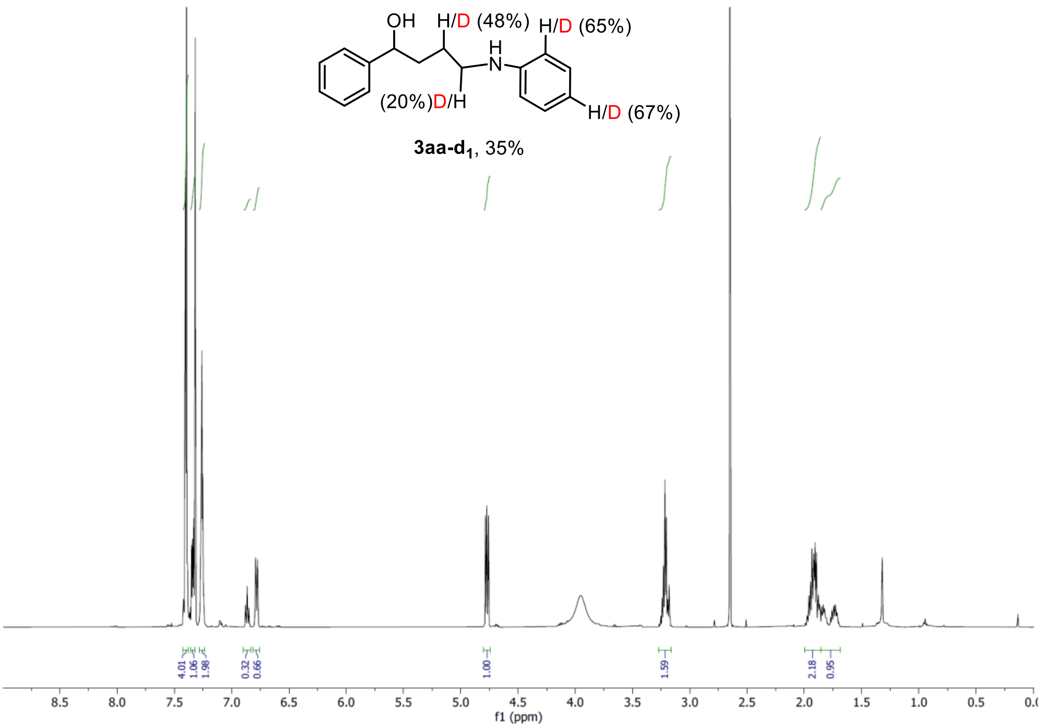

Figure S7. ^{1}H NMR of the product **3aa–d₁** from the reaction with **1a** in HFIP-d_2. Deuteration in Cα- and Cβ position of the product is observed.

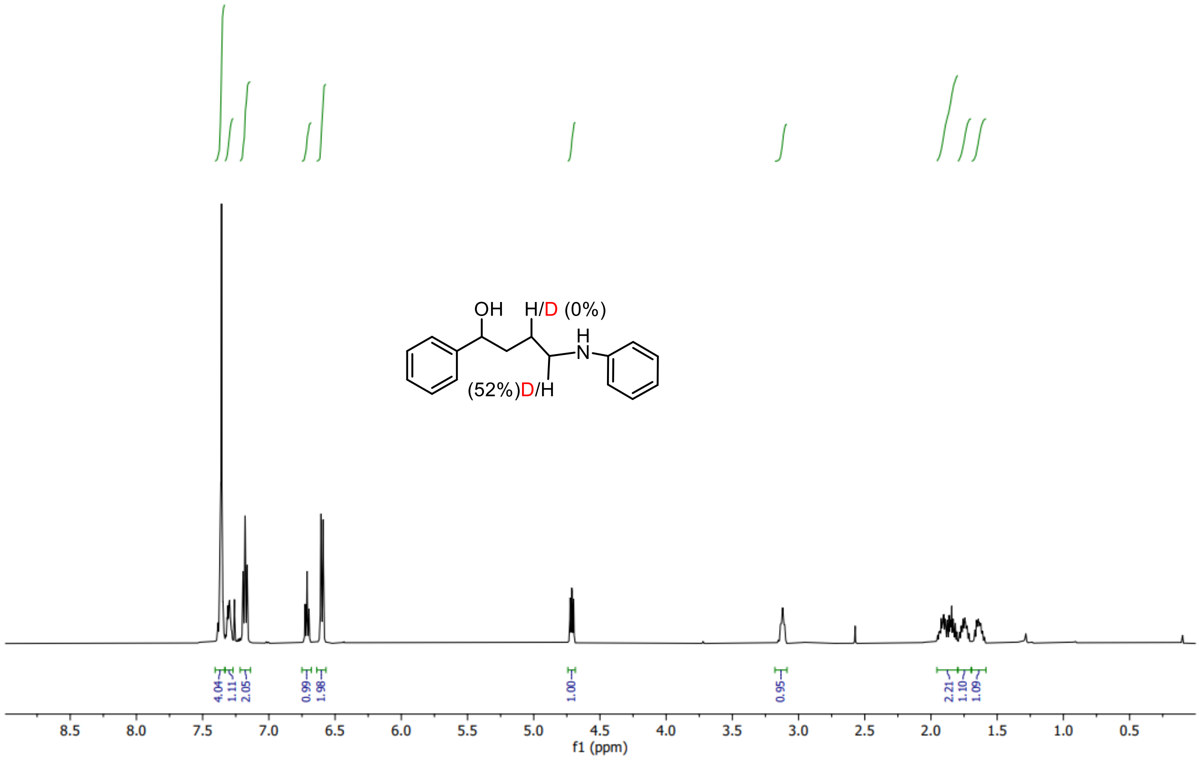

Figure S8. ^{1}H NMR of the product **3aa–d₂** from the reaction with **1a–d₂** in HFIP. Proton incorporation in *α* position of the product is observed.

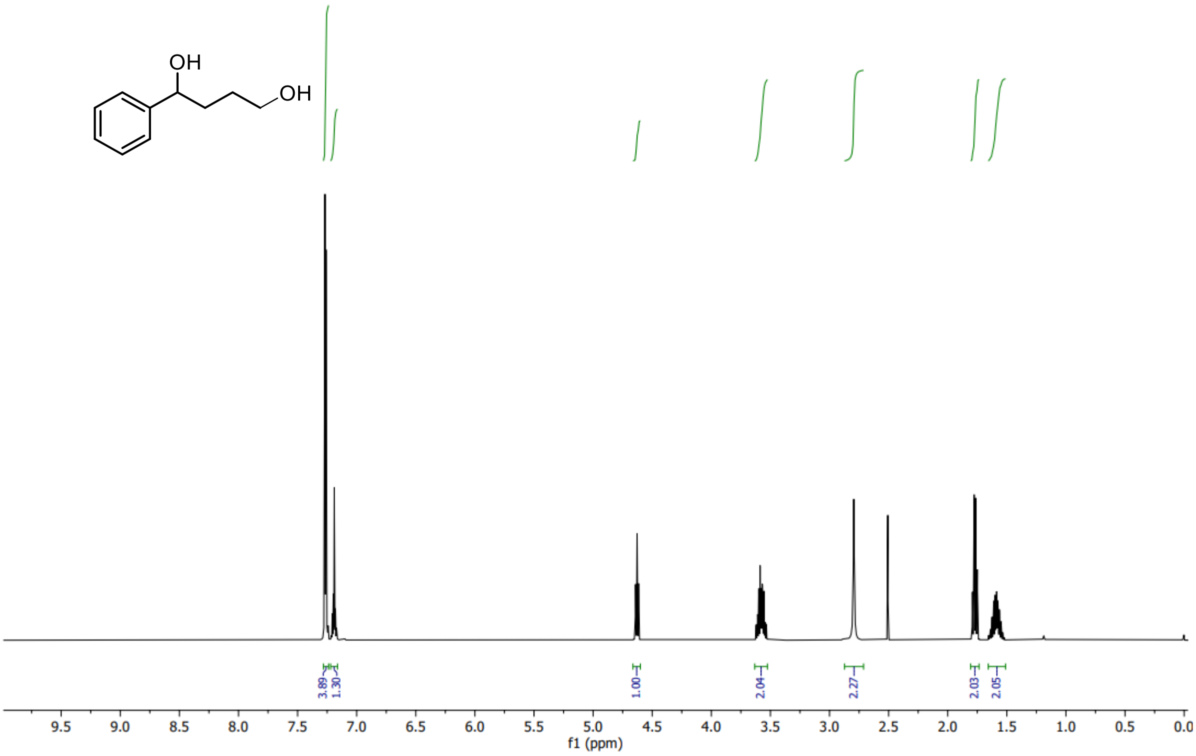

Figure S9. ^{1}H NMR of the recovered starting material **1a** after the reaction in HFIP-d_2. The signal at 3.6 ppm from the *CH_2*OH is still full protonated, so no deuterium incorporation from the solvent.

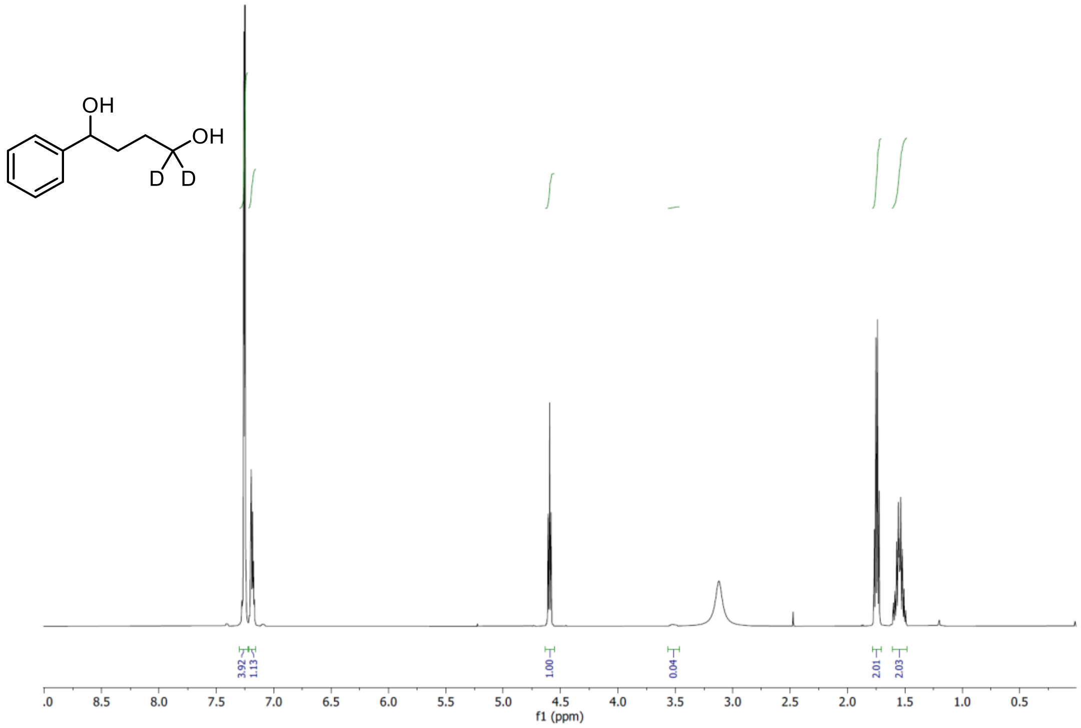

Figure S10. ^{1}H NMR of the recovered starting material **1a–d₂**. The compound is still full deuterated (98% D).

1.6 Biological tests on Lenalidomide functionalized products

The Lenalidomide products (**7ac – 7dd**) were analyzed towards CRBN binding assay, including cytotoxicity determination.

Table S3. Purity, physicochemical properties, VHL binding and Cytotoxic of selected compounds.

Substrate	UV purity (%)	Solubility pH=7.4 (µM)	ChromLogD pH=7.4	ePSA (Å)	CRBN binding IC_{50} (nM)	Cytotoxicity IC_{50} (µM)
**Lenalidomide**	99	>1000	N.D	98	100	>250
**7ac**	96	49	1.9	113	68	>100
**7cc**	97	136	2.8	117	54	>100
**7kc**	95	123	2.9	118	48	>100
**C5 Lenalidomide**	95	>1000	N.D	N.D	28	N.D
**7ad**	97	841	1.4	116	20	>100
**7cd**	98	137	2.3	119	12	>100
**7kd**	97	119	2.4	120	15	>100
**7dd**	97	82	3.9	104	10	>100

N.D = Not Determined. In house protocol was used for determination of physicochemical properties. CRBN binding was determined in a time-resolved-FRET competition assay using a Cy5-labeled CRBN ligand. Cytotoxicity was determined in a human monocytic cell line (THP1) using Resazurin reagent.

1.7 NMR spectra of compounds

1.7.1 Spectra of 1,4-diols starting material (**1**)

1-phenylbutane-1,4-diol (1a)

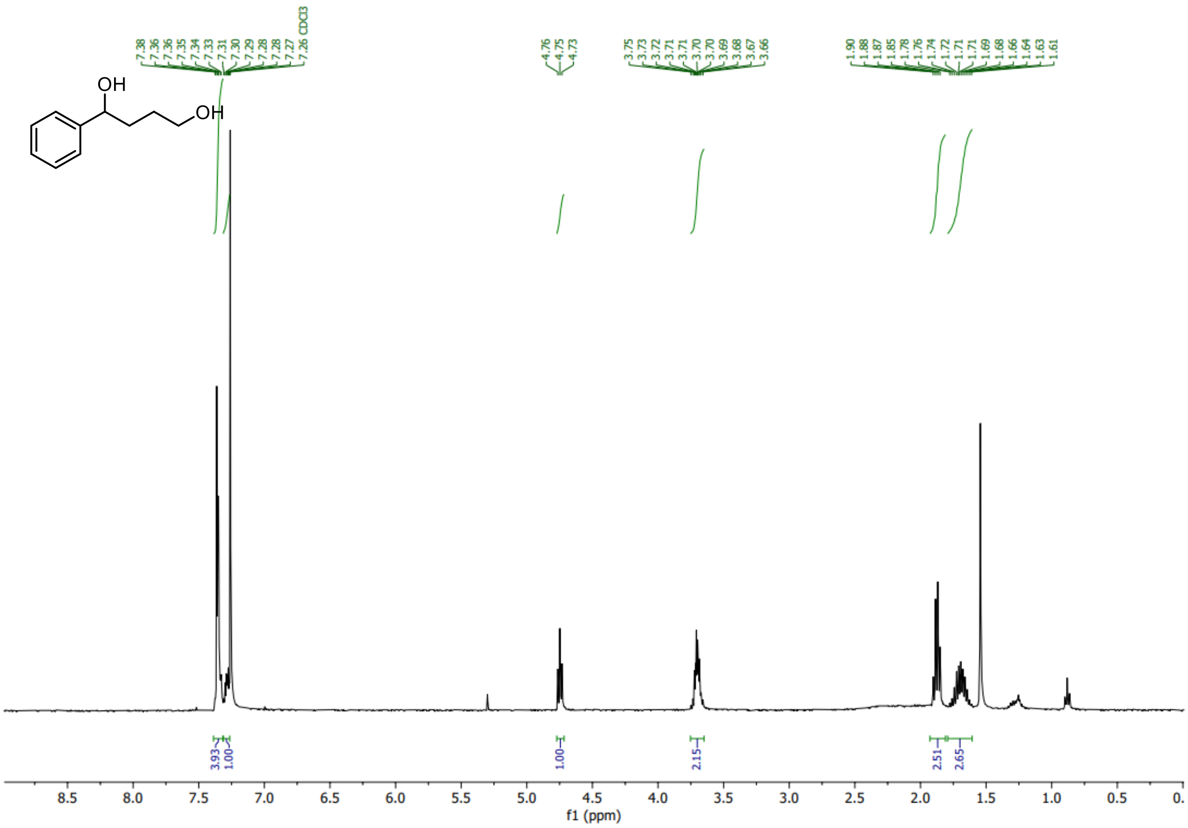

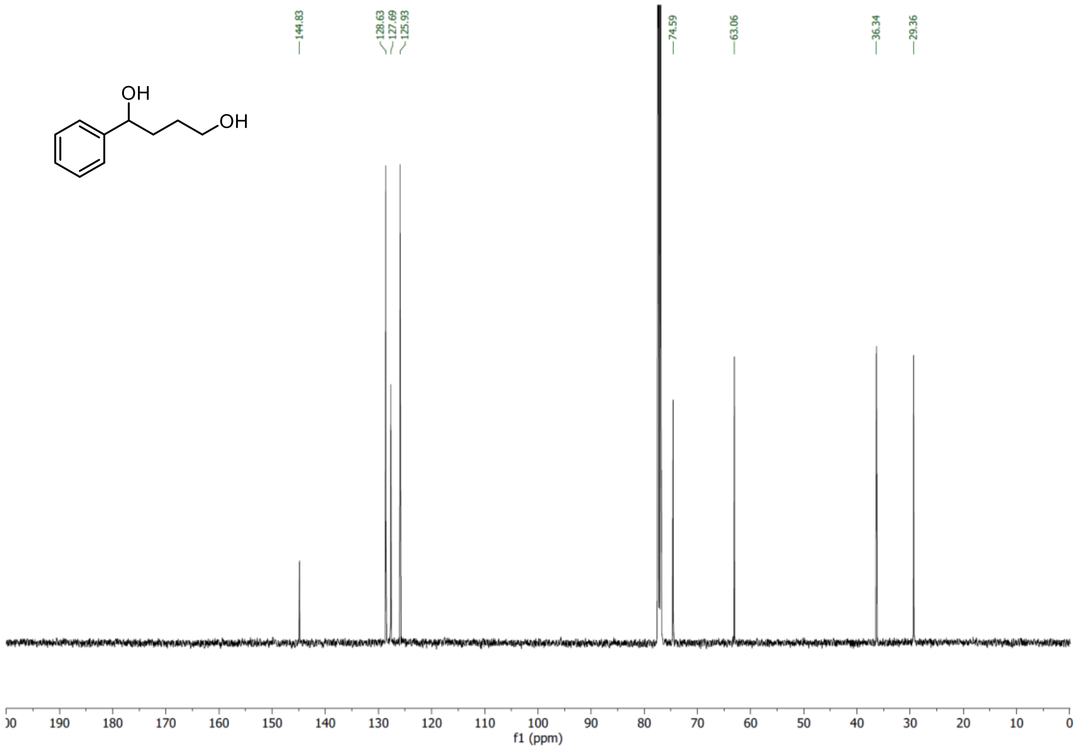

Figure S11. (Top) ^{1}H NMR (500 MHz) and (bottom) $^{13}C\{^{1}H\}$ NMR (126 MHz) spectra of **1a** in $CDCl_3$.

1–(2–chlorophenyl)butane–1,4–diol (1b)

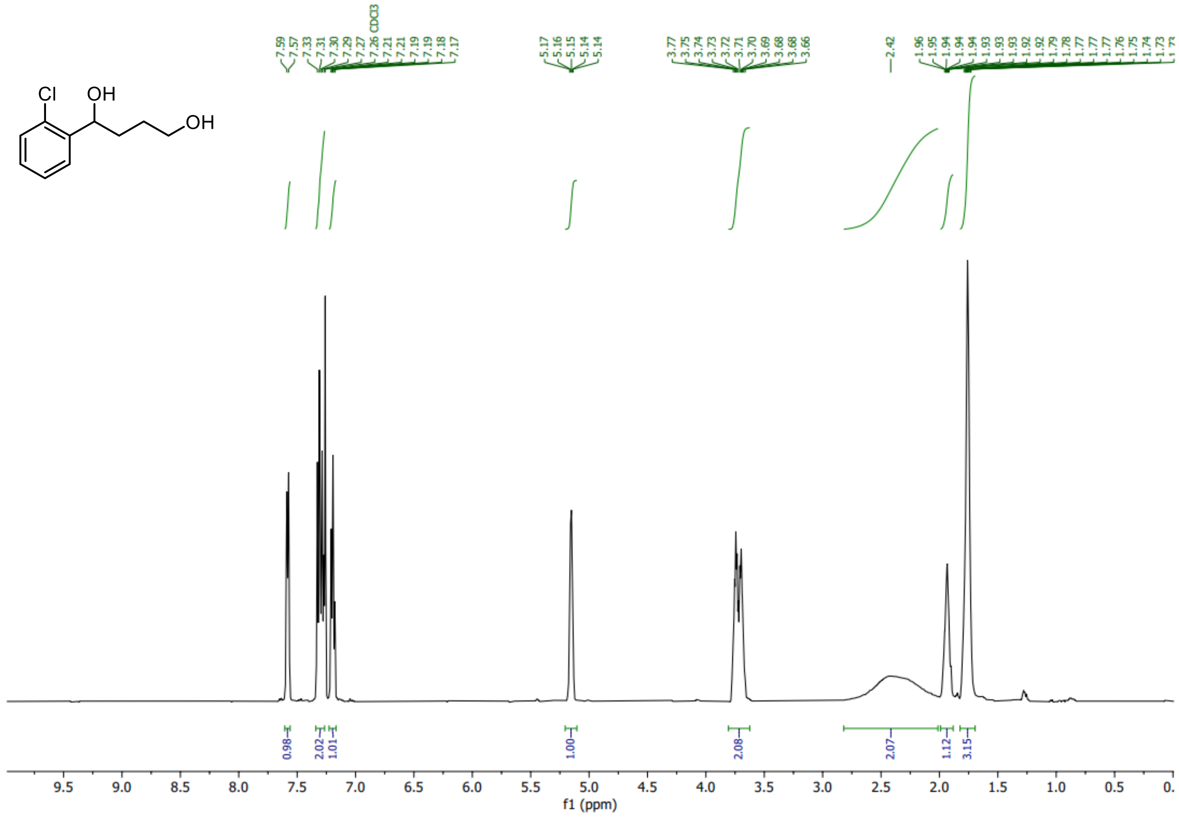

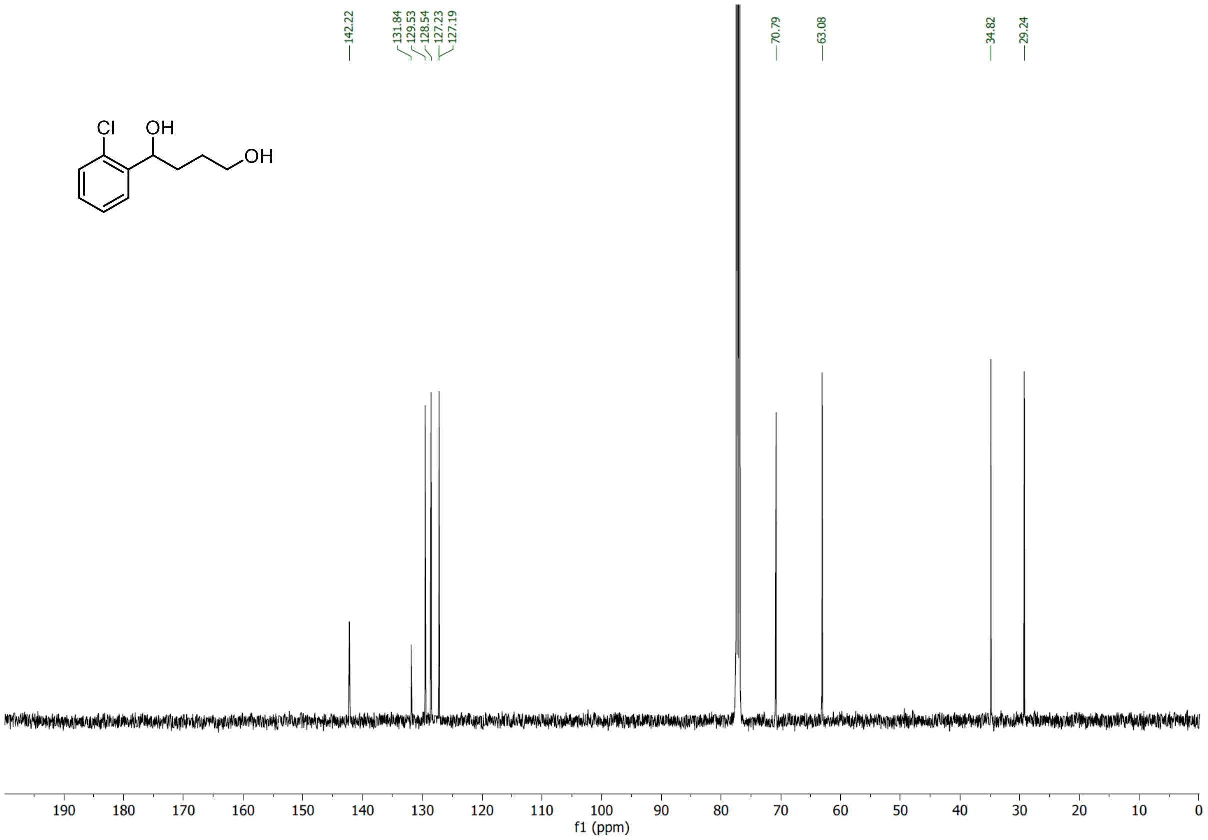

Figure S12. (Top) ^{1}H NMR (500 MHz) and (bottom) $^{13}C\{^{1}H\}$ NMR (126 MHz) spectra of **1b** in $CDCl_3$.

1–(2–bromophenyl)butane–1,4–diol (1c)

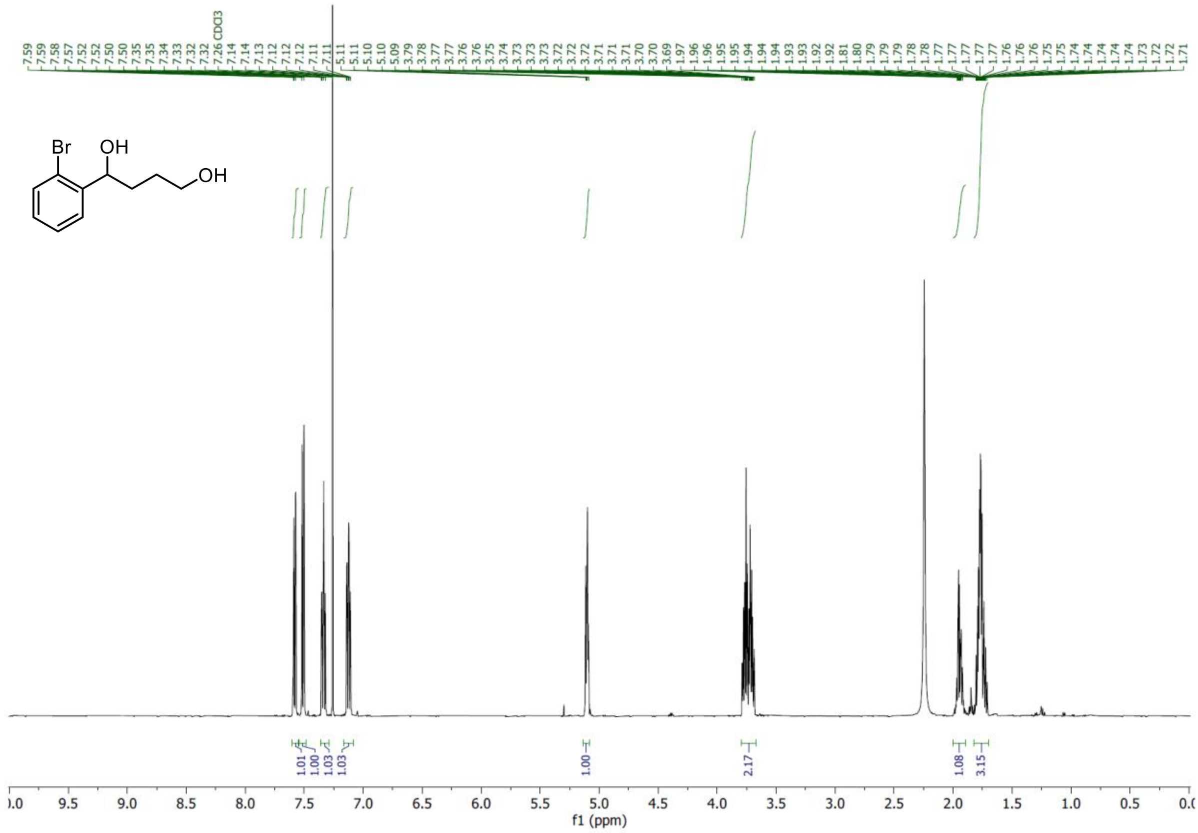

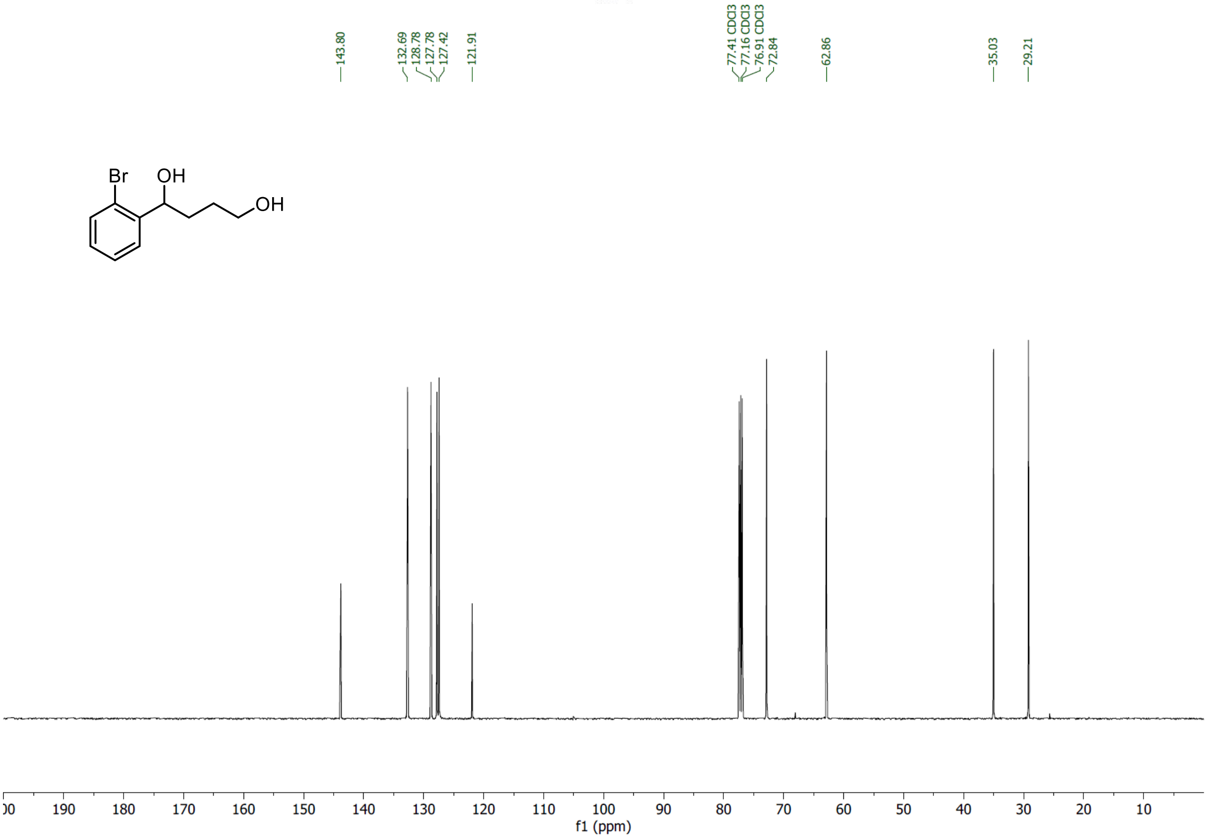

Figure S13. (Top) ^{1}H NMR (500 MHz) and (bottom) $^{13}C\{^{1}H\}$ NMR (126 MHz) spectra of **1c** in $CDCl_3$.

1–(o–tolyl)butane–1,4–diol (1d)

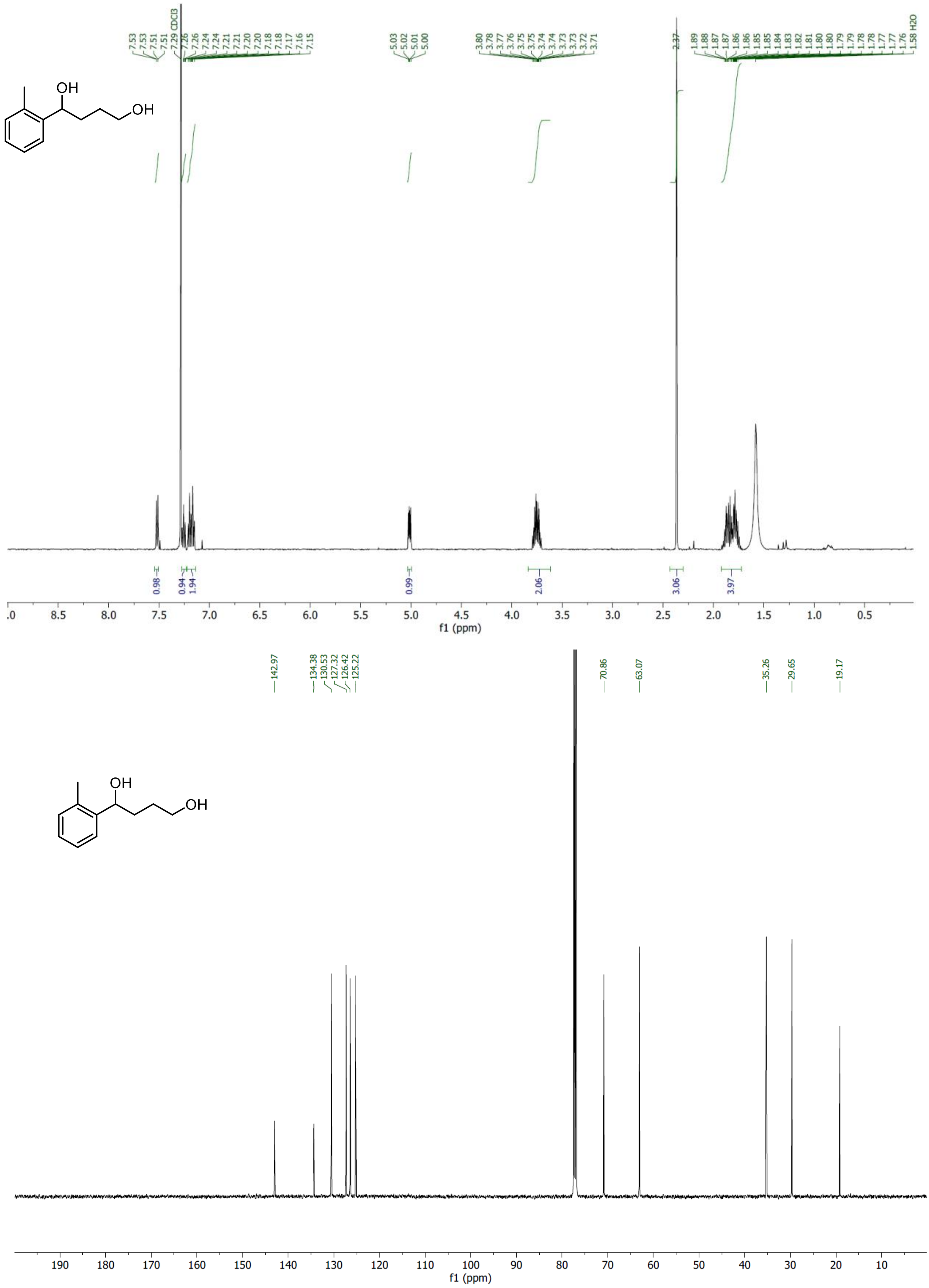

Figure S14. (Top) ^{1}H NMR (500 MHz) and (bottom) ^{13}C{^{1}H} NMR (126 MHz) spectra of **1d** in $CDCl_3$.

1–(2–methoxyphenyl)butane–1,4–diol (1e)

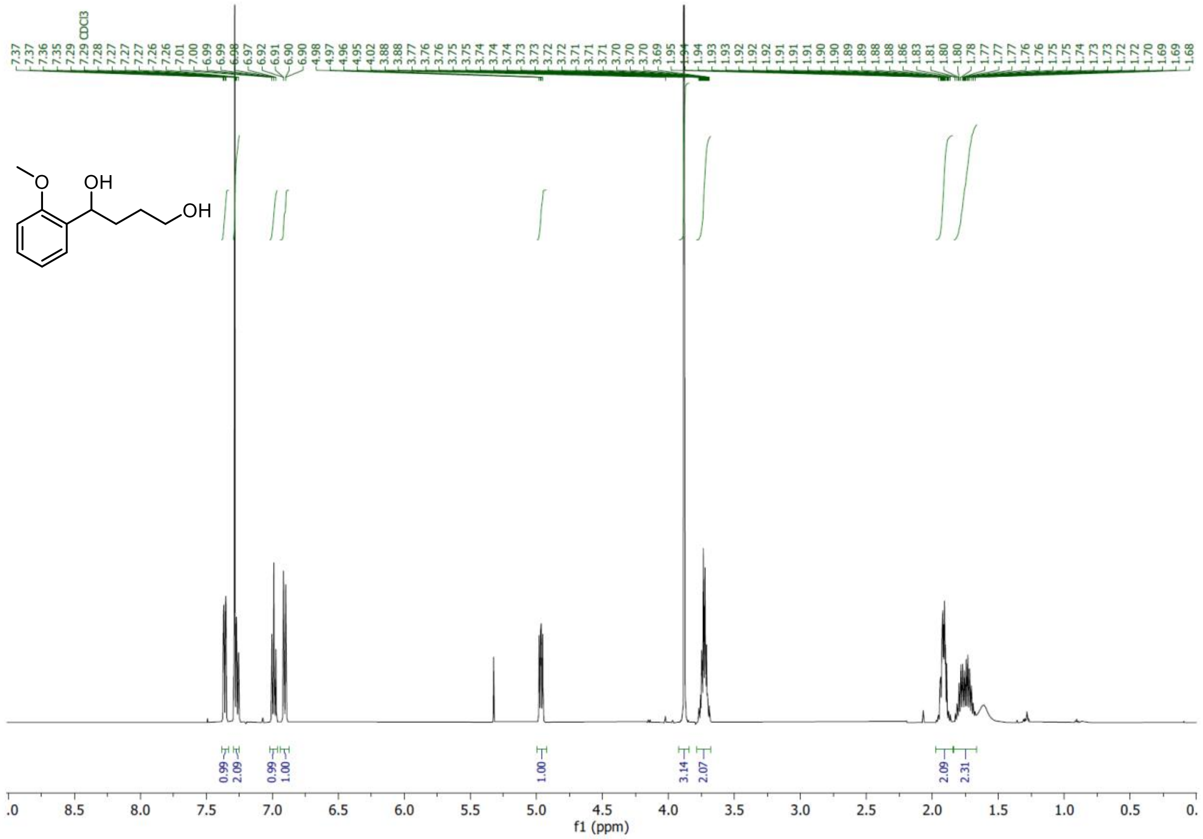

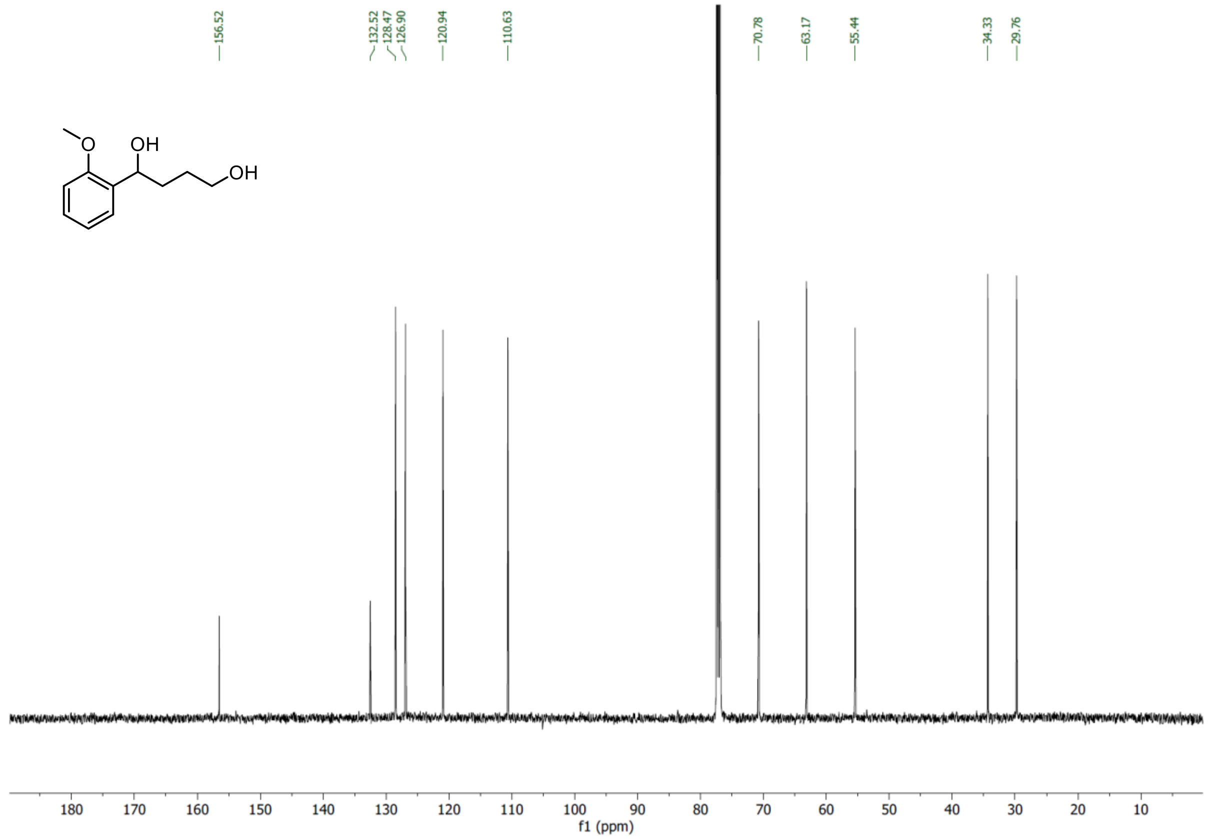

Figure S15. (Top) ^{1}H NMR (500 MHz) and (bottom) $^{13}C\{^{1}H\}$ NMR (126 MHz) spectra of **1e** in $CDCl_3$.

1–(3–methoxyphenyl)butane–1,4–diol (1f)

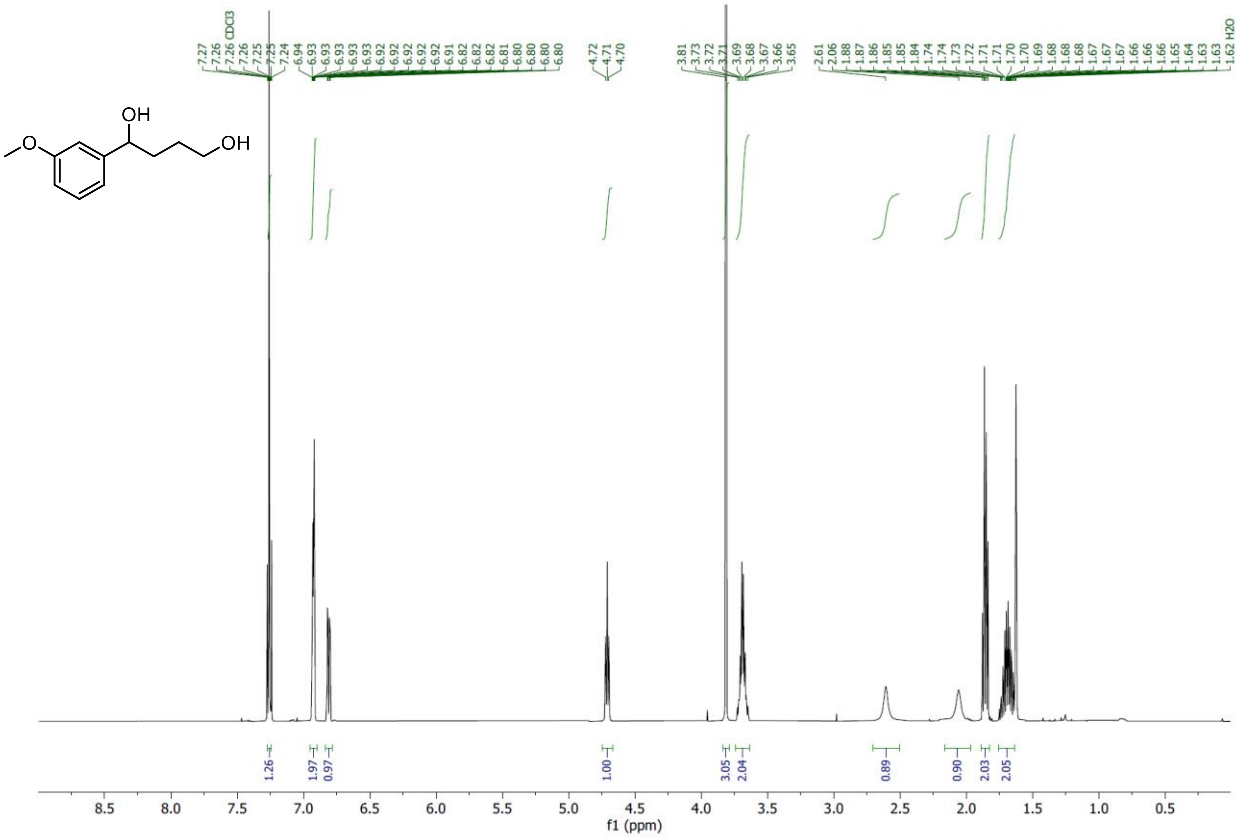

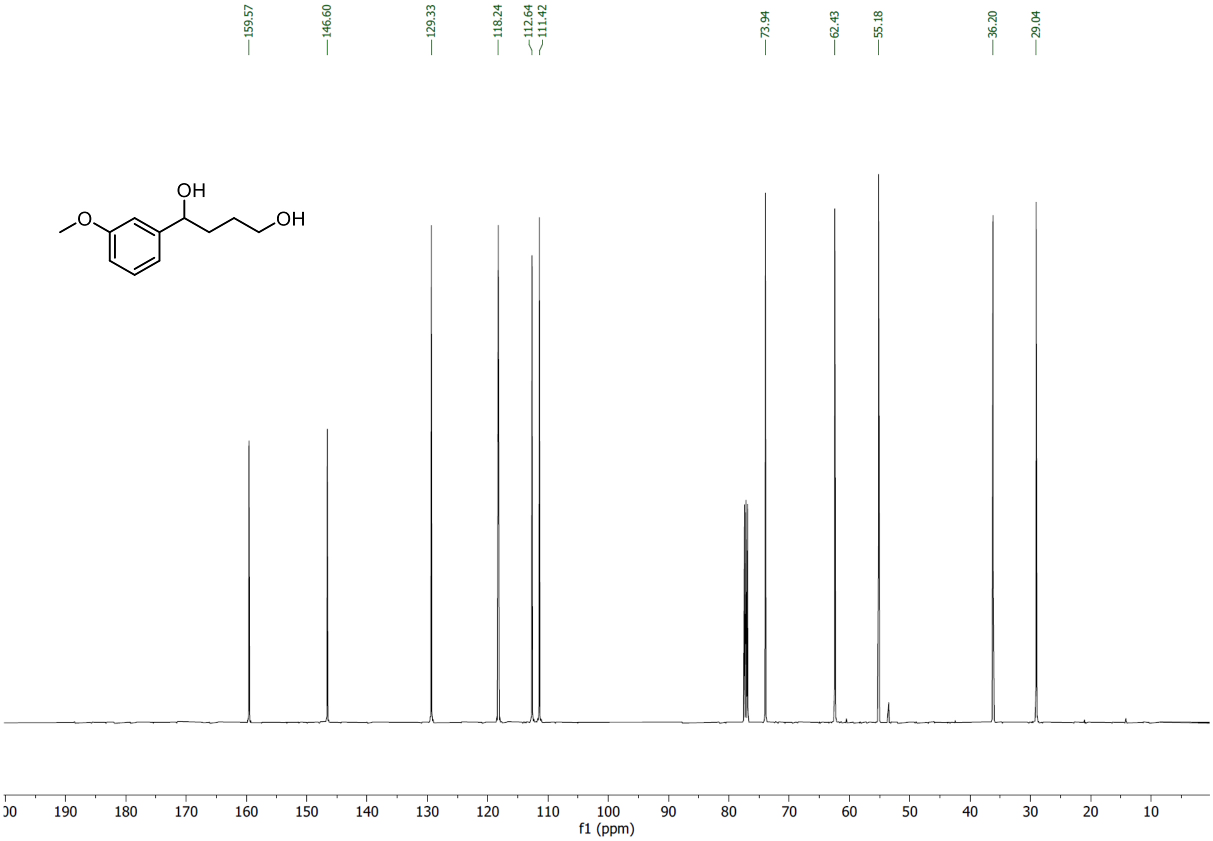

Figure S16. (Top) ^{1}H NMR (500 MHz) and (bottom) ^{13}C{^{1}H} NMR (126 MHz) spectra of **1f** in $CDCl_3$.

1-(m-tolyl)butane-1,4-diol (1g)

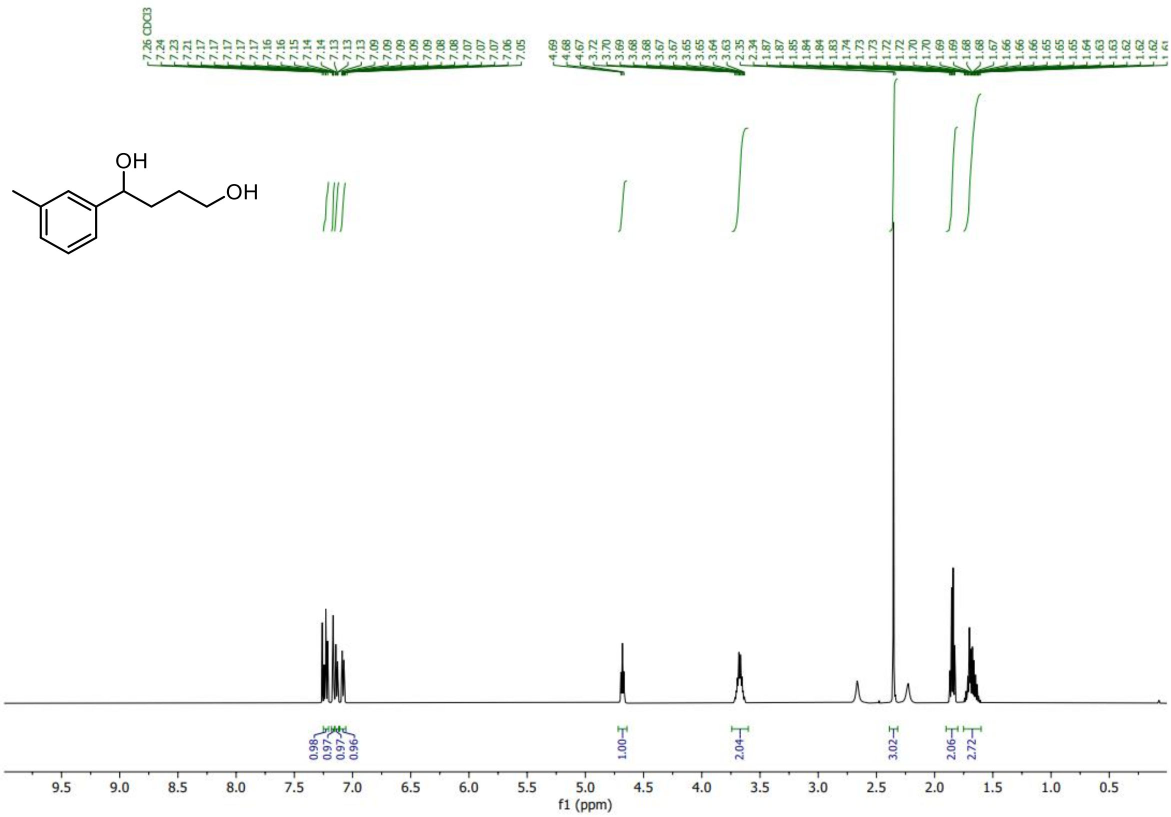

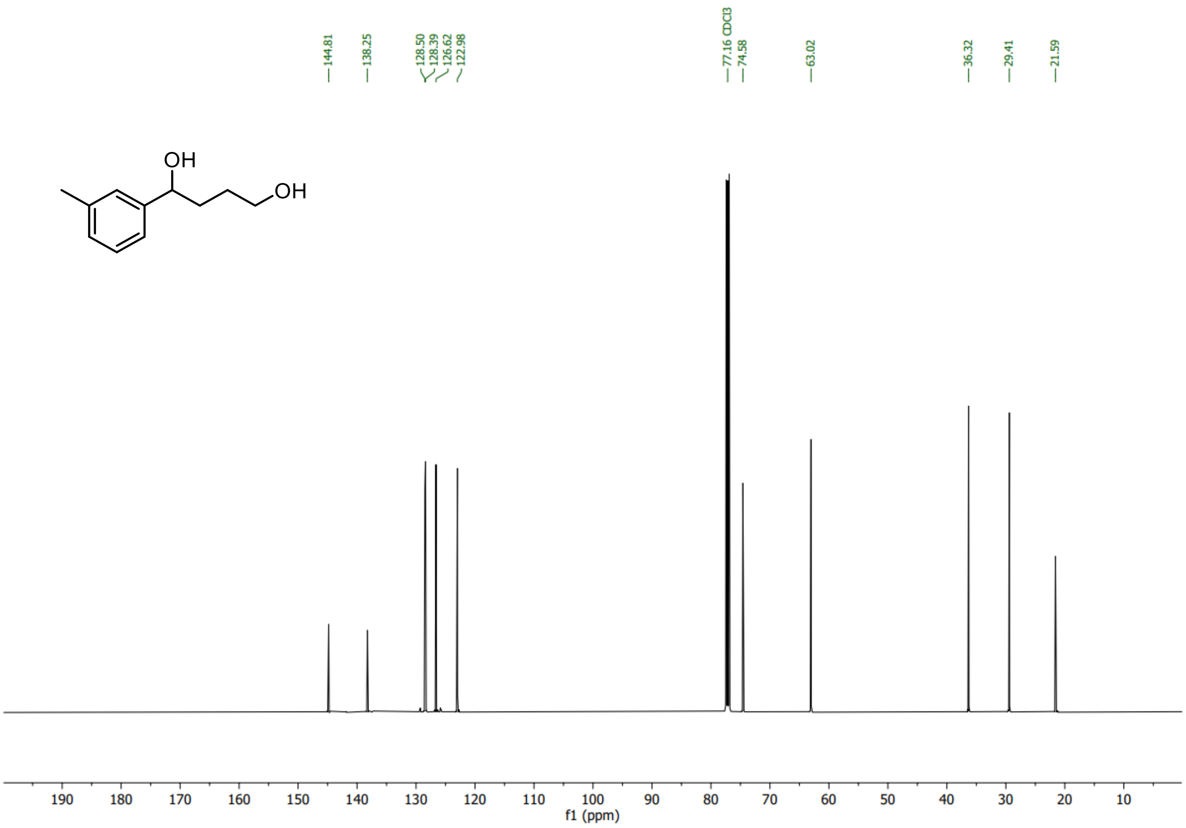

Figure S17. (Top) ^{1}H NMR (500 MHz) and (bottom) $^{13}C\{^{1}H\}$ NMR (126 MHz) spectra of **1g** in $CDCl_3$.

1–(naphthalen–2–yl)butane–1,4–diol (1h)

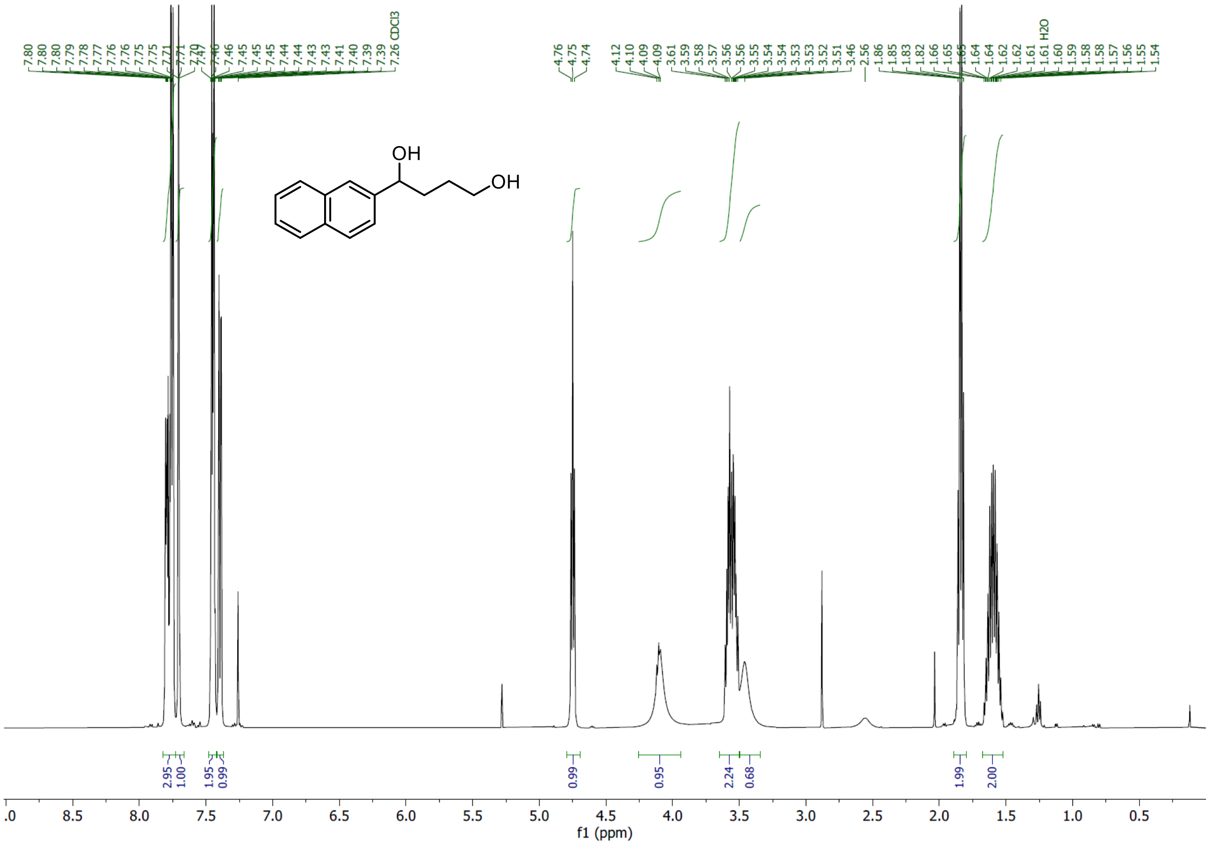

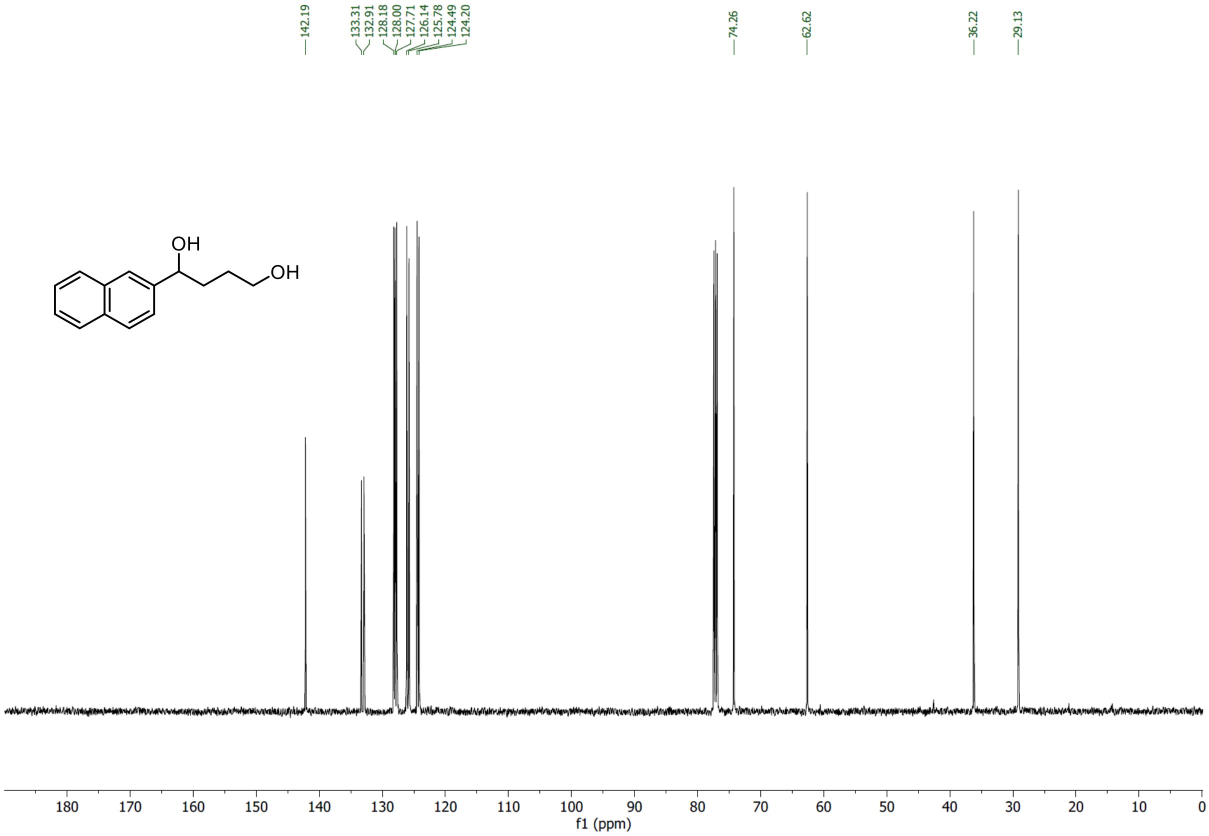

Figure S18. (Top) ^{1}H NMR (500 MHz) and (bottom) $^{13}C\{^{1}H\}$ NMR (126 MHz) spectra of **1h** in $CDCl_3$.

1–(4–fluorophenyl)butane–1,4–diol (1i)

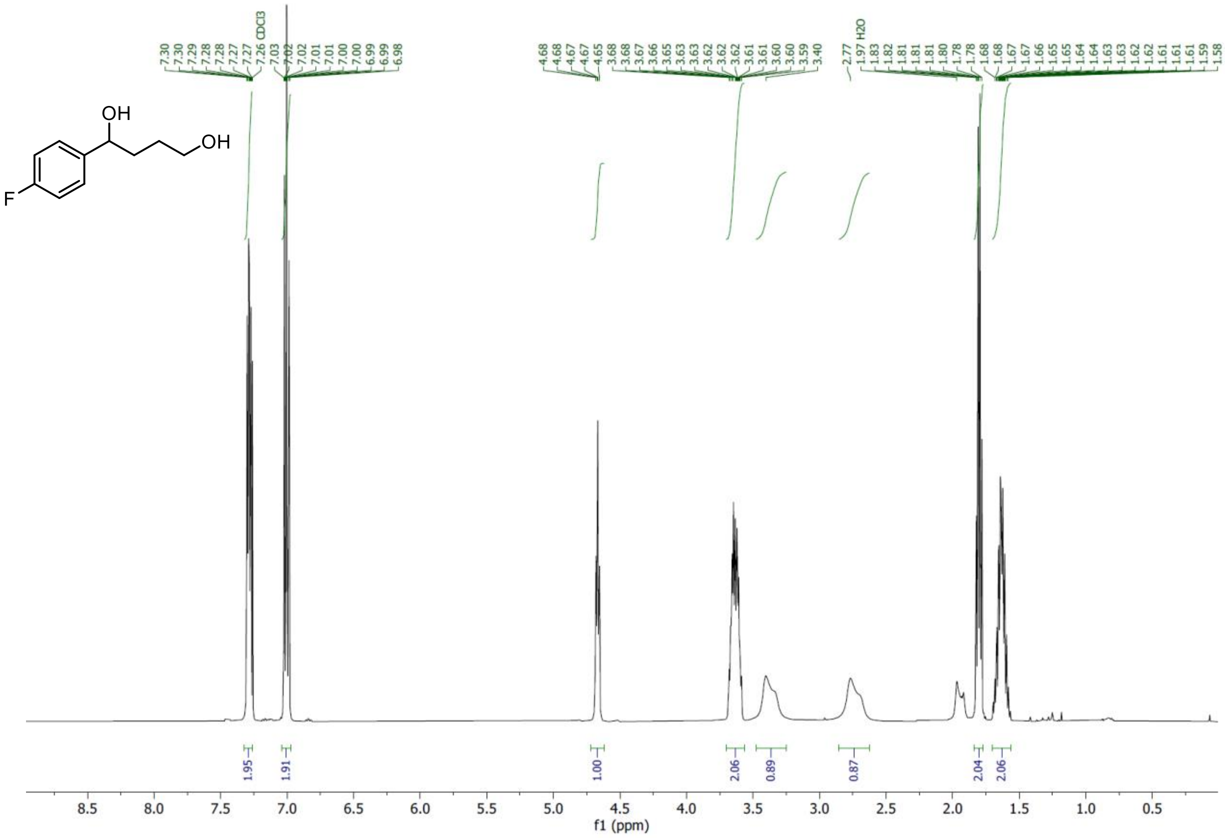

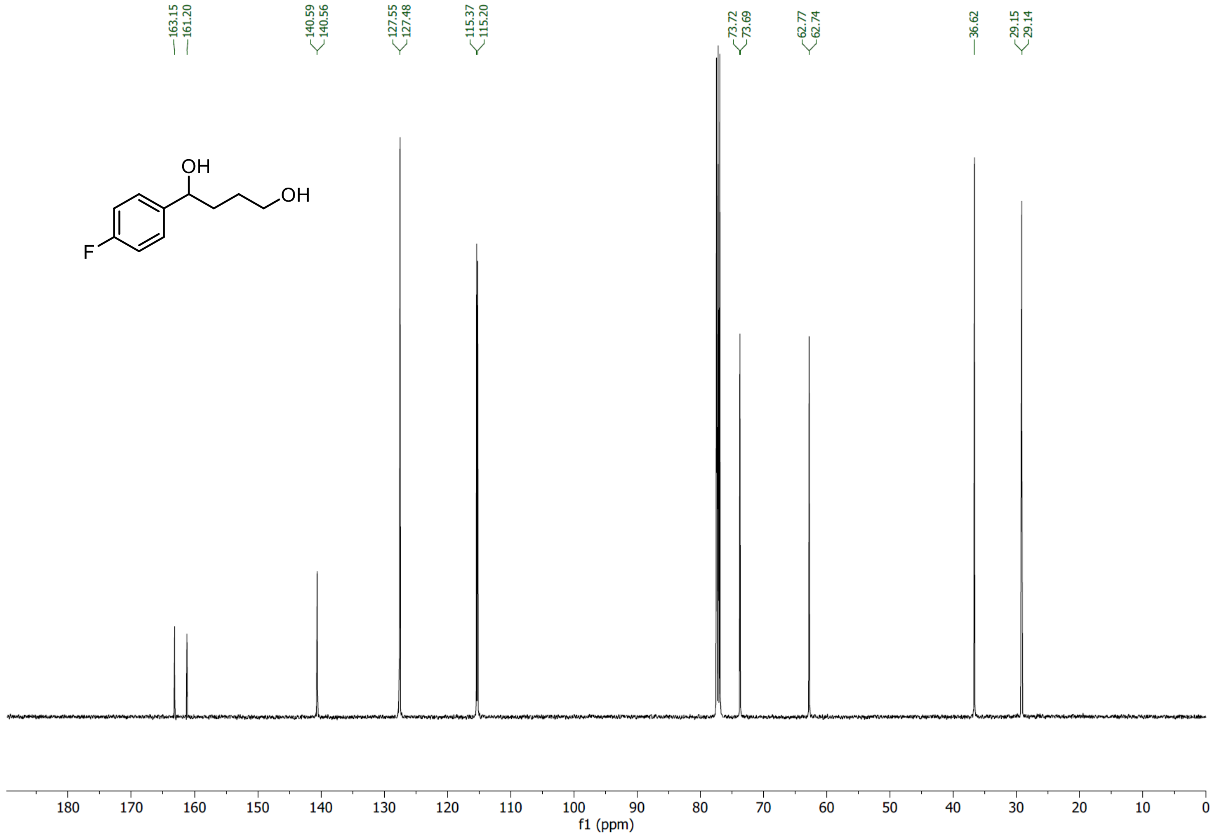

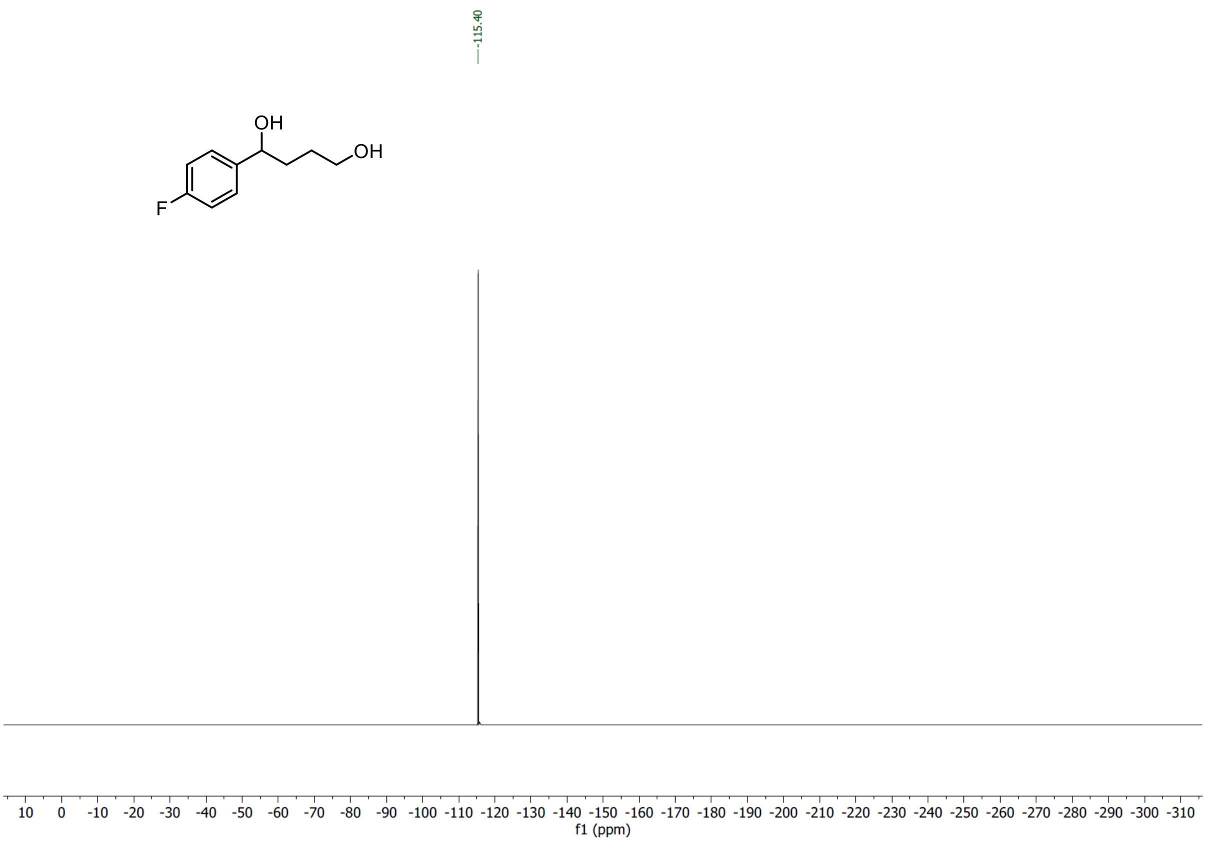

Figure S19. (Top) ^{1}H NMR (500 MHz), (centre) $^{13}C\{^{1}H\}$ NMR (126 MHz) and (bottom) ^{19}F NMR (470 MHz) spectra of **1i** in $CDCl_3$.

1–(4–chlorophenyl)butane–1,4–diol (1j)

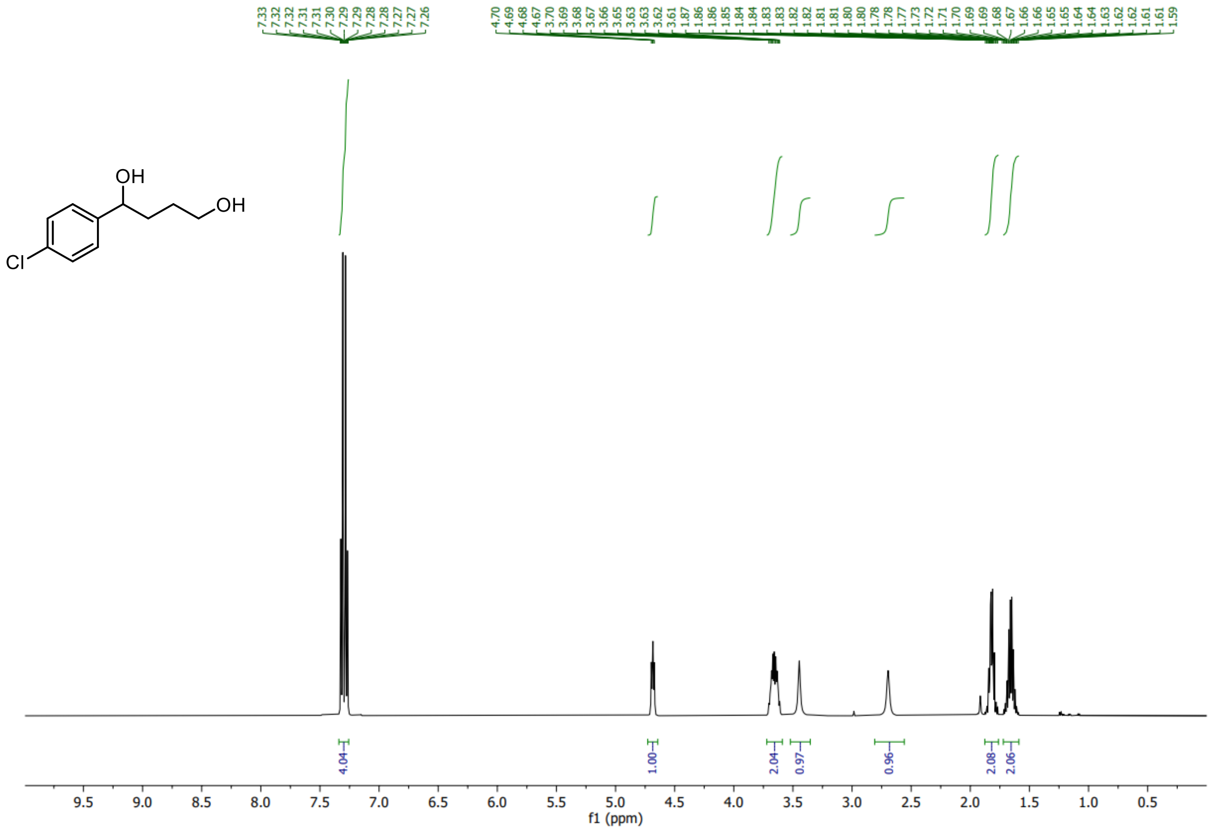

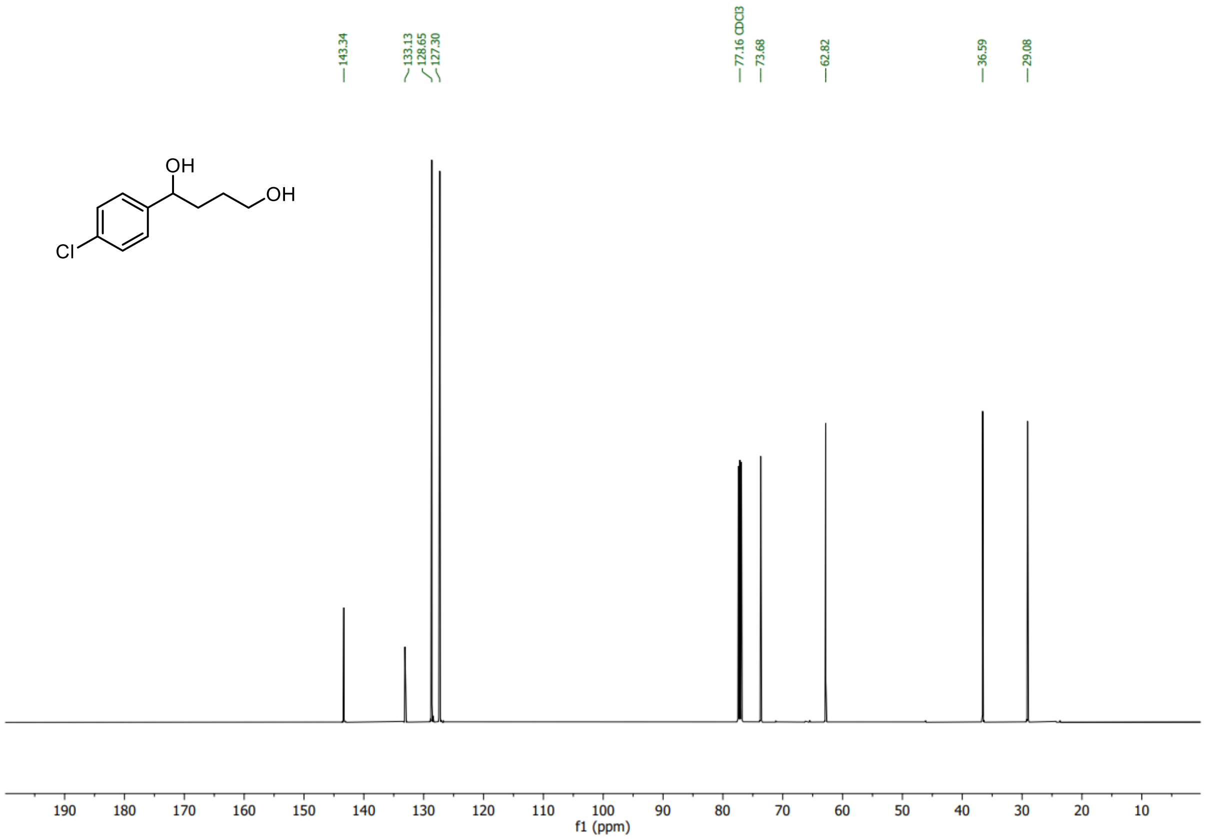

Figure S20. (Top) ^{1}H NMR (500 MHz) and (bottom) $^{13}C\{^{1}H\}$ NMR (126 MHz) spectra of **1j** in $CDCl_3$.

1–(4–bromophenyl)butane–1,4–diol (1k)

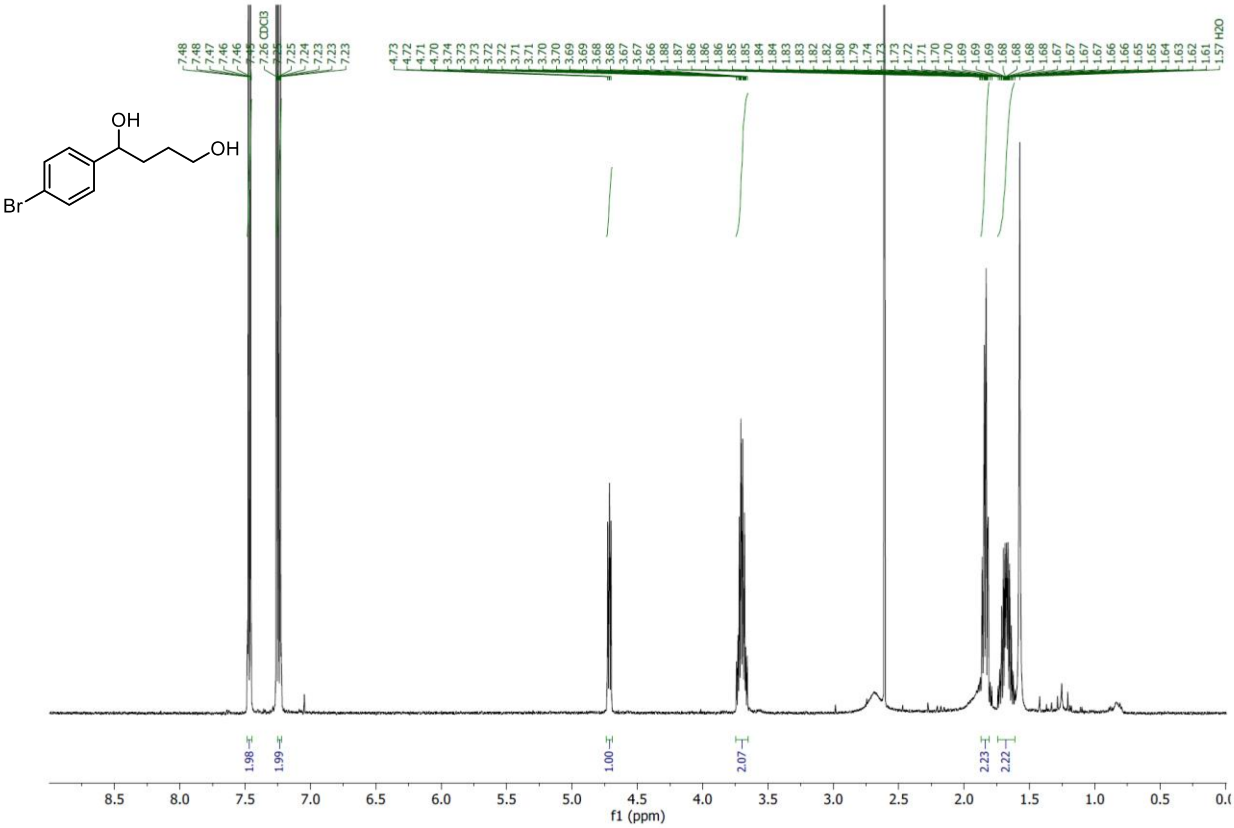

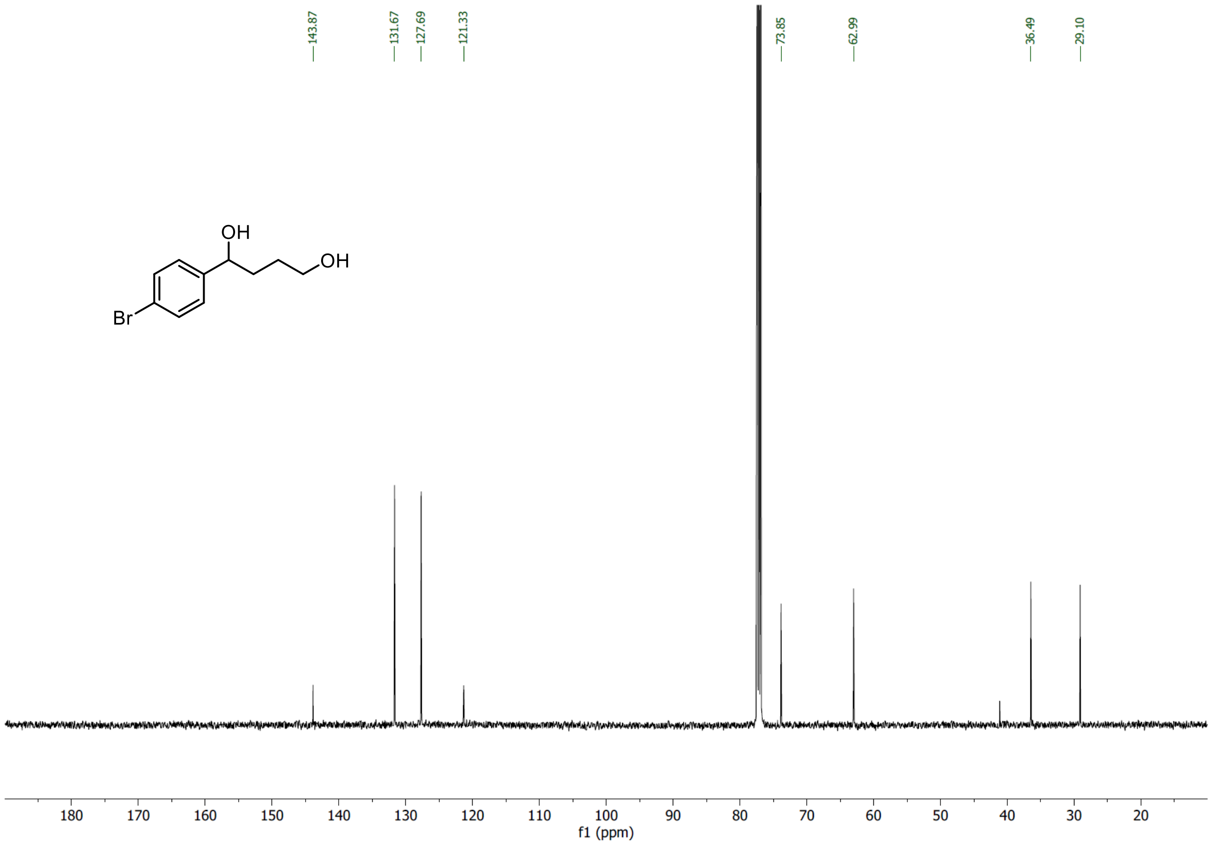

Figure S21. (Top) ^{1}H NMR (500 MHz) and (bottom) $^{13}C\{^{1}H\}$ NMR (126 MHz) spectra of **1k** in $CDCl_3$.

1–(p–tolyl)butane–1,4–diol (1l)

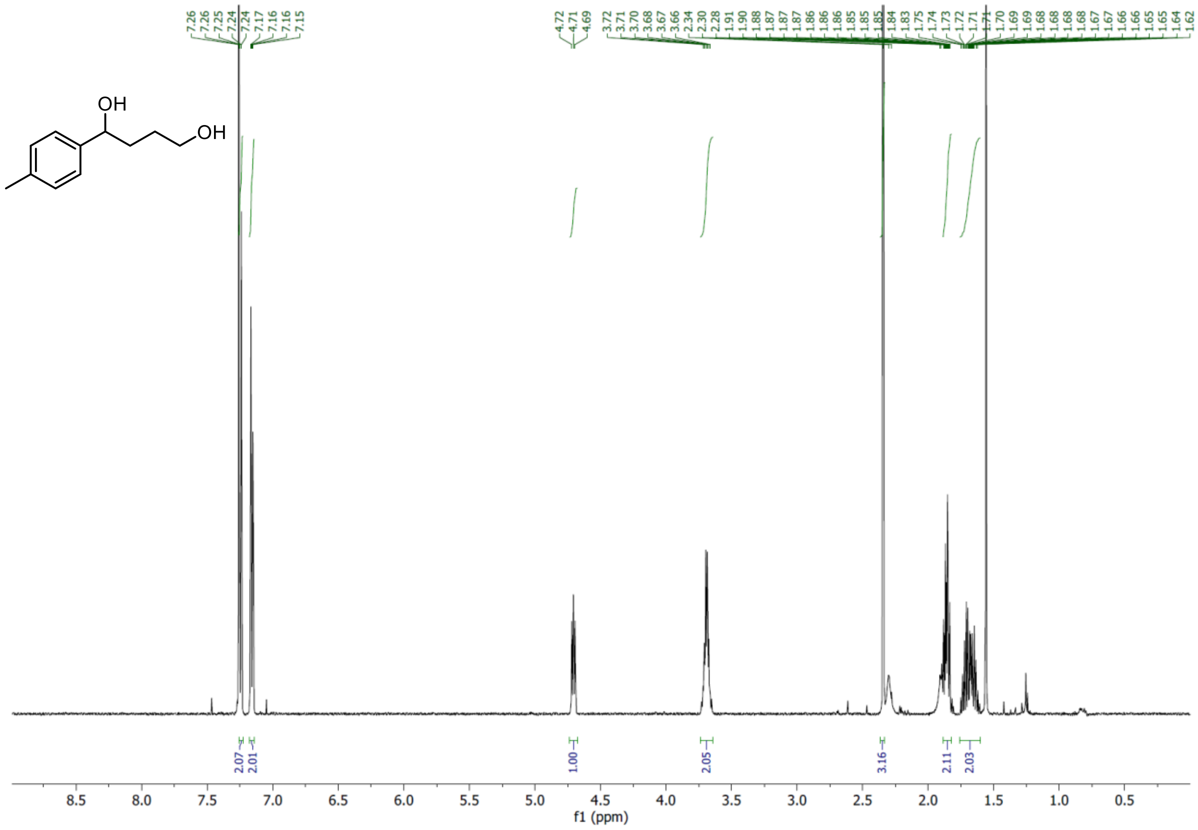

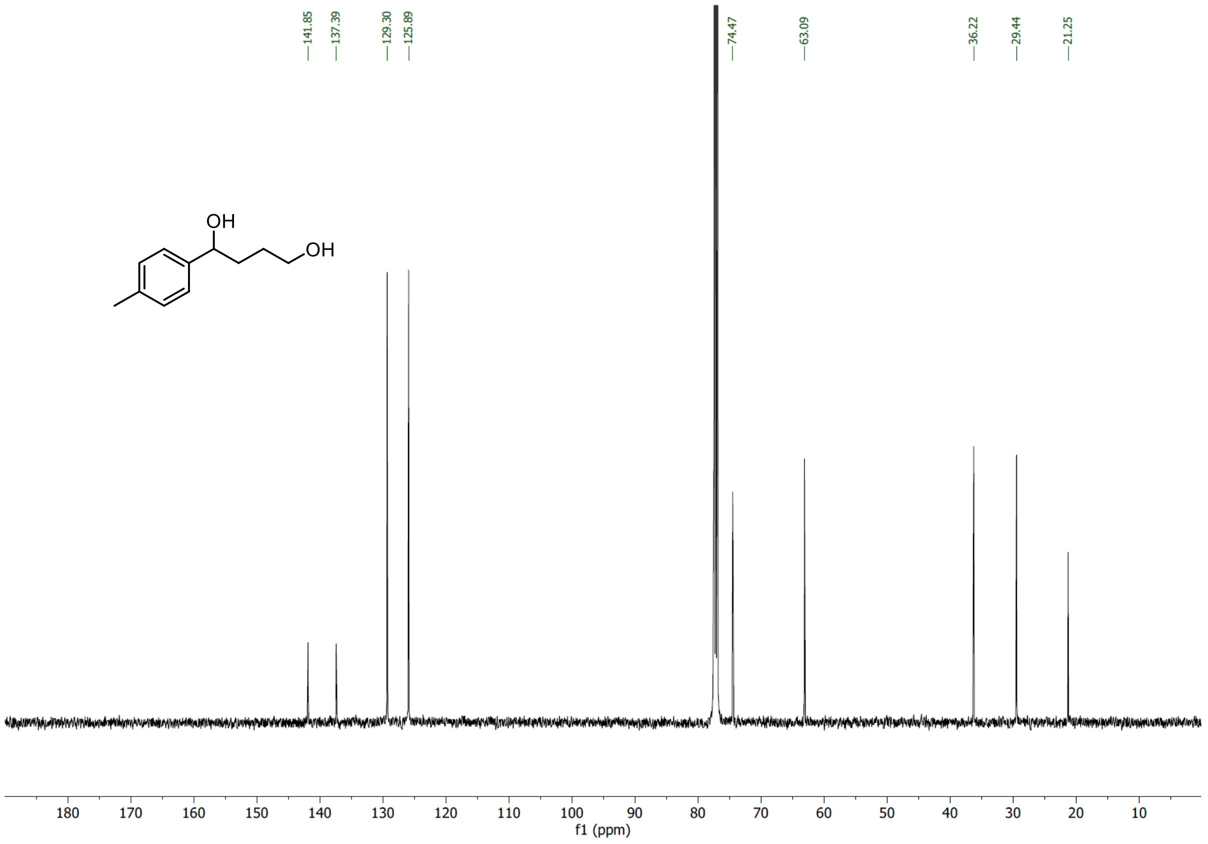

Figure S22. (Top) ^{1}H NMR (500 MHz) and (bottom) $^{13}C\{^{1}H\}$ NMR (126 MHz) spectra of **1l** in $CDCl_3$.

1–(4–(trifluoromethyl)phenyl)butane–1,4–diol (1m)

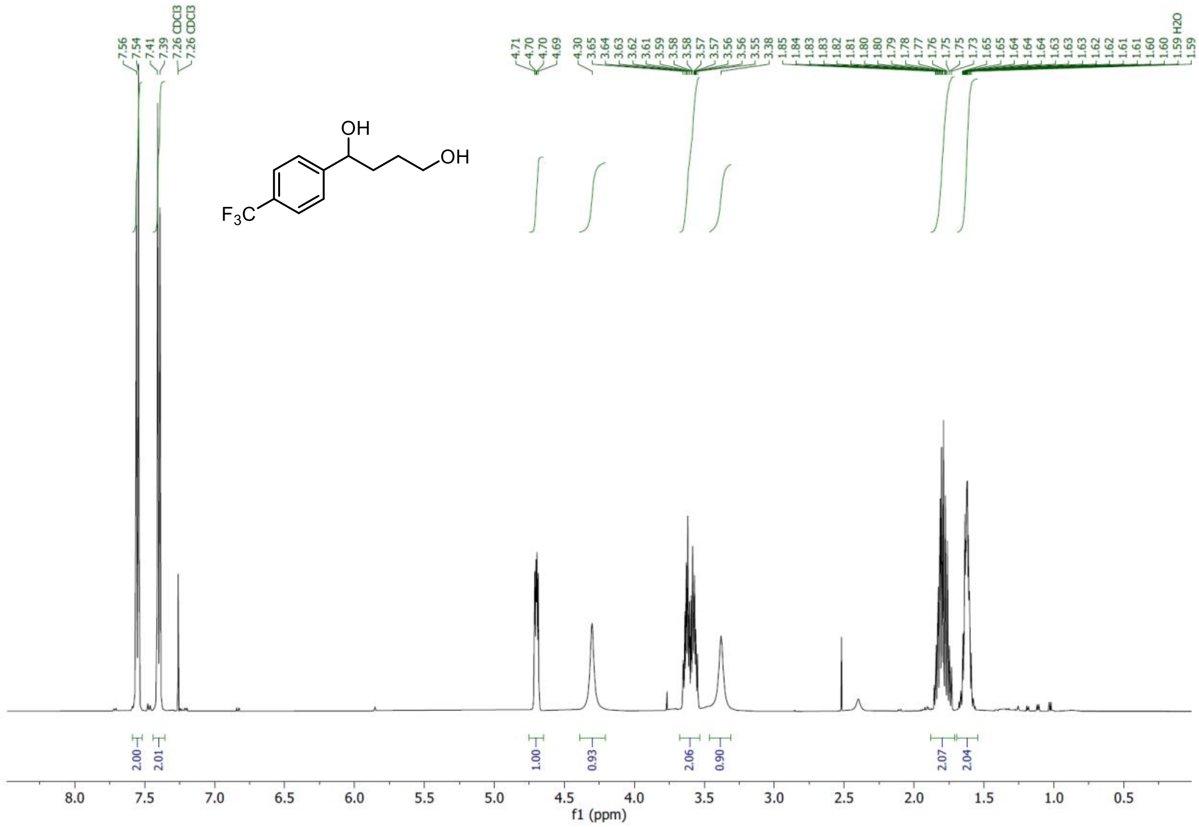

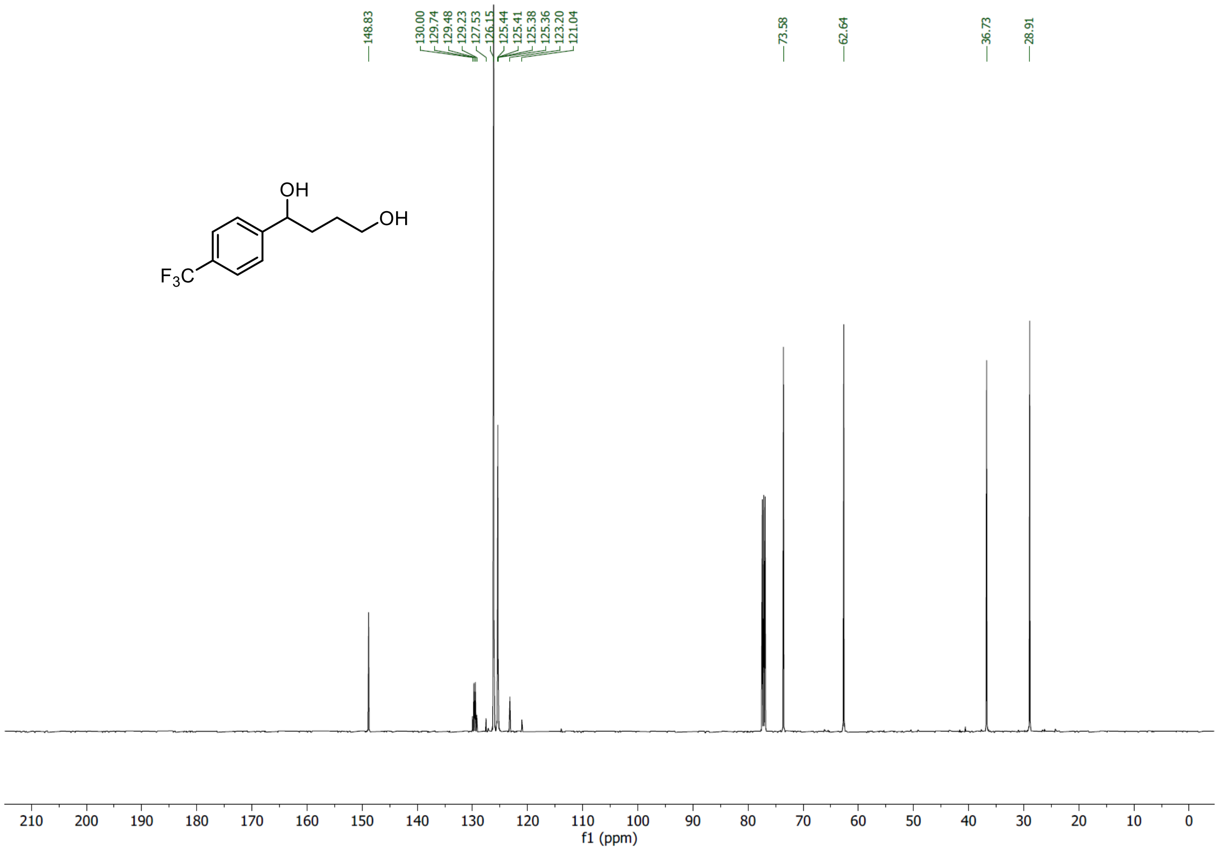

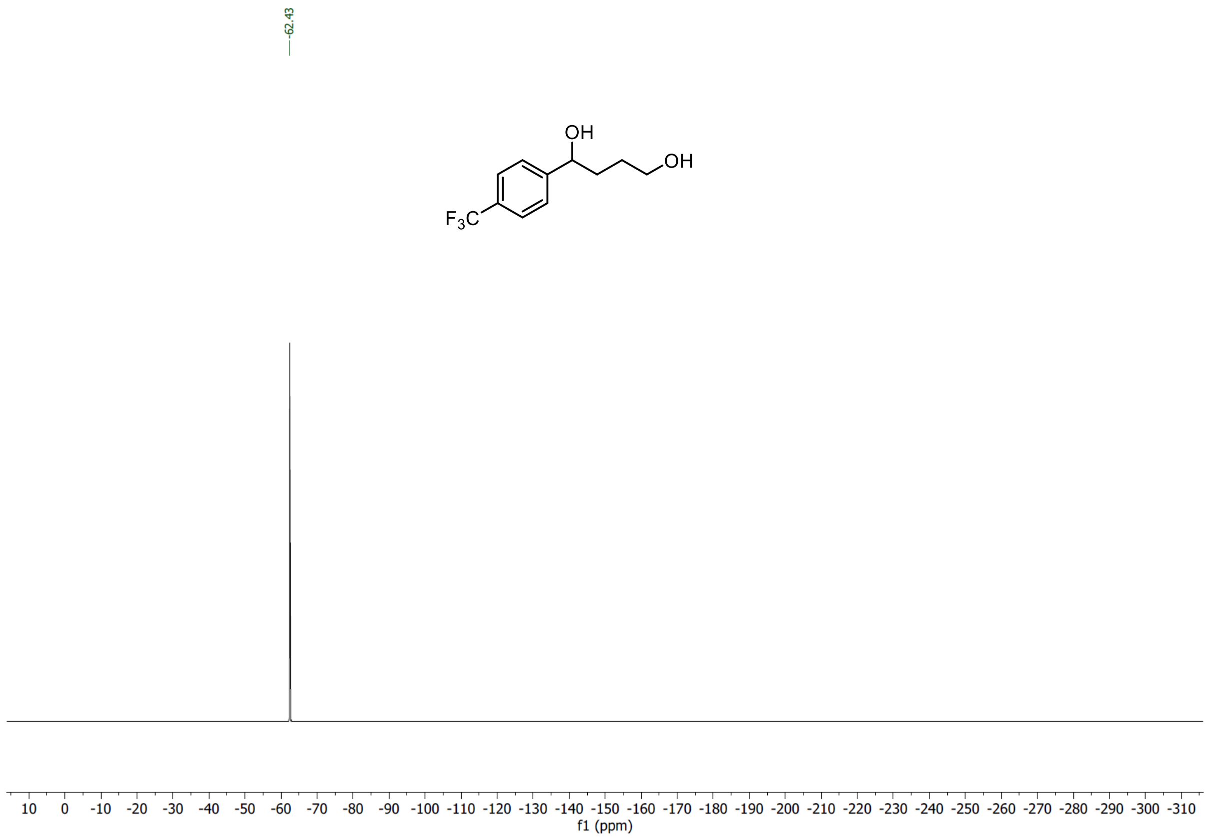

Figure S23. (Top) ^{1}H NMR (500 MHz), (centre) $^{13}C\{^{1}H\}$ NMR (126 MHz) and (bottom) ^{19}F NMR (470 MHz) spectra of **1m** in $CDCl_3$.

1-(4-ethylphenyl)butane-1,4-diol (1n)

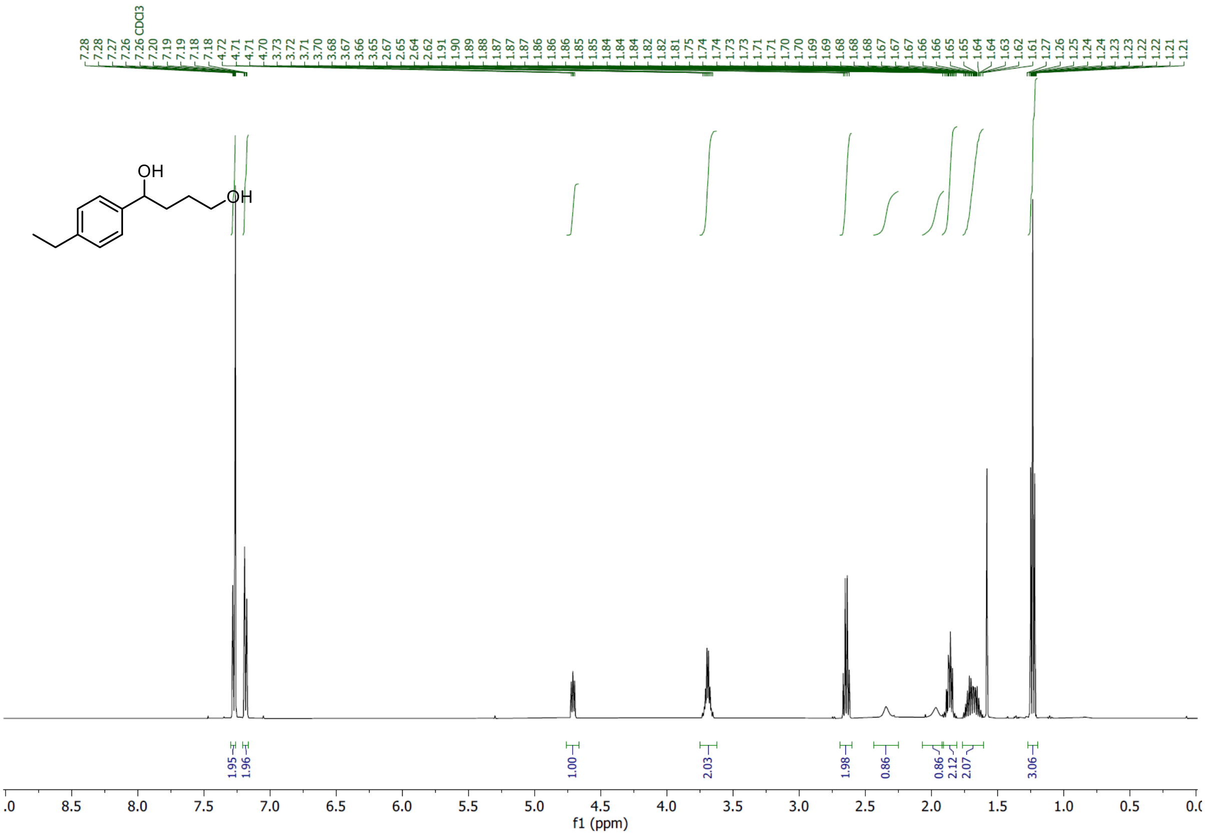

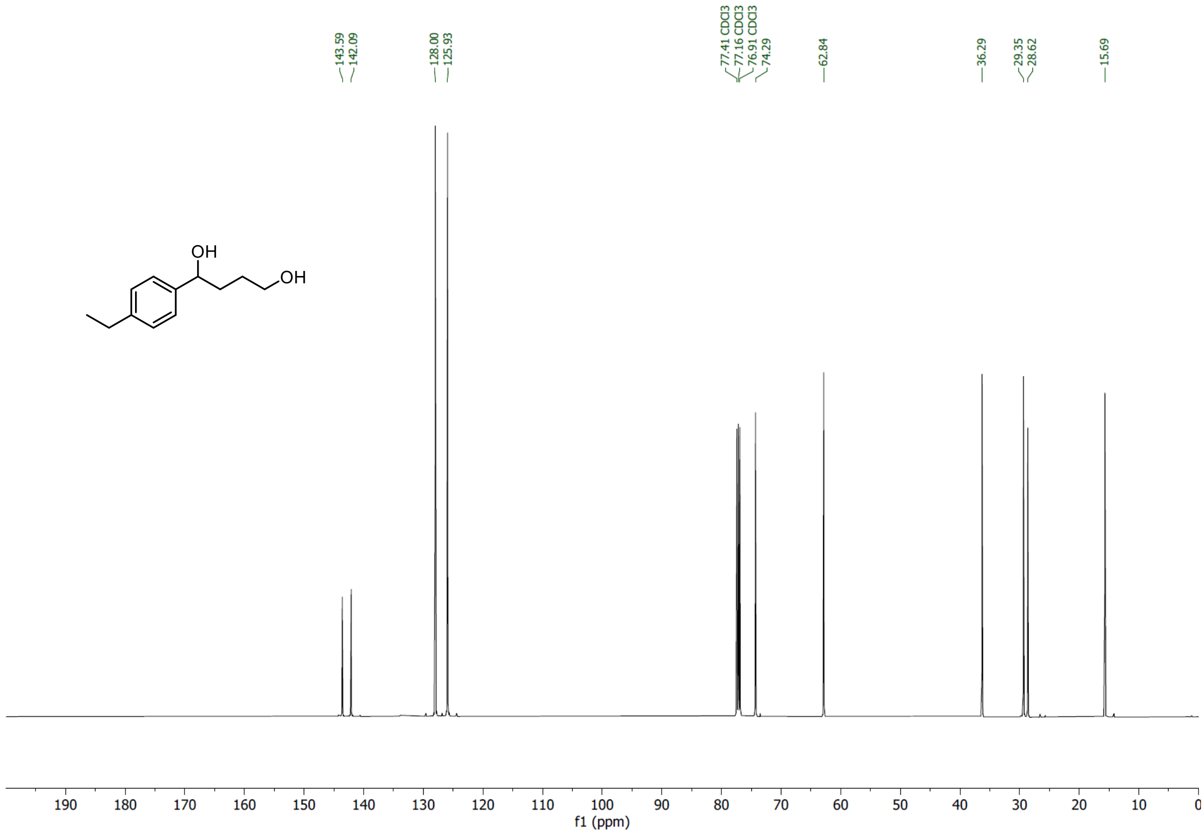

Figure S24. (Top) ^{1}H NMR (500 MHz) and (bottom) ^{13}C{^{1}H} NMR (126 MHz) spectra of **1n** in $CDCl_3$.

(6-chloropyridin-3-yl)butane-1,4-diol (1o)

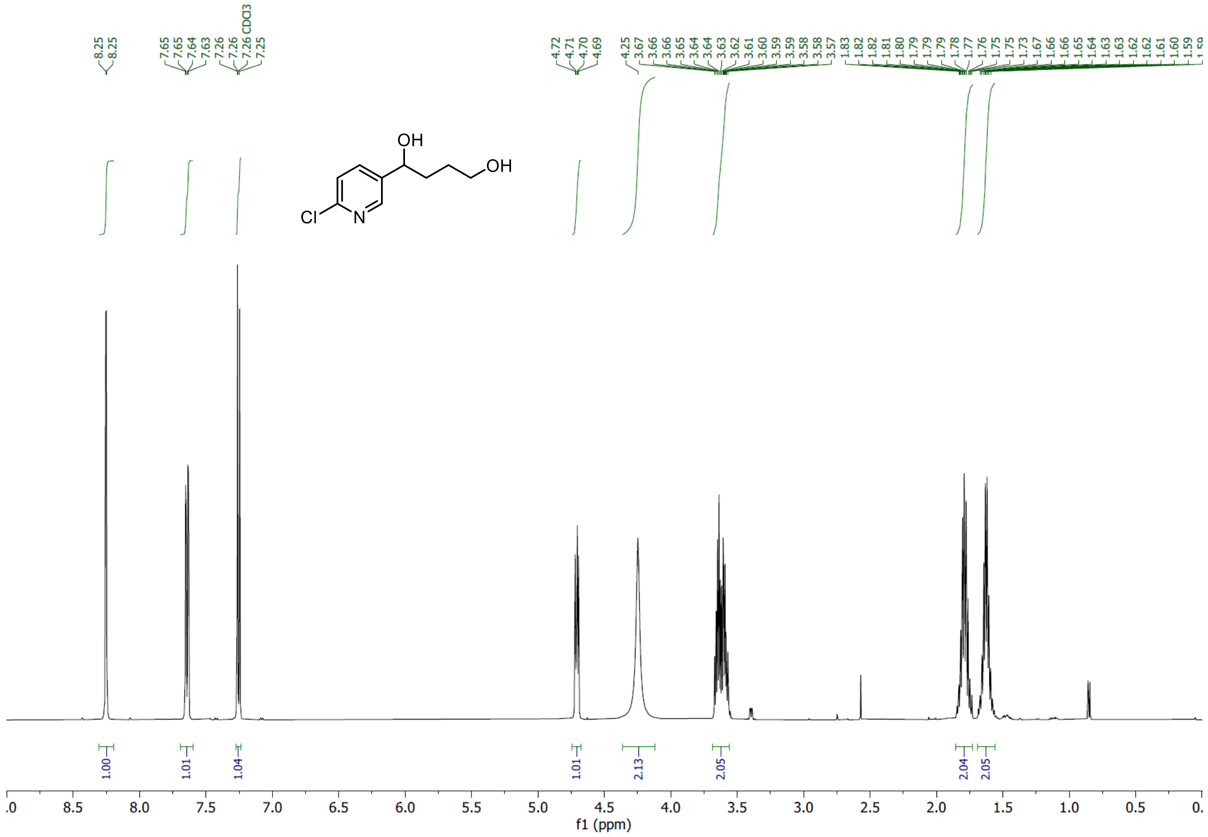

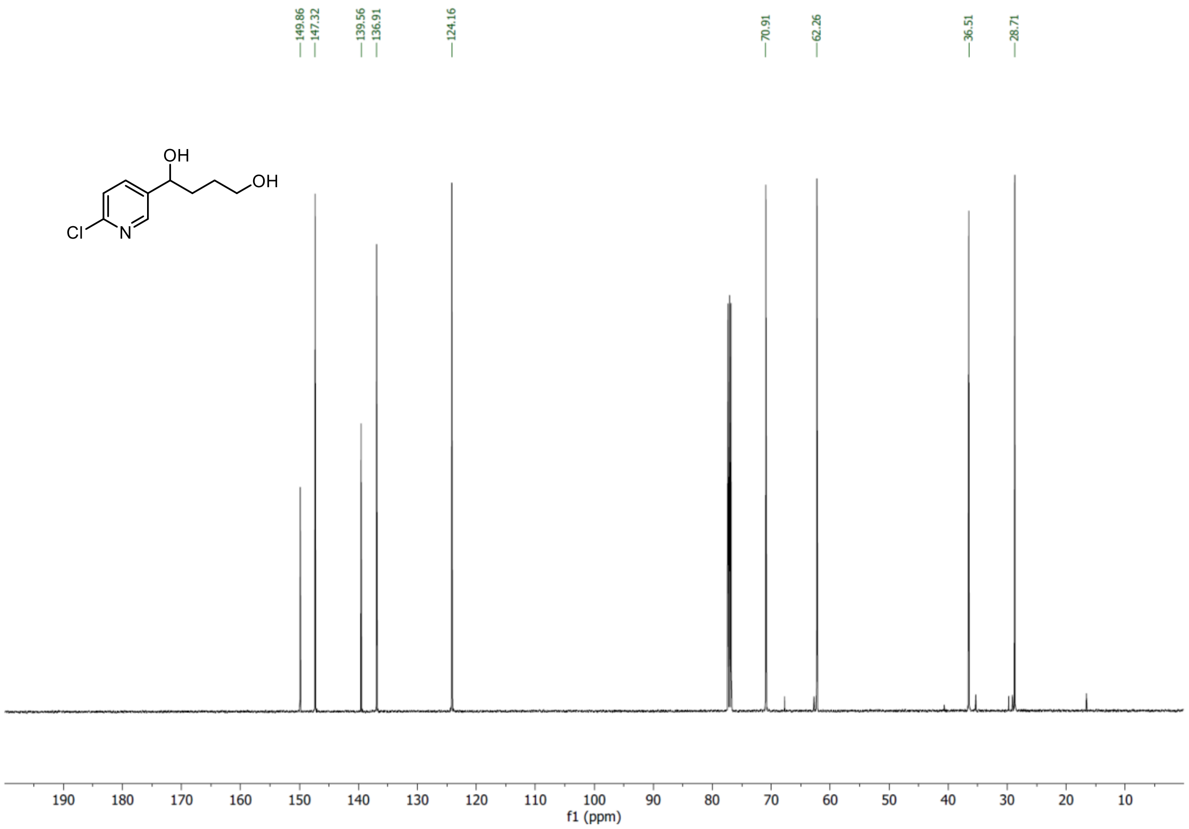

Figure S25. (Top) ^{1}H NMR (500 MHz) and (bottom) $^{13}C\{^{1}H\}$ NMR (126 MHz) spectra of **1o** in $CDCl_3$.

4-phenylpentane-1,4-diol (1p)

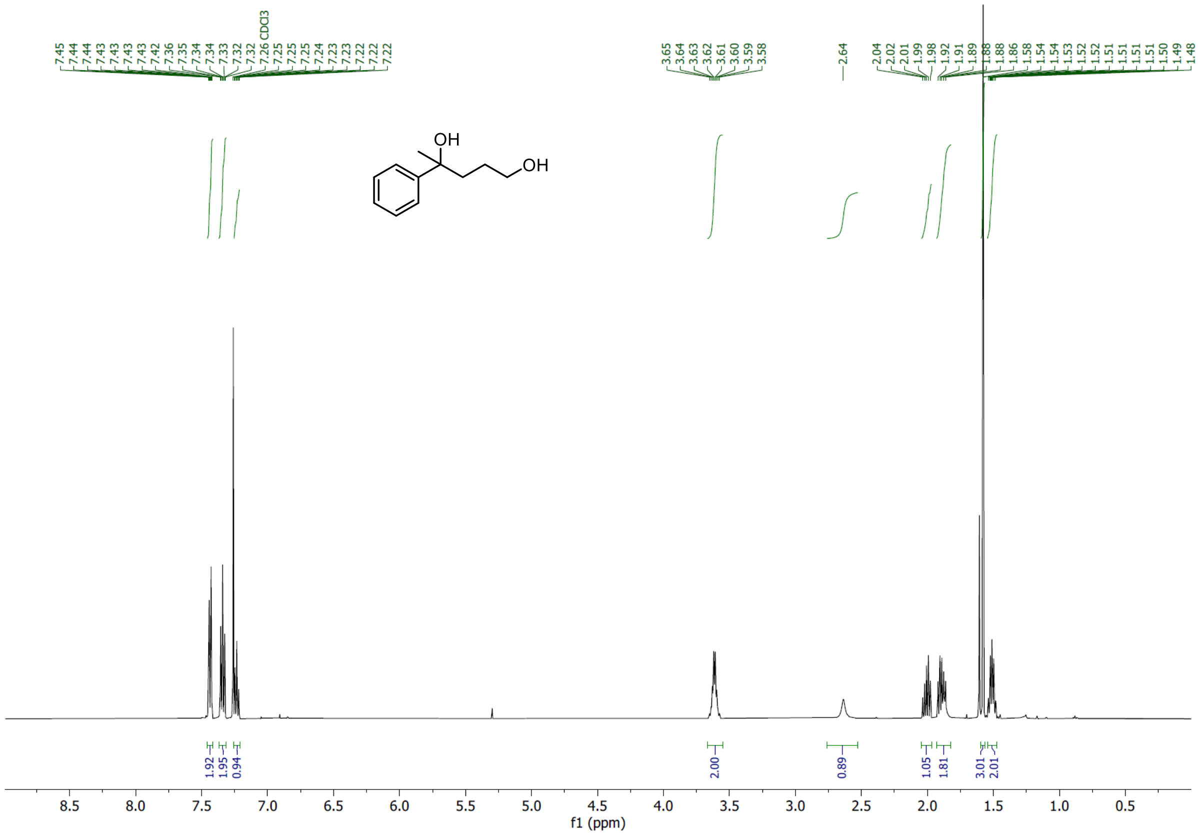

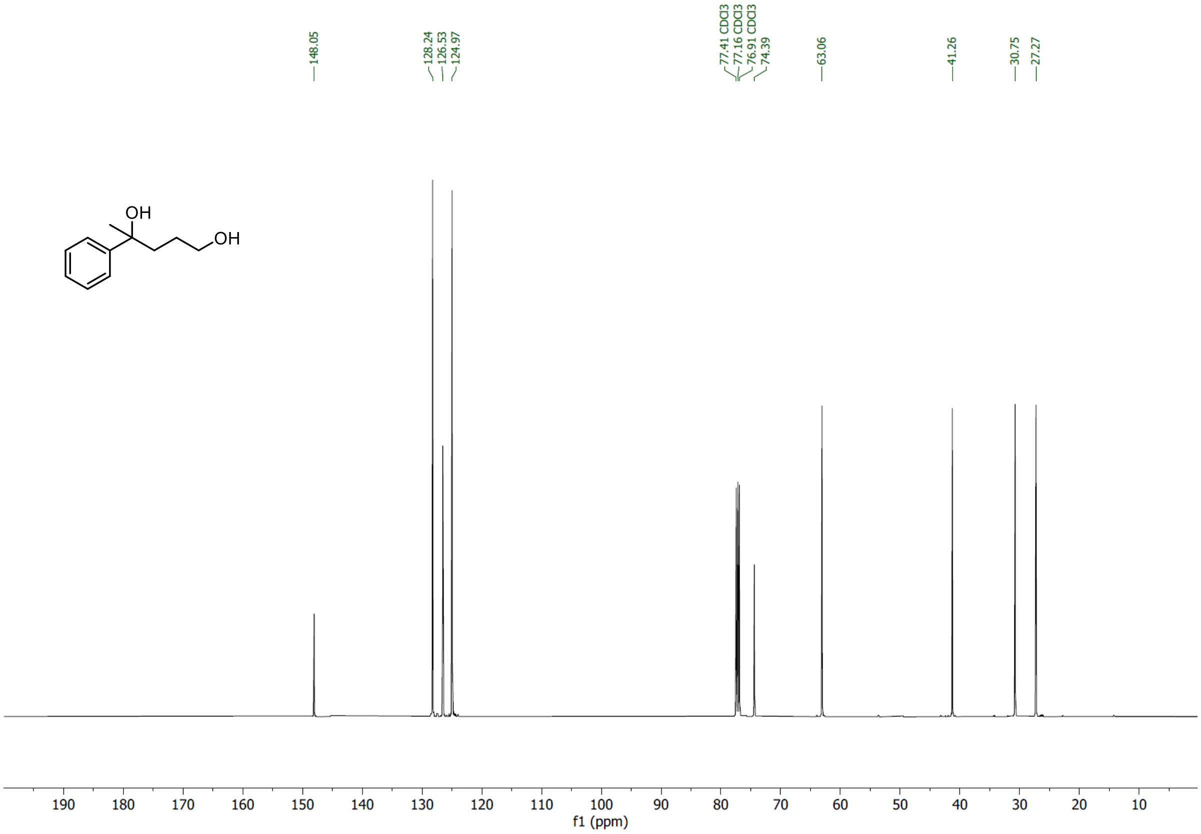

Figure S26. (Top) ^{1}H NMR (500 MHz) and (bottom) ^{13}C{^{1}H} NMR (126 MHz) spectra of **1p** in $CDCl_3$.

1-cyclopentylbutane-1,4-diol (1q)

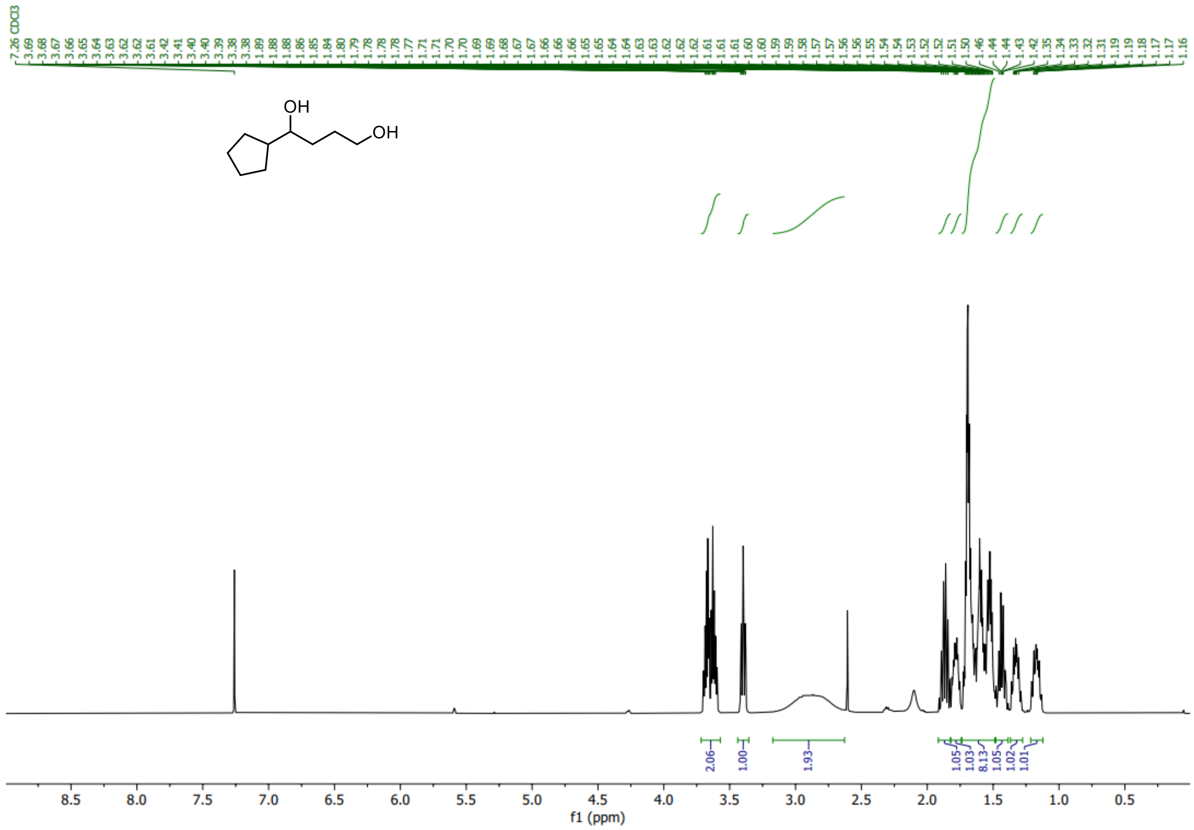

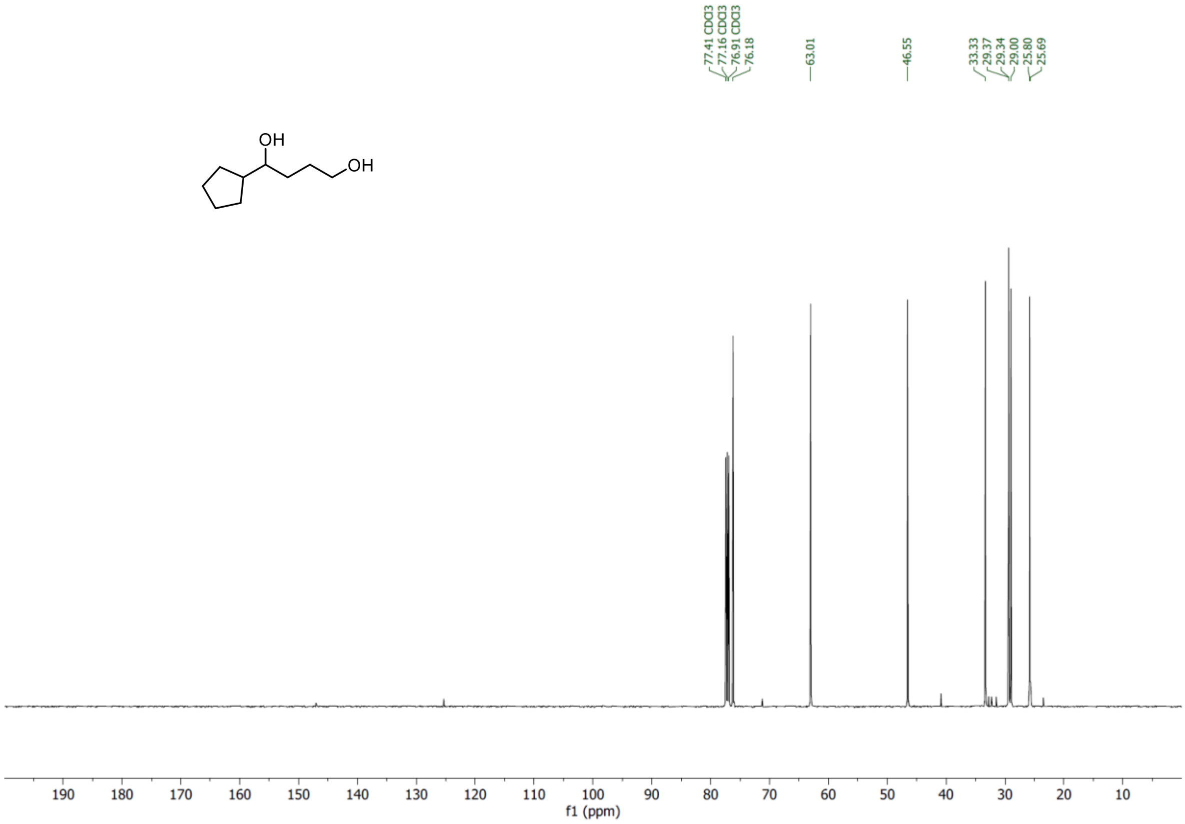

Figure S27. (Top) ^{1}H NMR (500 MHz) and (bottom) $^{13}C\{^{1}H\}$ NMR (126 MHz) spectra of **1q** in $CDCl_3$.

1-phenylpentane-1,5-diol (1s)

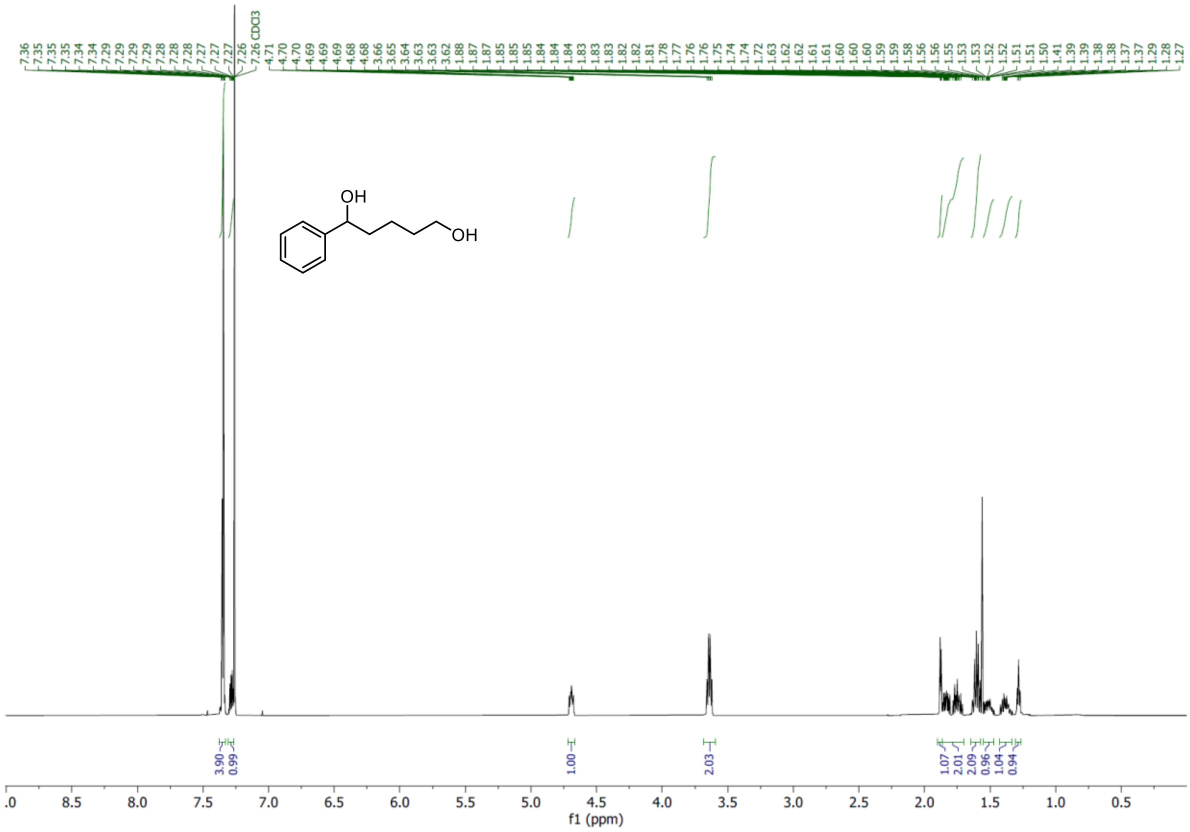

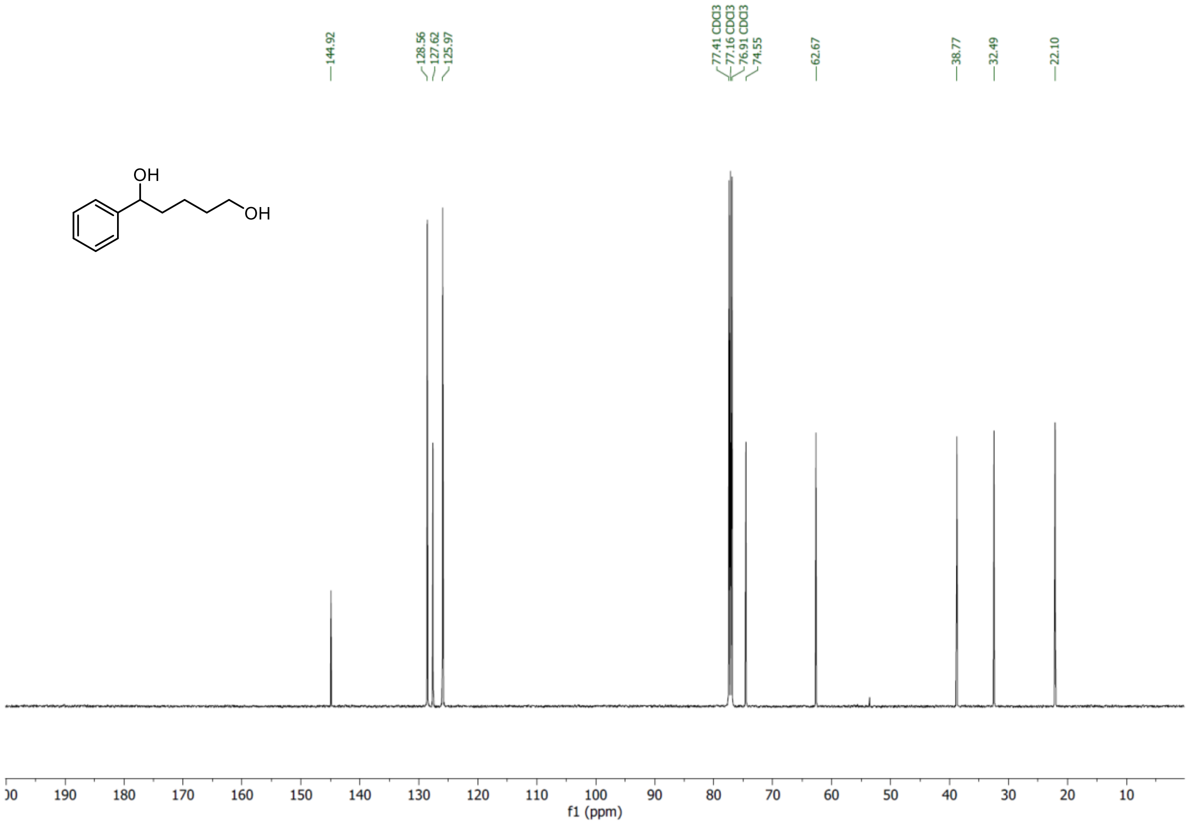

Figure S28. (Top) ^{1}H NMR (500 MHz) and (bottom) $^{13}C\{^{1}H\}$ NMR (126 MHz) spectra of **1s** in $CDCl_3$.

pentadecane-1,4-diol (1u)

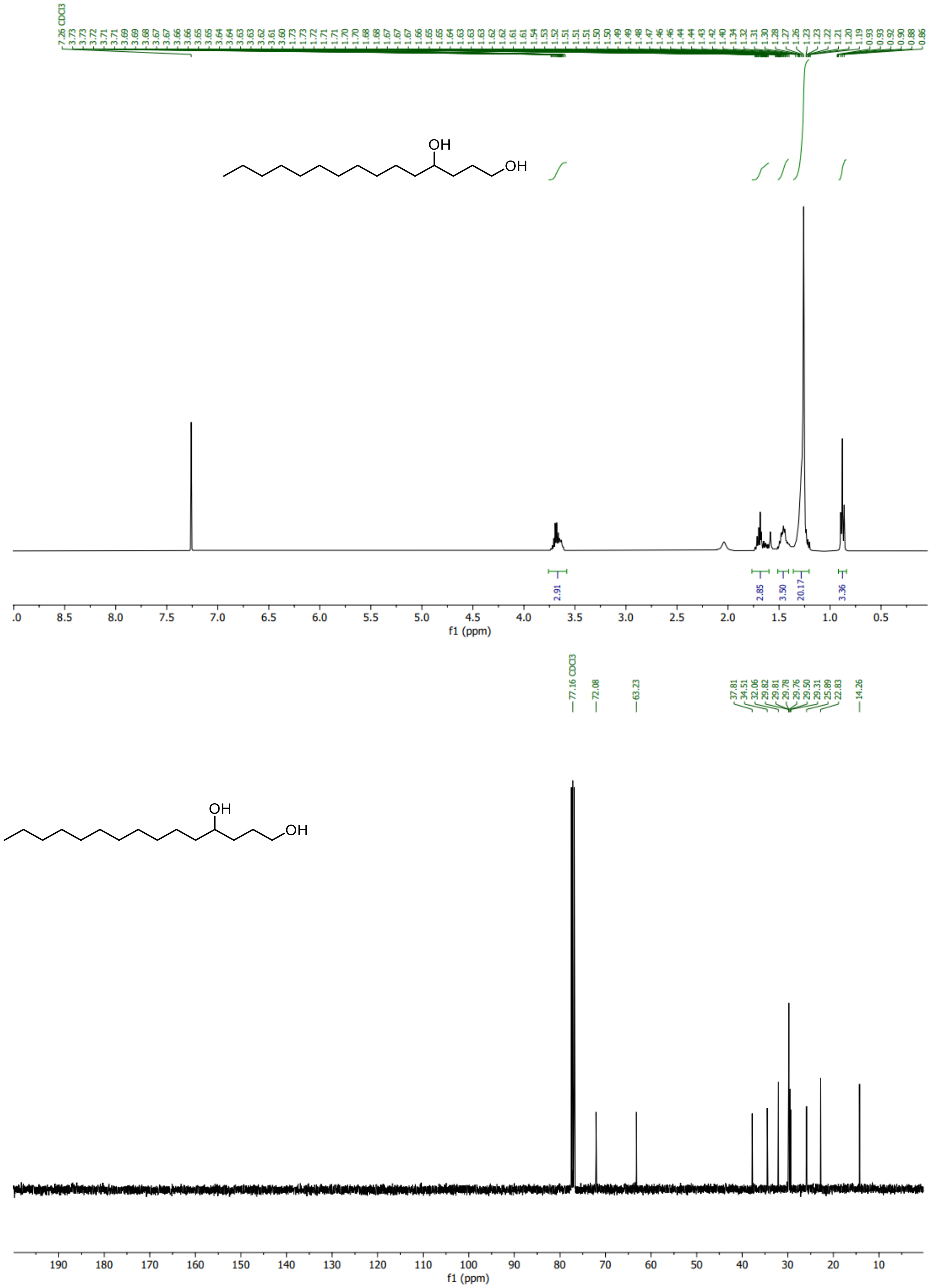

Figure S29. (Top) ^{1}H NMR (400 MHz) and (bottom) $^{13}C\{^{1}H\}$ NMR (101 MHz) spectra of **1u** in $CDCl_3$.

(*S*)-1–phenylbutane–1,4–diol [(*S*)-1a]

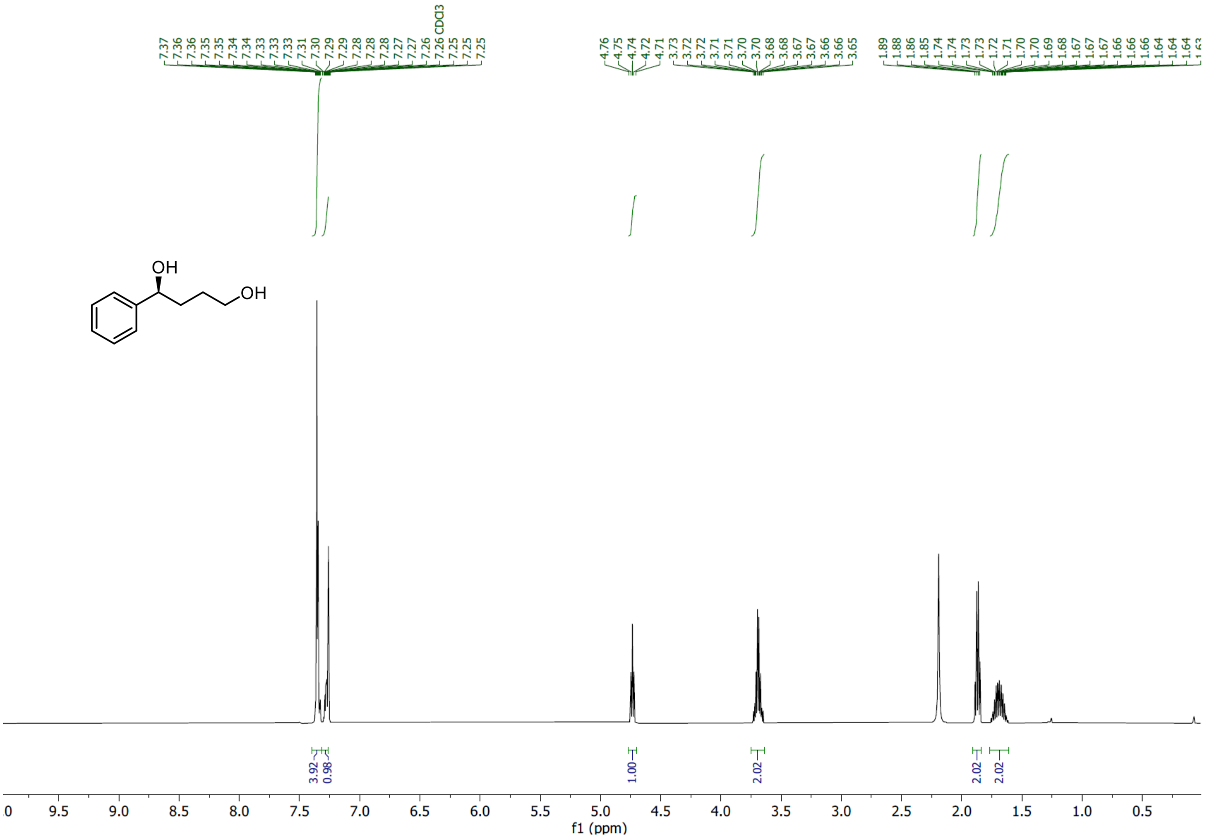

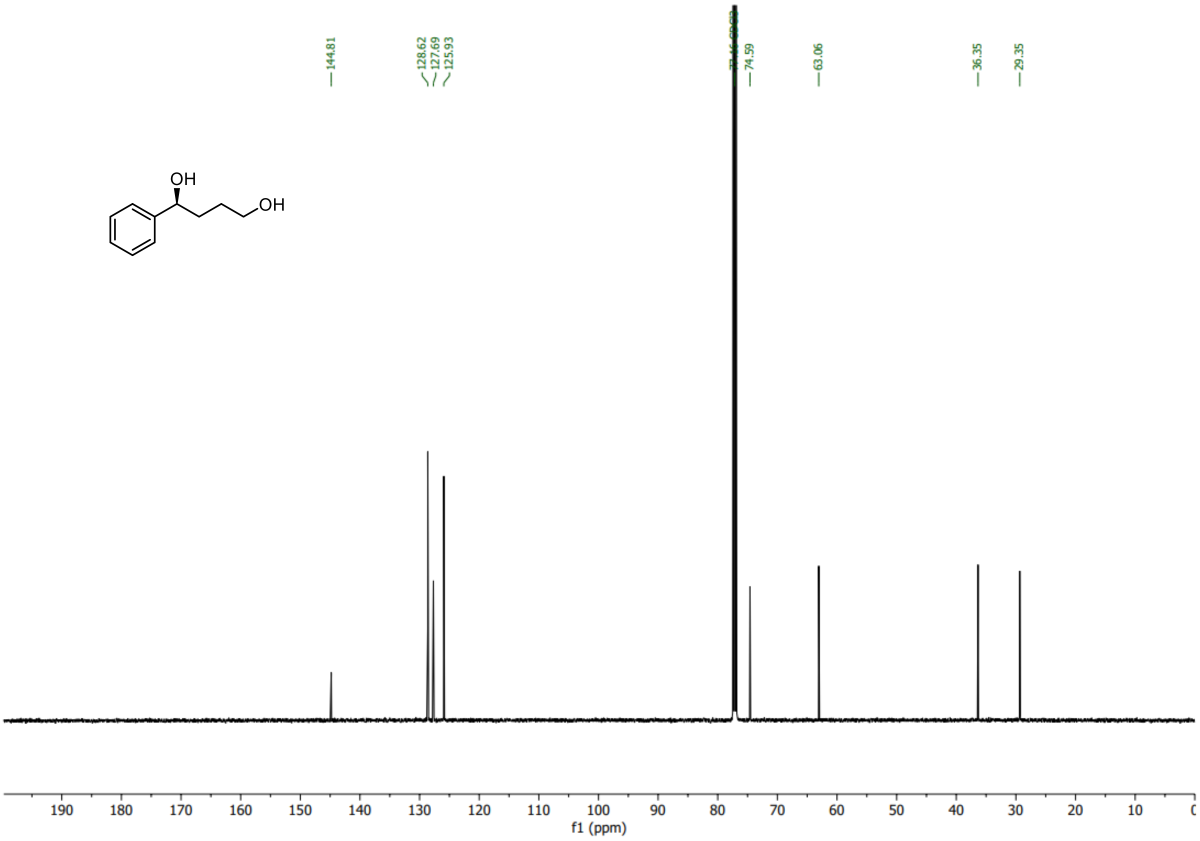

Figure S30. (Top) ^{1}H NMR (500 MHz) and (bottom) ^{13}C{^{1}H} NMR (126 MHz) spectra of **(*S*)-1a** in $CDCl_3$.

(*S*)-1-(2-chlorophenyl)butane-1,4-diol [(*S*)-1b]

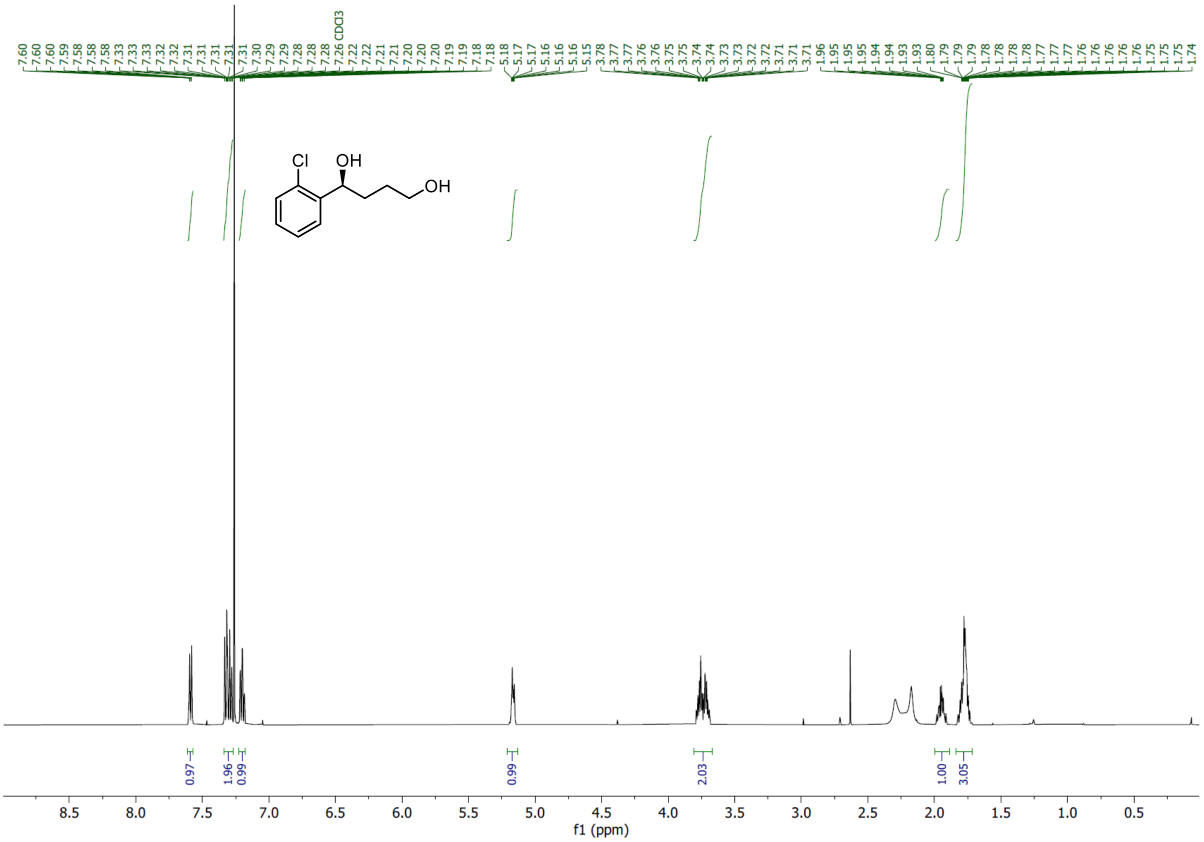

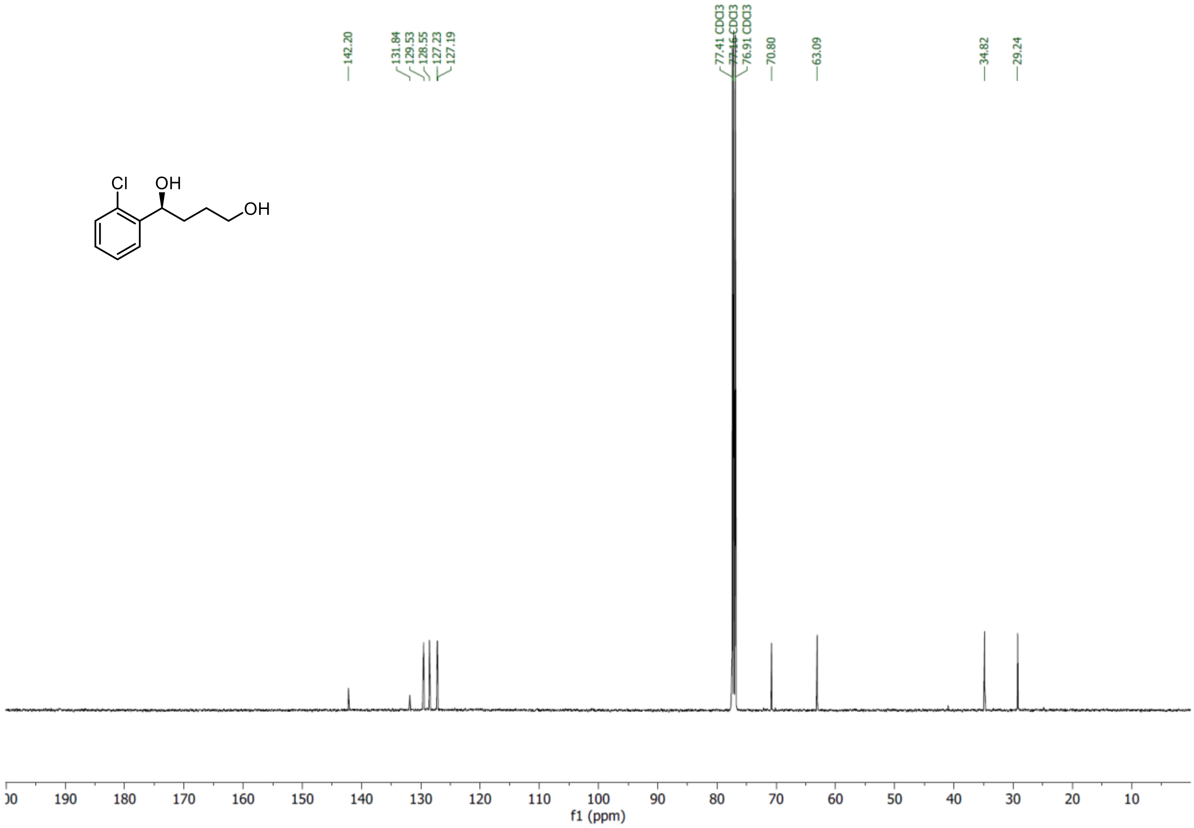

Figure S31. (Top) ^{1}H NMR (500 MHz) and (bottom) $^{13}C\{^{1}H\}$ NMR (126 MHz) spectra of **(*S*)-1b** in $CDCl_3$.

(*S*)-1-(m-tolyl)butane-1,4-diol [(*S*)-1g]

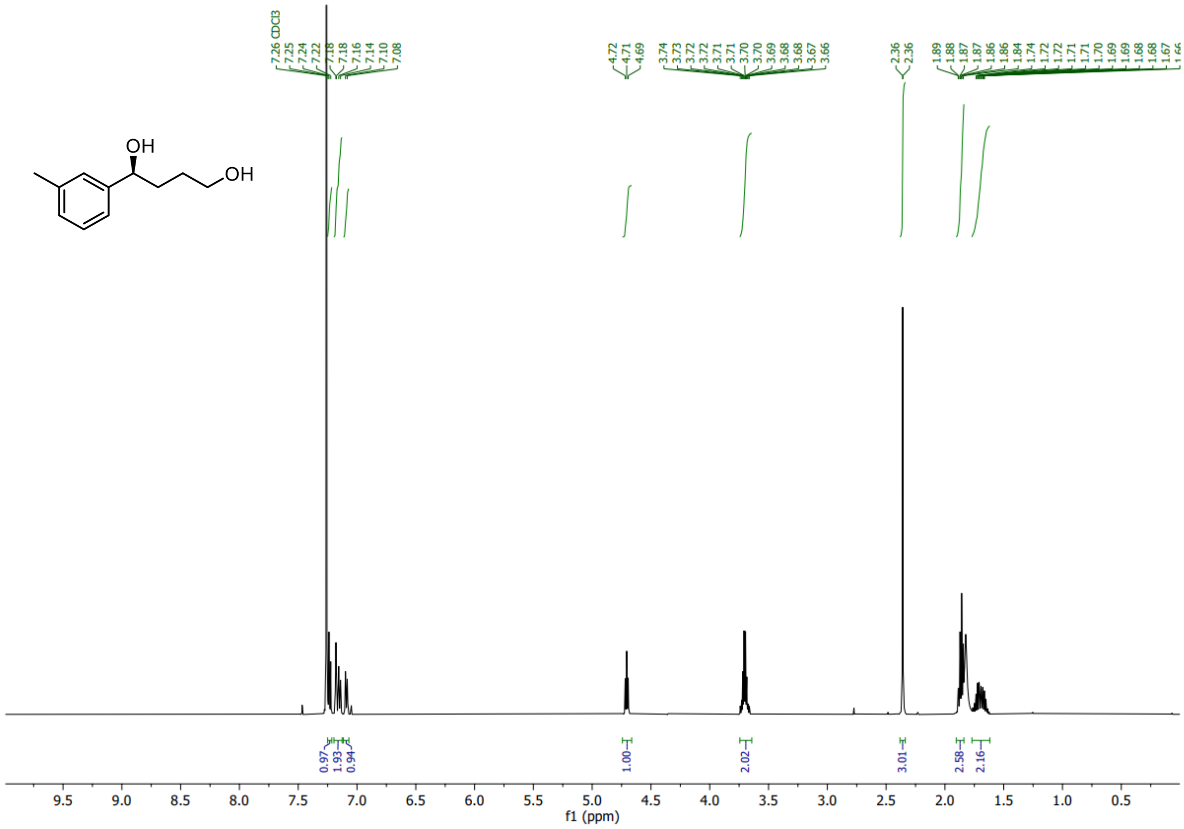

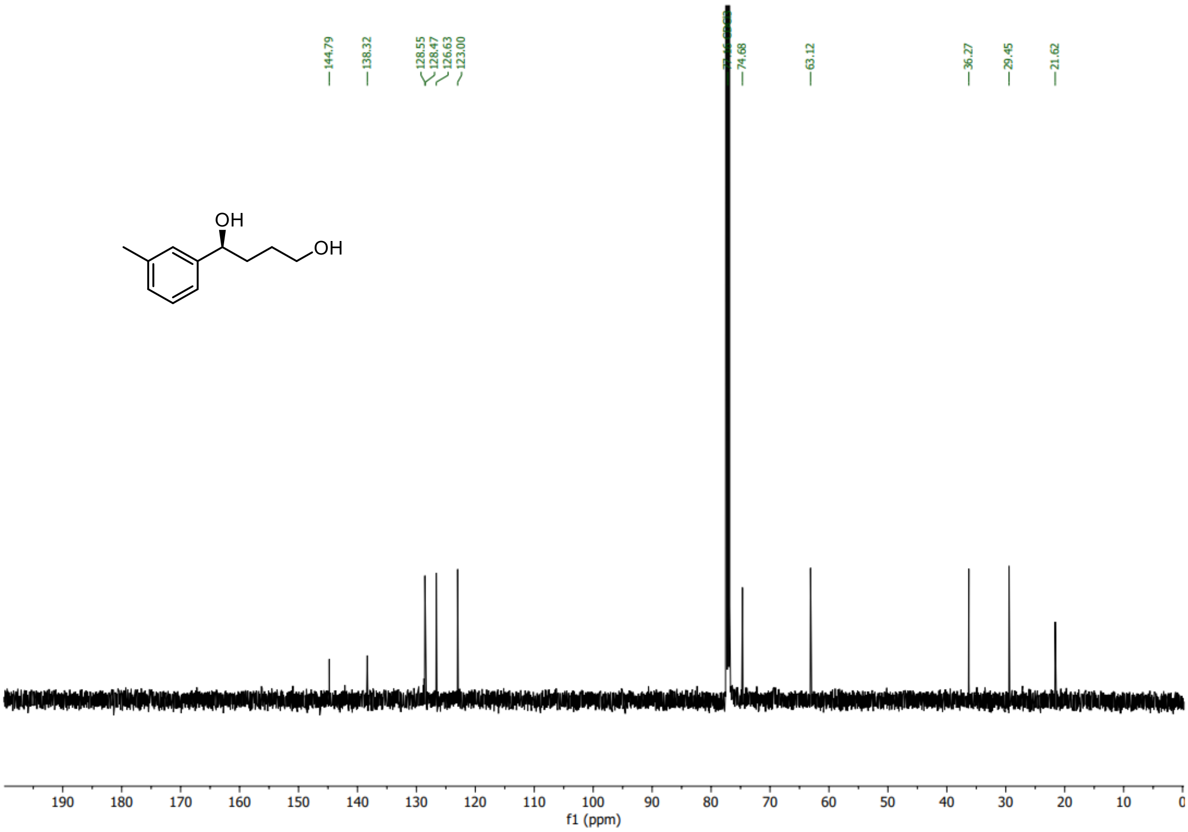

Figure S32. (Top) ^{1}H NMR (500 MHz) and (bottom) ^{13}C{^{1}H} NMR (126 MHz) spectra of **(*S*)-1g** in $CDCl_3$.

(*S*)-1-(4-fluorophenyl)butane-1,4-diol [(*S*)-1i]

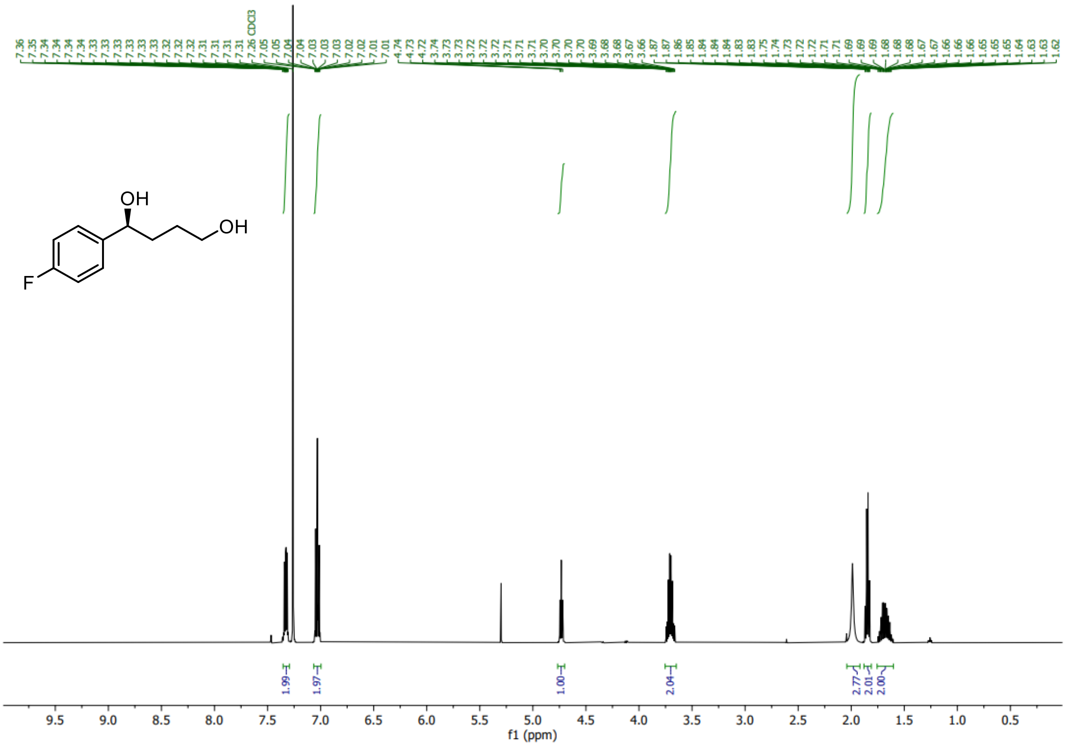

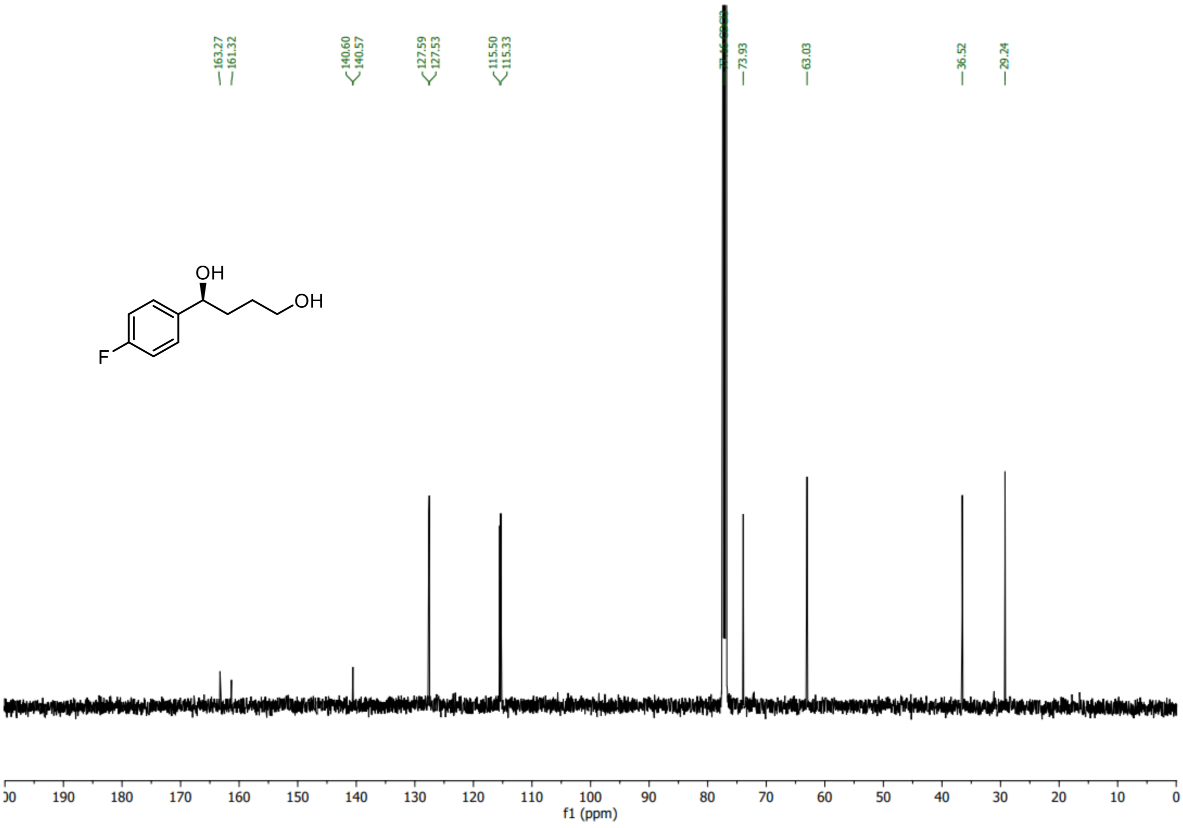

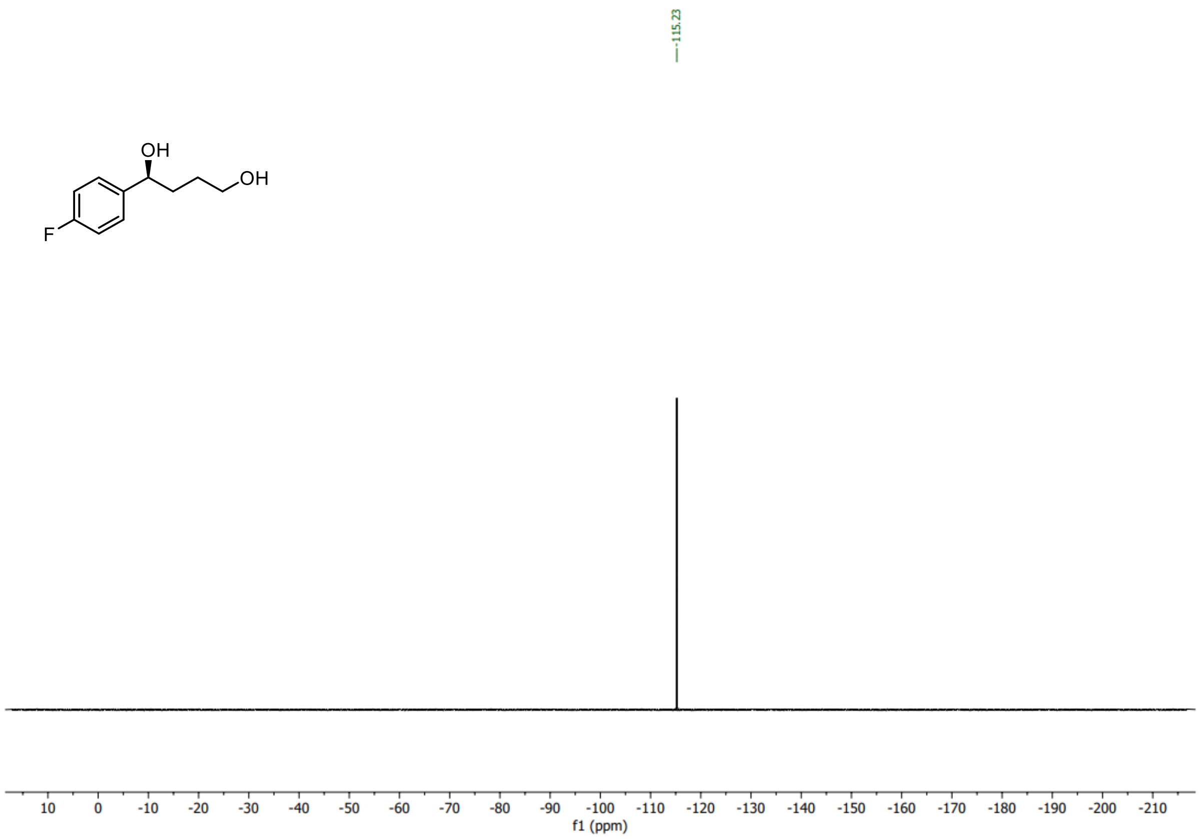

Figure S33. (Top) ^{1}H NMR (500 MHz) and (bottom) $^{13}C\{^{1}H\}$ NMR (126 MHz) spectra of ***(S)*-1i** in $CDCl_3$.

(*S*)-1-(4-chlorophenyl)butane-1,4-diol [(*S*)-1j]

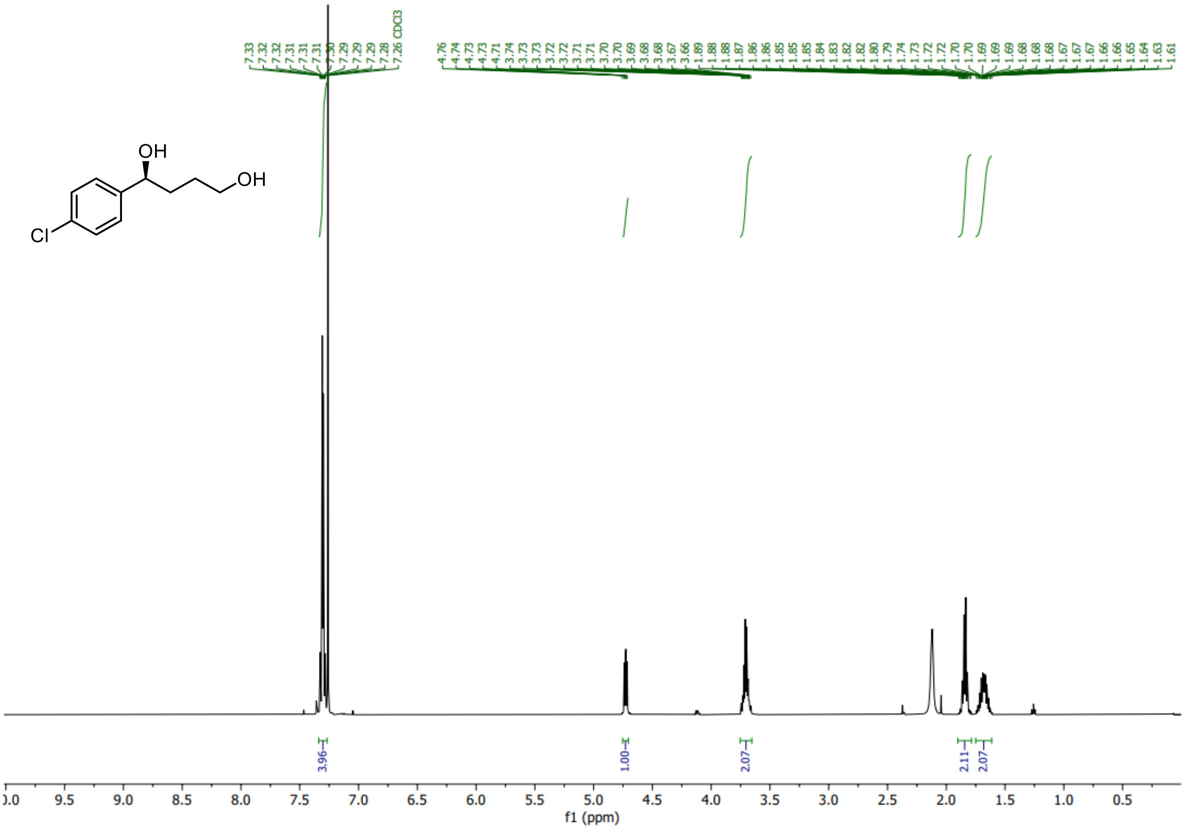

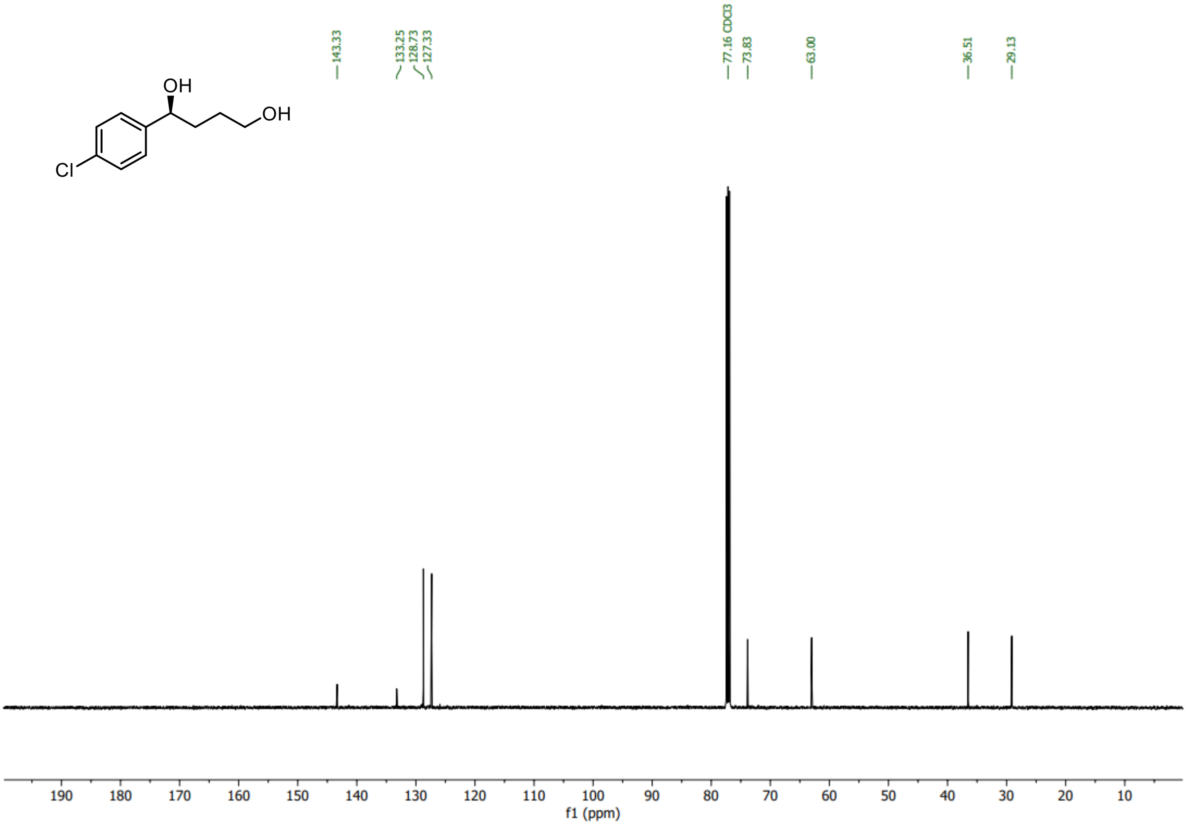

Figure S34. (Top) ^{1}H NMR (500 MHz) and (bottom) $^{13}C\{^{1}H\}$ NMR (126 MHz) spectra of **(*S*)-1j** in $CDCl_3$.

(*S*)-1-(4-bromophenyl)butane-1,4-diol [(*S*)-1k]

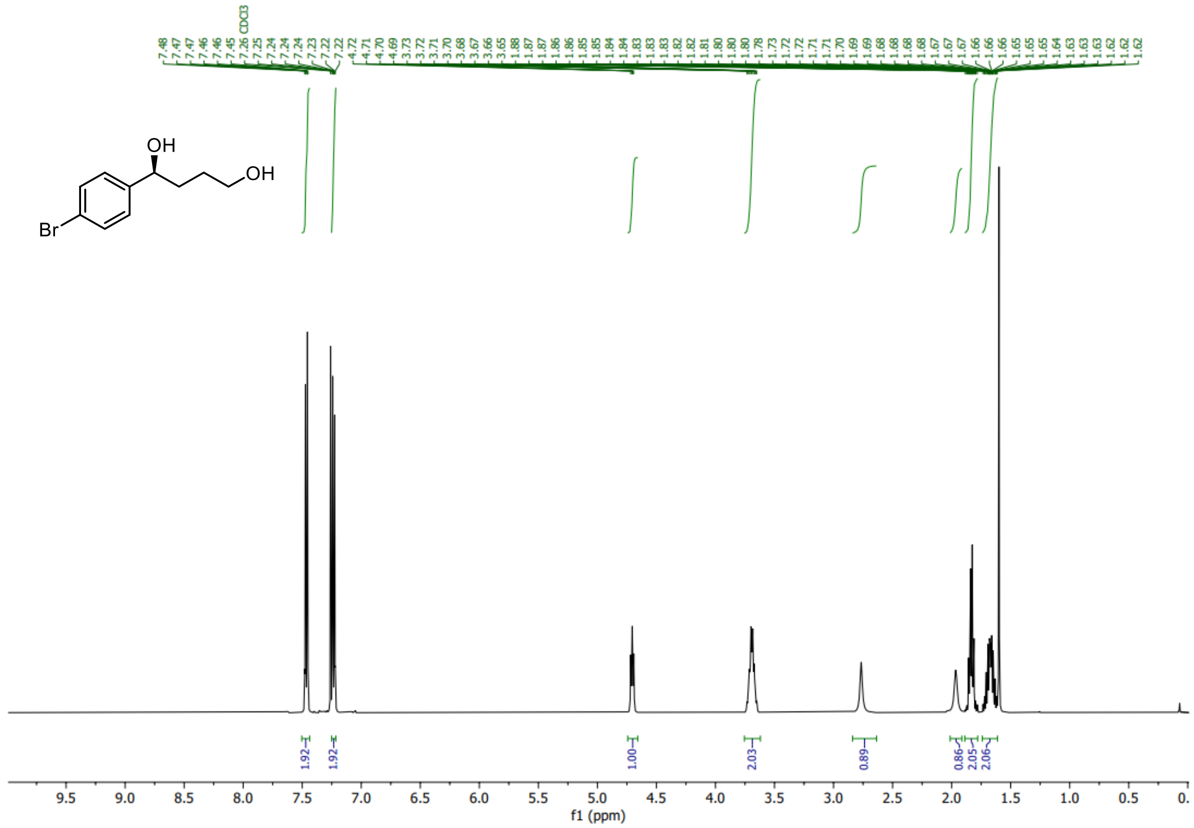

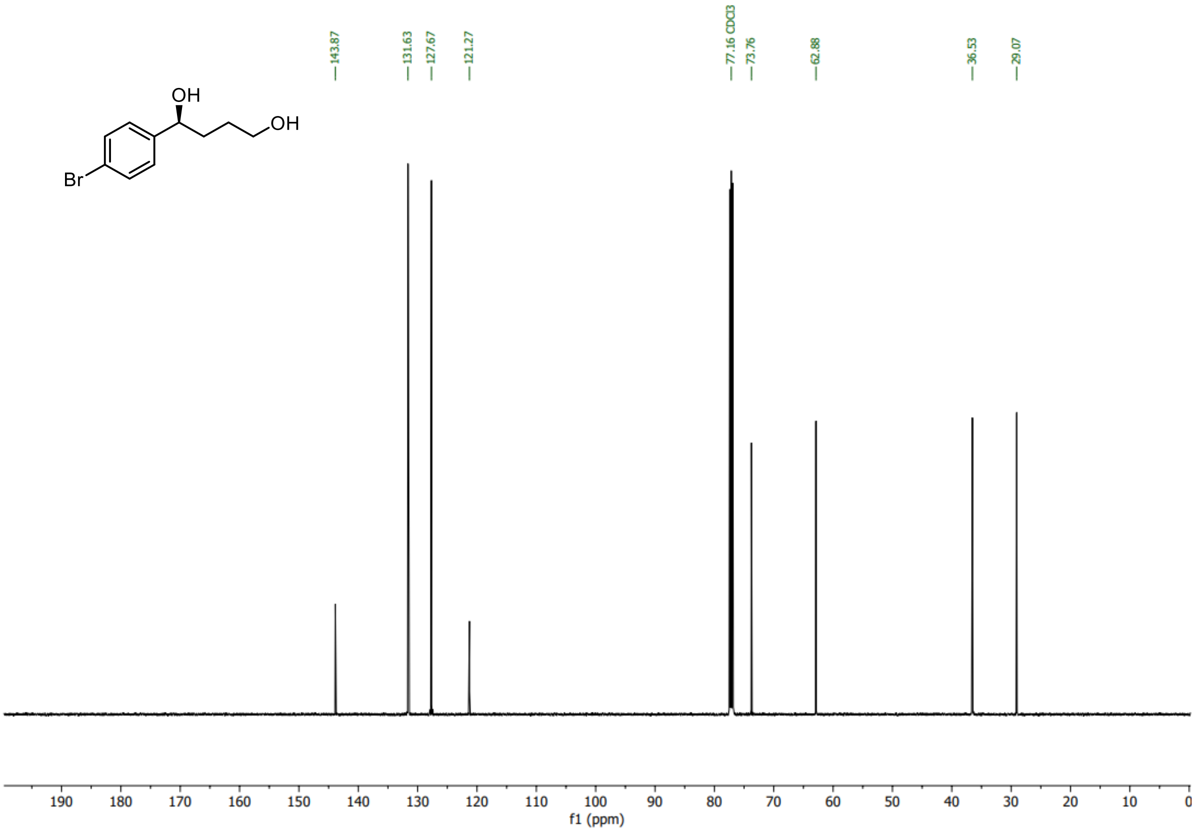

Figure S35. (Top) ^{1}H NMR (500 MHz) and (bottom) ^{13}C{^{1}H} NMR (126 MHz) spectra of **(*S*)-1k** in $CDCl_3$.

(*S*)-1-(4-ethylphenyl)butane-1,4-diol [(*S*)-1n]

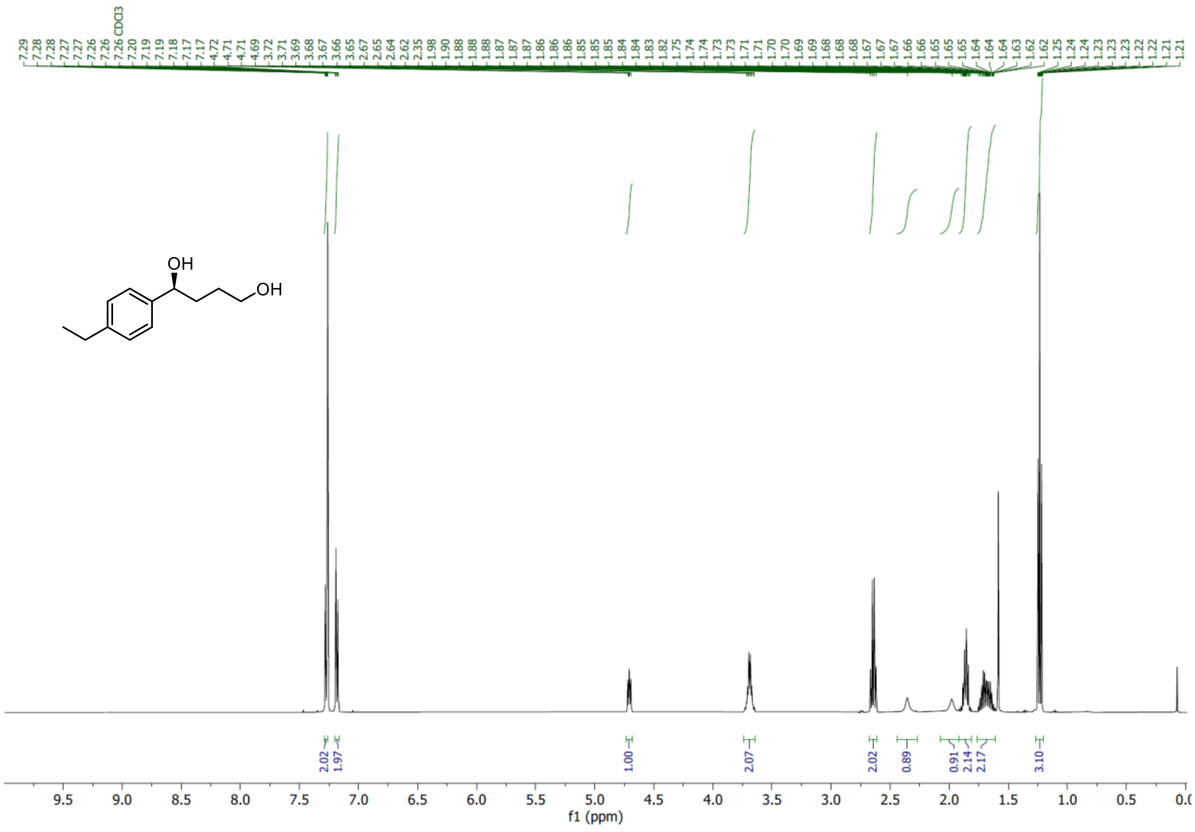

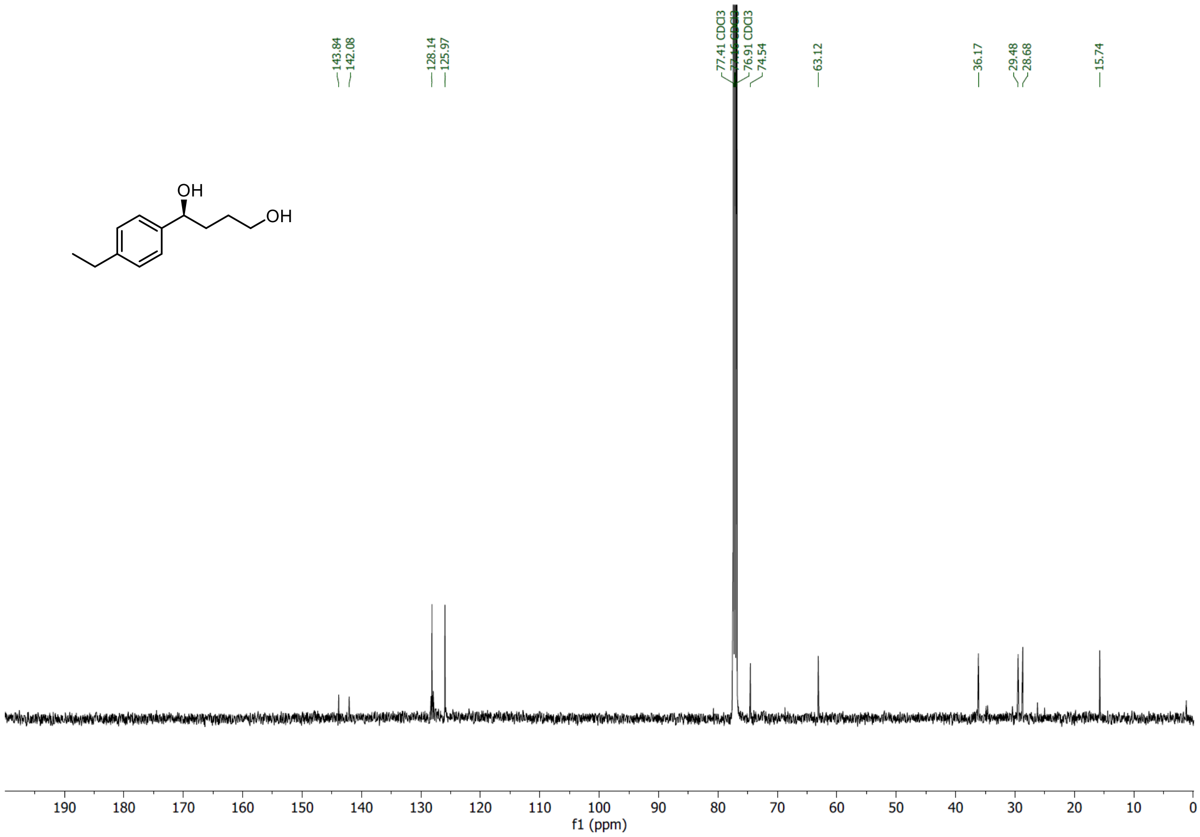

Figure S36. (Top) ^{1}H NMR (500 MHz) and (bottom) $^{13}C\{^{1}H\}$ NMR (126 MHz) spectra of **(*S*)-1n** in $CDCl_3$.

1-phenylbutane-4,4-d2-1,4-diol (1a-d2)

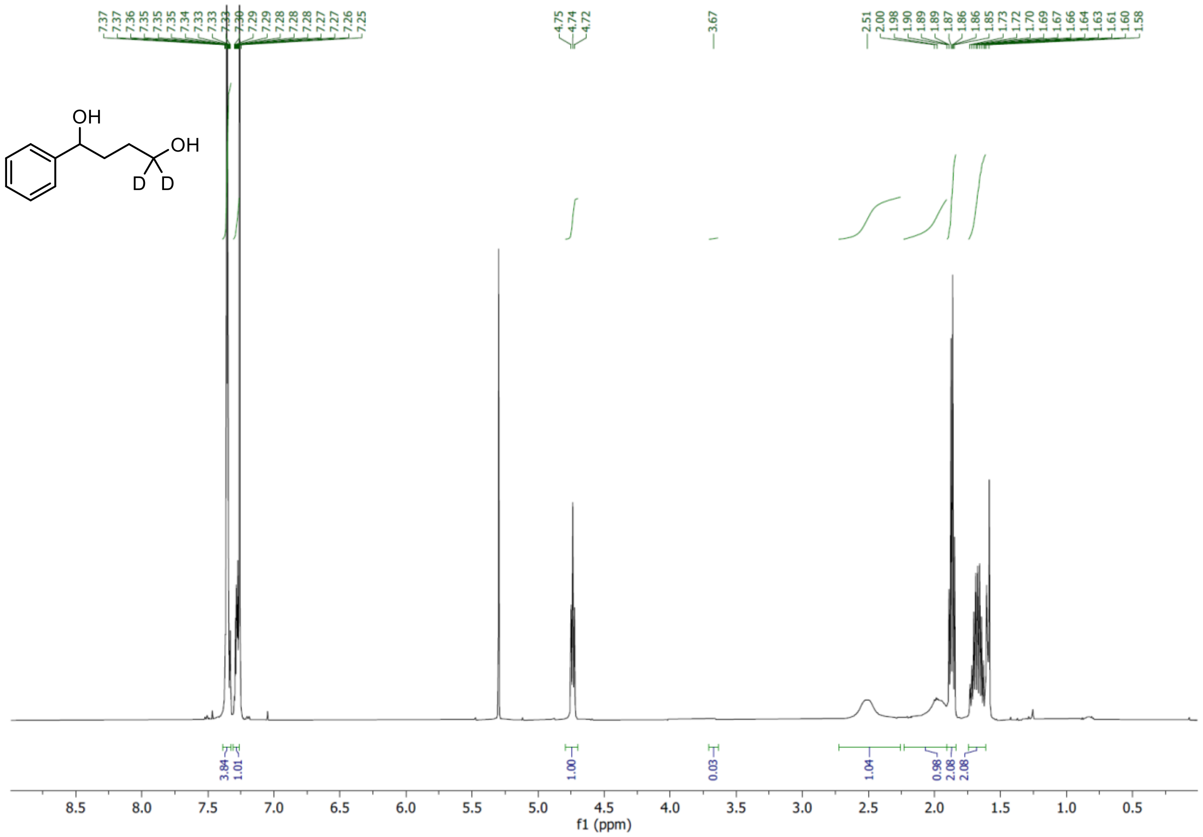

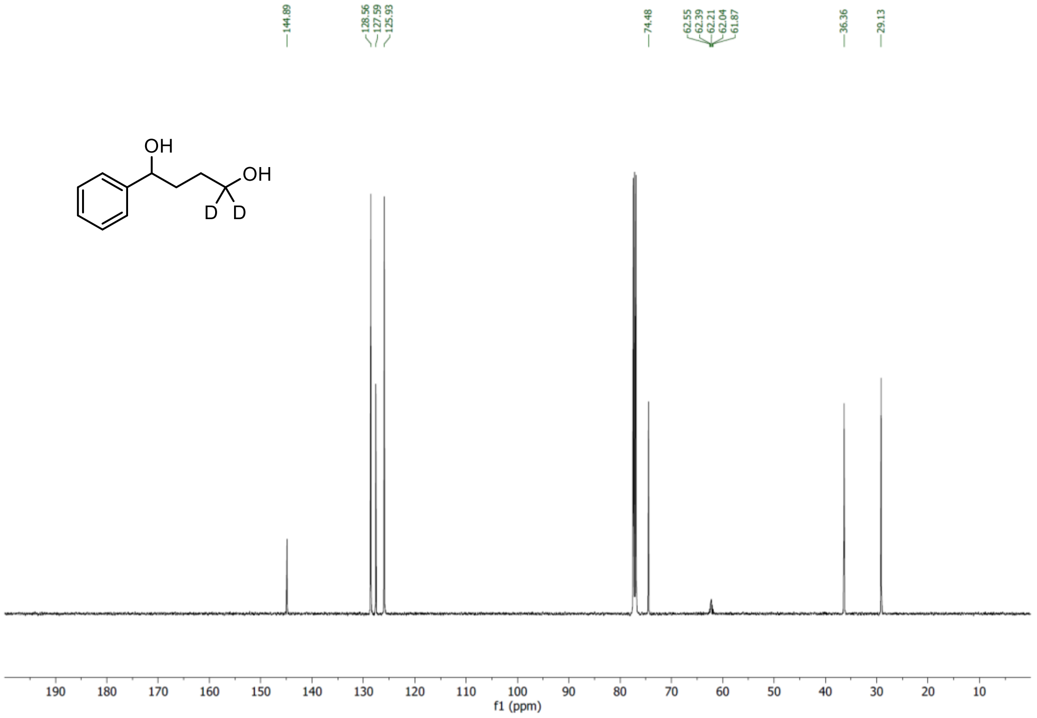

Figure S37. (Top) ^{1}H NMR (500 MHz) and (bottom) $^{13}C\{^{1}H\}$ NMR (126 MHz) spectra of **1a-d₂** in $CDCl_3$.

1.7.2 Spectra of *N*-alkylated anilines with 1,4-diols (**3**)

1–phenyl–4–(phenylamino)butan–1–ol (3aa)

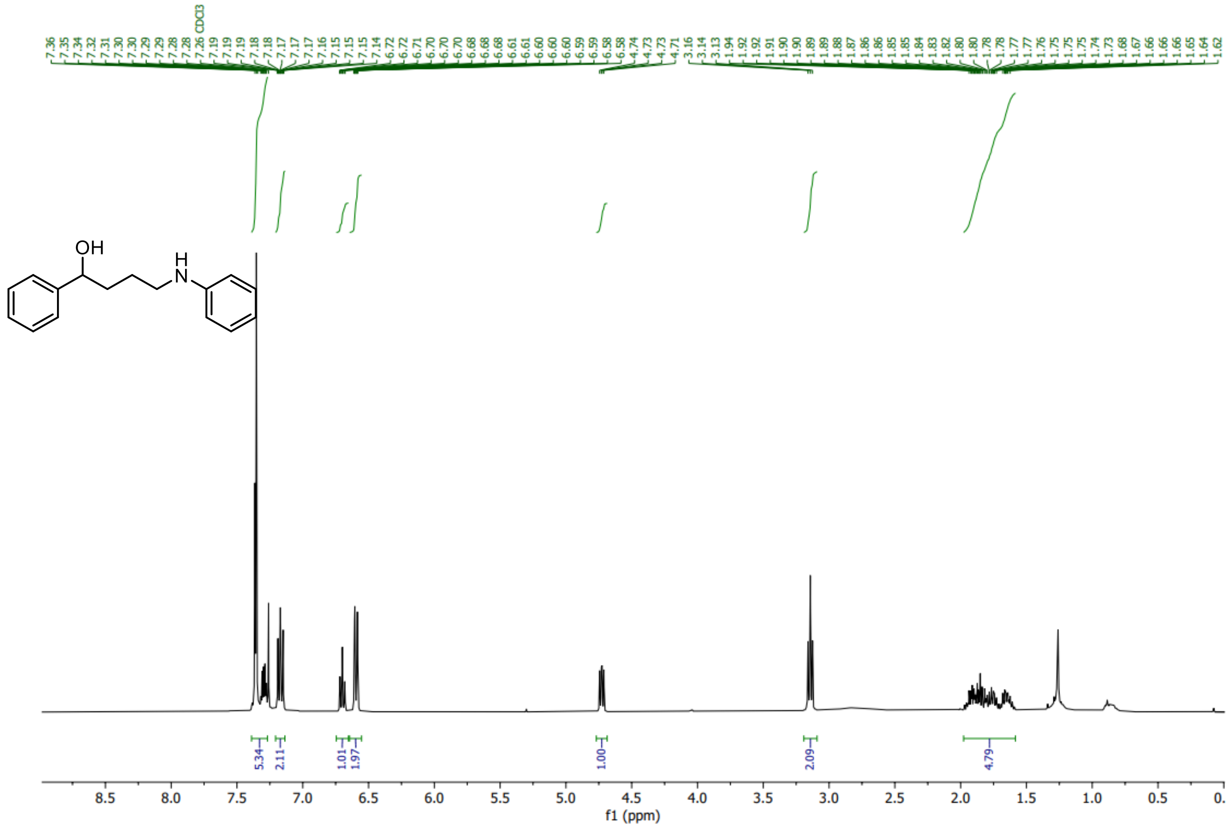

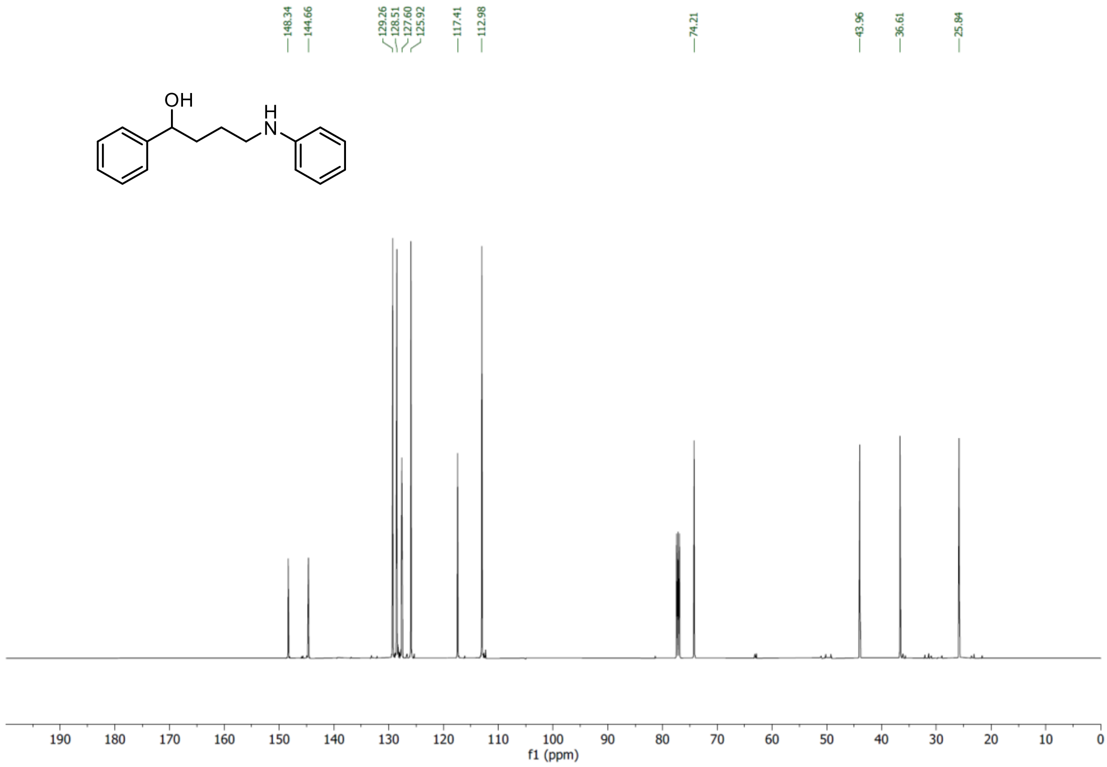

Figure S38. (Top) ^{1}H NMR (500 MHz) and (bottom) $^{13}C\{^{1}H\}$ NMR (126 MHz) spectra of **3aa** in $CDCl_3$.

1–(2–chlorophenyl)–4–(phenylamino)butan–1–ol (3ba)

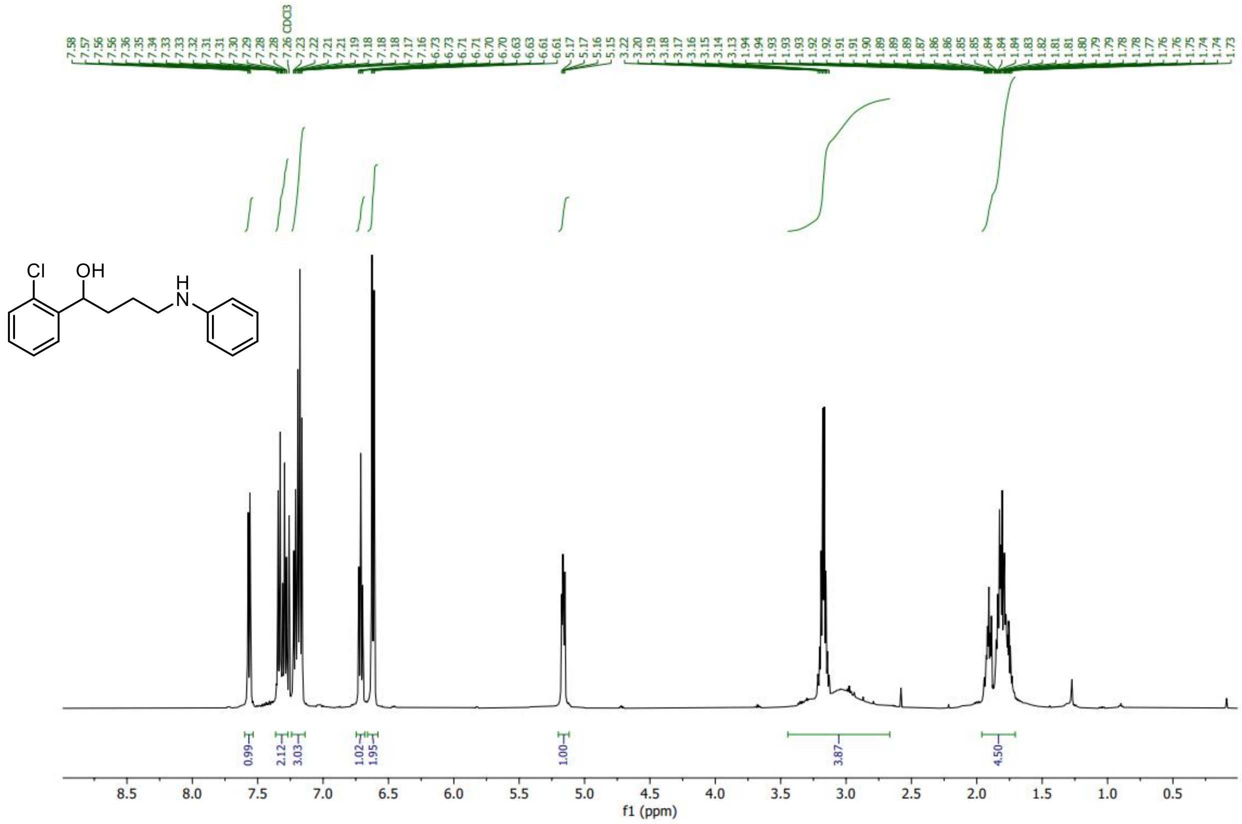

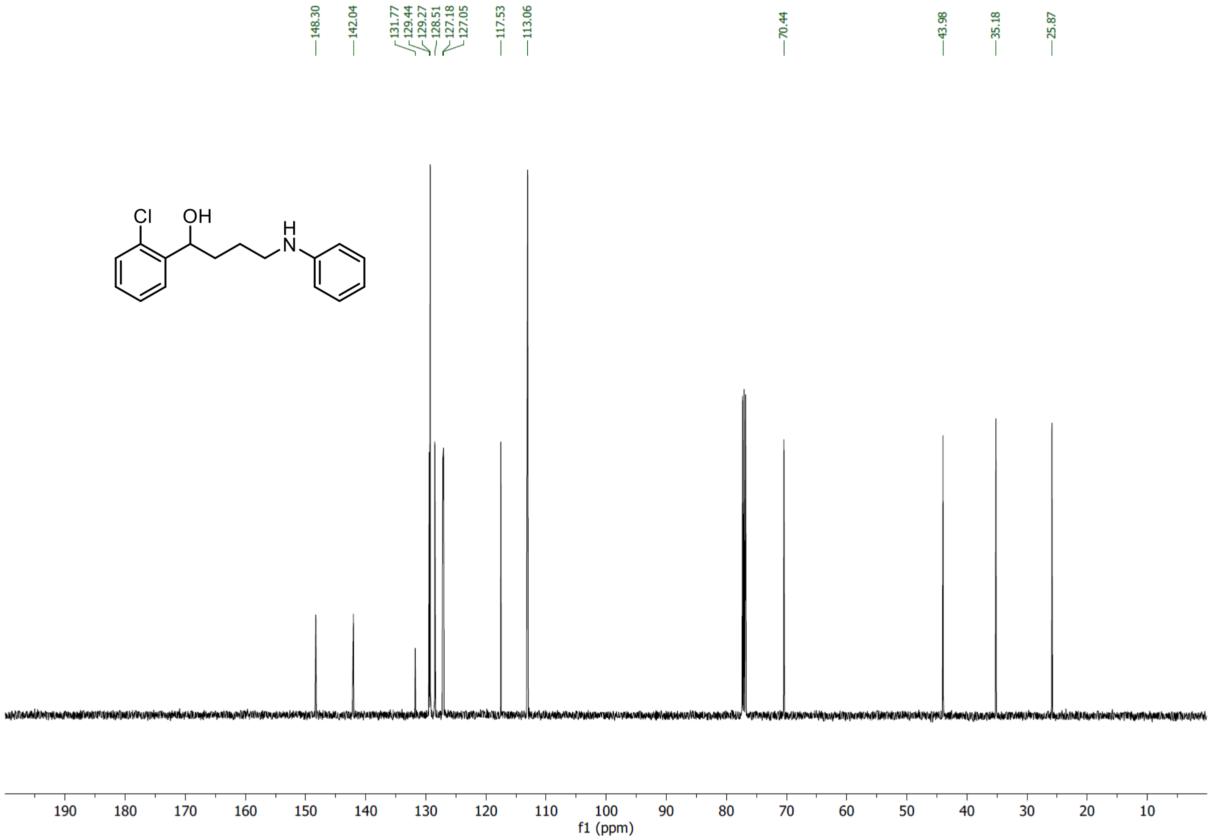

Figure S39. (Top) ^{1}H NMR (500 MHz) and (bottom) $^{13}C\{^{1}H\}$ NMR (126 MHz) spectra of **3ba** in $CDCl_3$.

1–(2–bromophenyl)–4–(phenylamino)butan–1–ol (3ca)

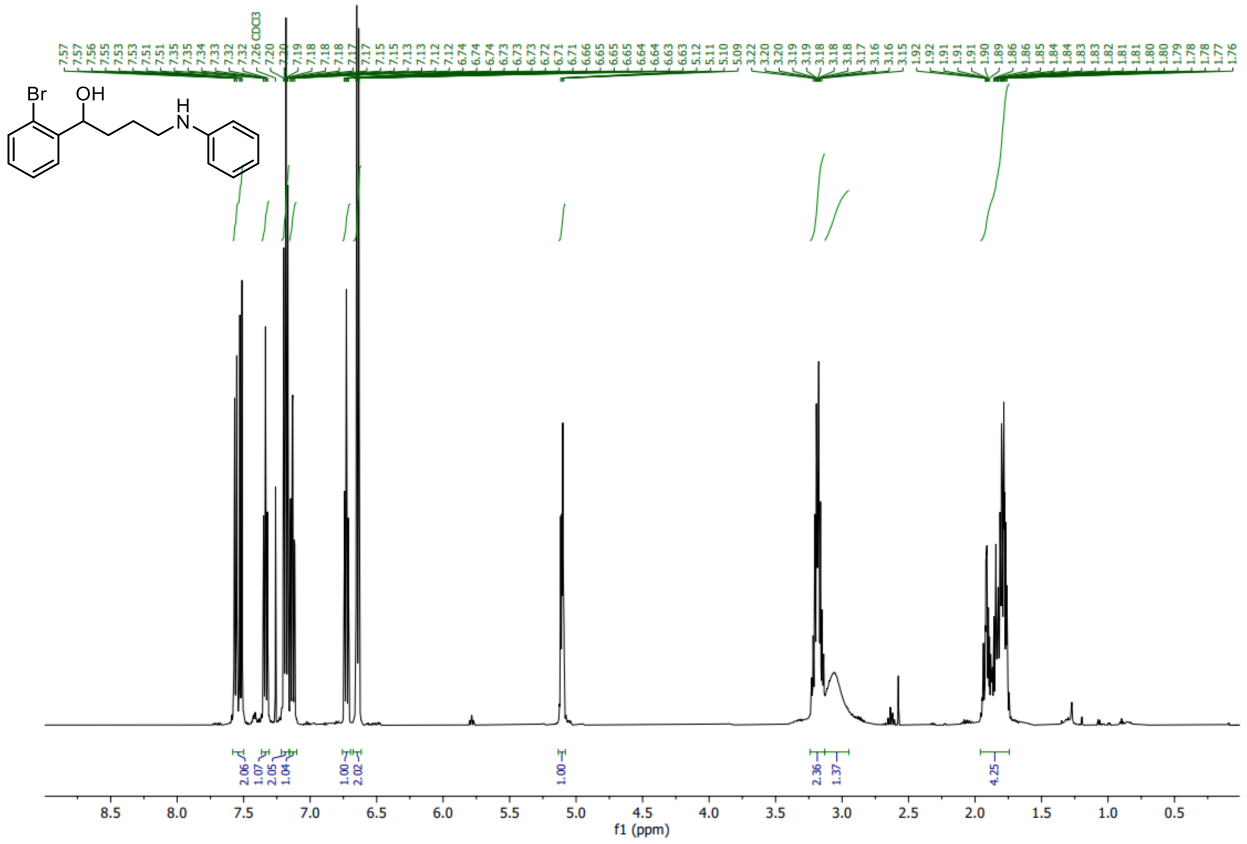

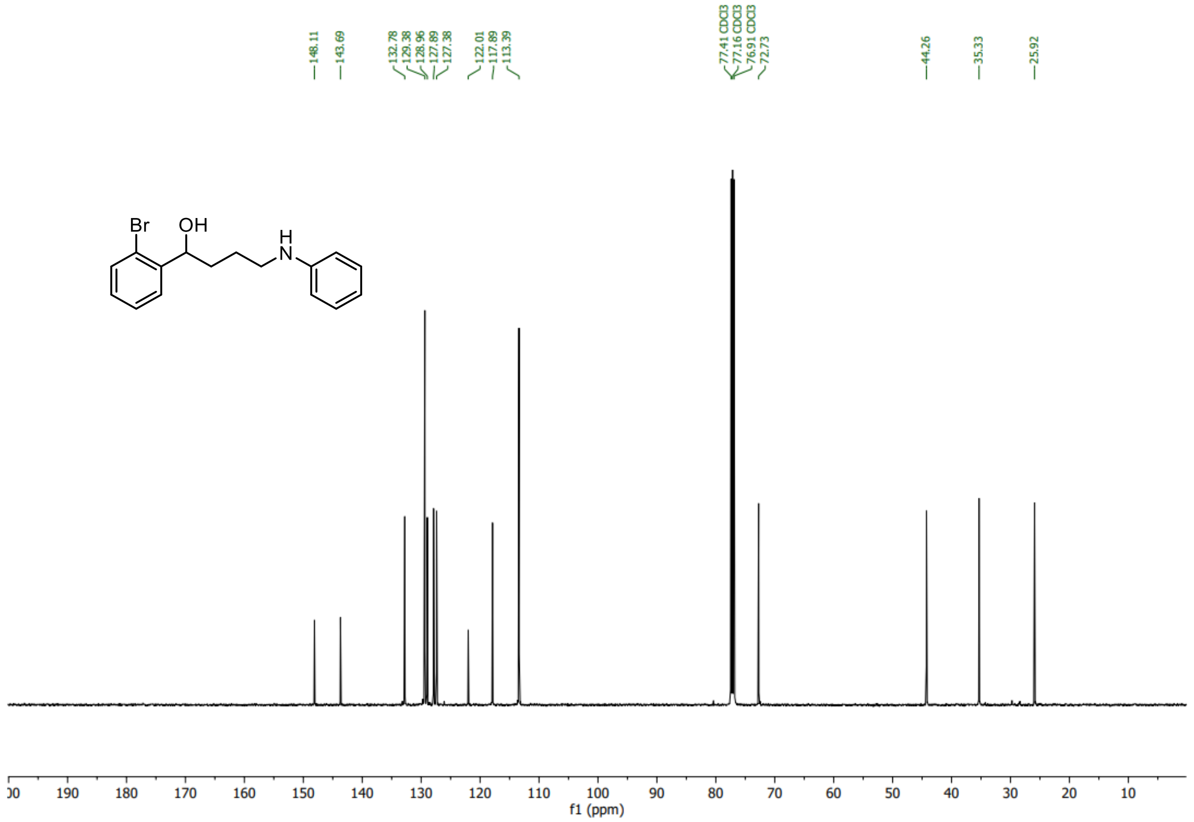

Figure S40. (Top) ^{1}H NMR (500 MHz) and (bottom) $^{13}C\{^{1}H\}$ NMR (126 MHz) spectra of **3ca** in $CDCl_3$.

4–(phenylamino)–1–(o–tolyl)butan–1–ol (3da)

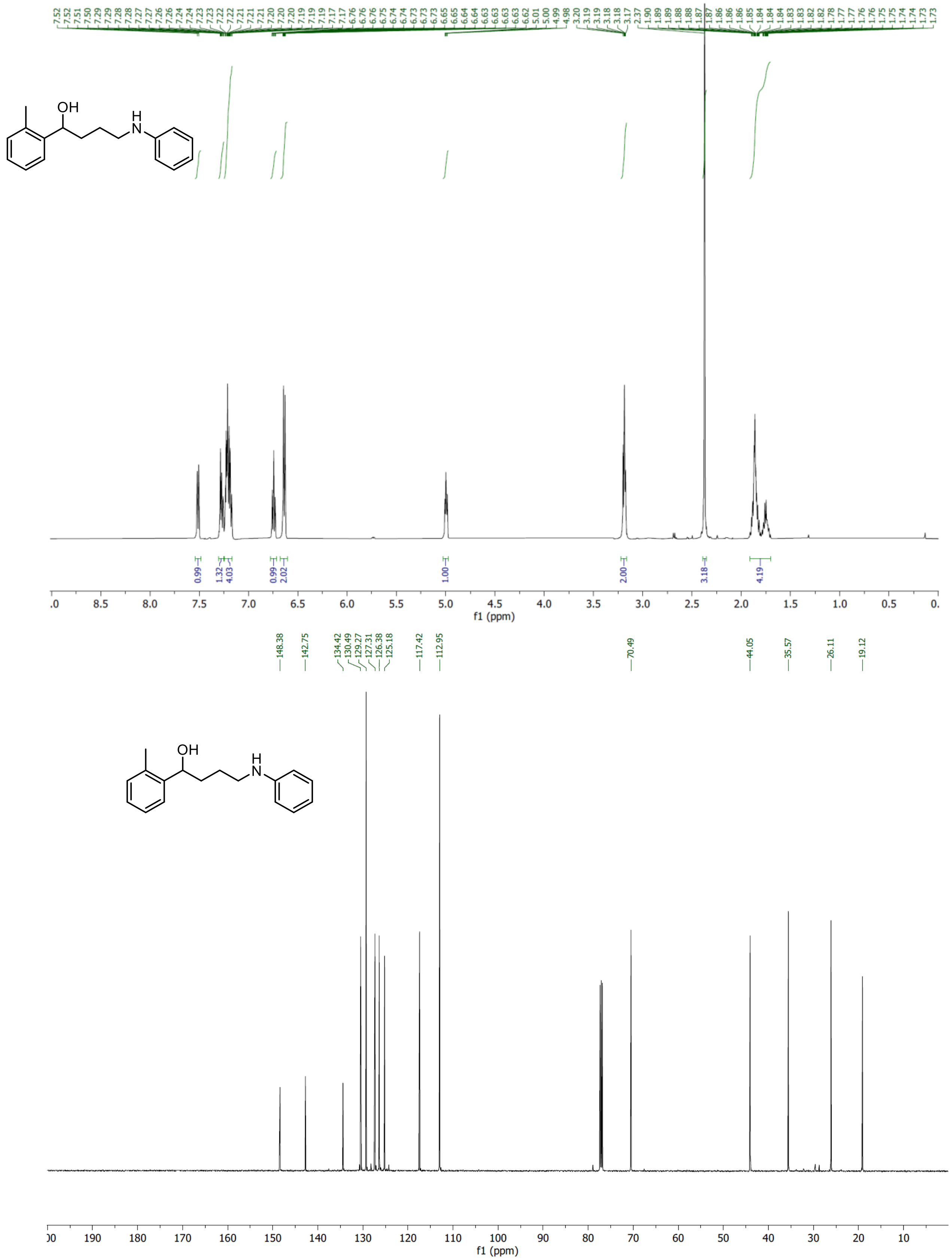

Figure S41. (Top) ^{1}H NMR (500 MHz) and (bottom) $^{13}C\{^{1}H\}$ NMR (126 MHz) spectra of **3da** in $CDCl_3$.

1–(2–methoxyphenyl)–4–(phenylamino)butan–1–ol (3ea)

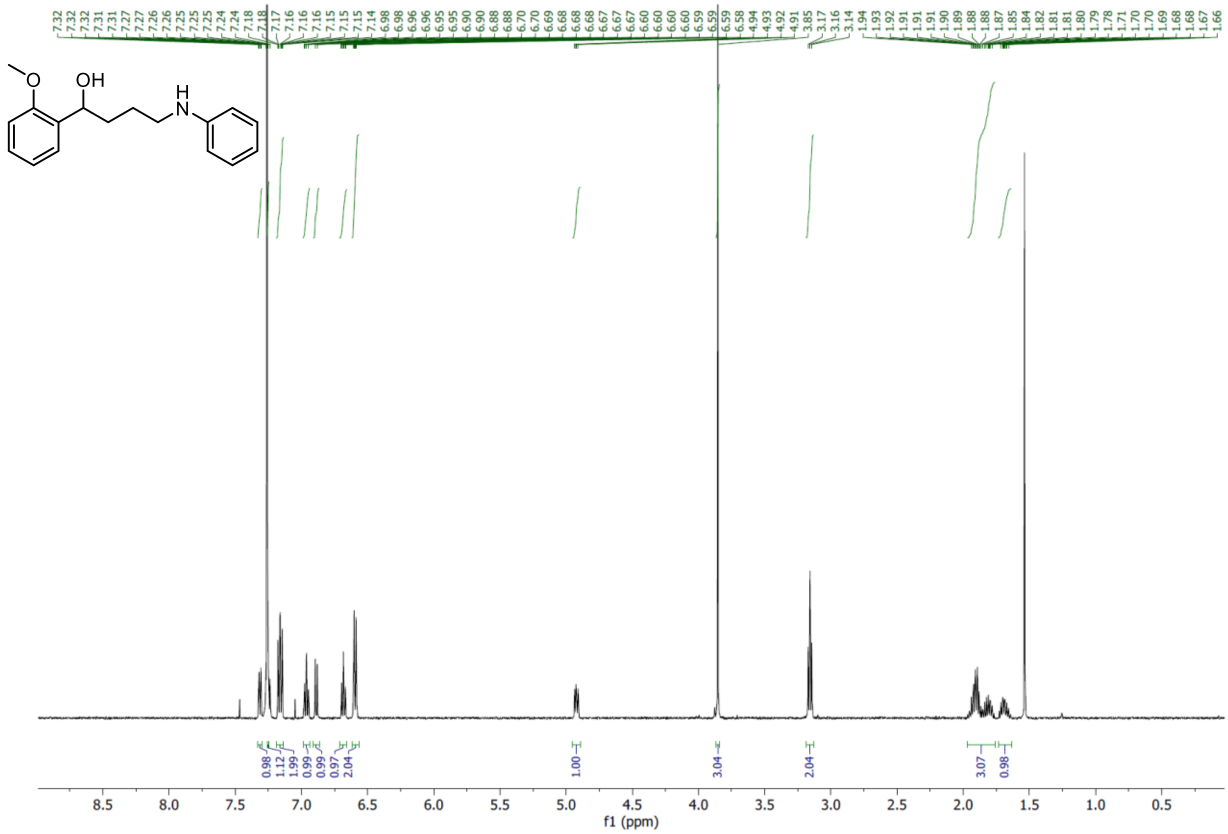

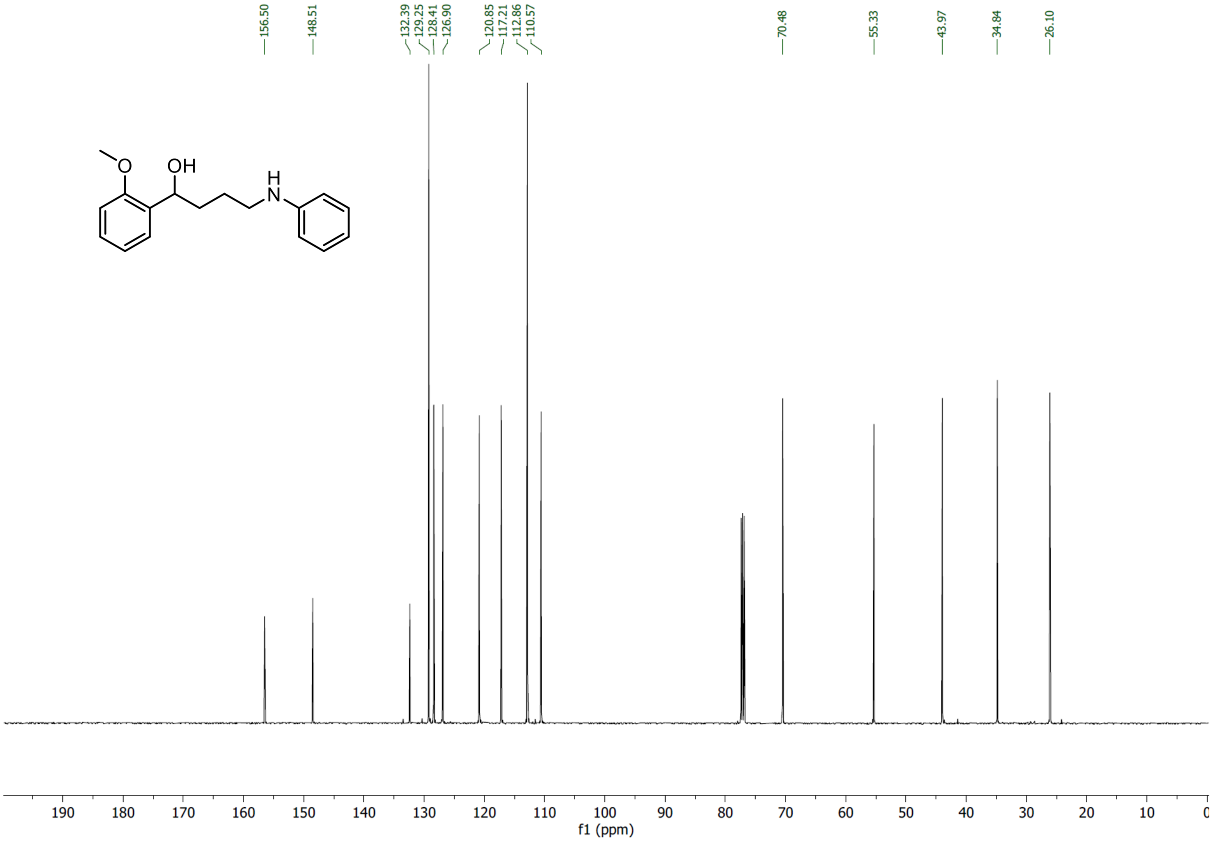

Figure S42. (Top) ^{1}H NMR (500 MHz) and (bottom) $^{13}C\{^{1}H\}$ NMR (126 MHz) spectra of **3ea** in $CDCl_3$.

1–(3–methoxyphenyl)–4–(phenylamino)butan–1–ol (3fa)

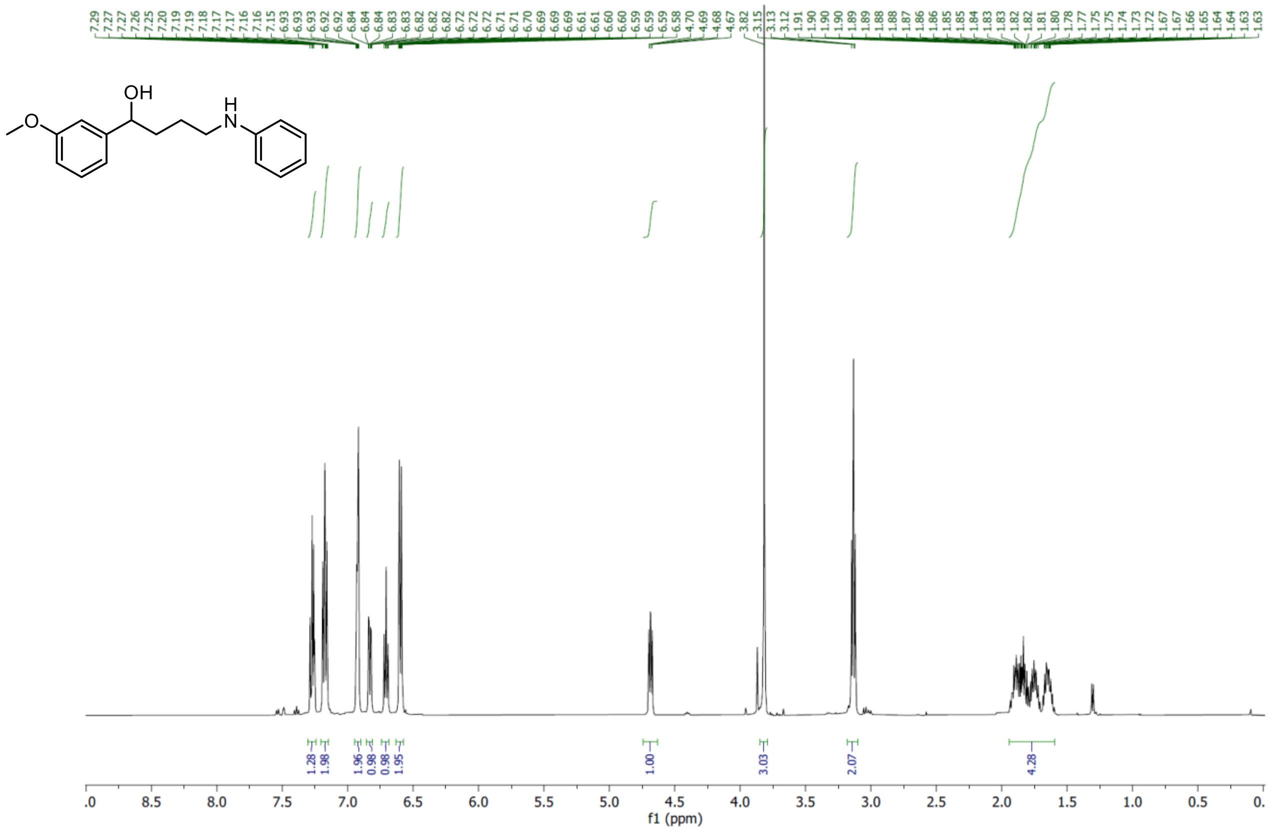

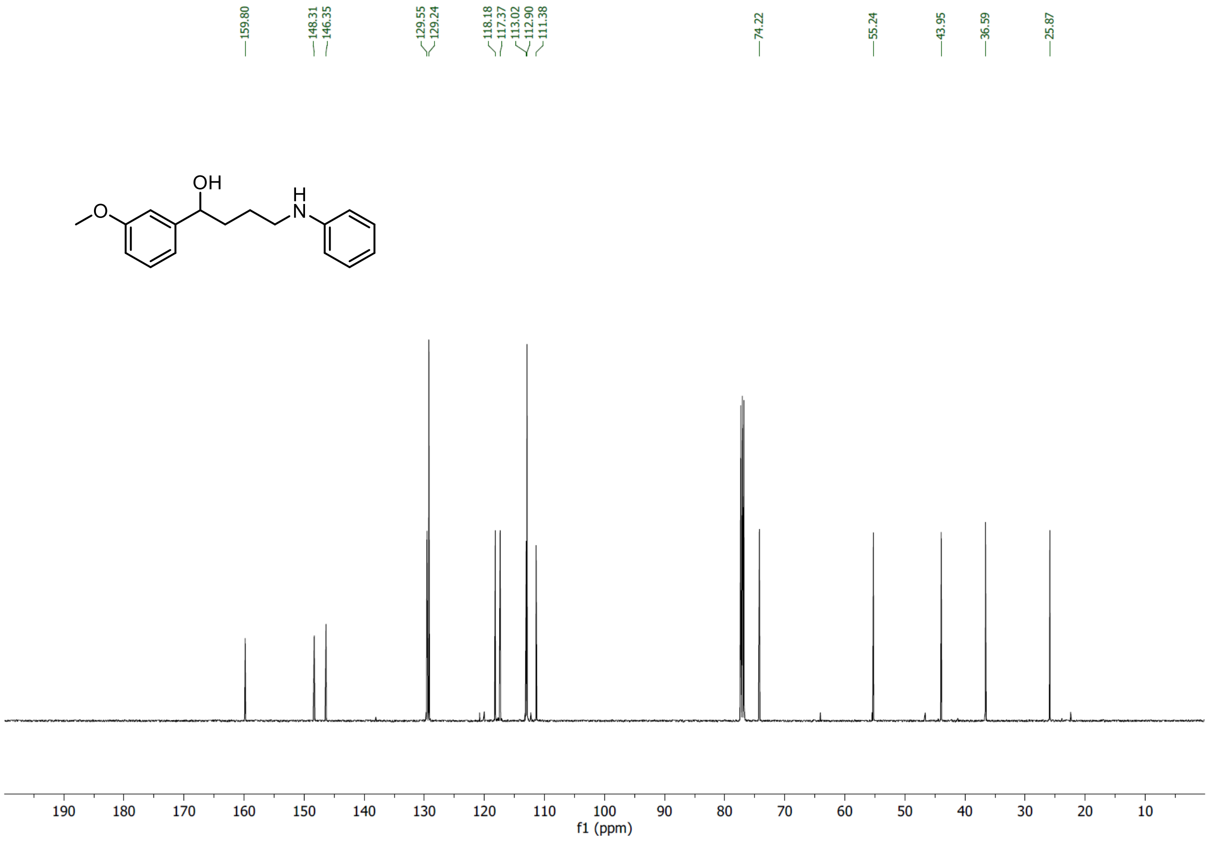

Figure S43. (Top) ^{1}H NMR (500 MHz) and (bottom) ^{13}C{^{1}H} NMR (126 MHz) spectra of **3fa** in $CDCl_3$.

1–(3–methylphenyl)–4–(phenylamino)butan–1–ol (3ga)

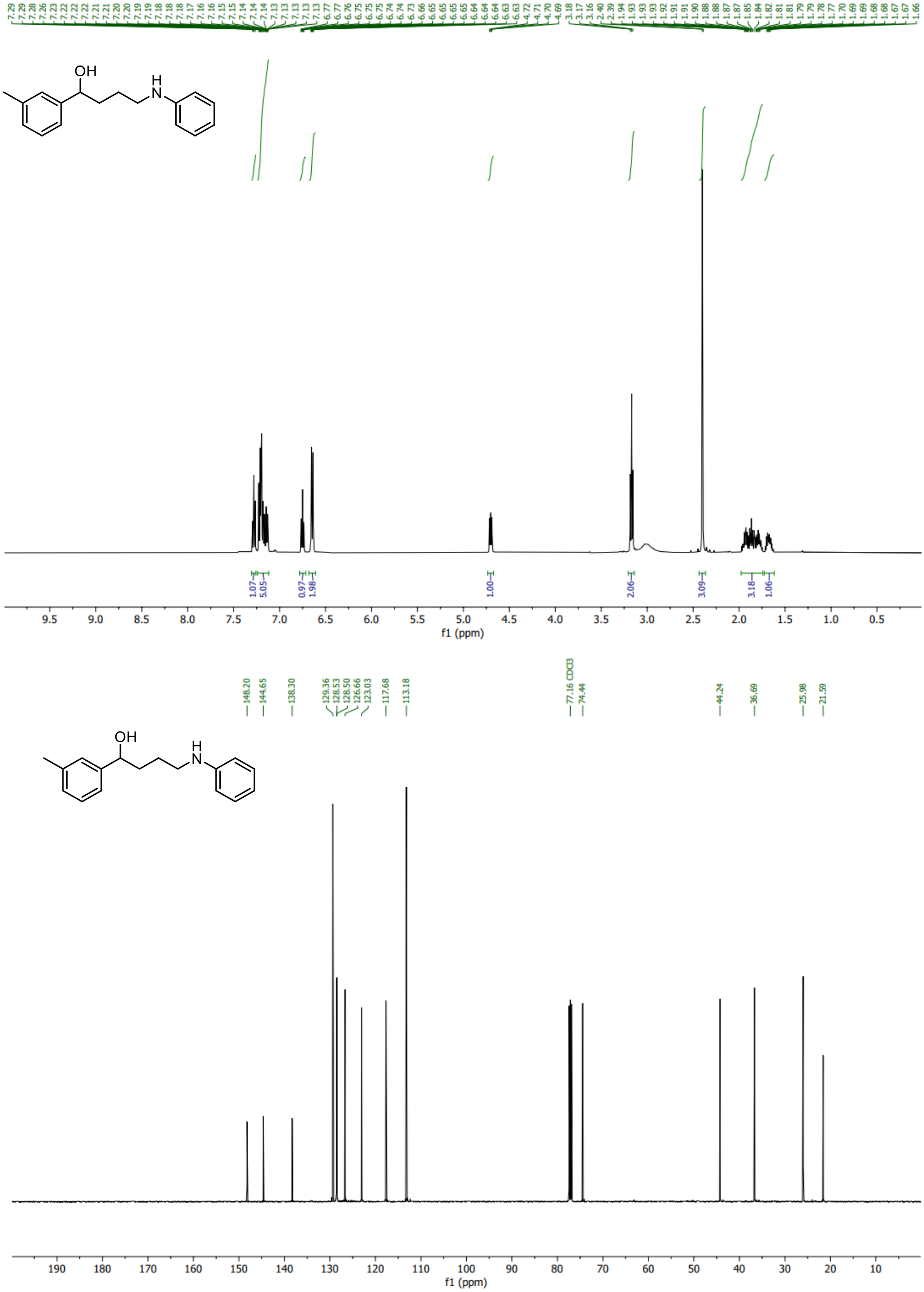

Figure S44. (Top) ^{1}H NMR (500 MHz) and (bottom) $^{13}C\{^{1}H\}$ NMR (126 MHz) spectra of **3ga** in $CDCl_3$.

1–(naphthalen–2–yl)–4–(phenylamino)butan–1–ol (3ha)

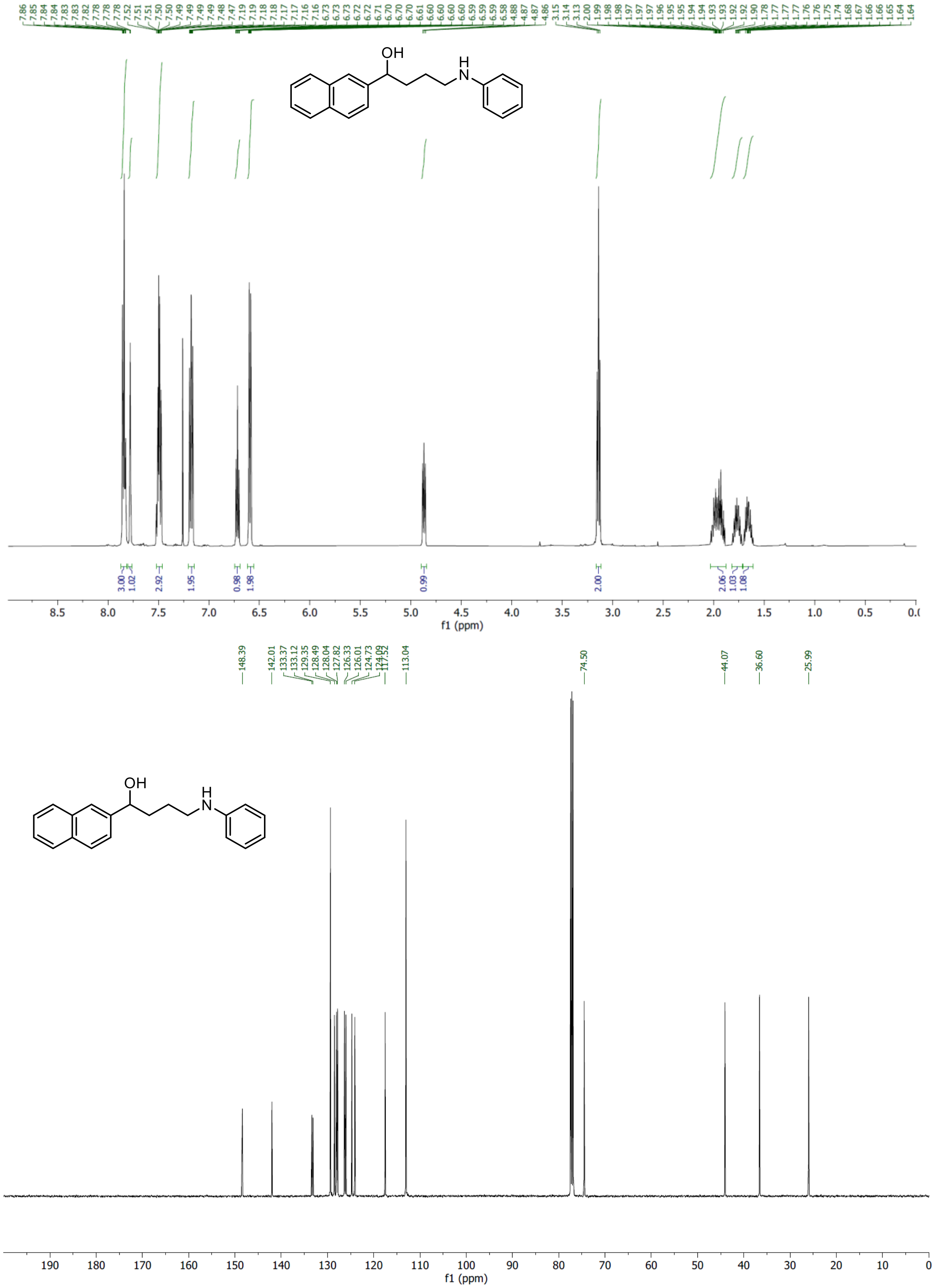

Figure S45. (Top) ^{1}H NMR (500 MHz) and (bottom) ^{13}C{^{1}H} NMR (126 MHz) spectra of **3ha** in $CDCl_3$.

1–(4–fluorophenyl)–4–(phenylamino)butan–1–ol (3ia)

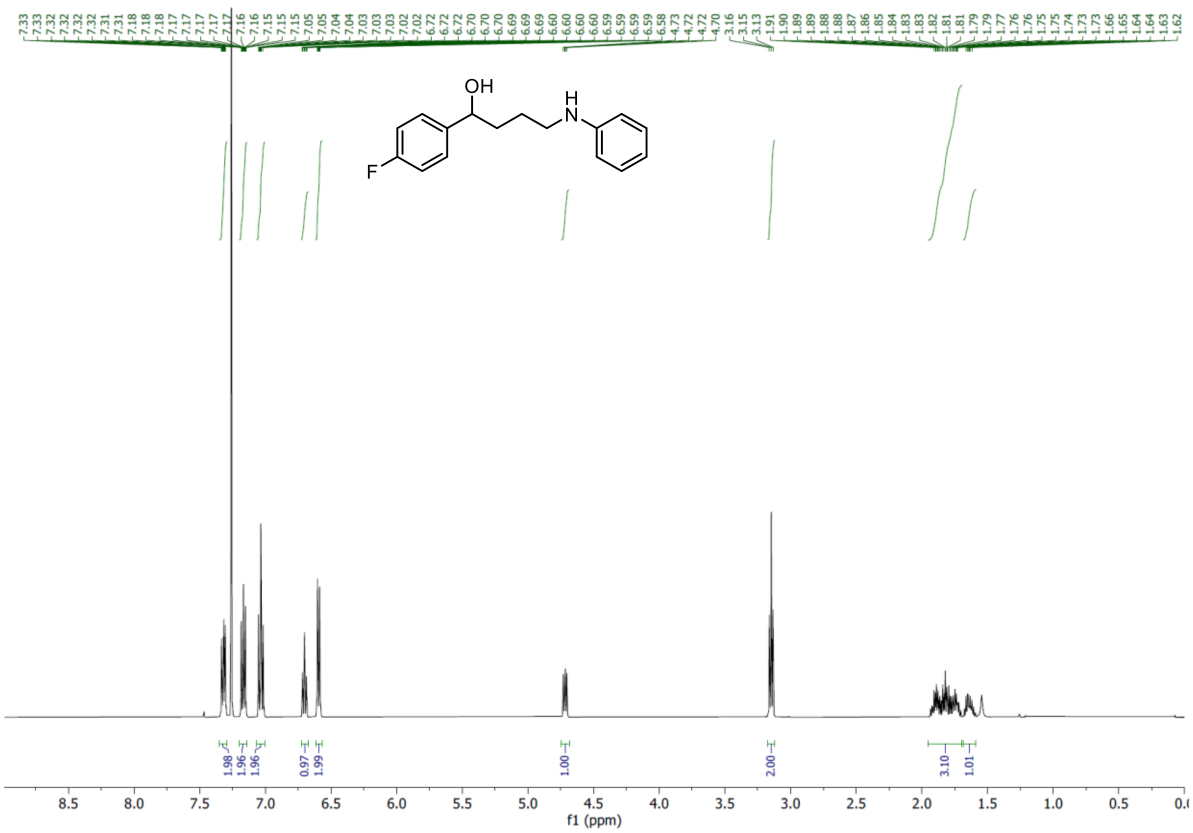

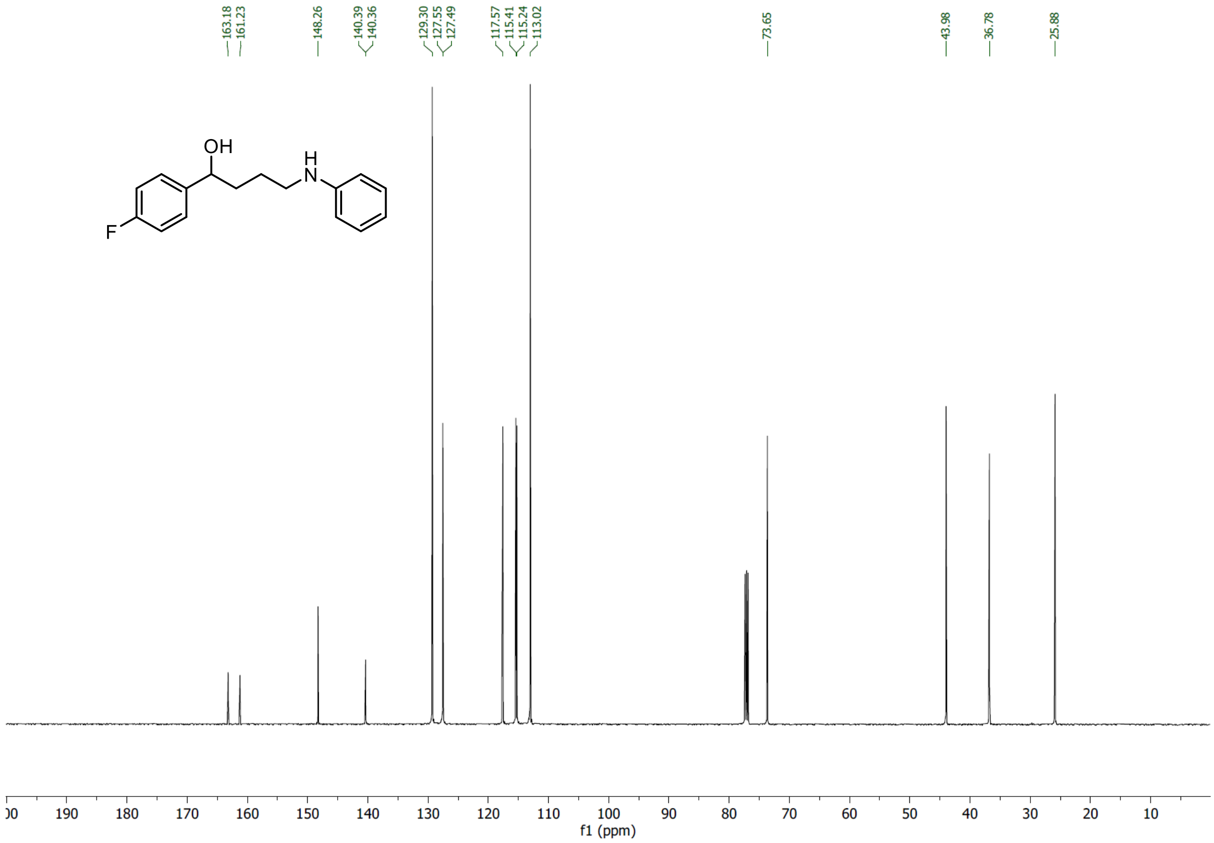

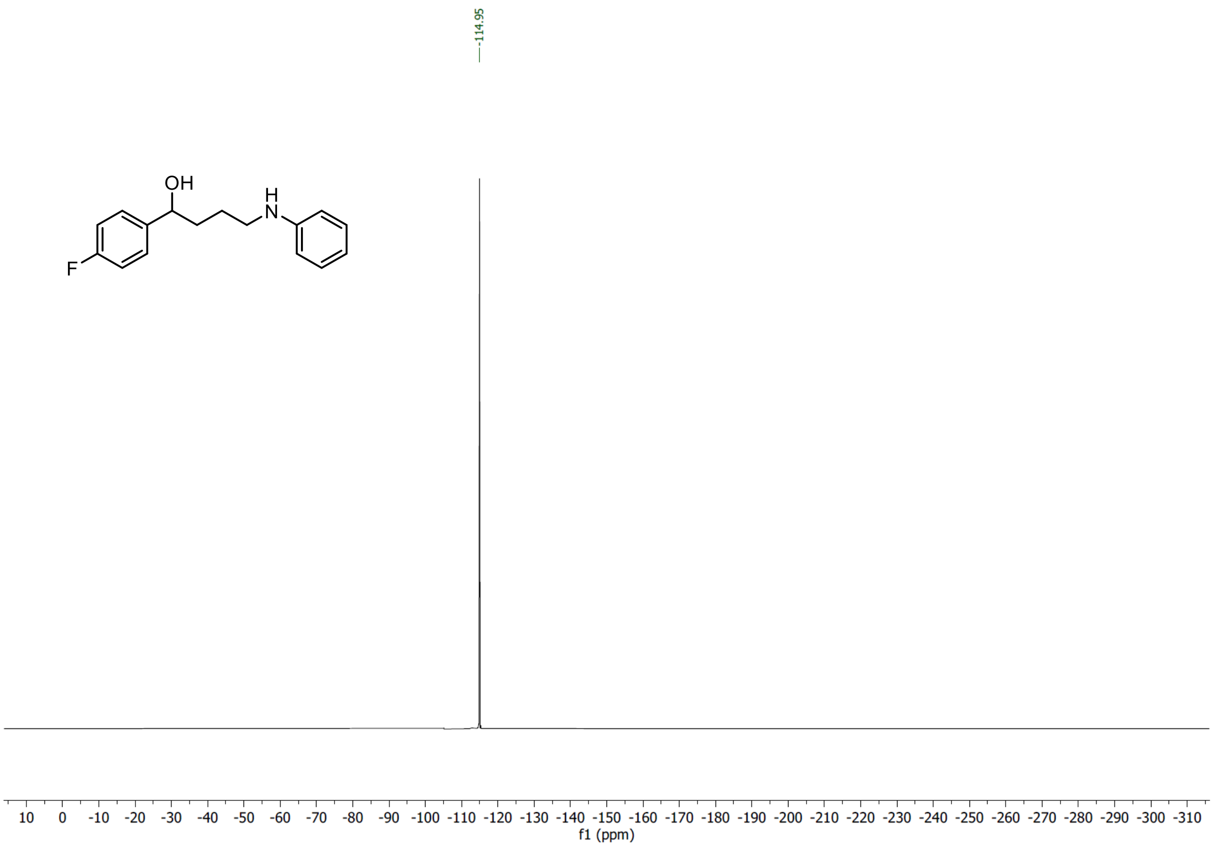

Figure S46. (Top) ^{1}H NMR (500 MHz), (centre) $^{13}C\{^{1}H\}$ NMR (126 MHz) and (bottom) $^{19}F\{^{1}H\}$ NMR (470 MHz) spectra of **3ia** in $CDCl_3$.

1–(4–chlorophenyl)–4–(phenylamino)butan–1–ol (3ja)

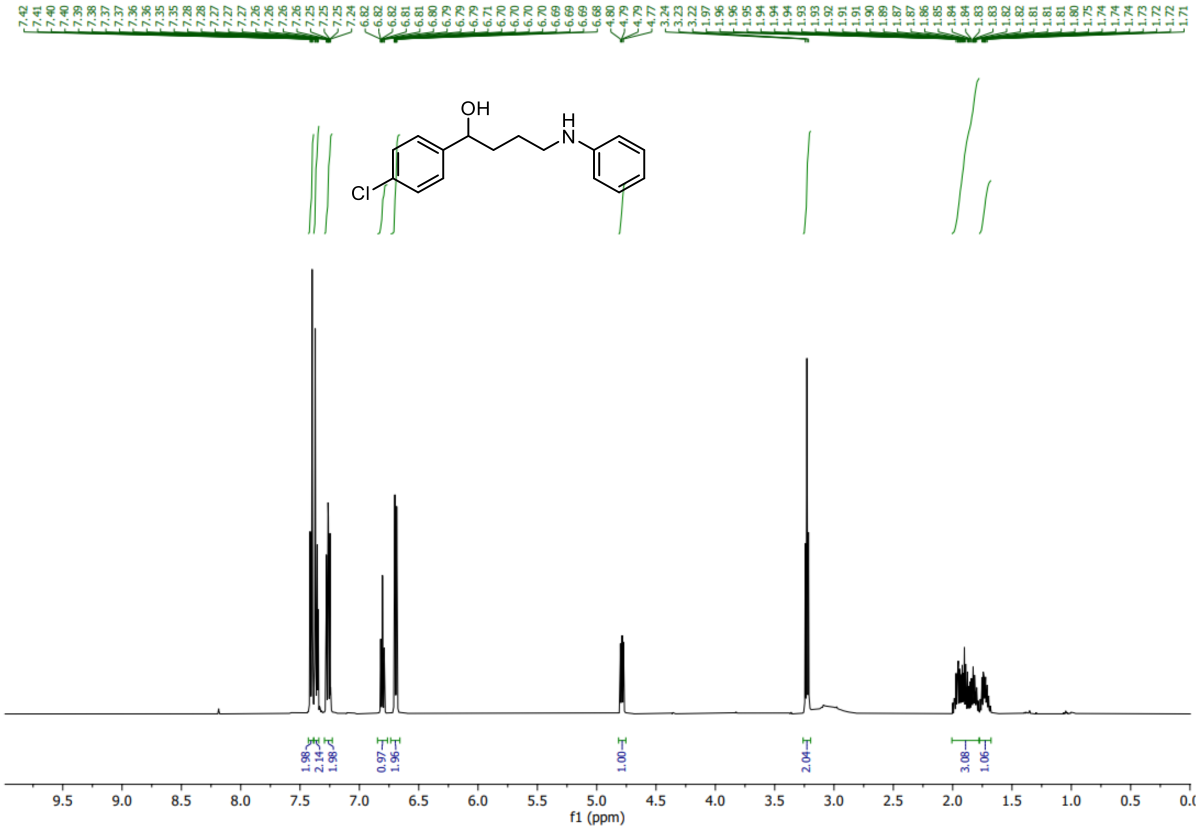

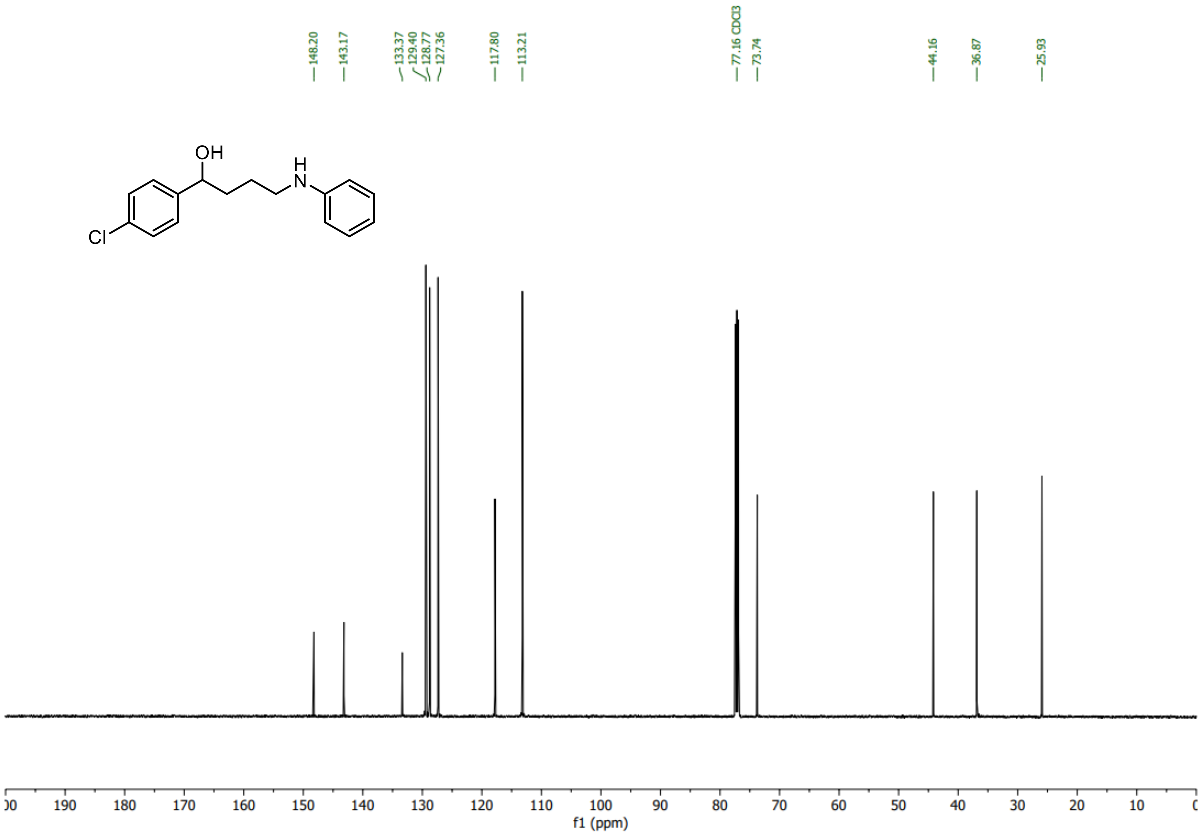

Figure S47. (Top) ^{1}H NMR (500 MHz) and (bottom) $^{13}C\{^{1}H\}$ NMR (126 MHz) spectra of **3ia** in $CDCl_3$.

1–(4–bromophenyl)–4–(phenylamino)butan–1–ol (3ka)

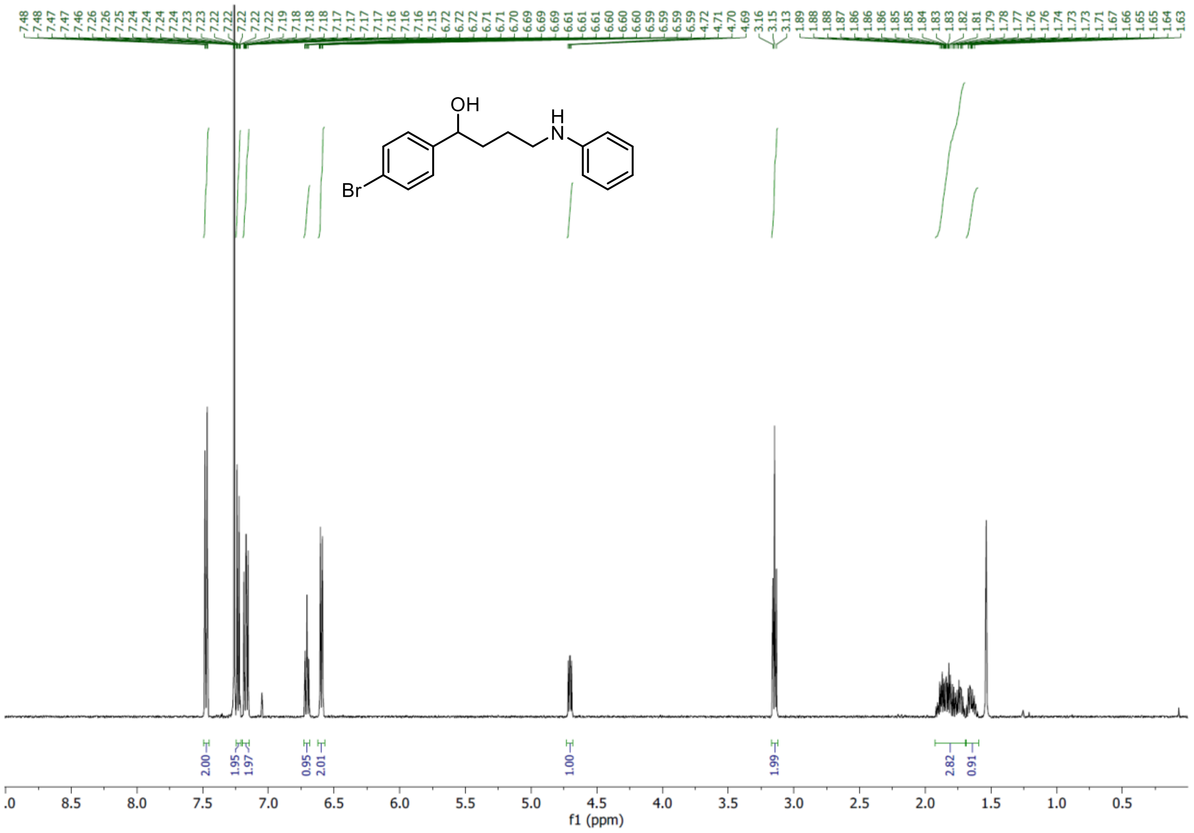

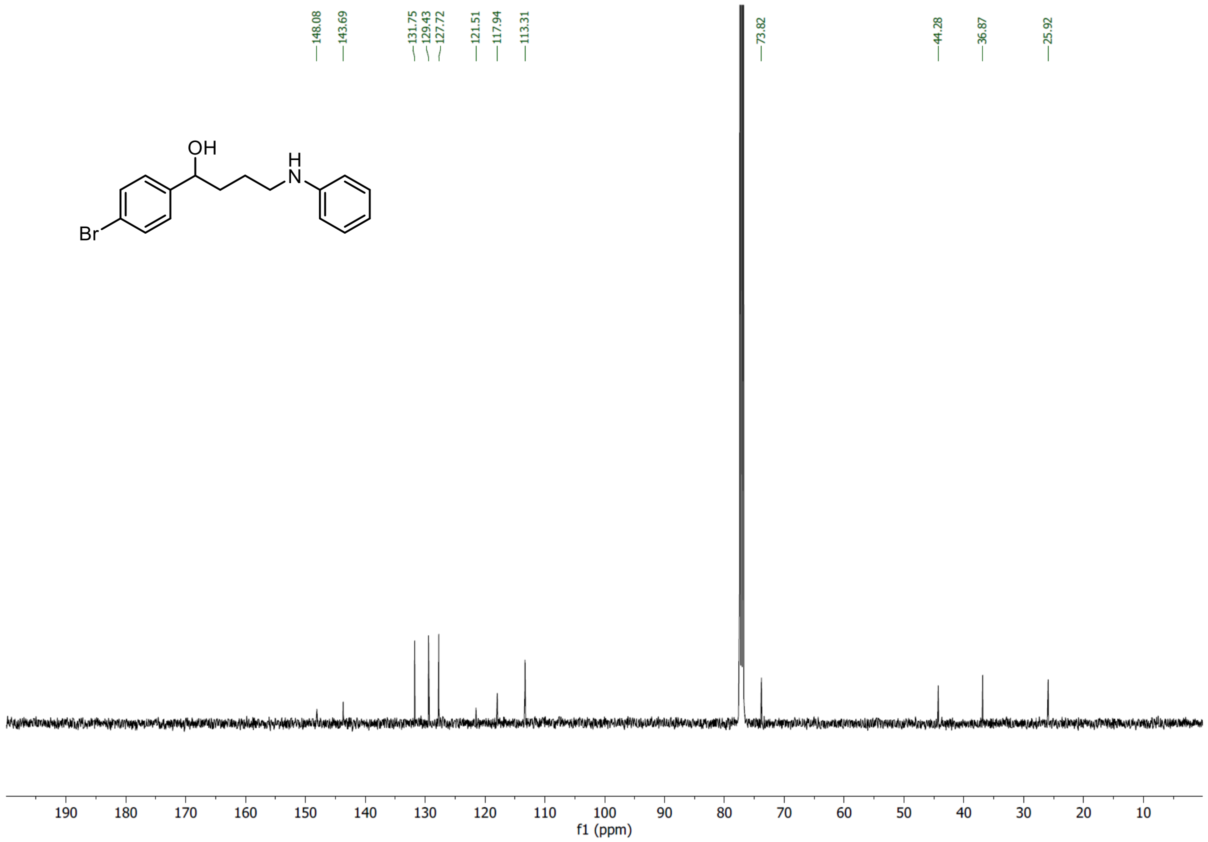

Figure S48. (Top) ^{1}H NMR (500 MHz) and (bottom) $^{13}C\{^{1}H\}$ NMR (126 MHz) spectra of **3ka** in $CDCl_3$.

4–(phenylamino)–1–(p–tolyl)butan–1–ol (3la)

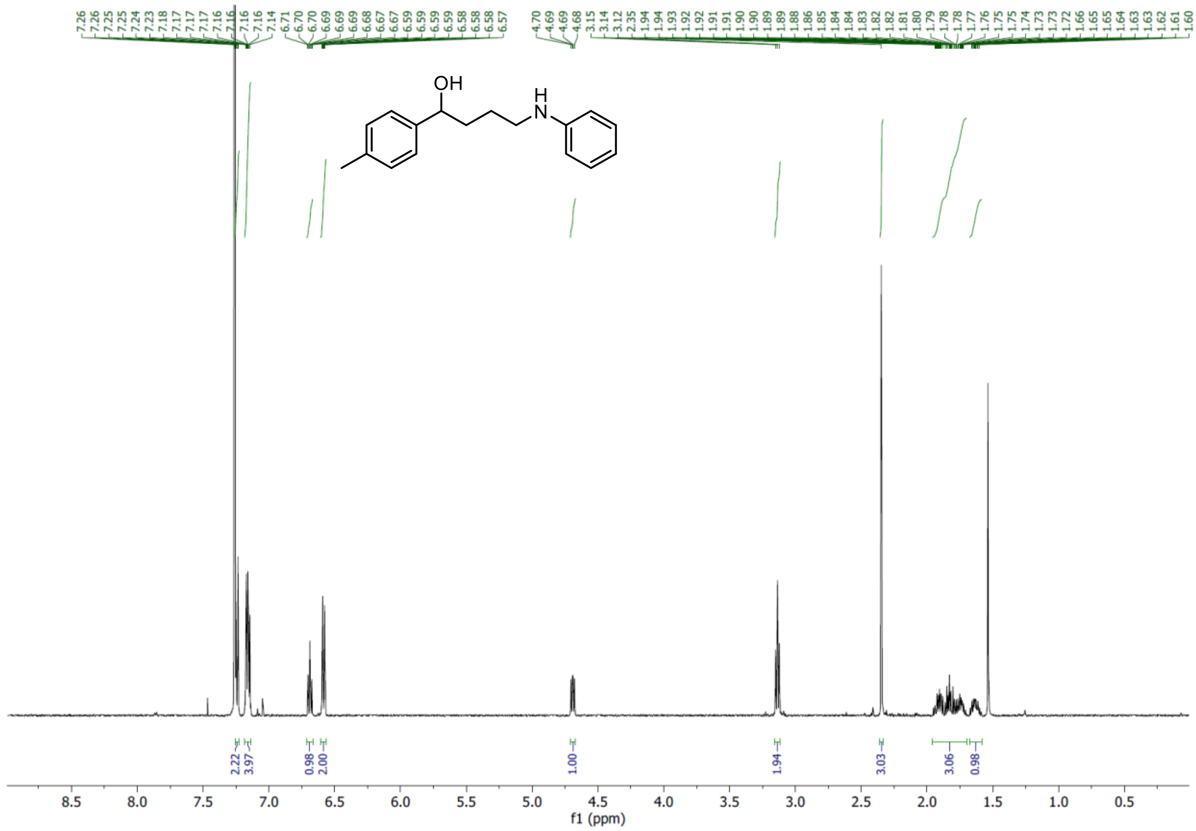

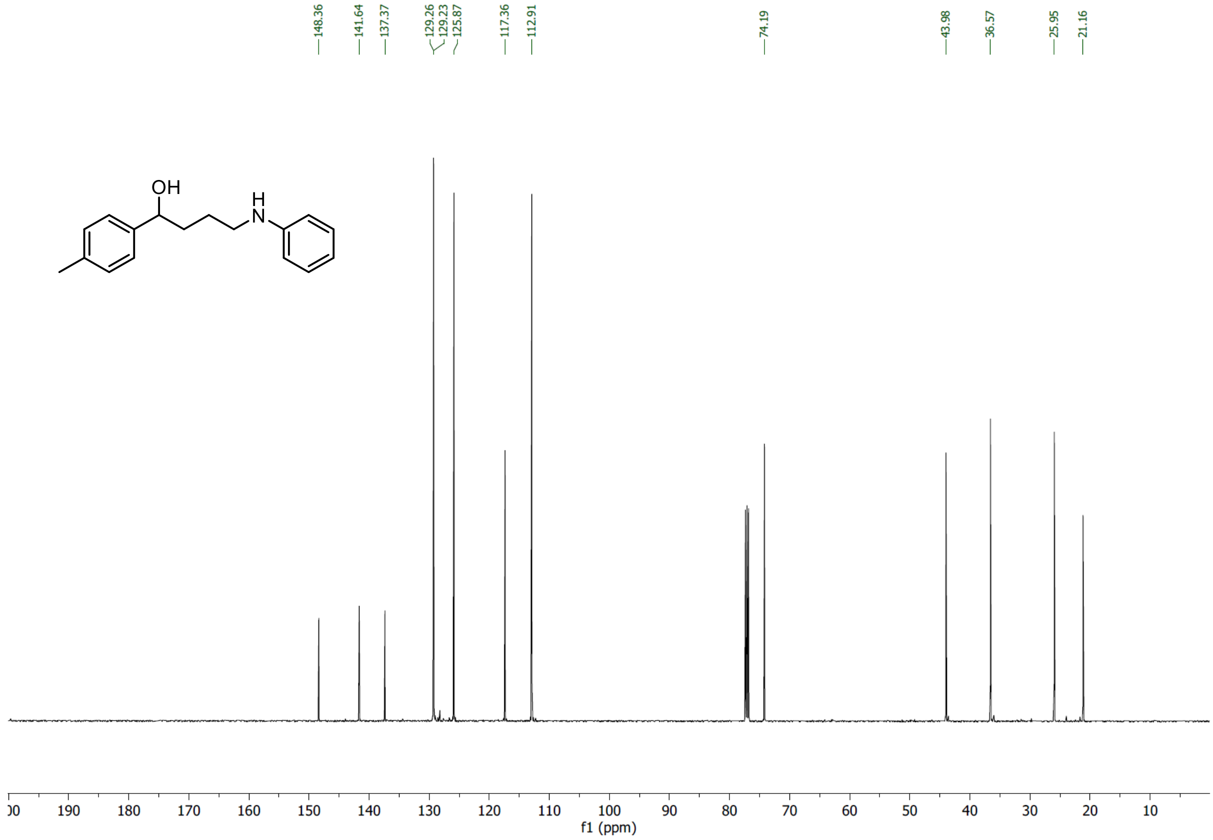

Figure S49. (Top) ^{1}H NMR (500 MHz) and (bottom) $^{13}C\{^{1}H\}$ NMR (126 MHz) spectra of **3la** in $CDCl_3$.

4–(phenylamino)–1–(4–(trifluoromethyl)phenyl)butan–1–ol (3ma)

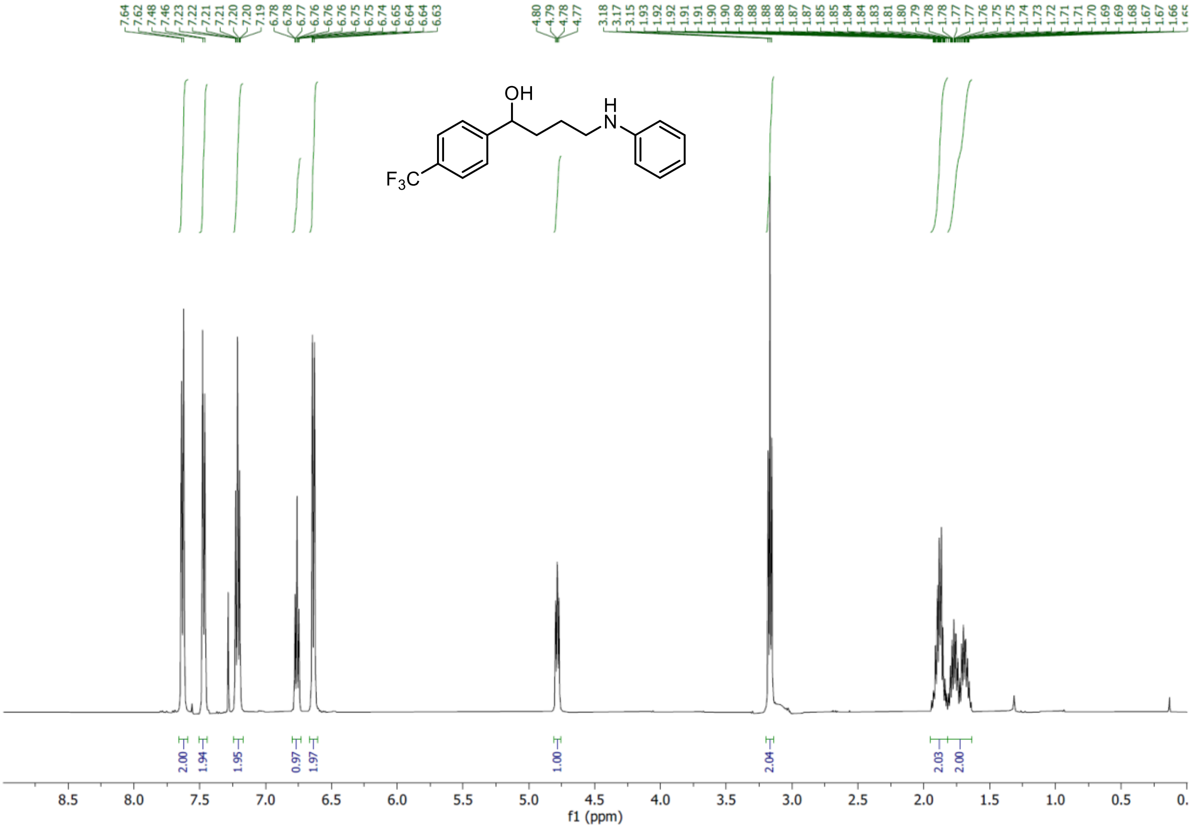

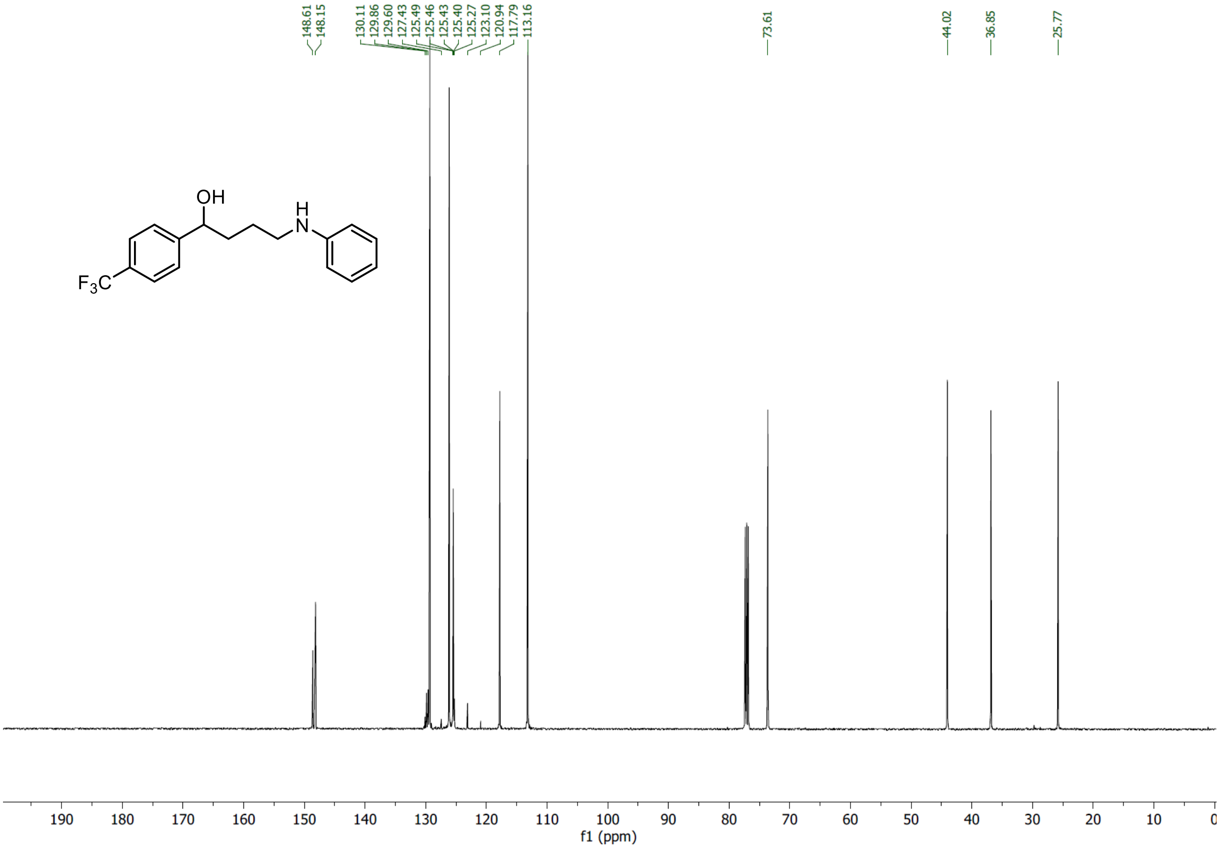

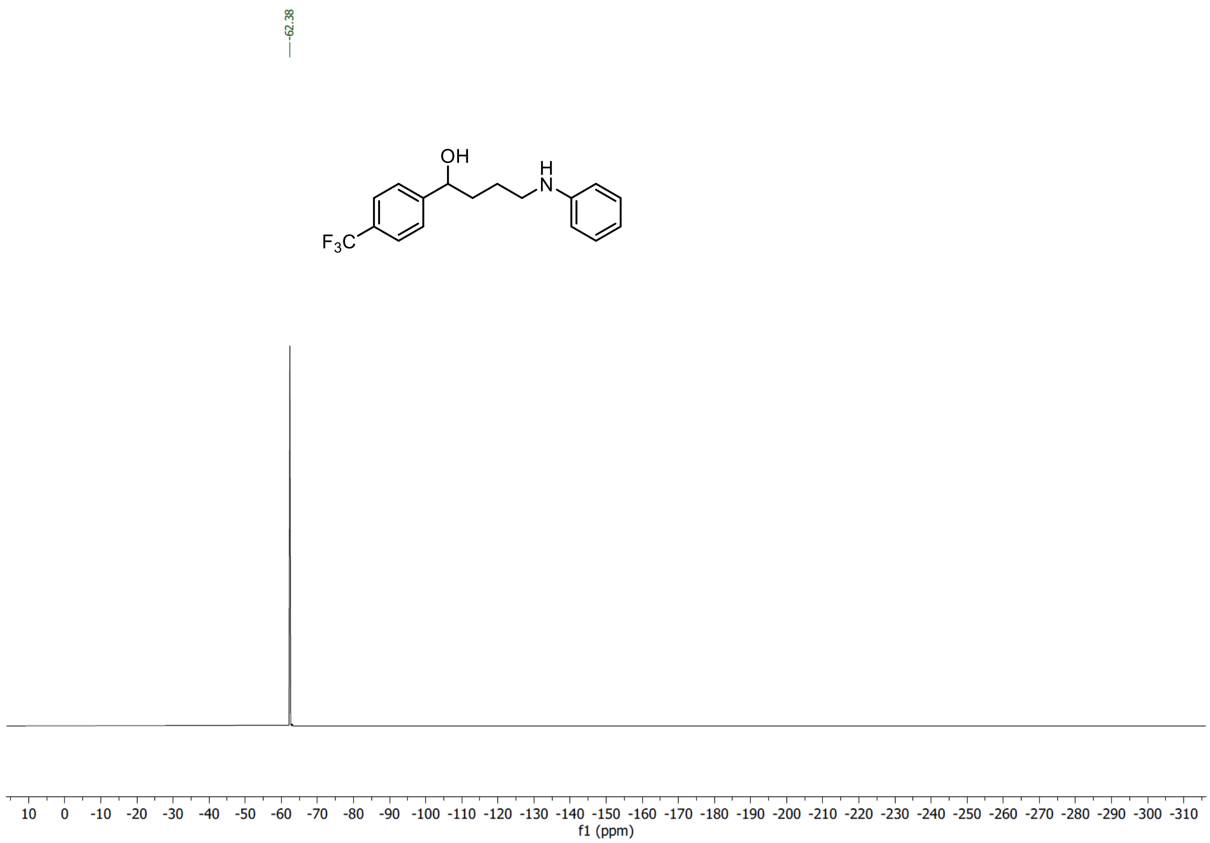

Figure S50. (Top) ^{1}H NMR (500 MHz), (centre) $^{13}C\{^1H\}$ NMR (126 MHz) and (bottom) ^{19}F NMR (470 MHz) spectra of **3ma** in $CDCl_3$.

1-(4-ethylphenyl)-4-(phenylamino)butan-1-ol (3na)

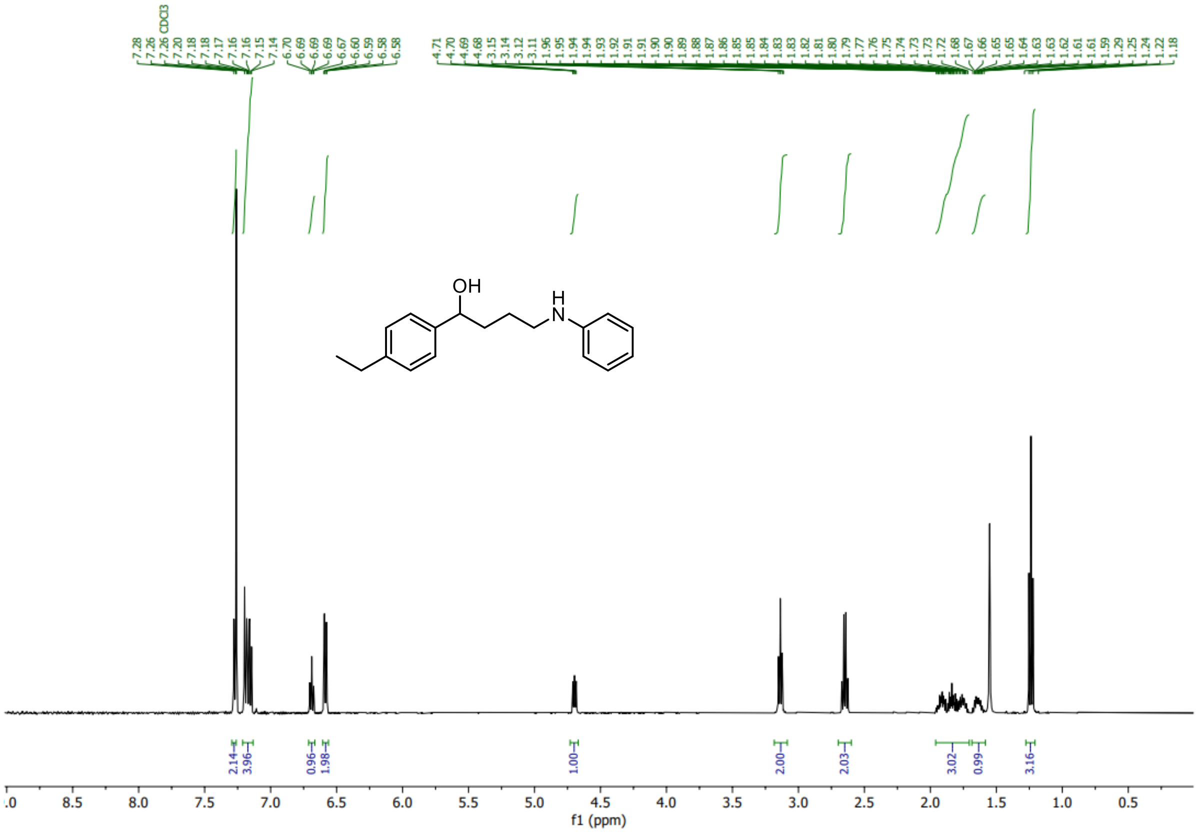

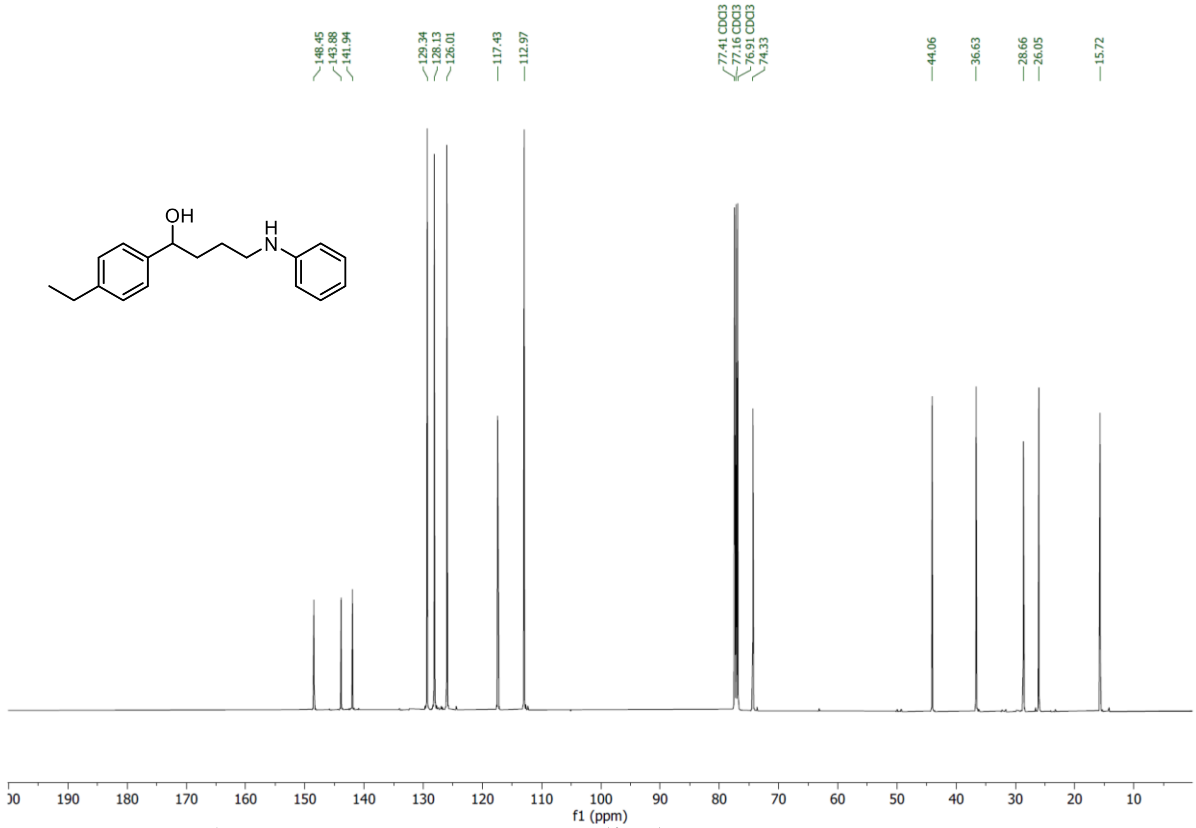

Figure S51. (Top) ^{1}H NMR (500 MHz) and (bottom) ^{13}C{^{1}H} NMR (126 MHz) spectra of **3na** in $CDCl_3$.

1-(6-chloropyridin-3-yl)-4-(phenylamino)butan-1-ol (3oa)

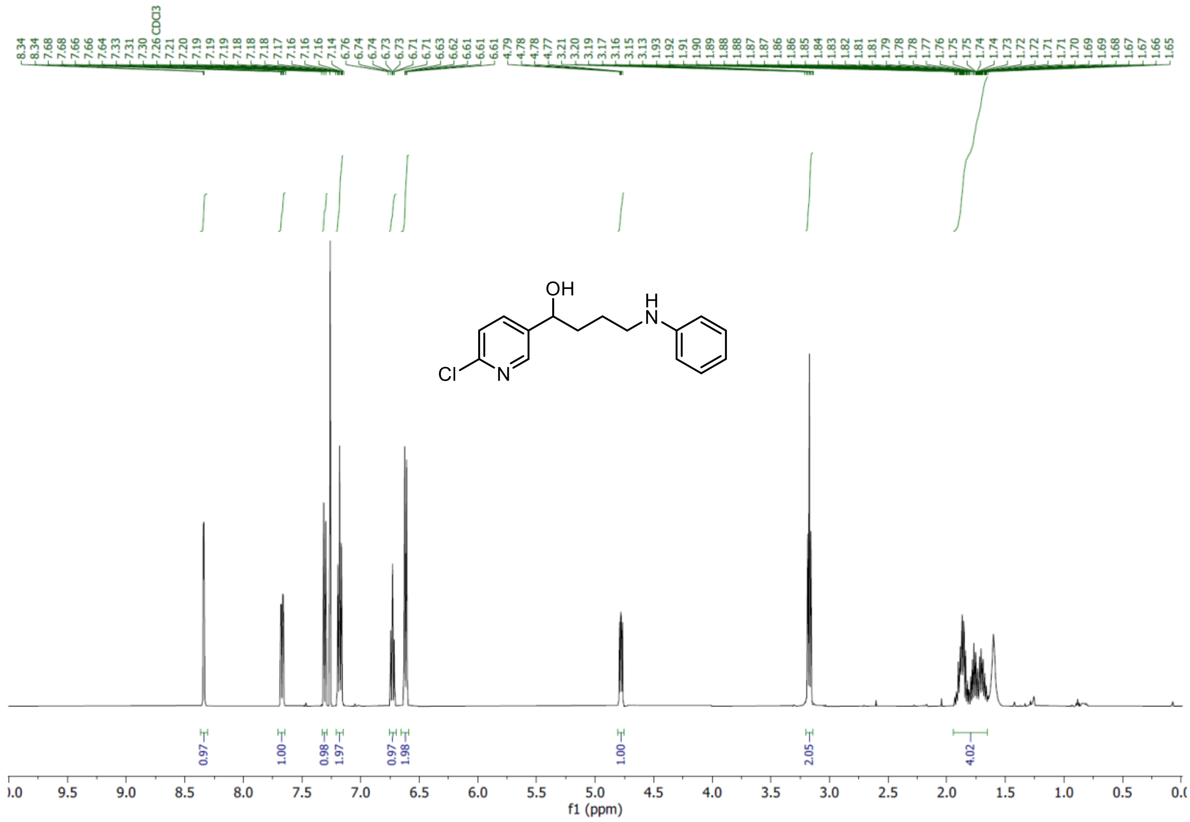

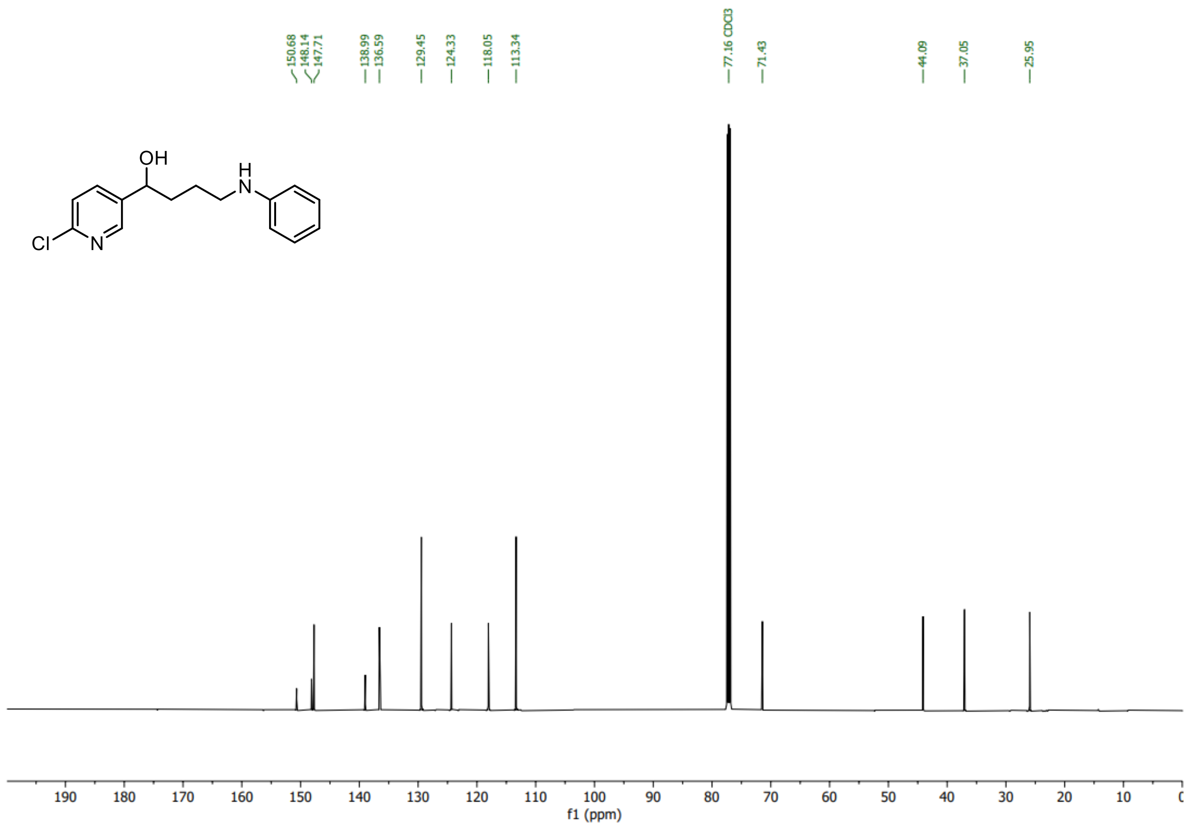

Figure S52. (Top) ^{1}H NMR (500 MHz) and (bottom) $^{13}C\{^{1}H\}$ NMR (126 MHz) spectra of **3oa** in $CDCl_3$.

2-phenyl-5-(phenylamino)pentan-2-ol (3pa)

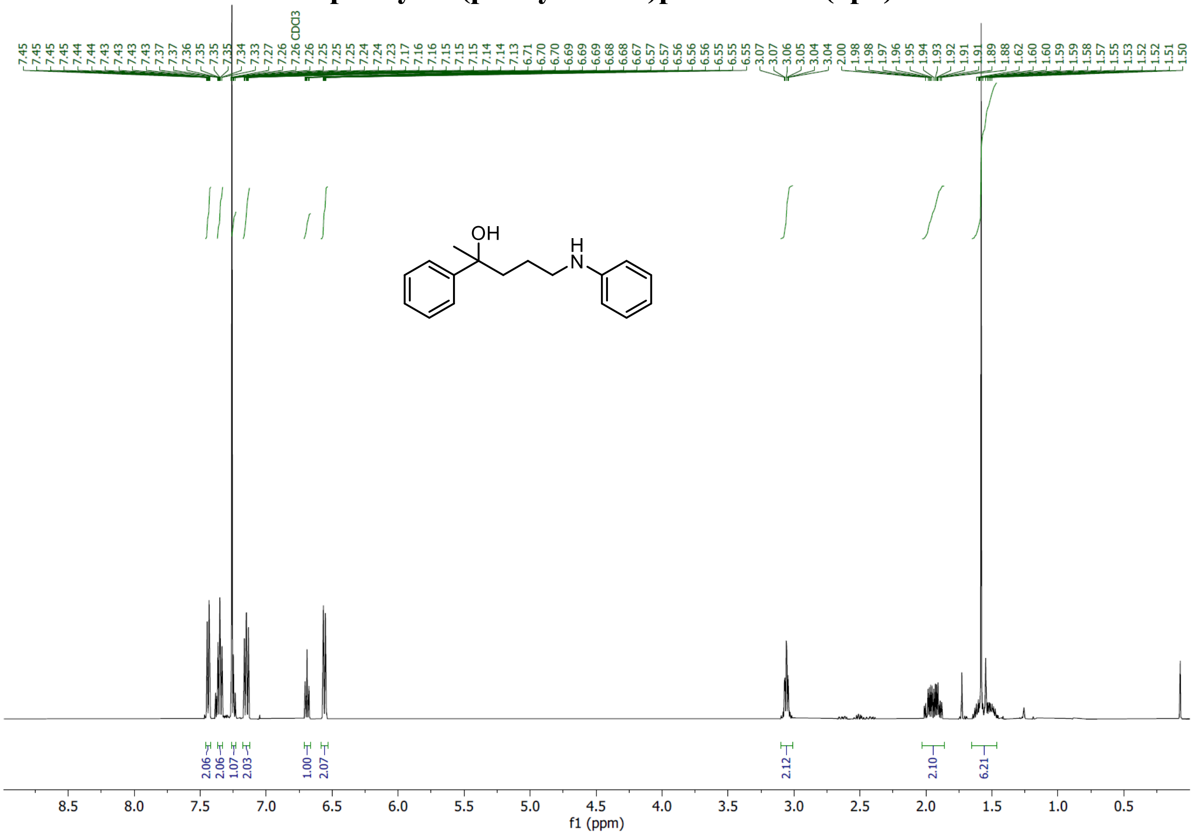

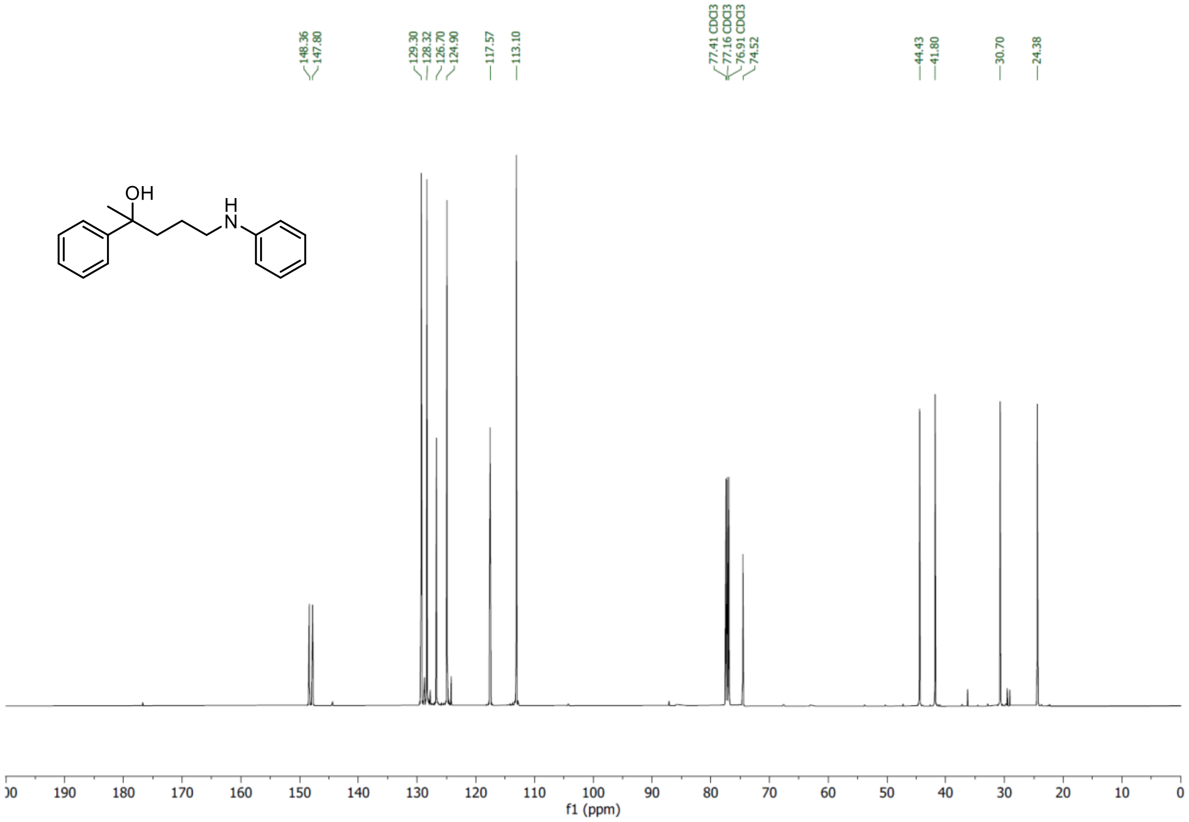

Figure S53. (Top) ^{1}H NMR (500 MHz) and (bottom) $^{13}C\{^{1}H\}$ NMR (126 MHz) spectra of **3pa** in $CDCl_3$.

1-cyclopentyl-4-(phenylamino)butan-1-ol (3qa)

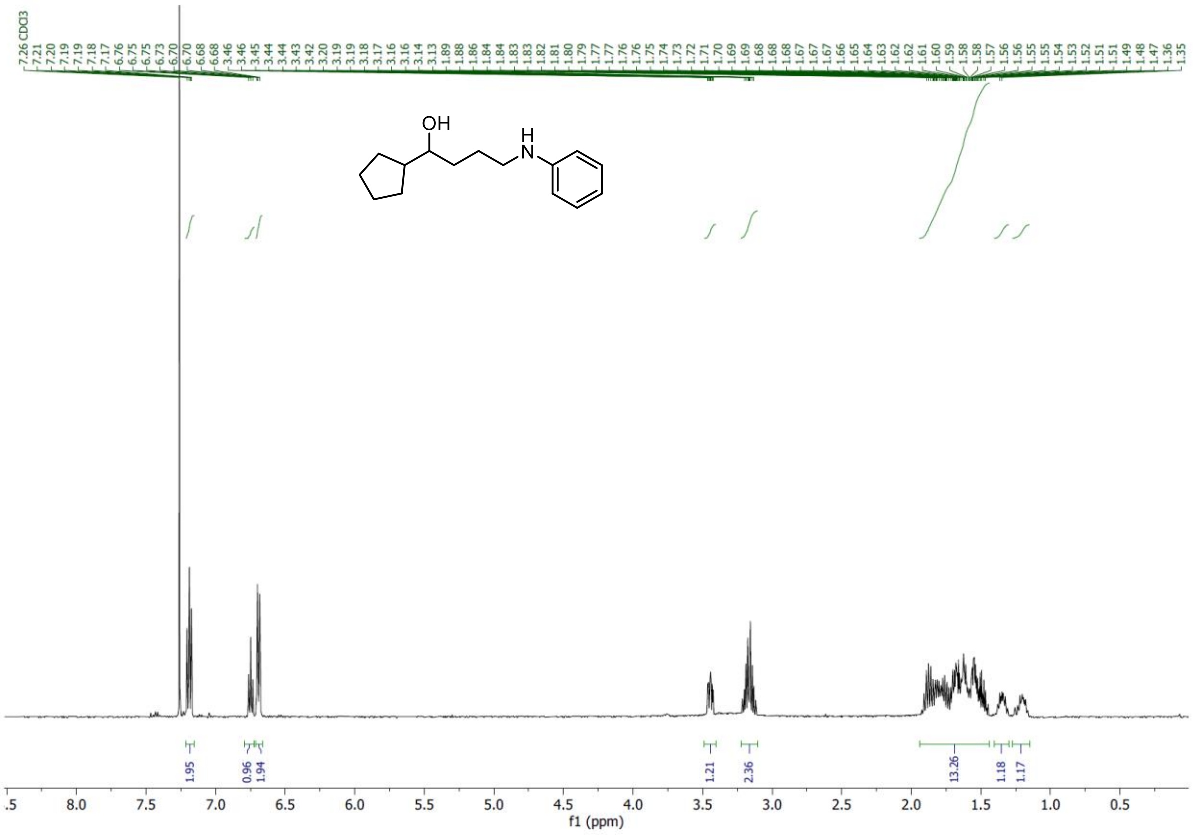

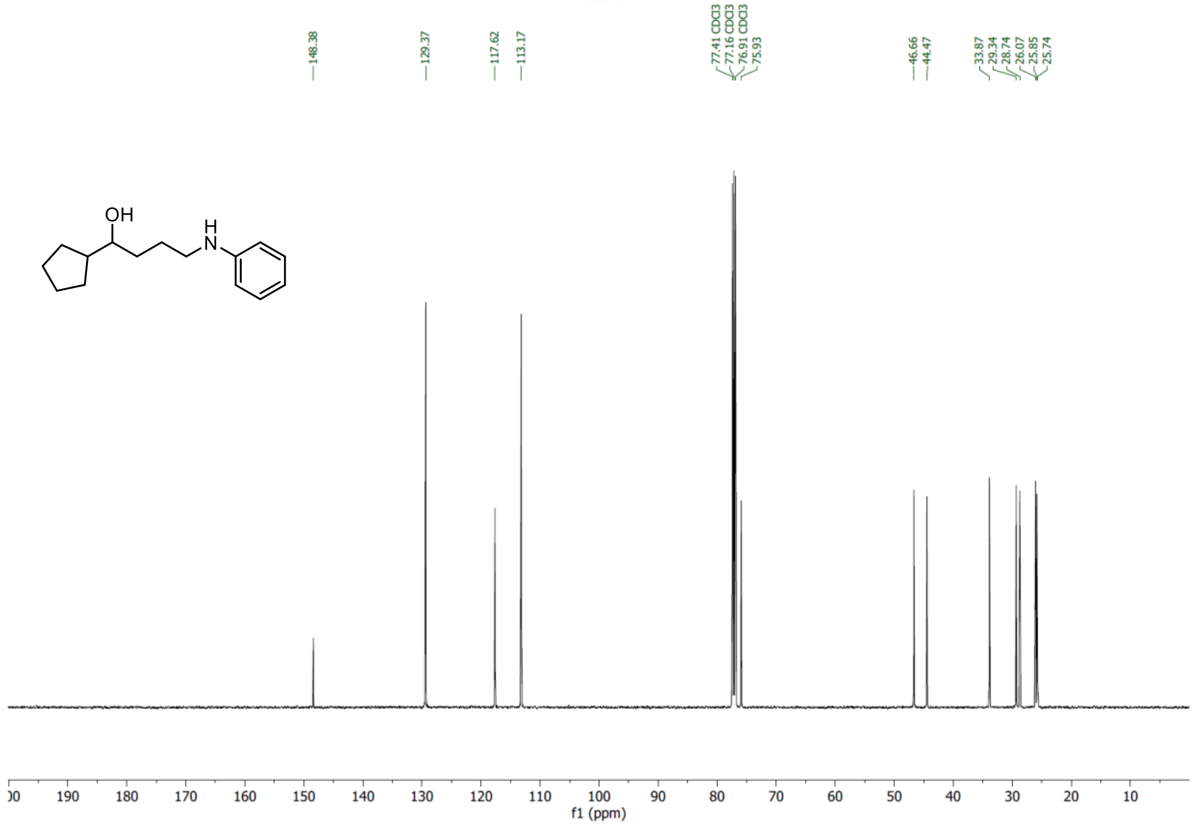

Figure S54. (Top) ^{1}H NMR (500 MHz) and (bottom) $^{13}C\{^{1}H\}$ NMR (126 MHz) spectra of **3qa** in $CDCl_3$.

(1s,3r,5R,7S)-3-((phenylamino)methyl)adamantan-1-ol (3ra)

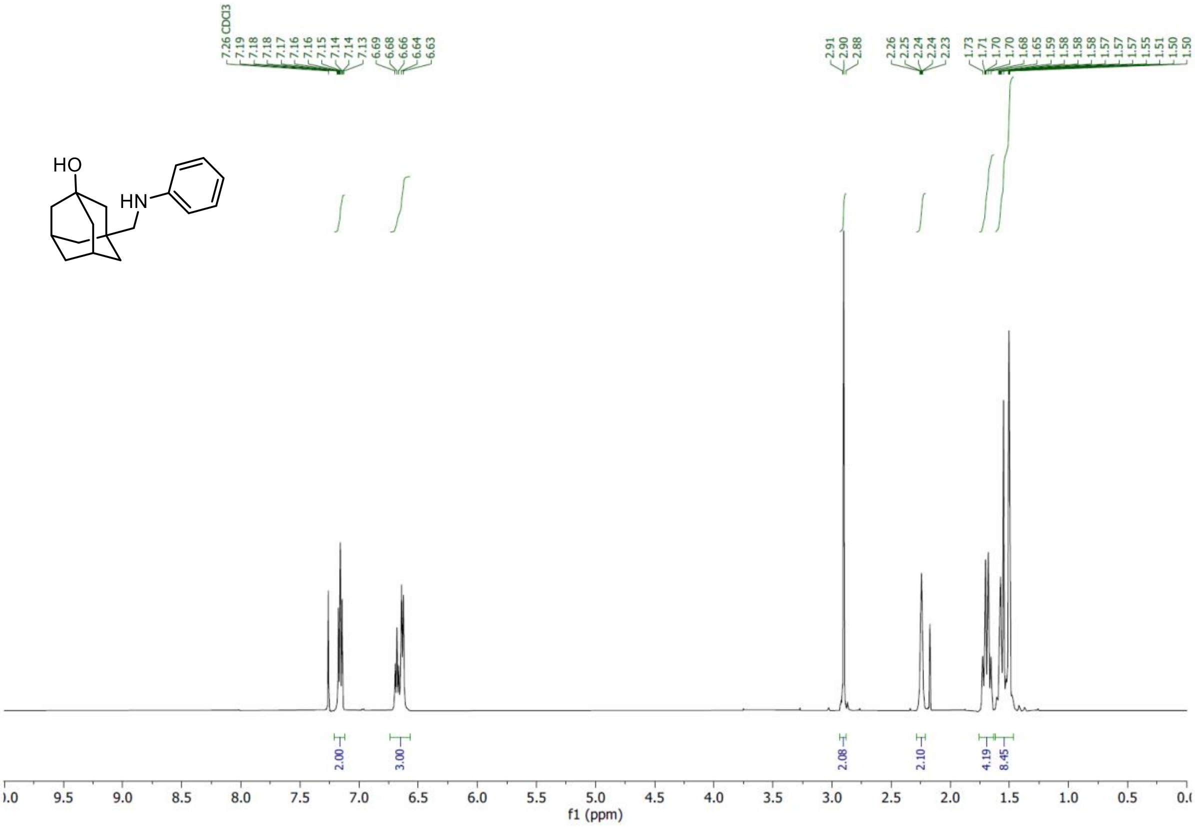

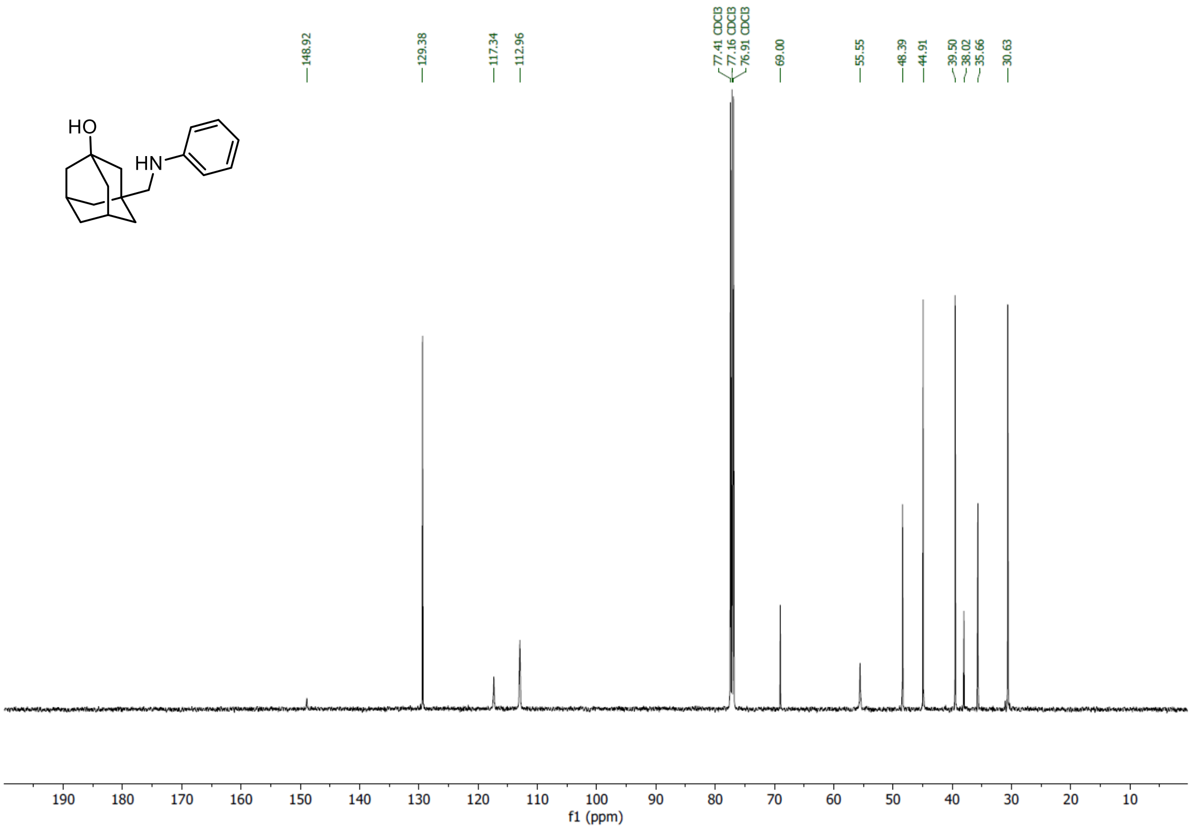

Figure S55. (Top) ^{1}H NMR (500 MHz) and (bottom) $^{13}C\{^{1}H\}$ NMR (126 MHz) spectra of **3ra** in $CDCl_3$.

1-phenyl-5-(phenylamino)pentan-1-ol (3sa)

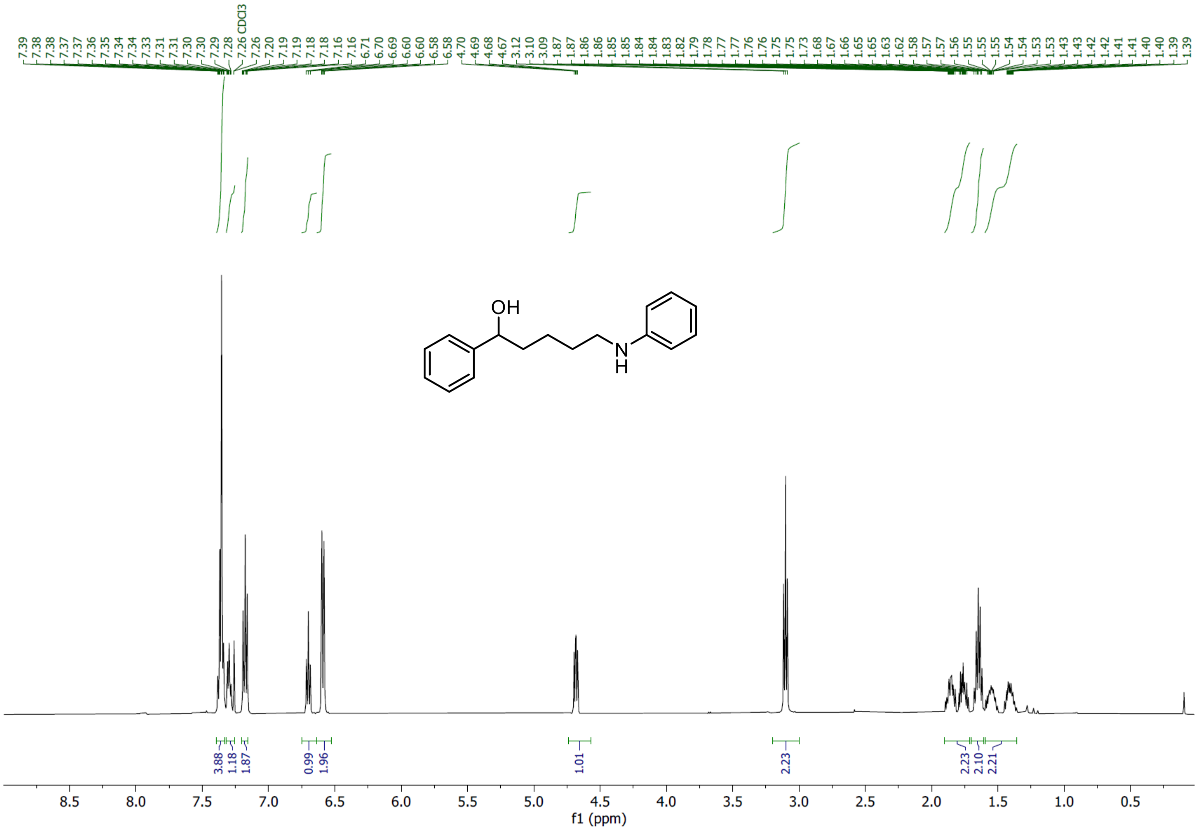

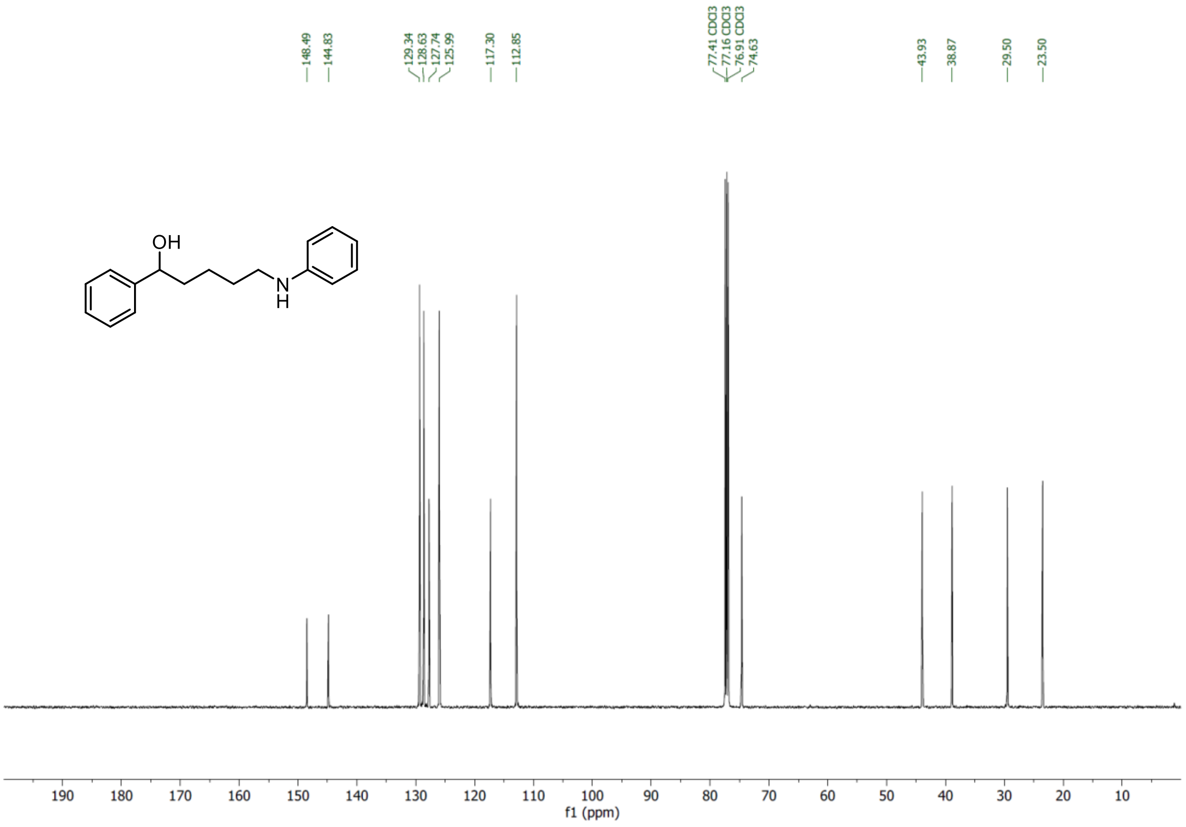

Figure S56. (Top) ^{1}H NMR (500 MHz) and (bottom) $^{13}C\{^{1}H\}$ NMR (126 MHz) spectra of **3sa** in $CDCl_3$.

5-(phenylamino)pentan-2-ol (3ta)

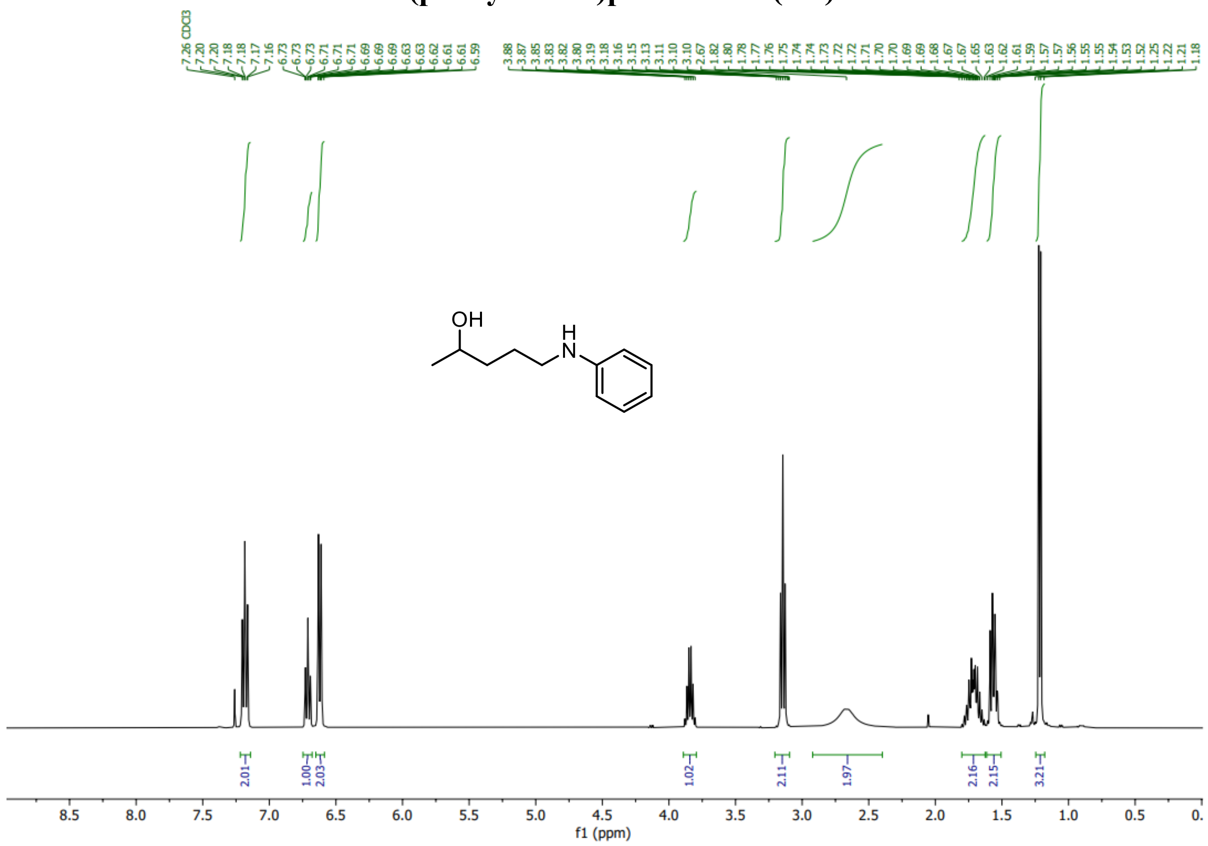

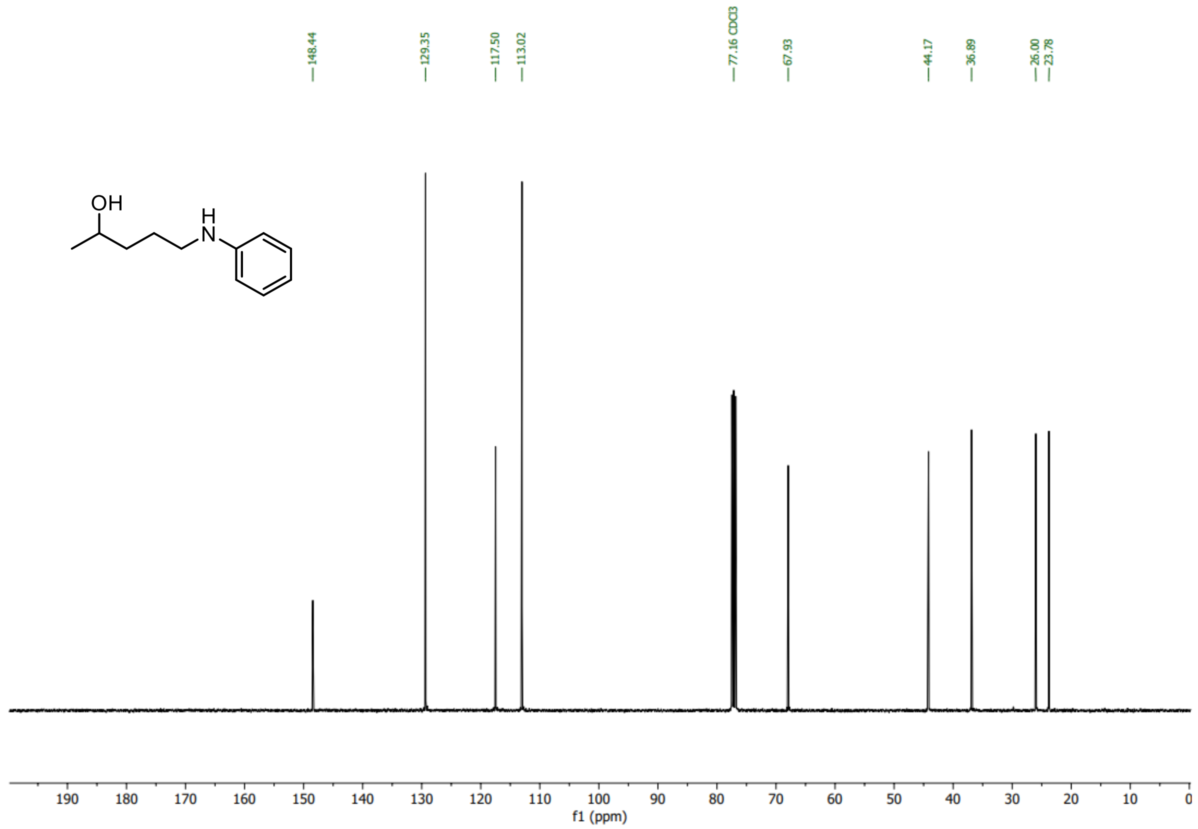

Figure S57. (Top) ^{1}H NMR (400 MHz) and (bottom) ^{13}C{^{1}H} NMR (101 MHz) spectra of **3ta** in $CDCl_3$.

1-(phenylamino)pentadecan-4-ol (3ua)

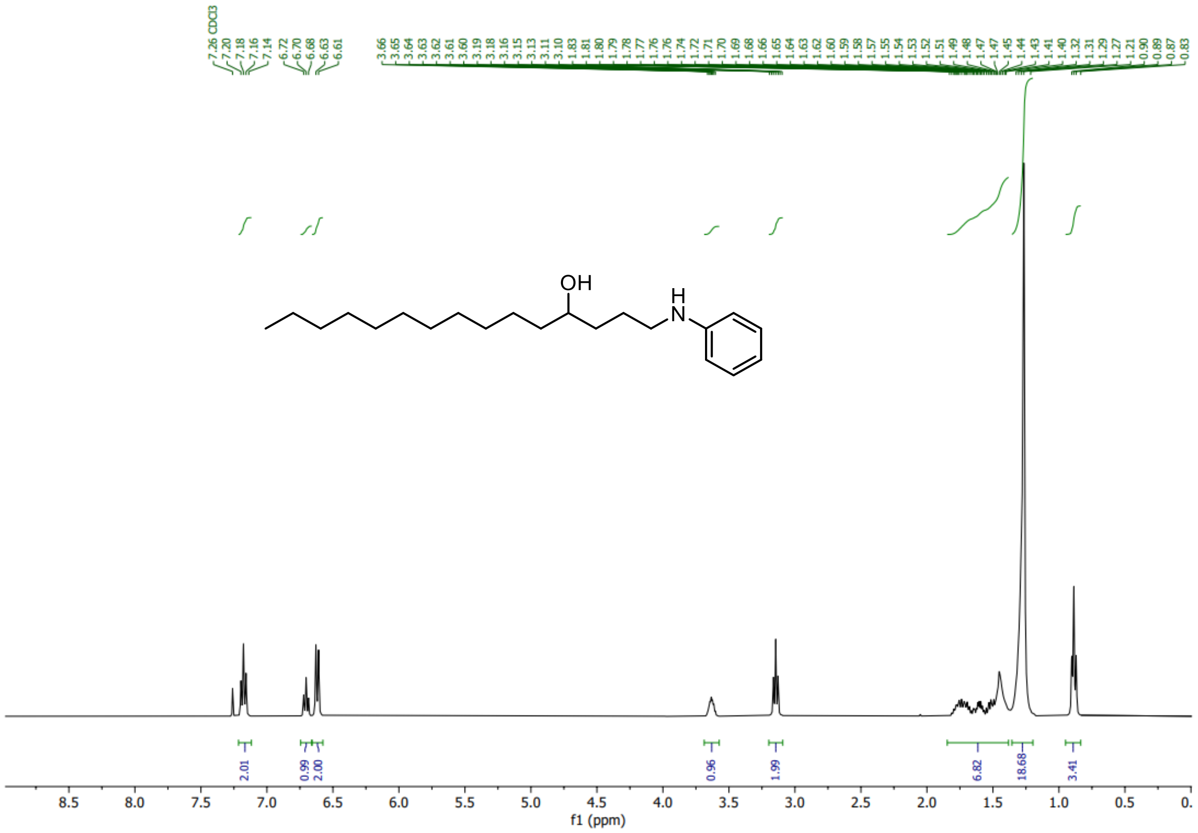

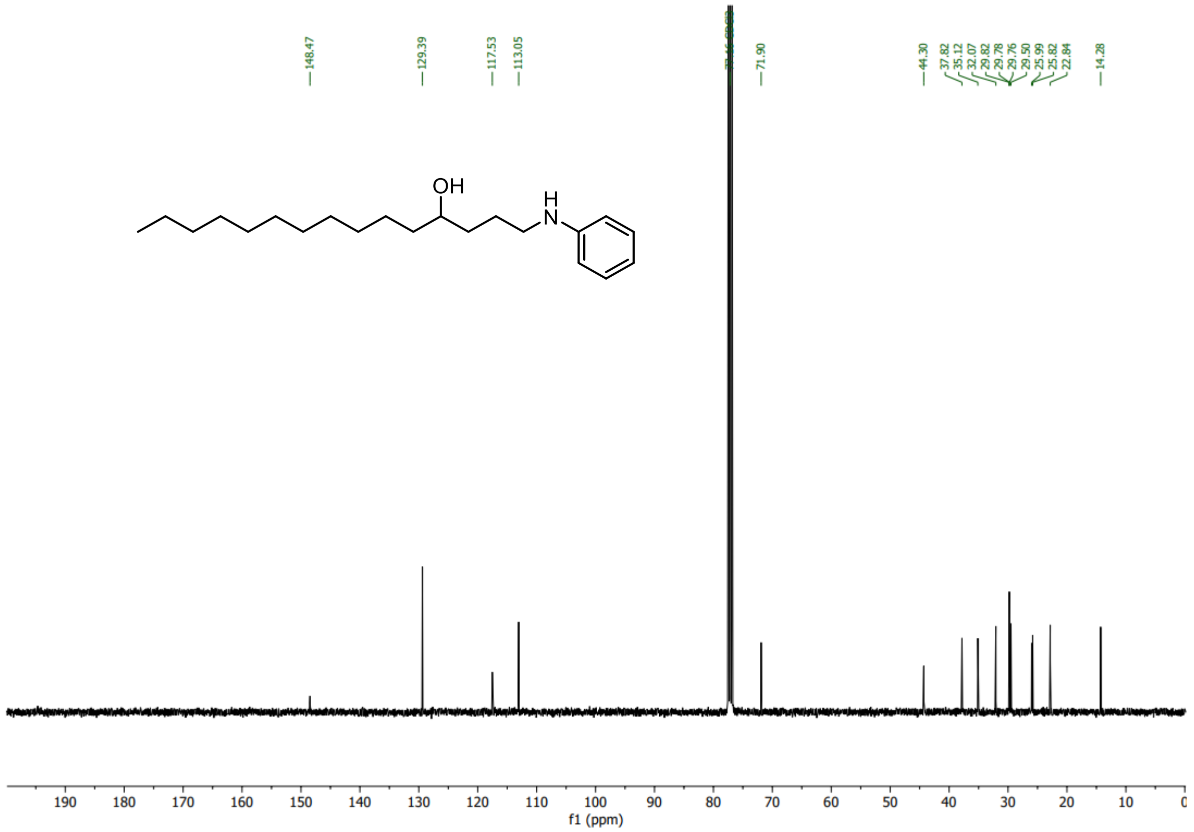

Figure S58. (Top) ^{1}H NMR (400 MHz) and (bottom) $^{13}C\{^{1}H\}$ NMR (101 MHz) spectra of **3ua** in $CDCl_3$.

(*S*)-1-phenyl-4-(phenylamino)butan-1-ol [(*S*)-3aa]

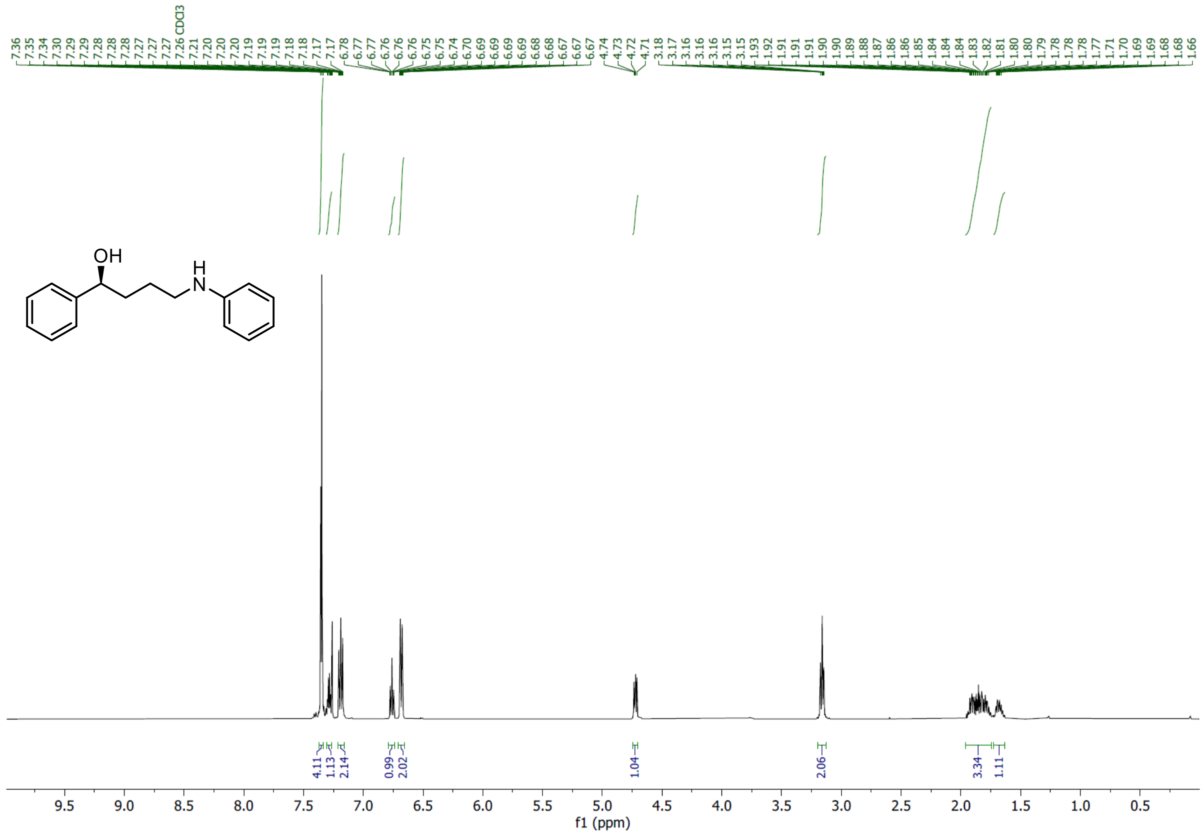

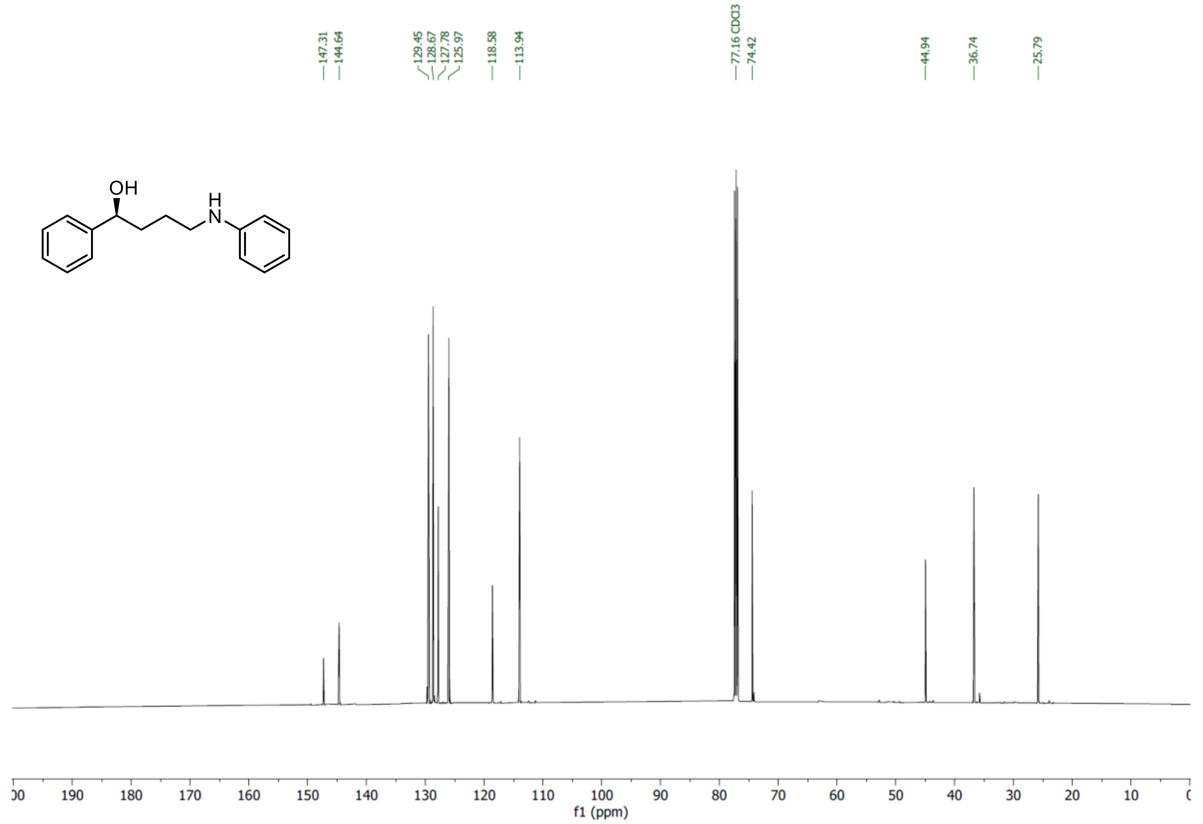

Figure S59. (Top) ^{1}H NMR (500 MHz) and (bottom) ^{13}C{^{1}H} NMR (126 MHz) spectra of **(*S*)-3aa** in $CDCl_3$.

(*S*)-1-(2-chlorophenyl)-4-(phenylamino)butan-1-ol [(*S*)-3ba]

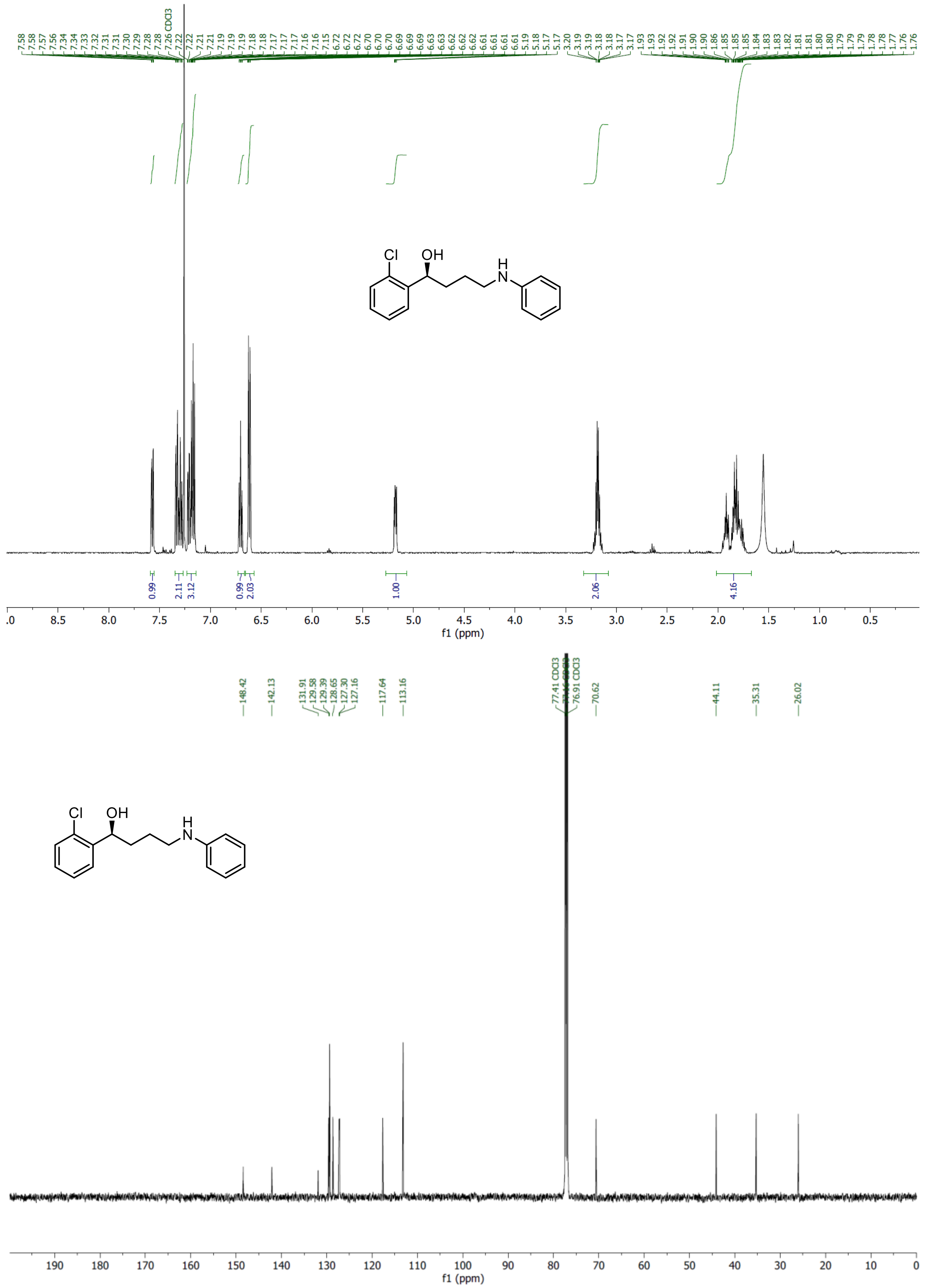

Figure S60. (Top) ^{1}H NMR (500 MHz) and (bottom) ^{13}C{^{1}H} NMR (126 MHz) spectra of **(*S*)-3ba** in $CDCl_3$.

(*S*)-4-(phenylamino)-1-(m-tolyl)butan-1-ol [(*S*)-3ga]

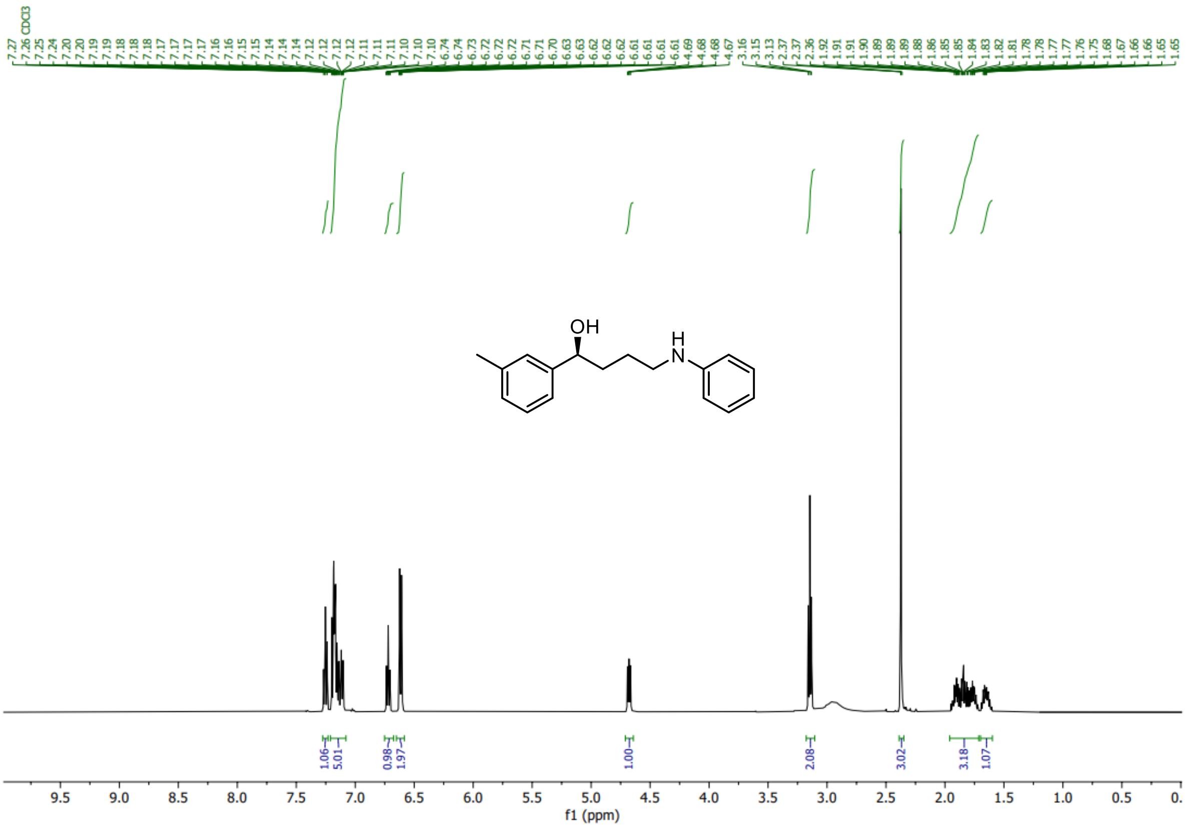

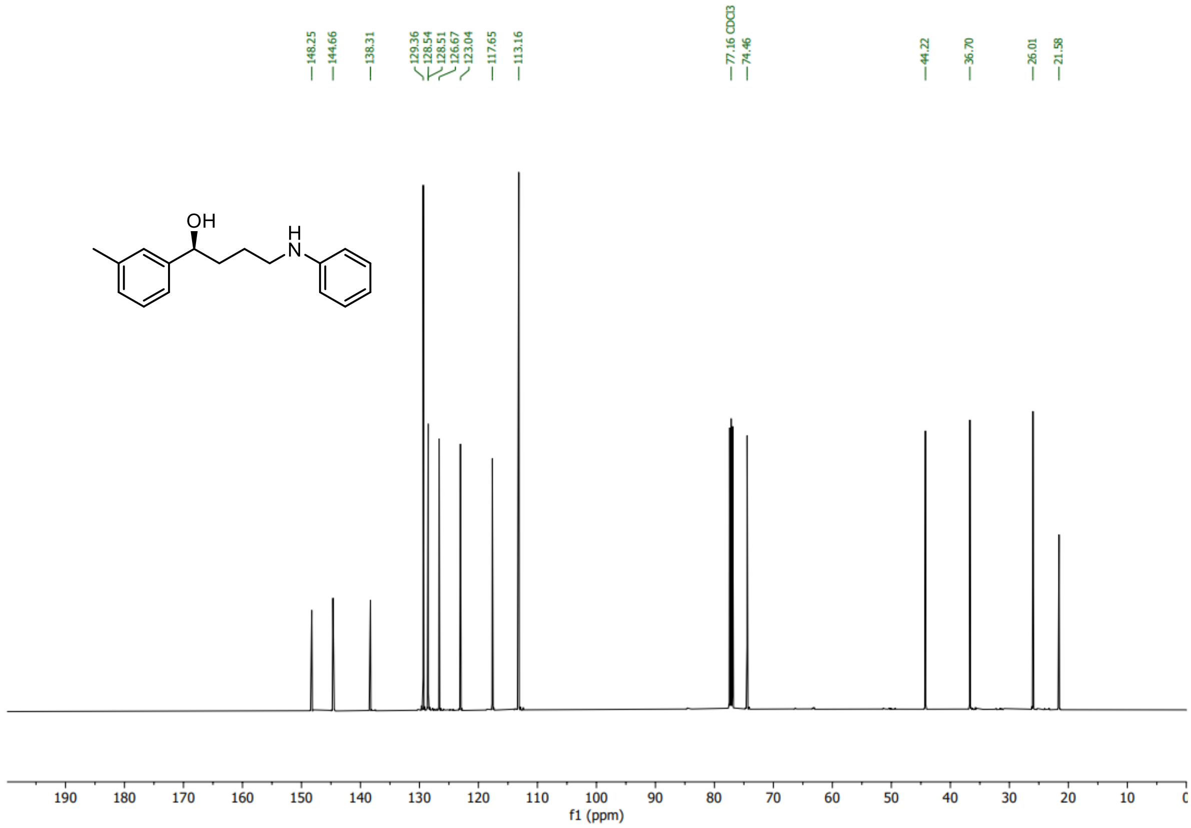

Figure S61. (Top) ^{1}H NMR (500 MHz) and (bottom) $^{13}C\{^{1}H\}$ NMR (126 MHz) spectra of **(*S*)-3ga** in $CDCl_3$.

(*S*)-1-(4-fluorophenyl)-4-(phenylamino)butan-1-ol [(*S*)-3ia]

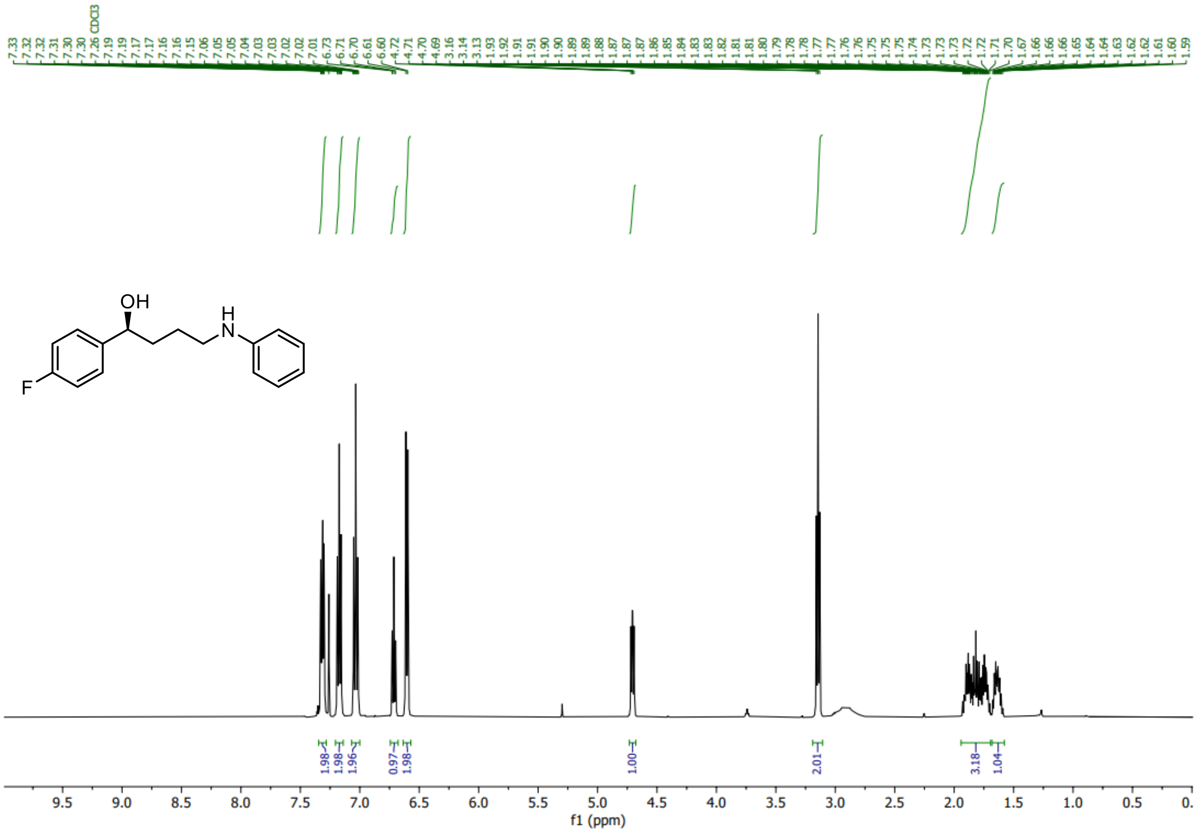

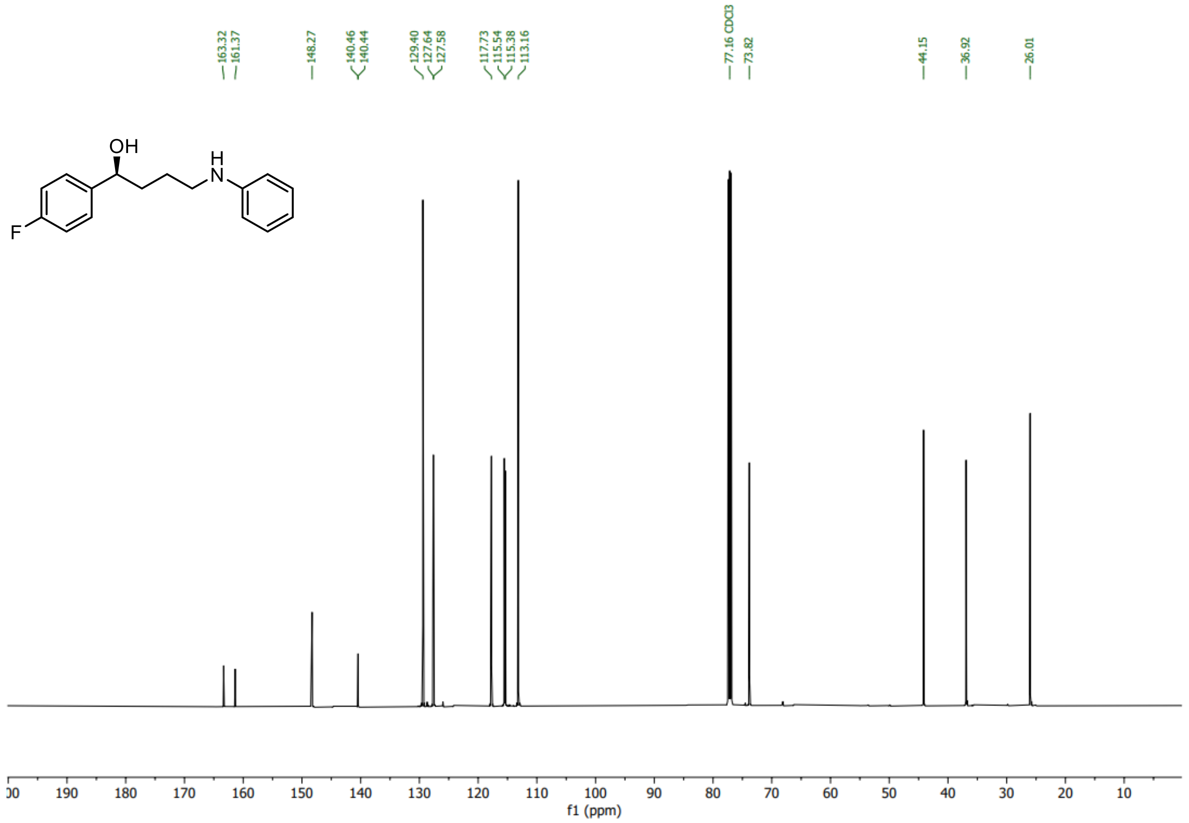

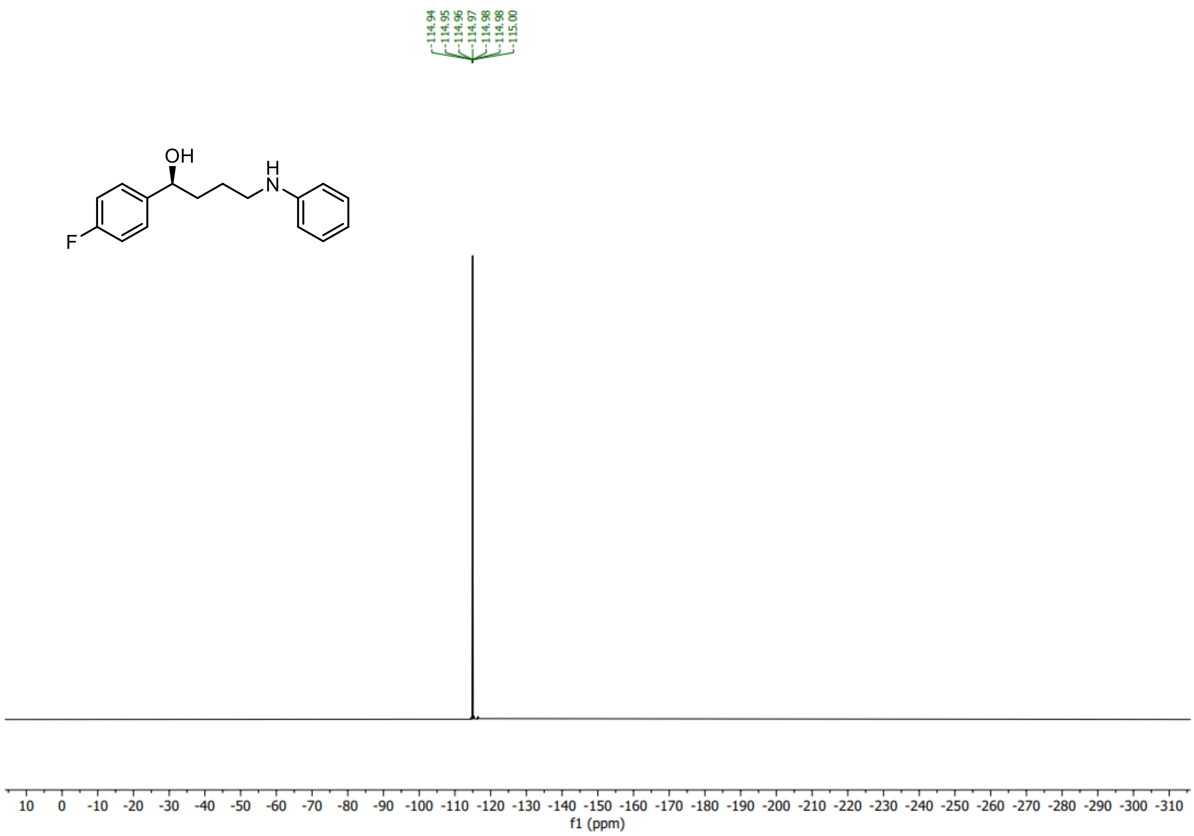

Figure S62. (Top) ^{1}H NMR (500 MHz), (centre) $^{13}C\{^{1}H\}$ NMR (126 MHz) and (bottom) ^{19}F NMR (470 MHz) spectra of ***(S)*-3ia** in $CDCl_3$

(*S*)-1-(4-chlorophenyl)-4-(phenylamino)butan-1-ol [(*S*)-3ja]

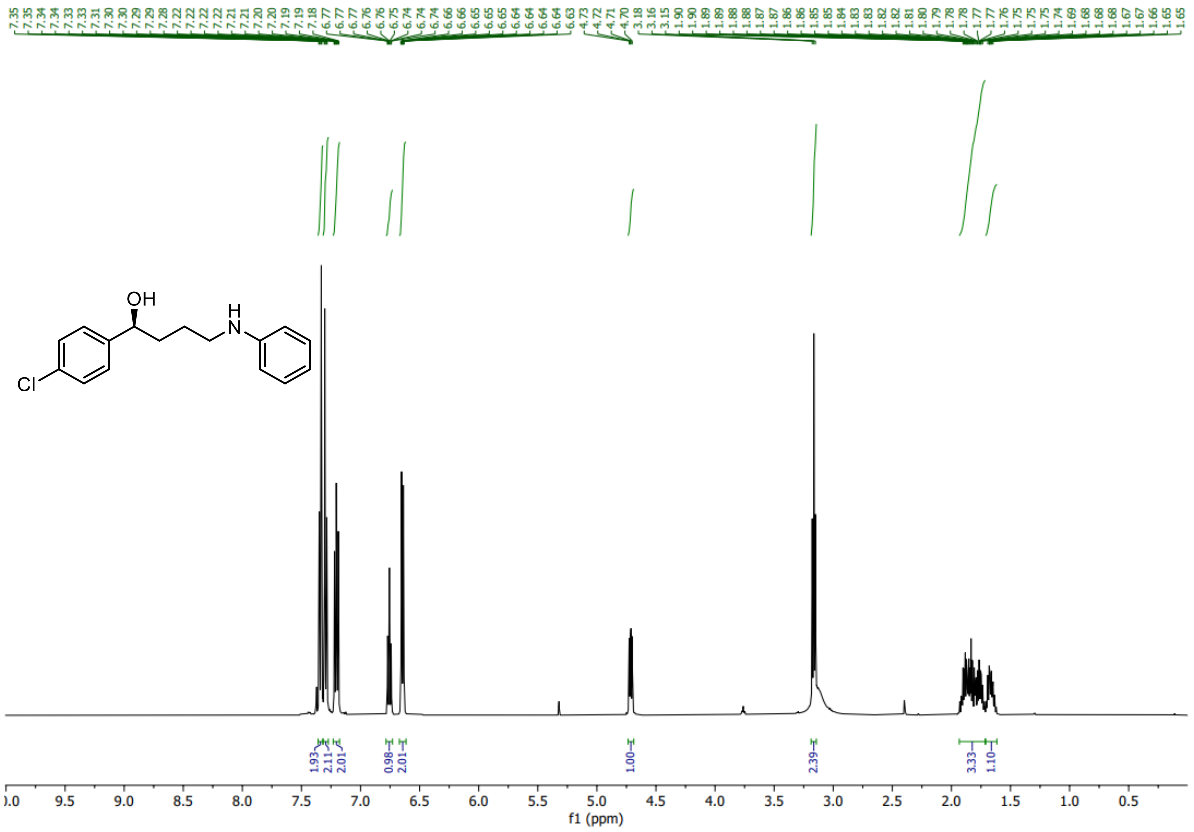

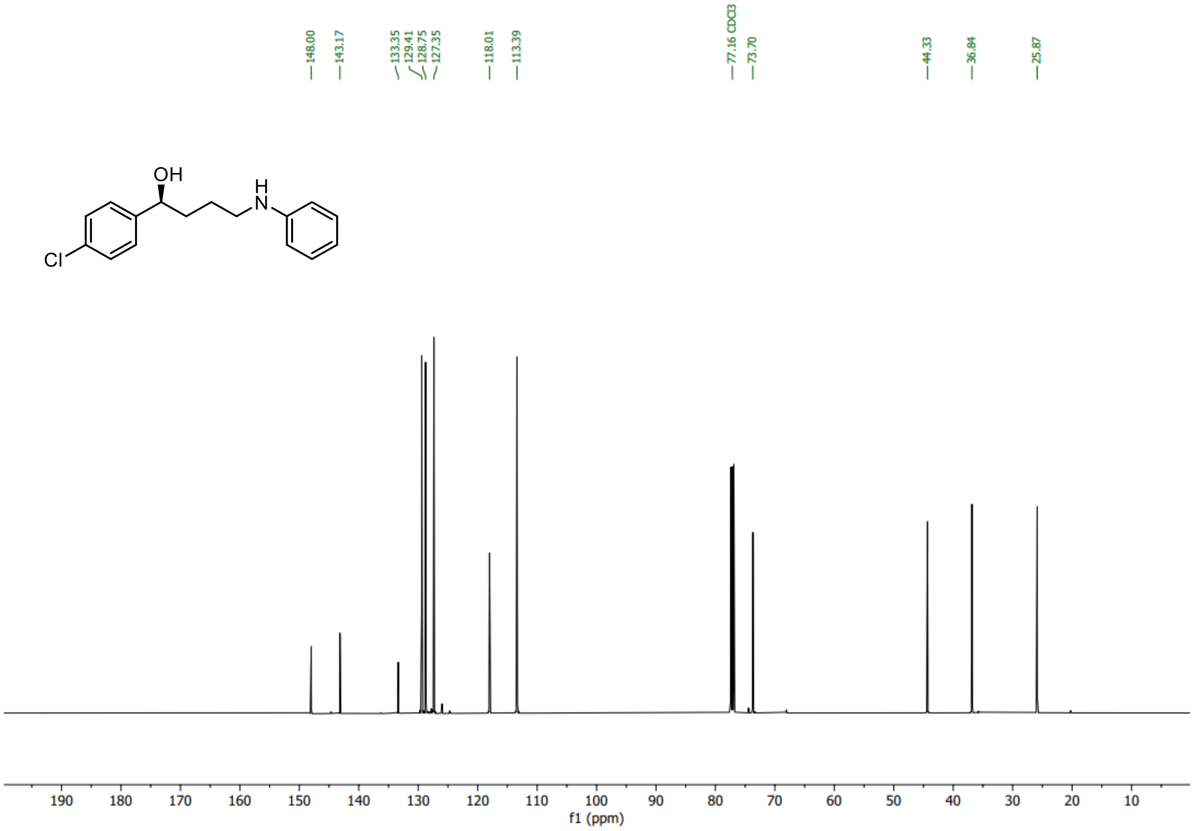

Figure S63. (Top) ^{1}H NMR (500 MHz) and (bottom) ^{13}C{^{1}H} NMR (126 MHz) spectra of **(*S*)-3ja** in $CDCl_3$.

(*S*)-1-(4-bromophenyl)-4-(phenylamino)butan-1-ol [(*S*)-3ka]

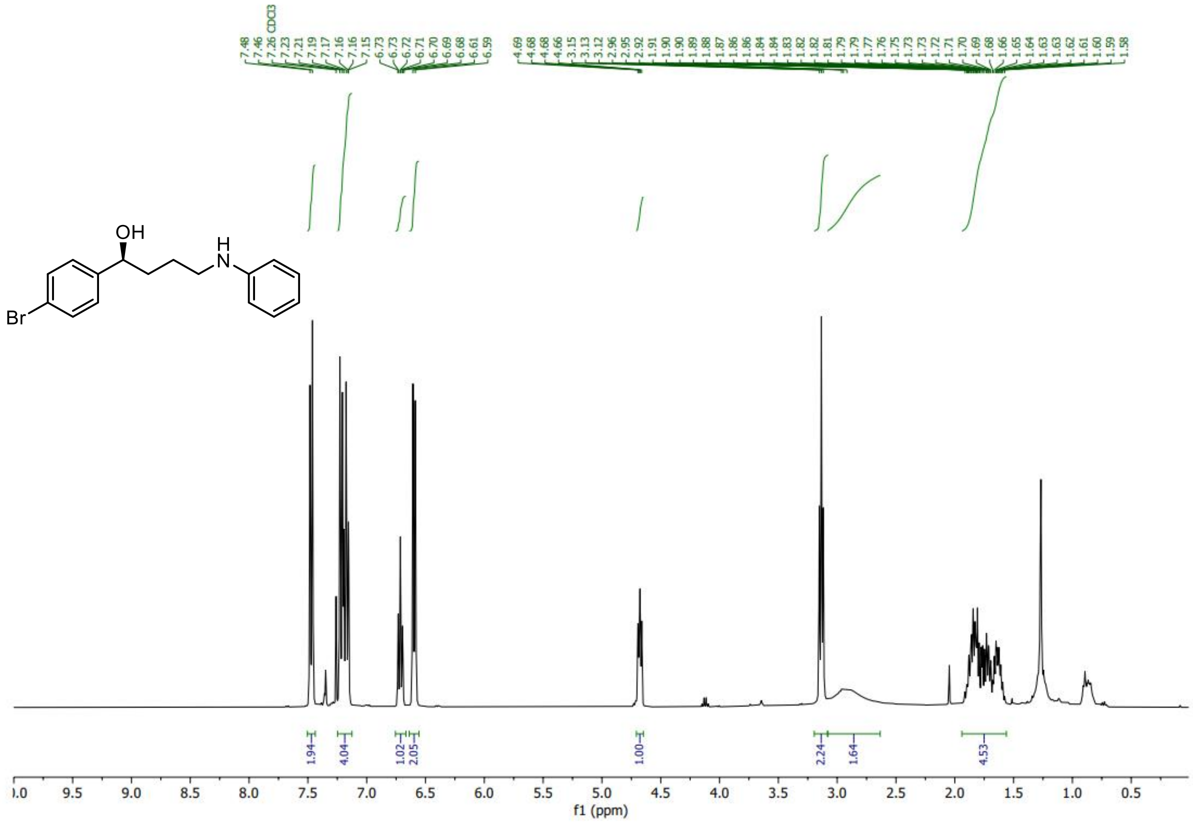

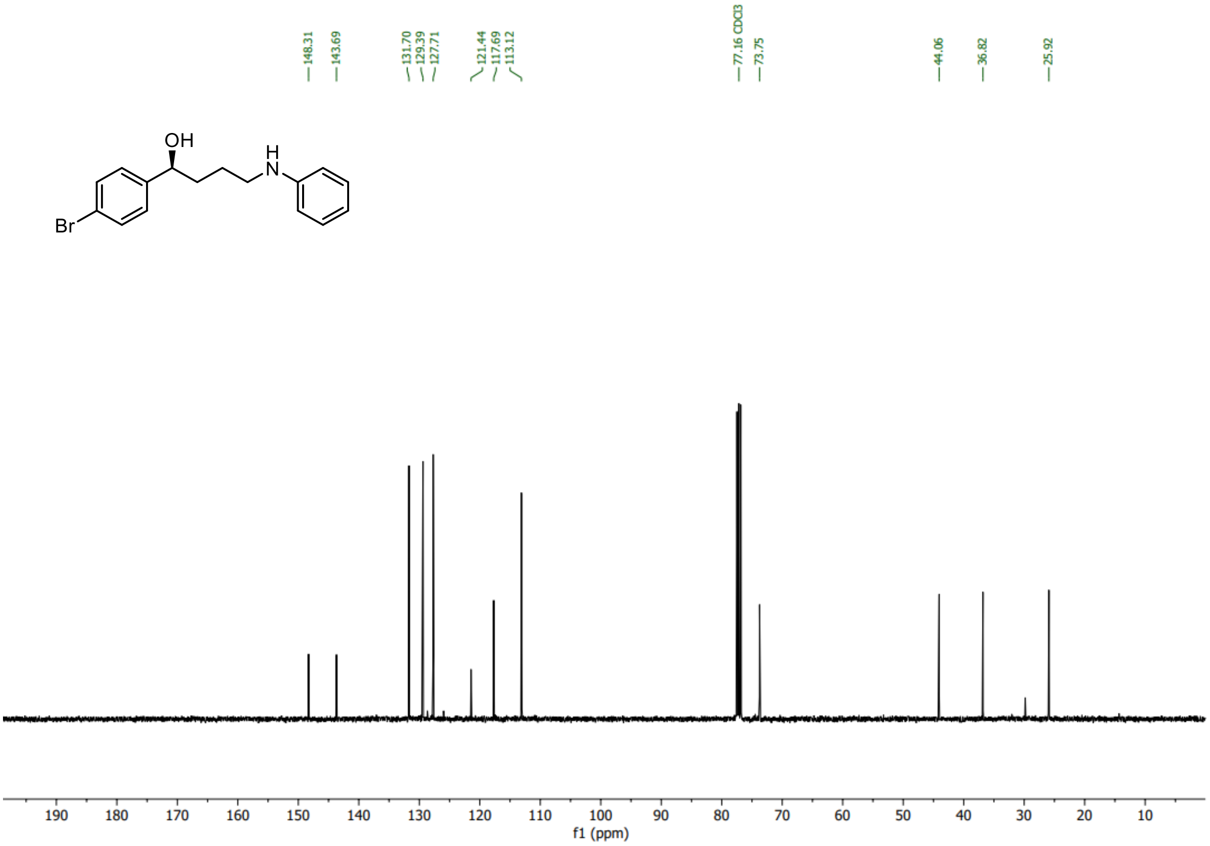

Figure S64. (Top) ^{1}H NMR (400 MHz) and (bottom) $^{13}C\{^{1}H\}$ NMR (101 MHz) spectra of **(*S*)-3ka** in $CDCl_3$.

(*S*)-1-(4-ethylphenyl)-4-(phenylamino)butan-1-ol [(*S*)-3na]

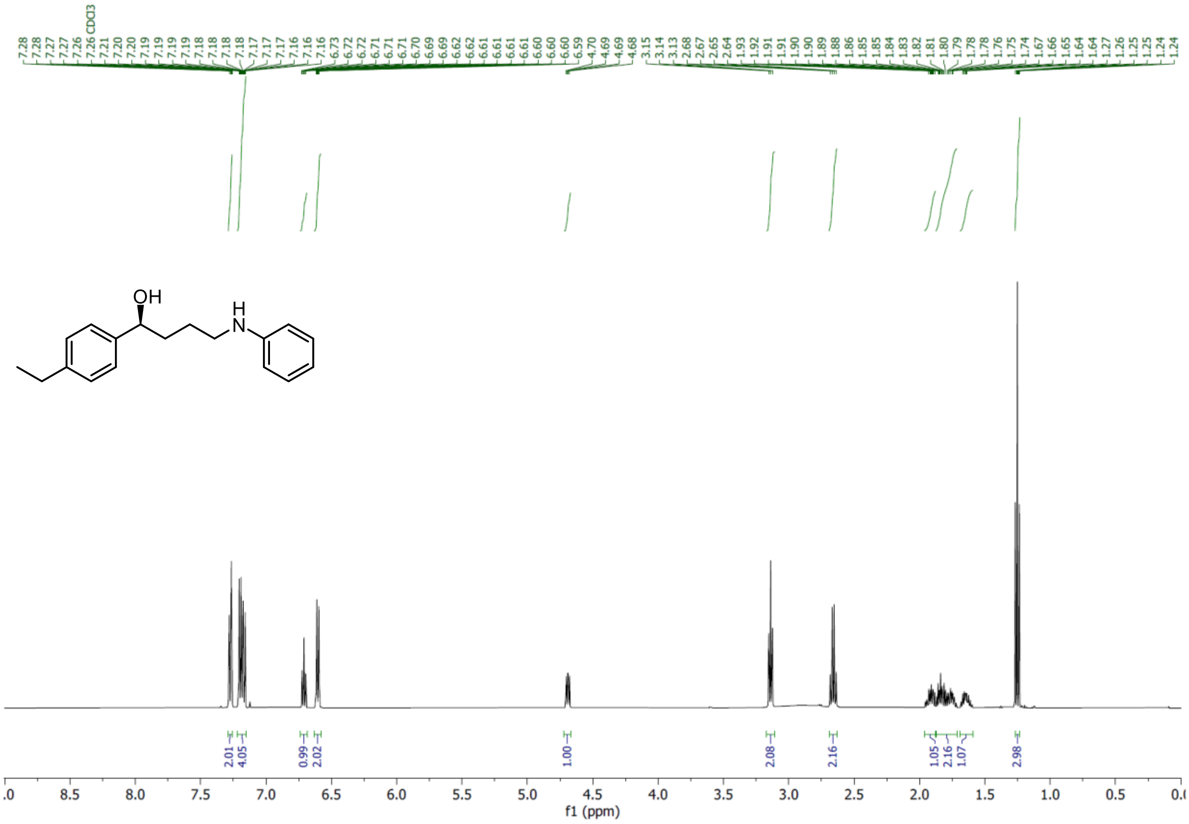

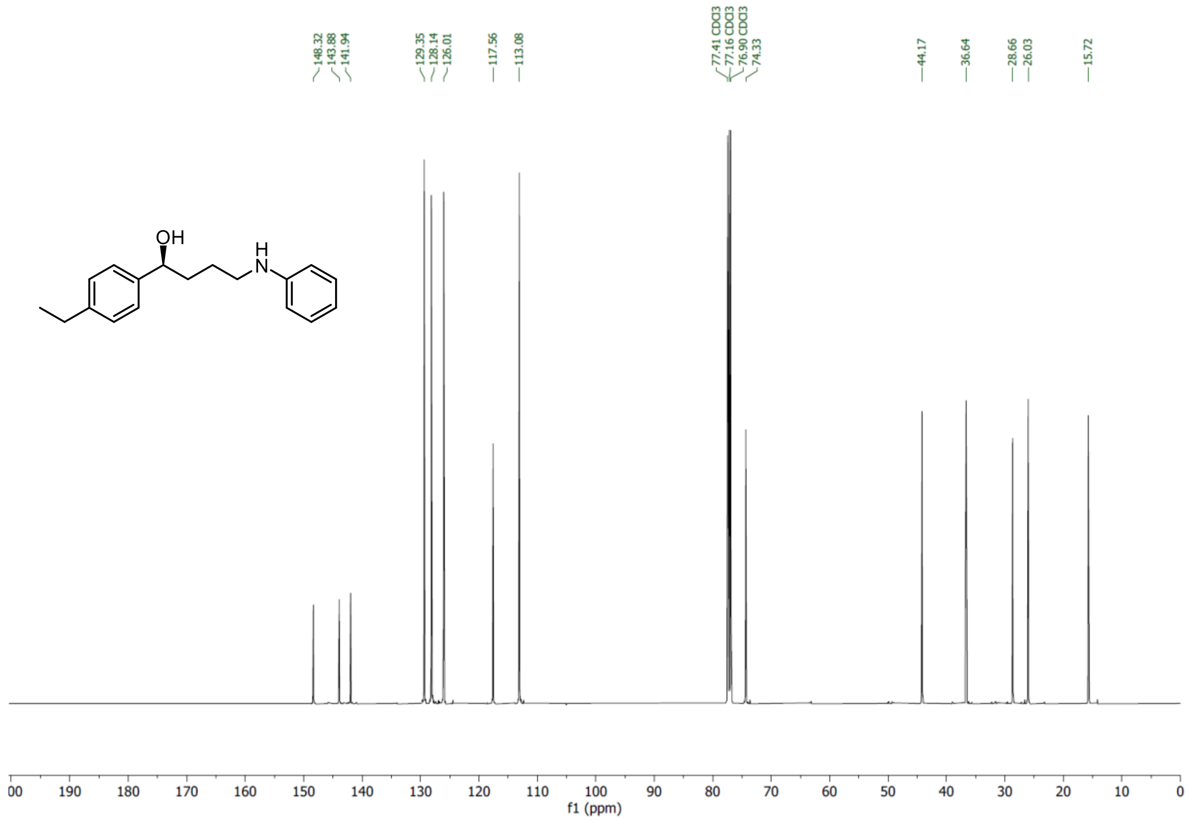

Figure S65. (Top) ^{1}H NMR (500 MHz) and (bottom) ^{13}C{^{1}H} NMR (126 MHz) spectra of **(*S*)-3na** in $CDCl_3$.

1–phenyl–4–(phenylamino)butan–3–*D*–1–ol [3aa–d(1)]

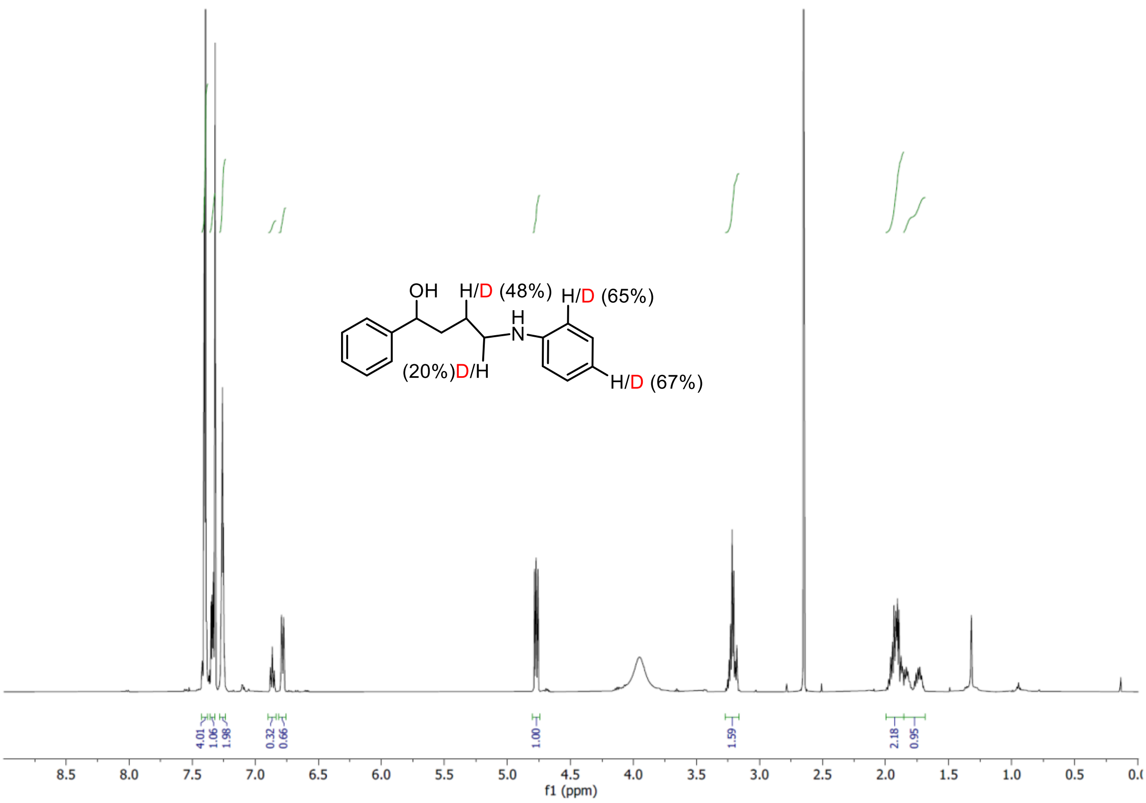

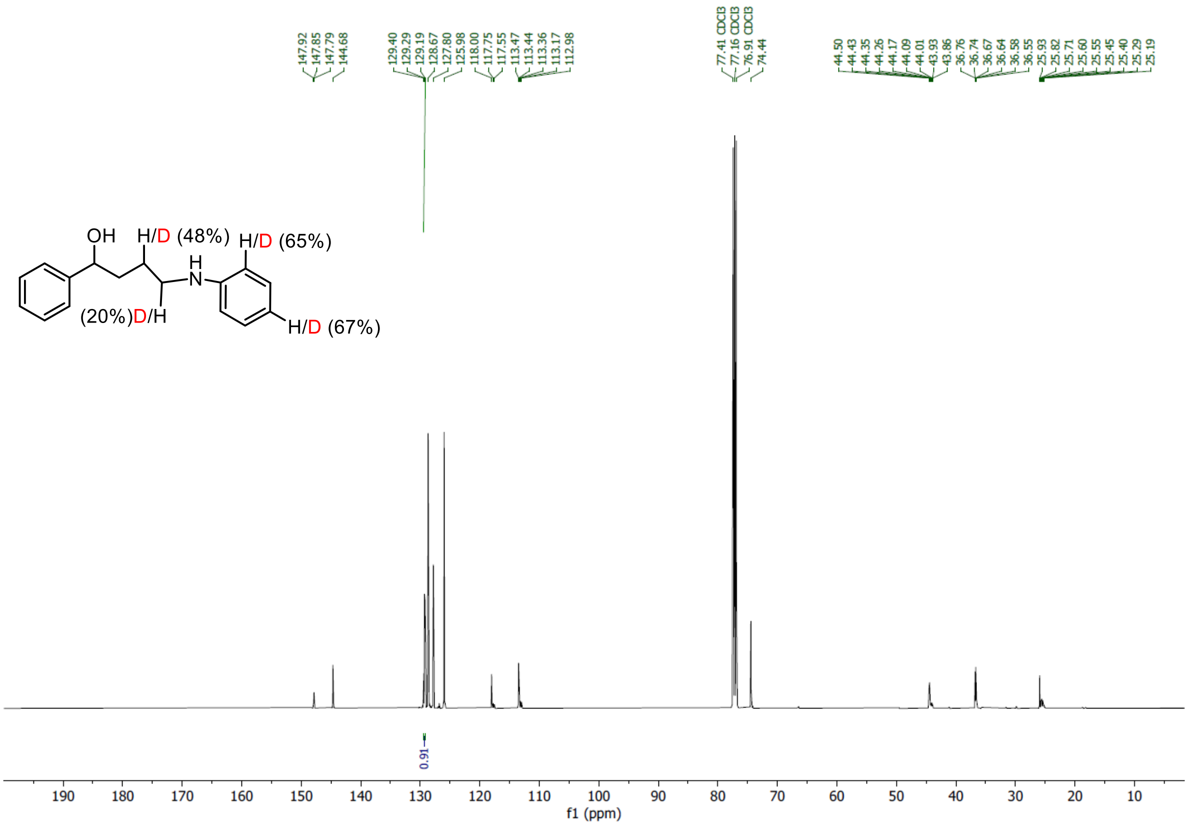

Figure S66. (Top) ^{1}H NMR (500 MHz) and (bottom) $^{13}C\{^{1}H\}$ NMR (126 MHz) spectra of **3aa–d(1)** in $CDCl_3$.

1–phenyl–4–(phenylamino)butan–3–*D*–1–ol [3aa–d(2)]

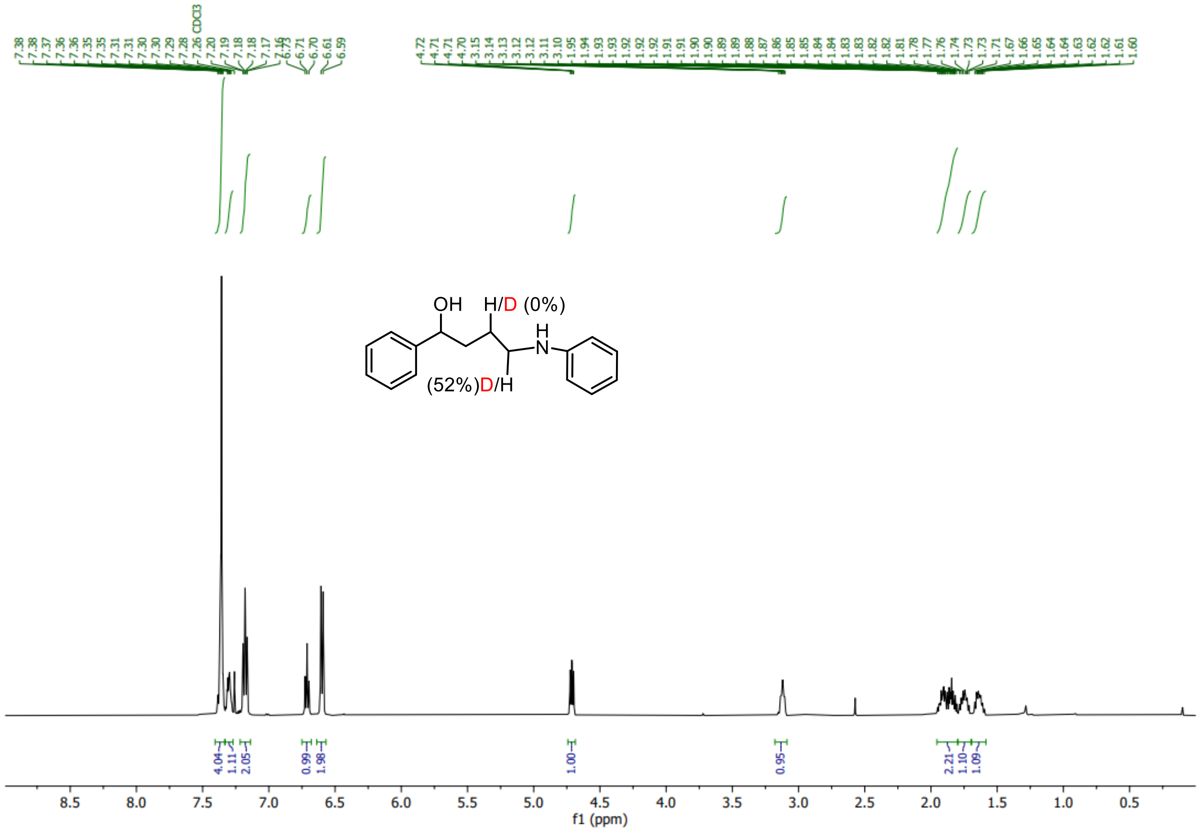

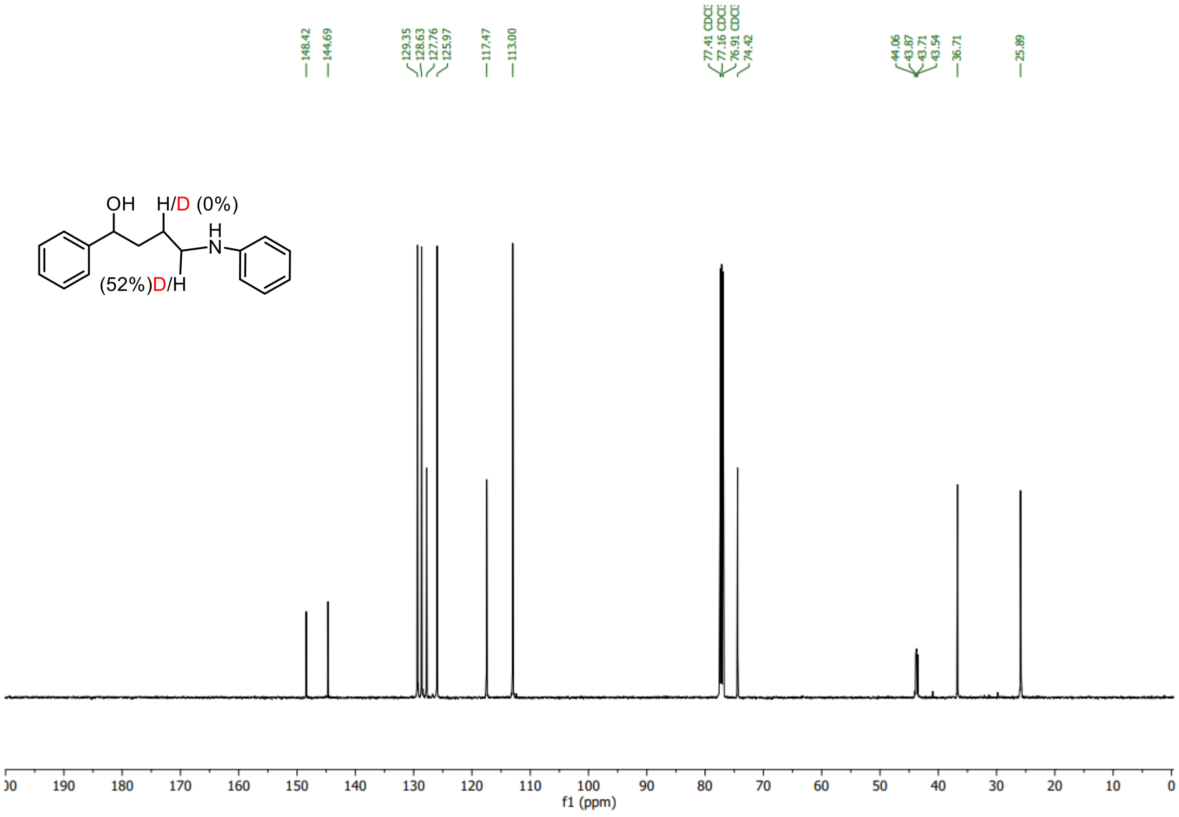

Figure S67. (Top) ^{1}H NMR (500 MHz) and (bottom) ^{13}C{^{1}H} NMR (126 MHz) spectra of **3aa–d(2)** in $CDCl_3$.

4–((2–methoxyphenyl)amino)–1–phenylbutan–1–ol (3ab)

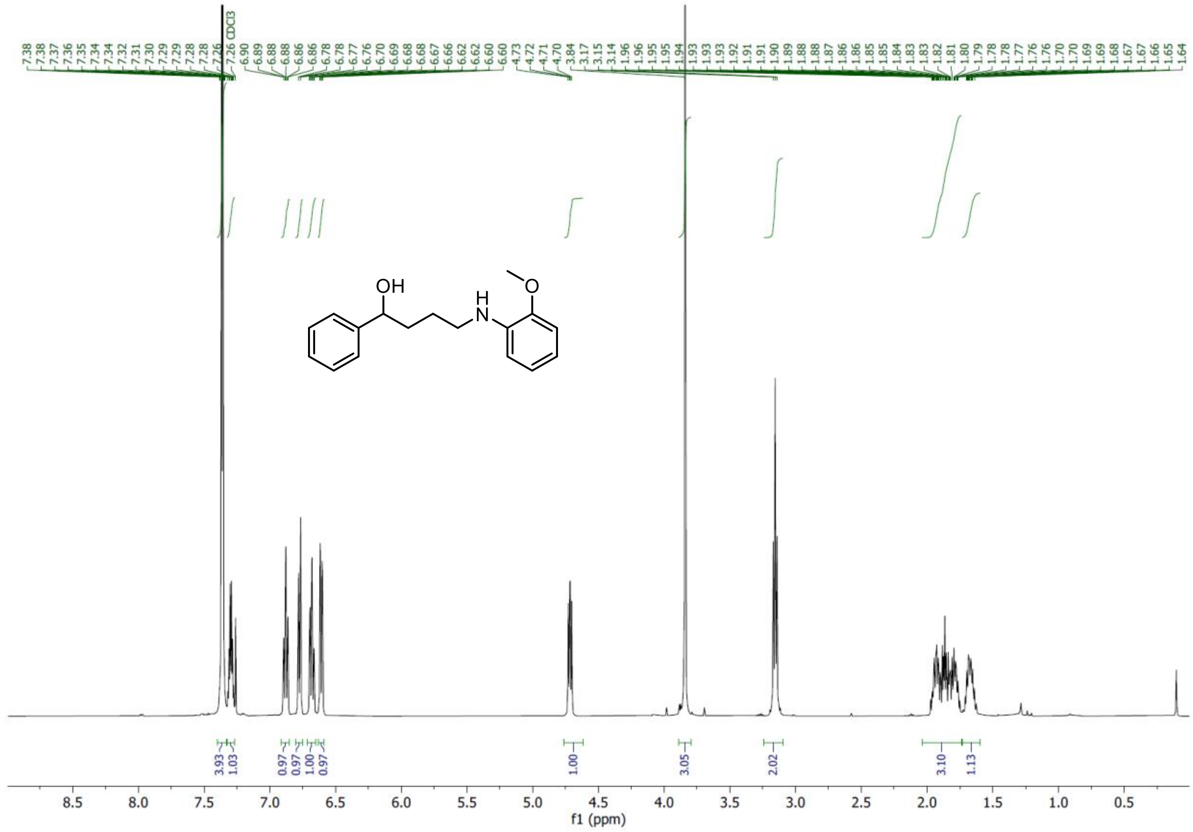

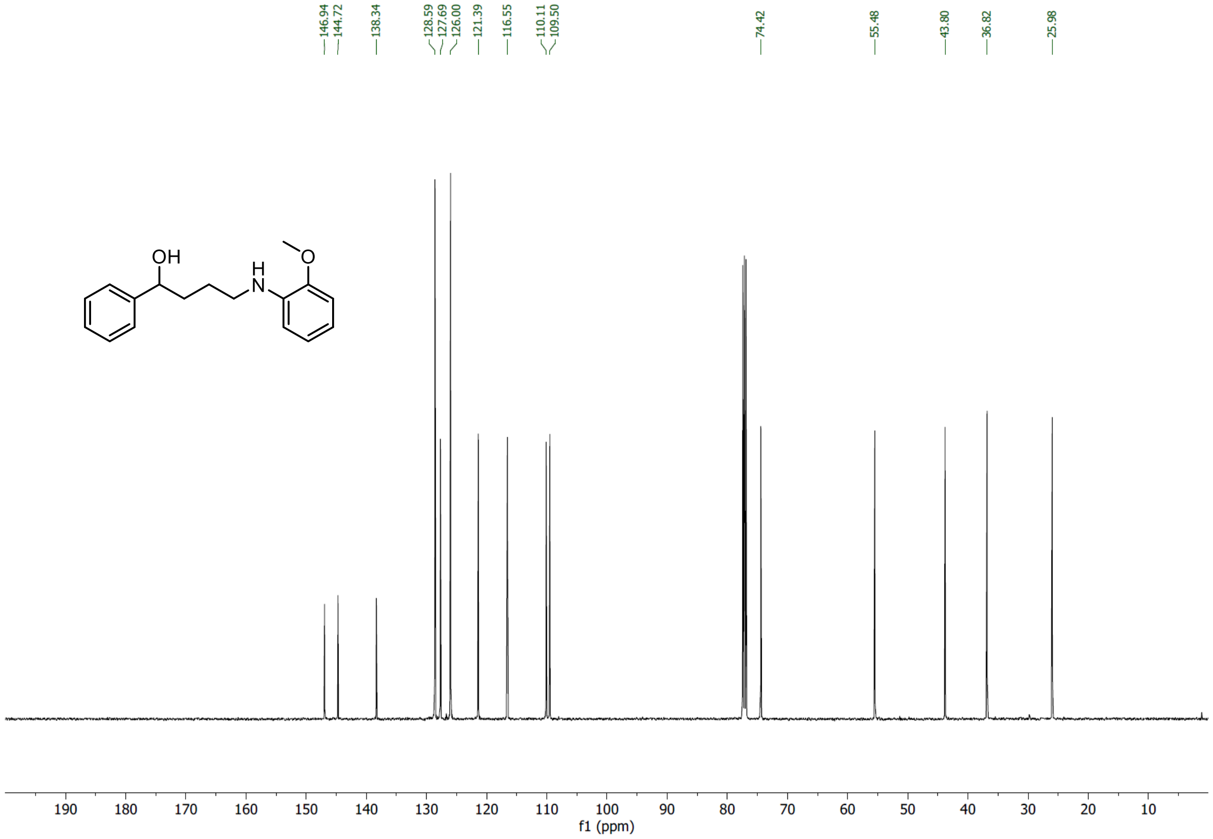

Figure S68. (Top) ^{1}H NMR (500 MHz) and (bottom) $^{13}C\{^{1}H\}$ NMR (126 MHz) spectra of **3ab** in $CDCl_3$.

4–((2–chlorophenyl)amino)–1–phenylbutan–1–ol (3ac)

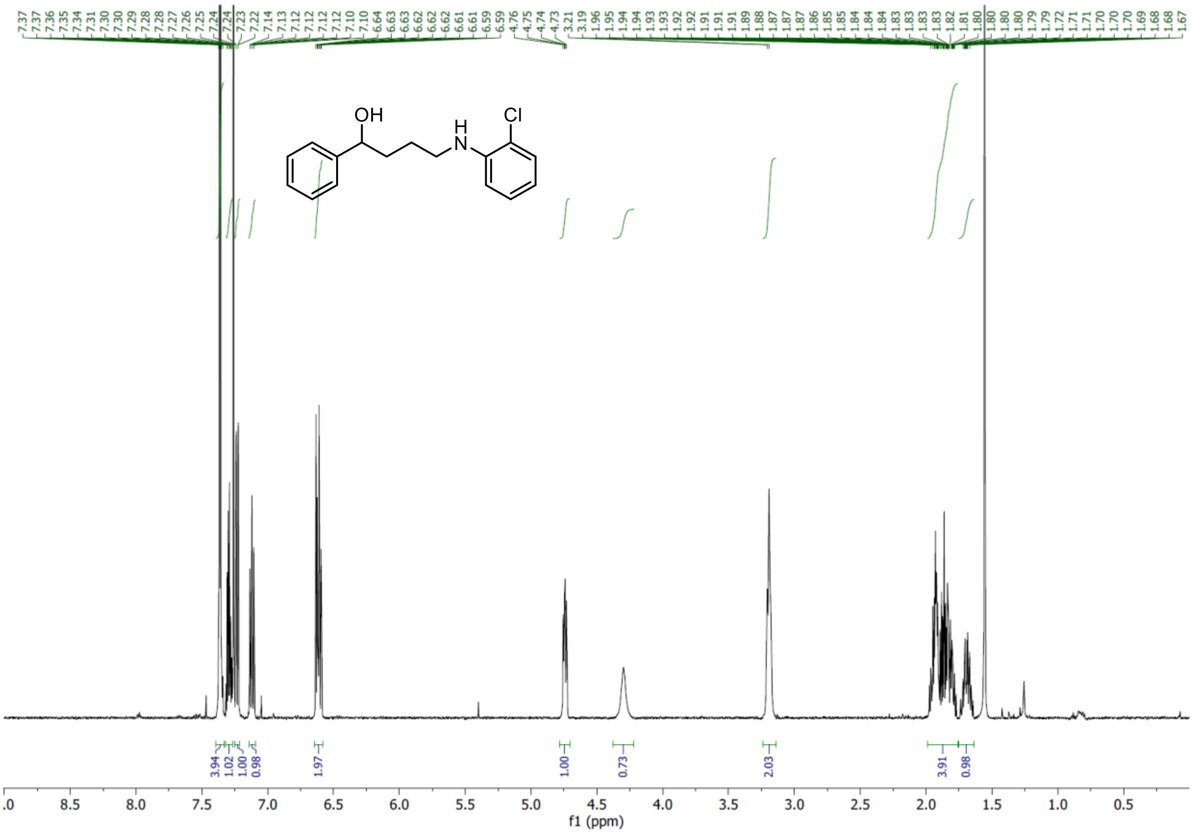

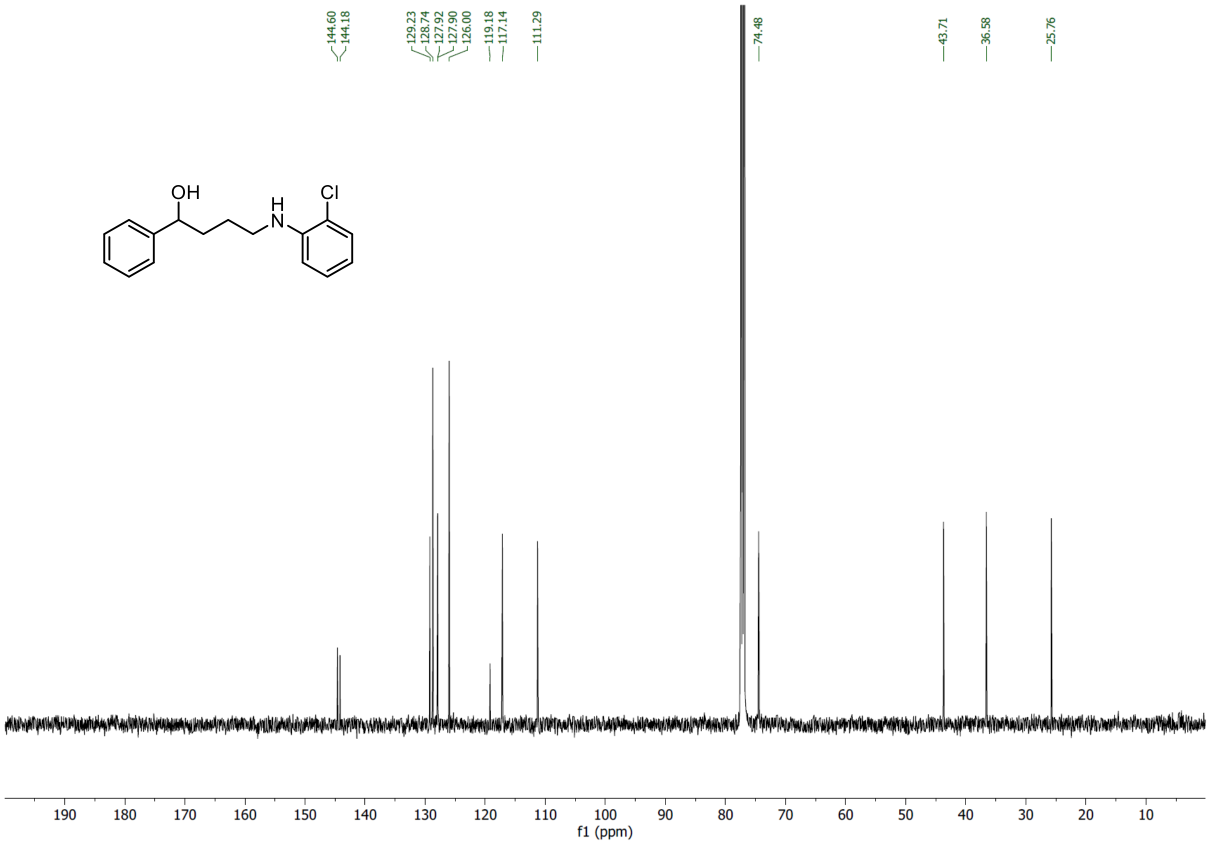

Figure S69. (Top) ^{1}H NMR (500 MHz) and (bottom) $^{13}C\{^{1}H\}$ NMR (126 MHz) spectra of **3ac** in $CDCl_3$.

1–phenyl–4–((3–(4,4,5,5–tetramethyl–1,3,2–dioxaborolan–2–yl)phenyl)amino)butan–1–ol (3ad)

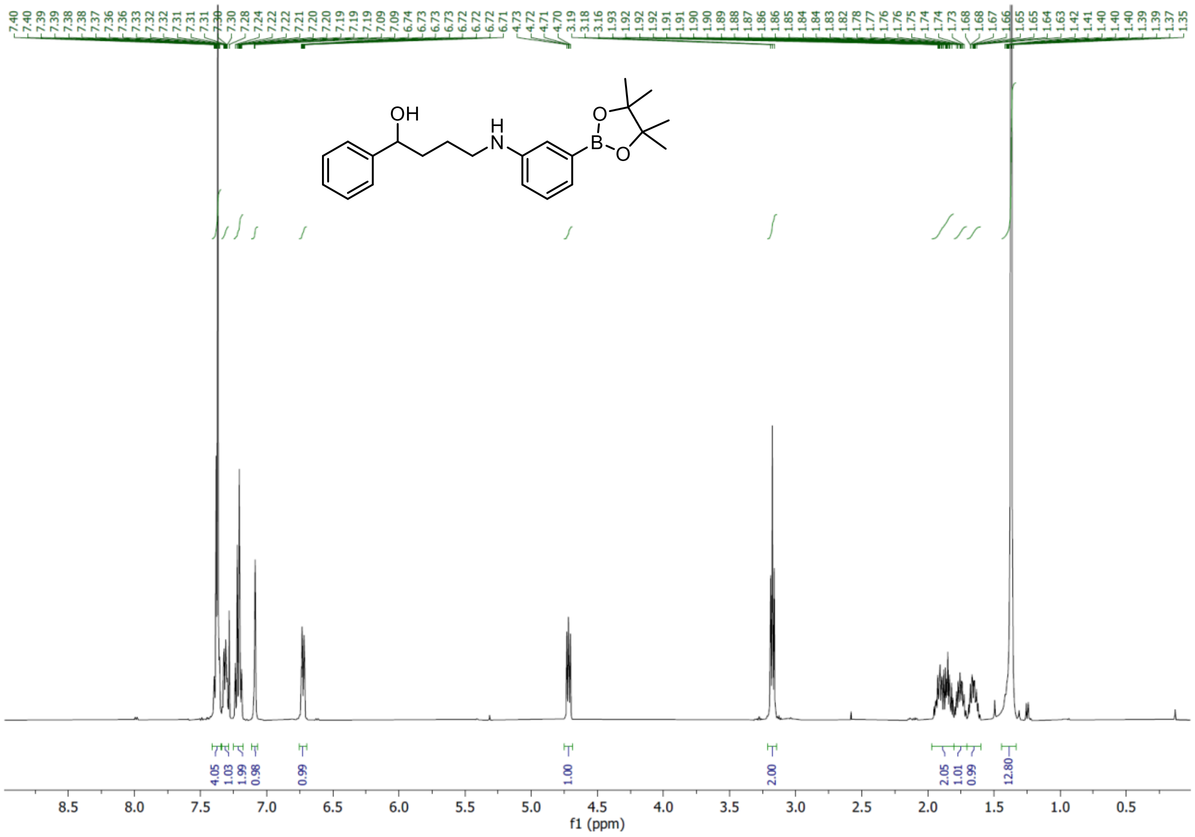

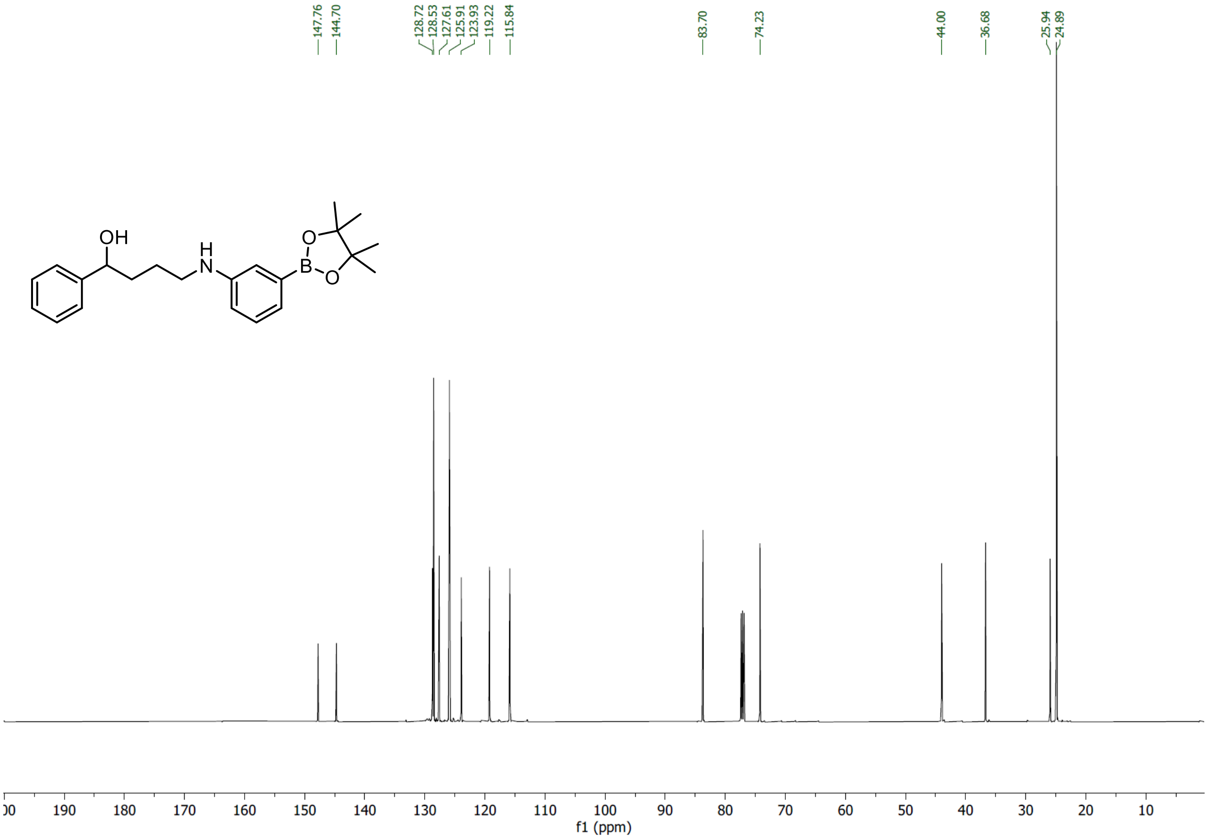

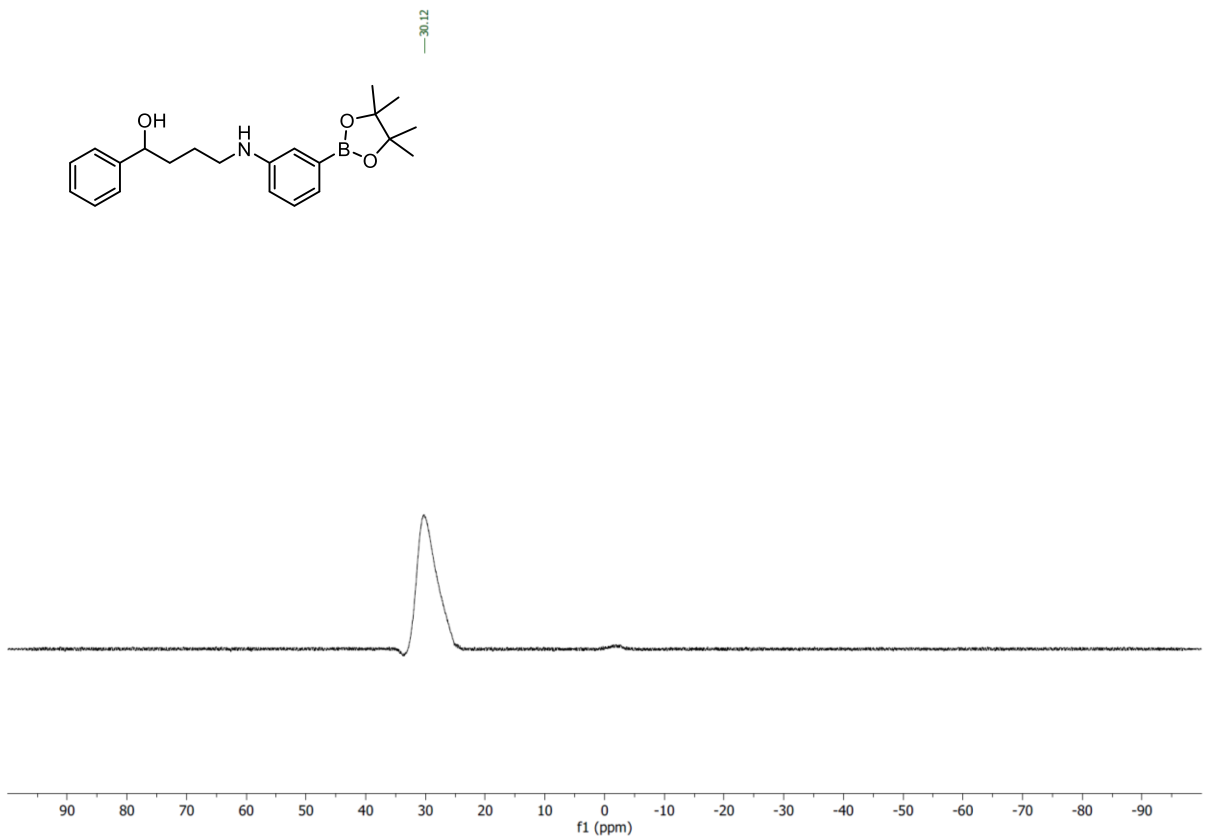

Figure S70. (Top) ^{1}H NMR (500 MHz), (center) $^{13}C\{^{1}H\}$ NMR (126 MHz) and (bottom) ^{11}B NMR (160 MHz) spectra of **3ad** in $CDCl_3$.

4–((3–bromophenyl)amino)–1–phenylbutan–1–ol (3ae)

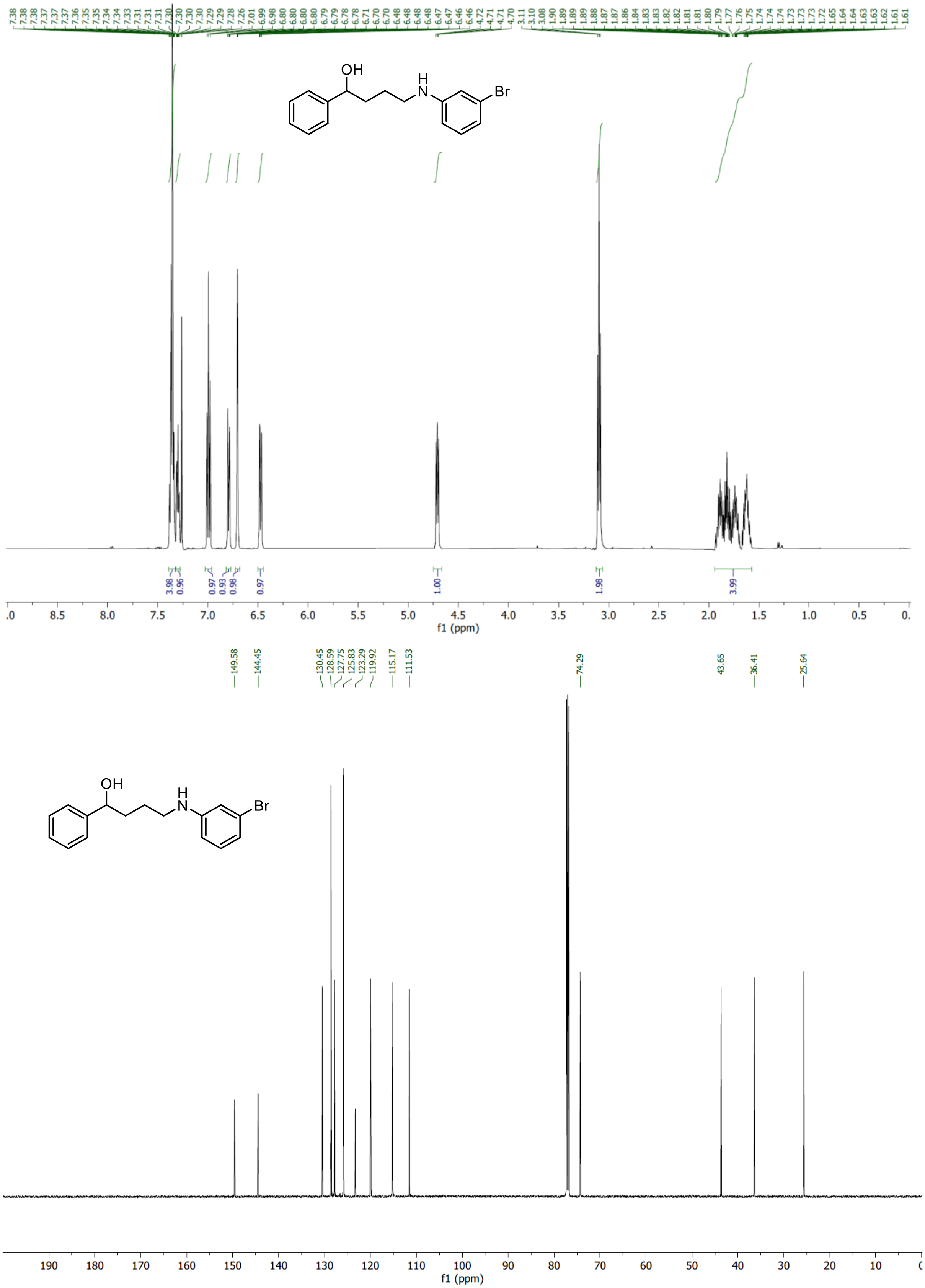

Figure S71. (Top) ^{1}H NMR (500 MHz) and (bottom) ^{13}C{^{1}H} NMR (126 MHz) spectra of **3ae** in $CDCl_3$.

1–phenyl–4–(p–tolylamino)butan–1–ol (3af)

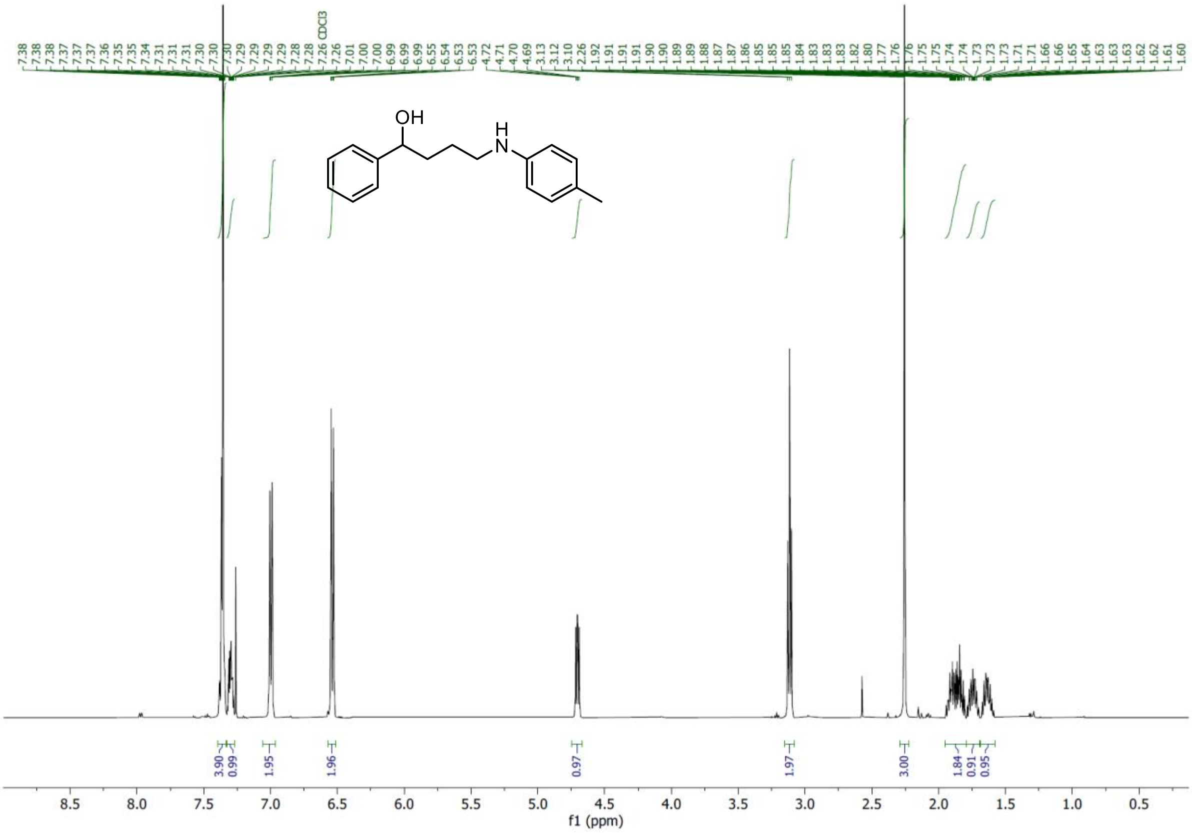

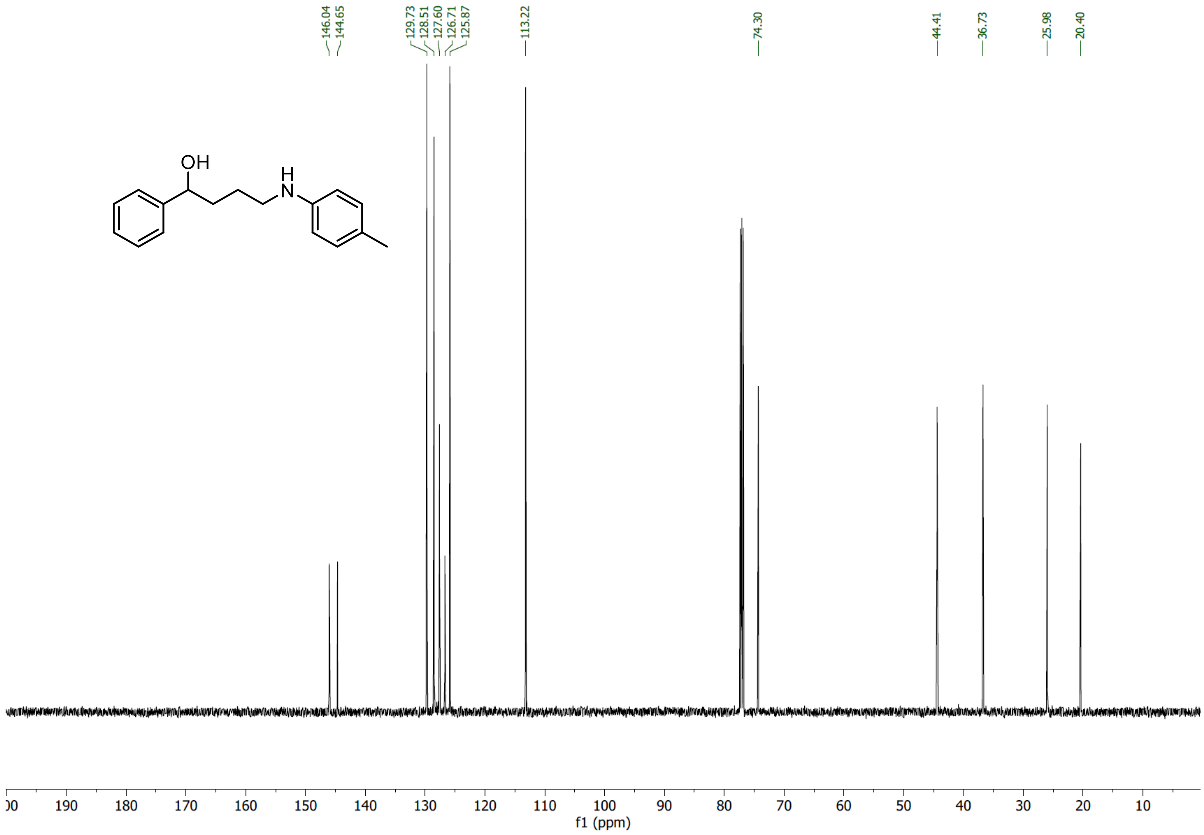

Figure S72. (Top) ^{1}H NMR (500 MHz) and (bottom) ^{13}C{^{1}H} NMR (126 MHz) spectra of **3af** in $CDCl_3$.

1–phenyl–4–((4–(trifluoromethoxy)phenyl)amino)butan–1–ol (3ag)

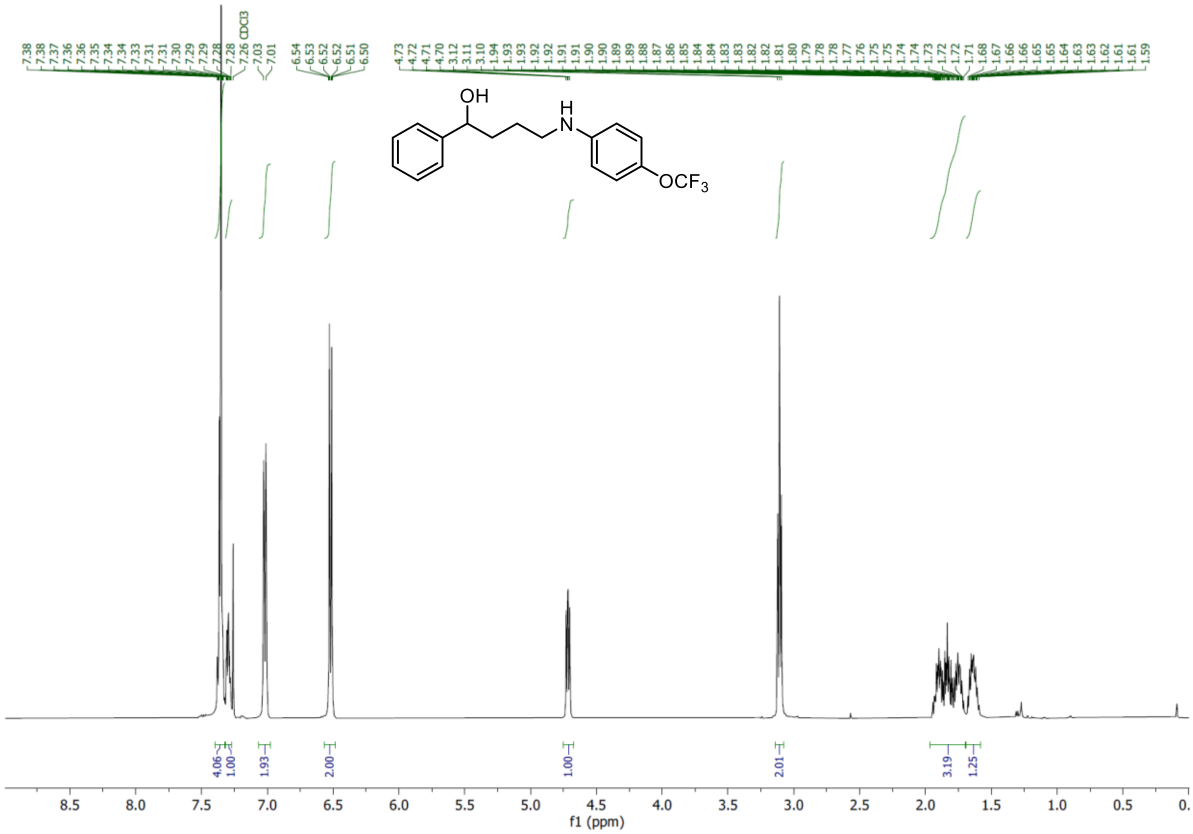

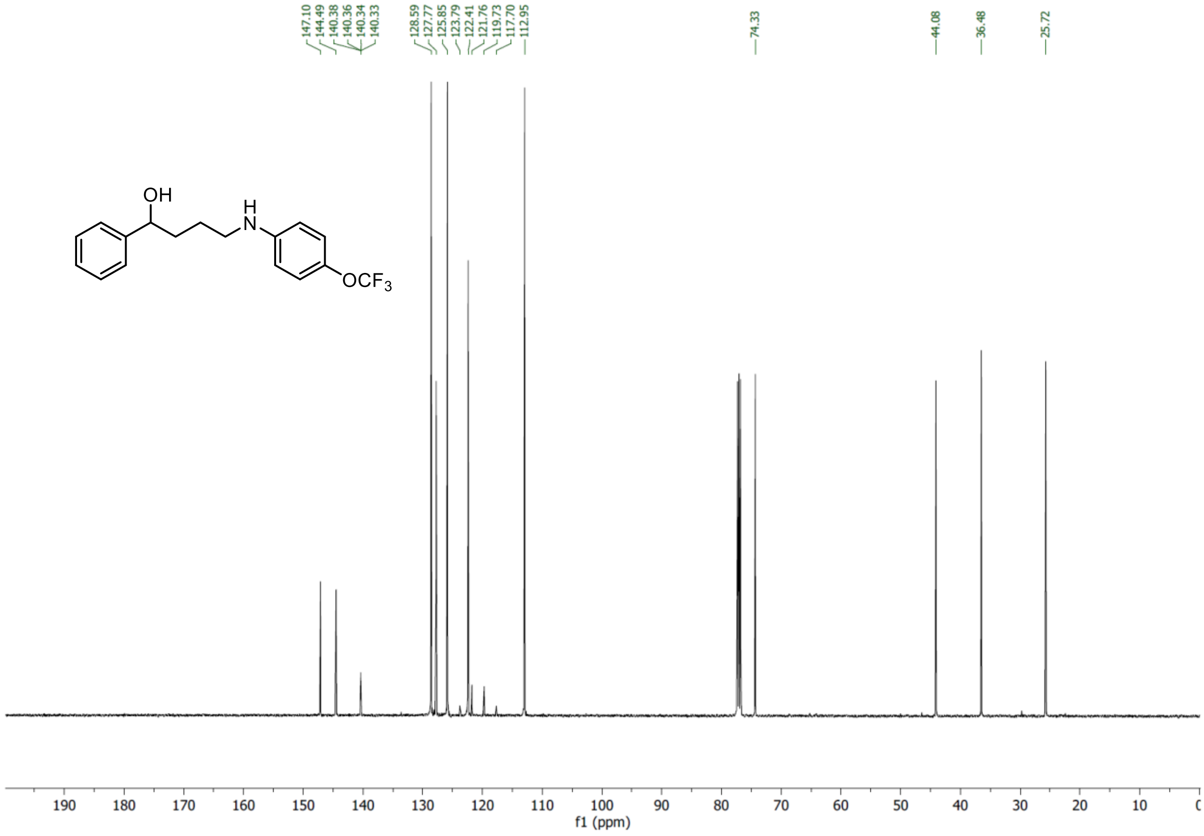

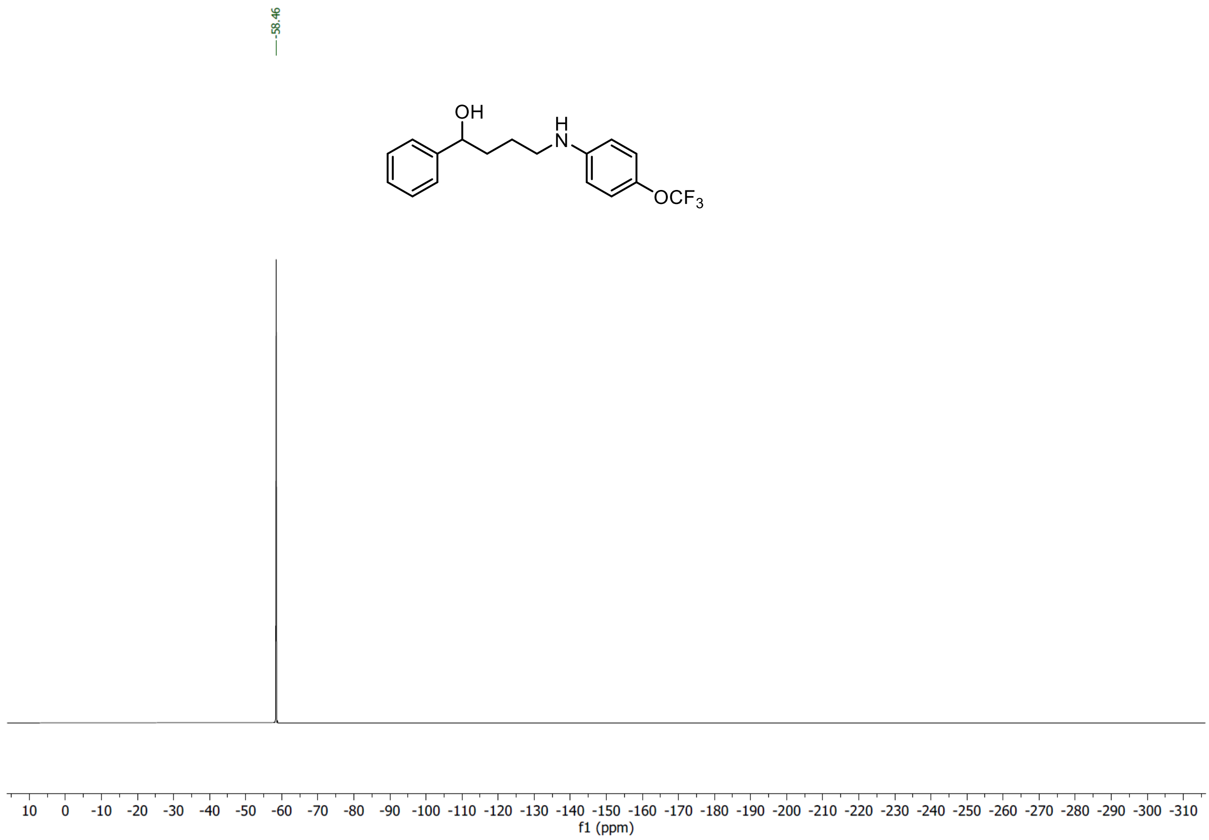

Figure S73. (Top) ^{1}H NMR (500 MHz), (centre) $^{13}C\{^{1}H\}$ NMR (126 MHz) and (bottom) ^{19}F NMR (470 MHz) spectra of **3ag** in $CDCl_3$.

4–((4–methoxyphenyl)amino)–1–phenylbutan–1–ol (3ah)

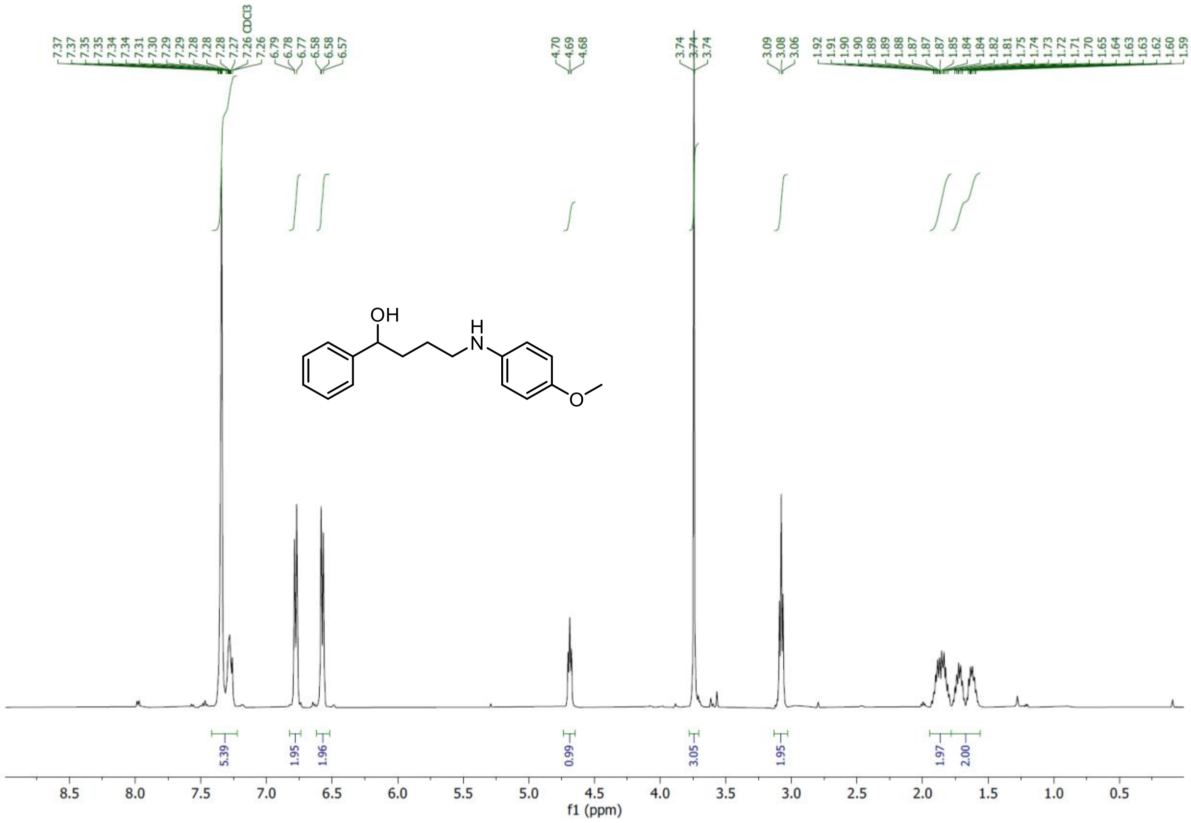

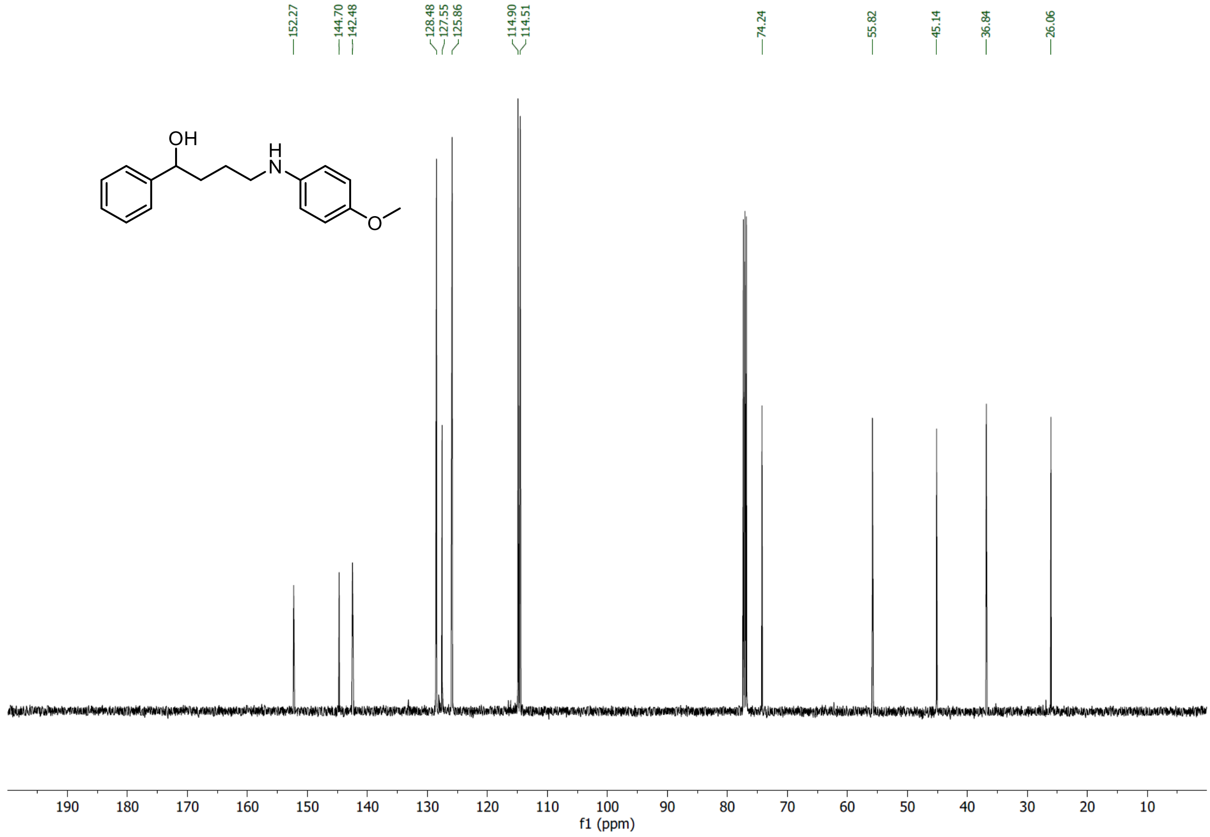

Figure S74. (Top) ^{1}H NMR (500 MHz) and (bottom) $^{13}C\{^{1}H\}$ NMR (126 MHz) spectra of **3ah** in $CDCl_3$.

1–phenyl–4–((4–((trifluoromethyl)thio)phenyl)amino)butan–1–ol (3ai)

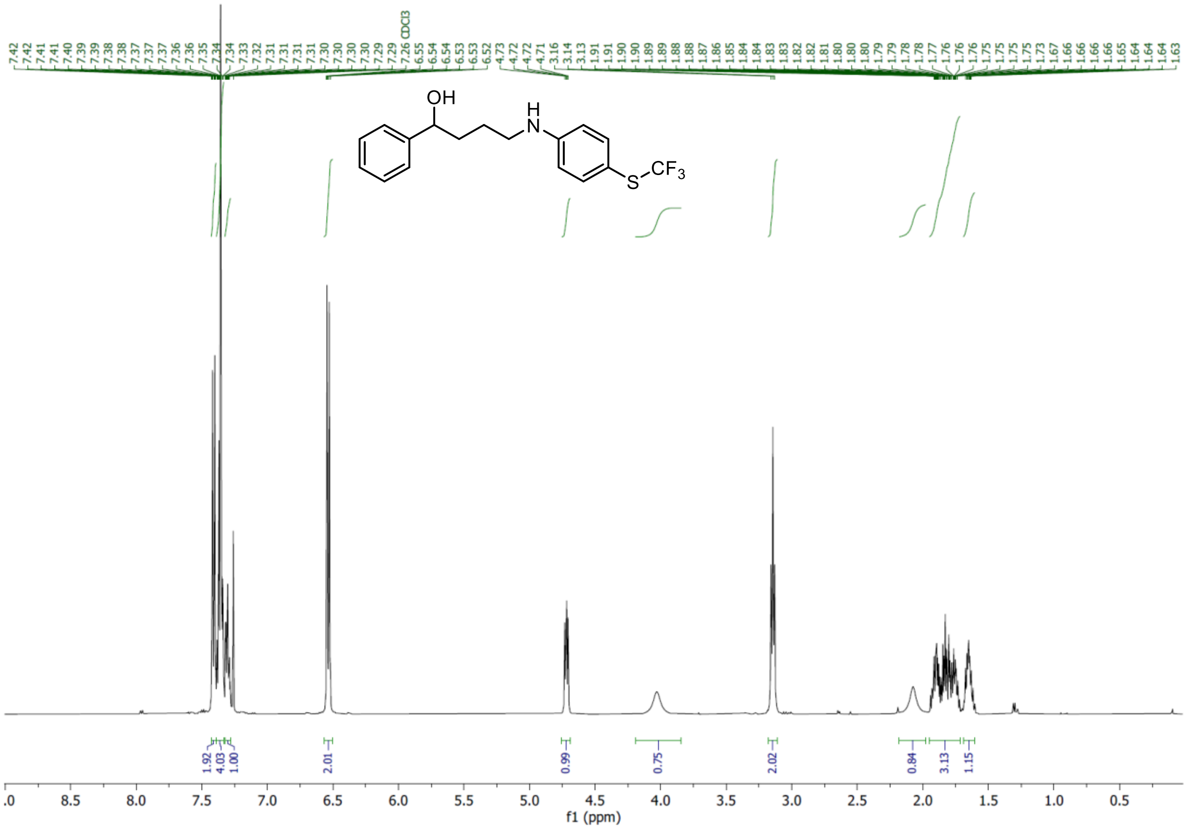

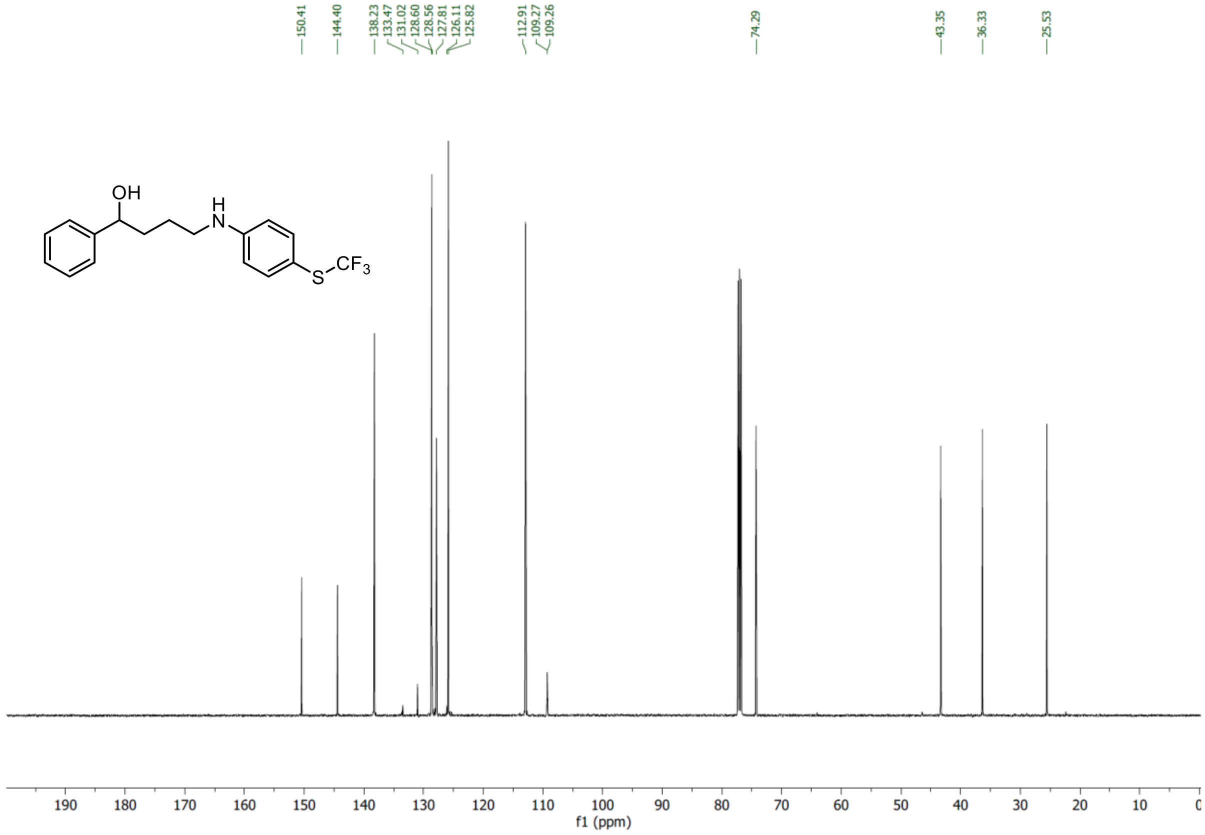

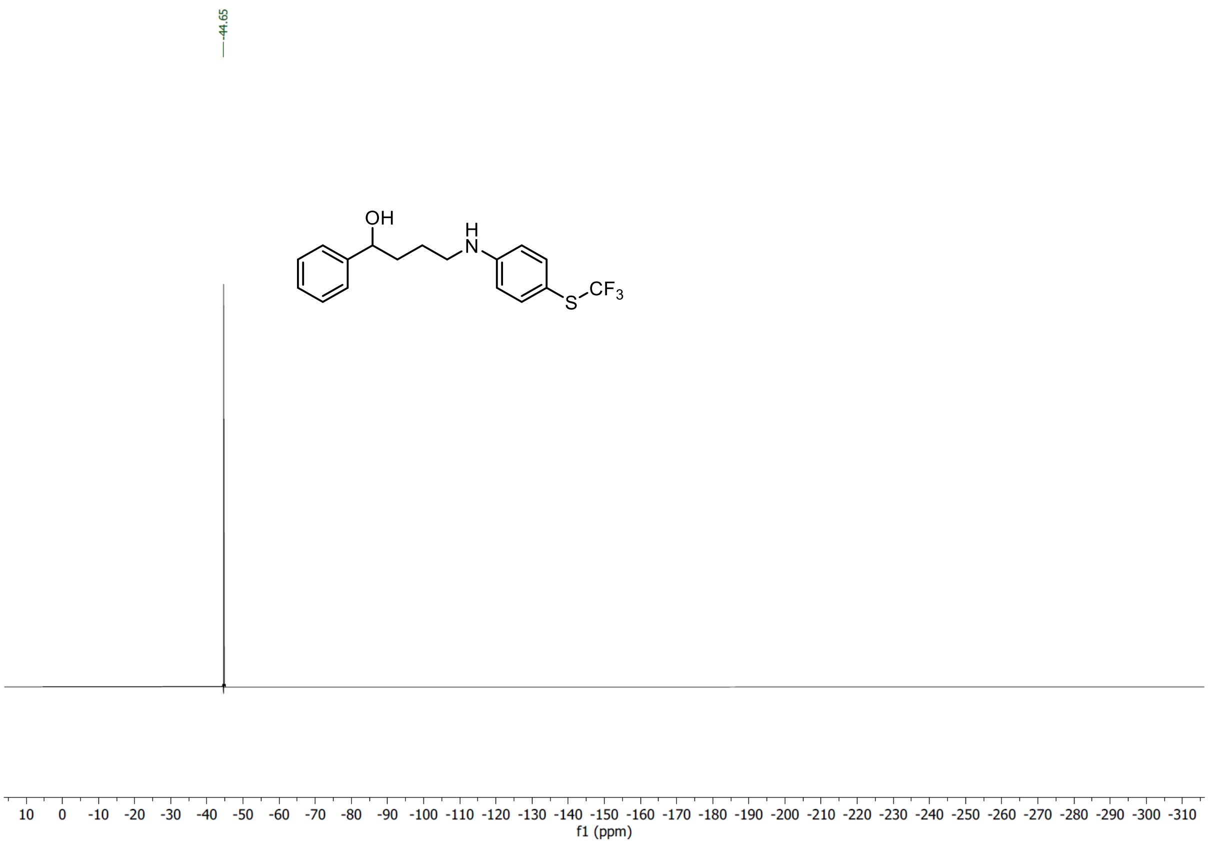

Figure S75. (Top) ^{1}H NMR (500 MHz), (centre) $^{13}C\{^{1}H\}$ NMR (126 MHz) and (bottom) ^{19}F NMR (470 MHz) spectra of **3ai** in $CDCl_3$.

4–((4–fluorophenyl)amino)–1–phenylbutan–1–ol (3aj)

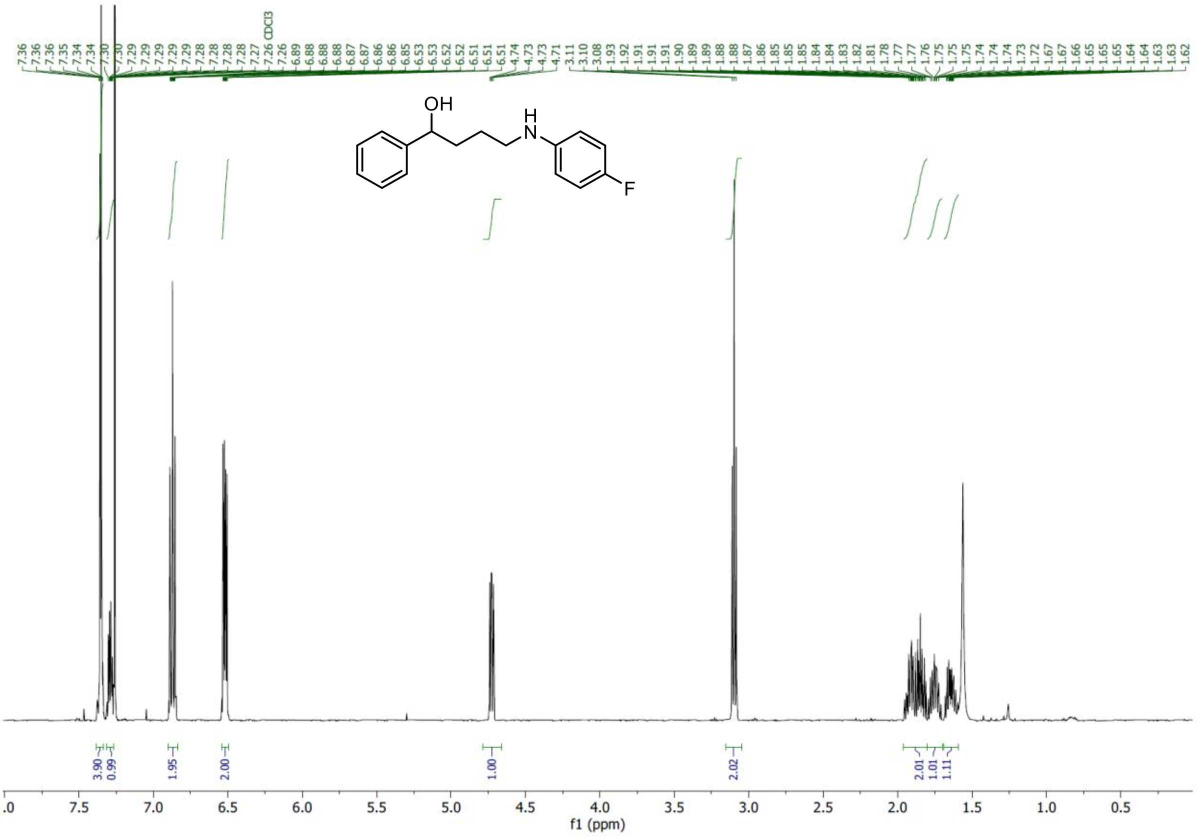

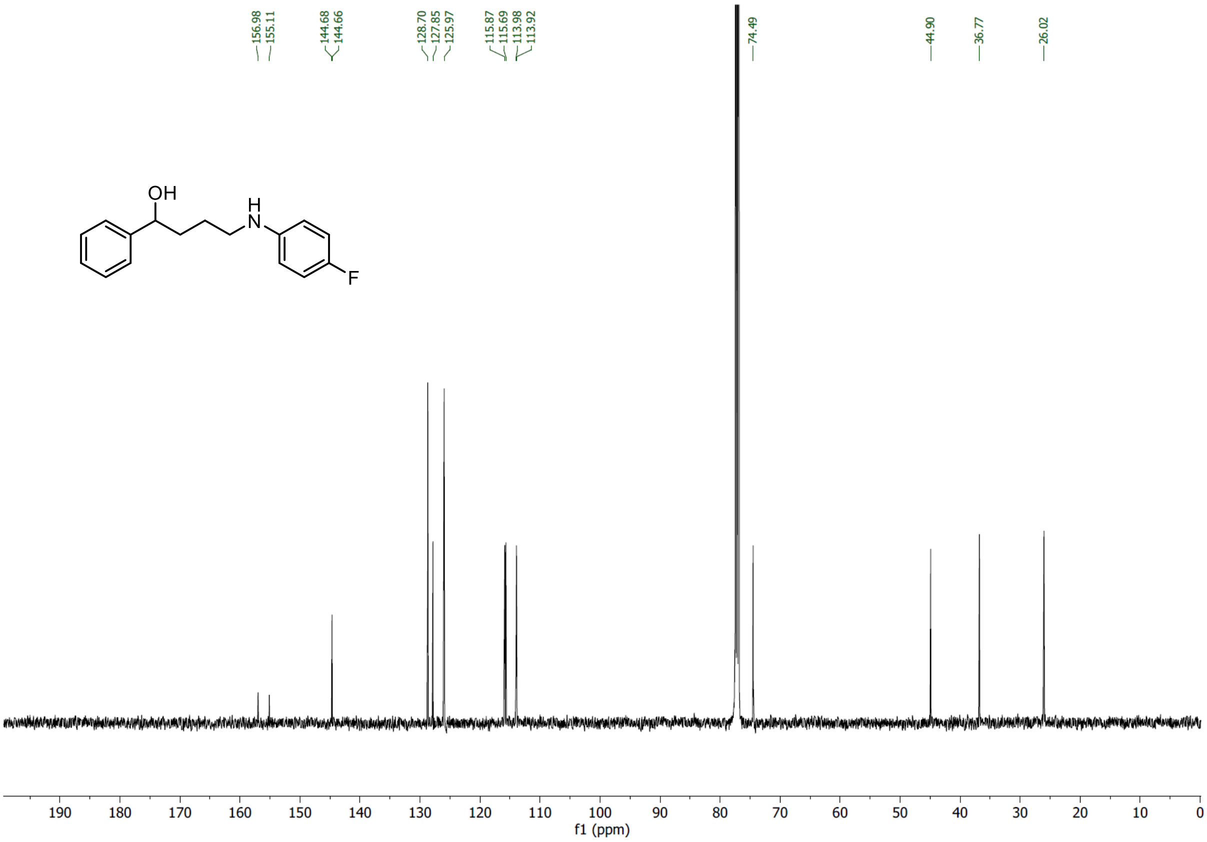

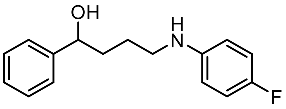

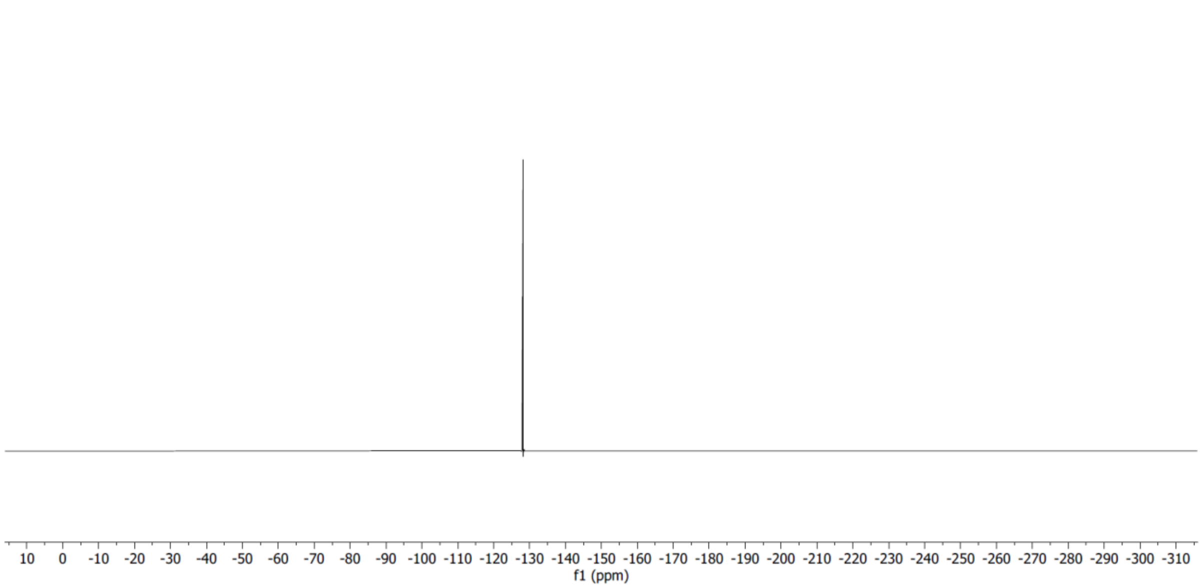

Figure S76. (Top) ^{1}H NMR (500 MHz), (centre) ^{13}C{^{1}H} NMR (126 MHz) and (bottom) ^{19}F NMR (470 MHz) spectra of **3aj** in $CDCl_3$.

Diethyl (4–((4–hydroxy–4–phenylbutyl)amino)benzyl)phosphonate (3ak)

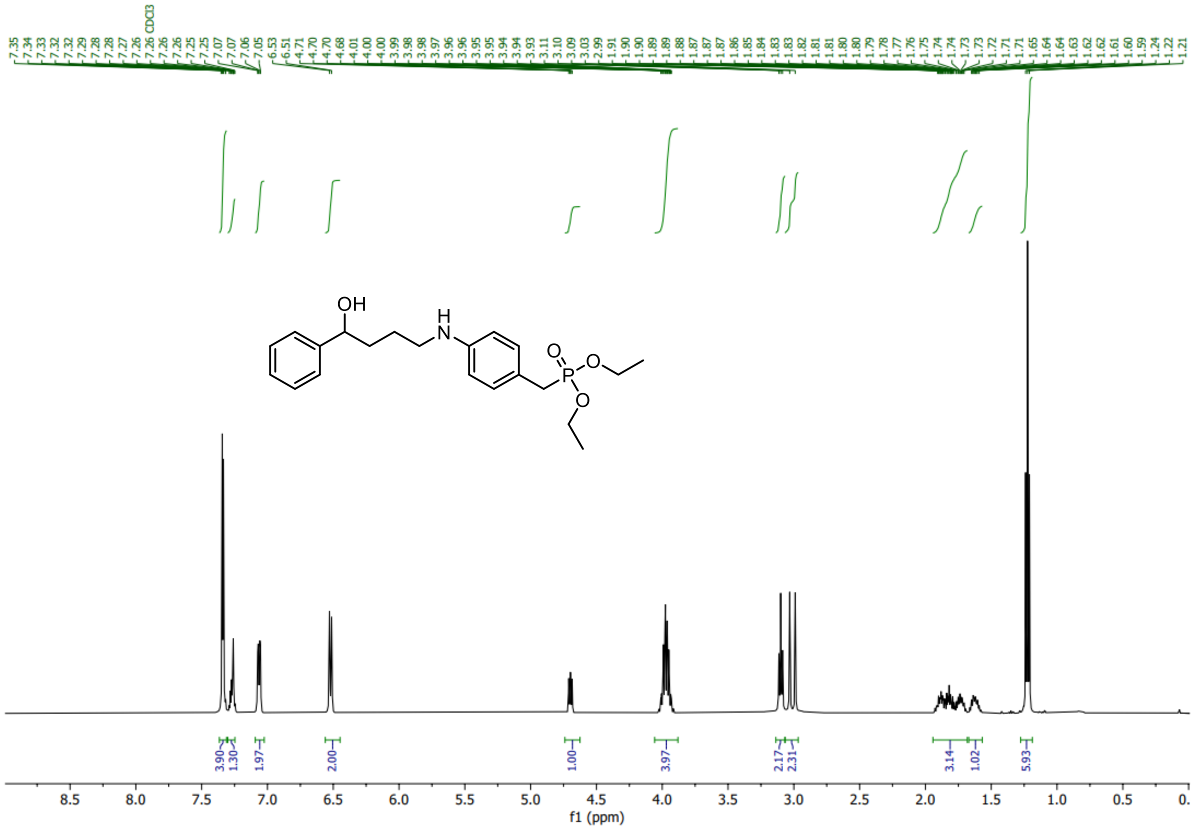

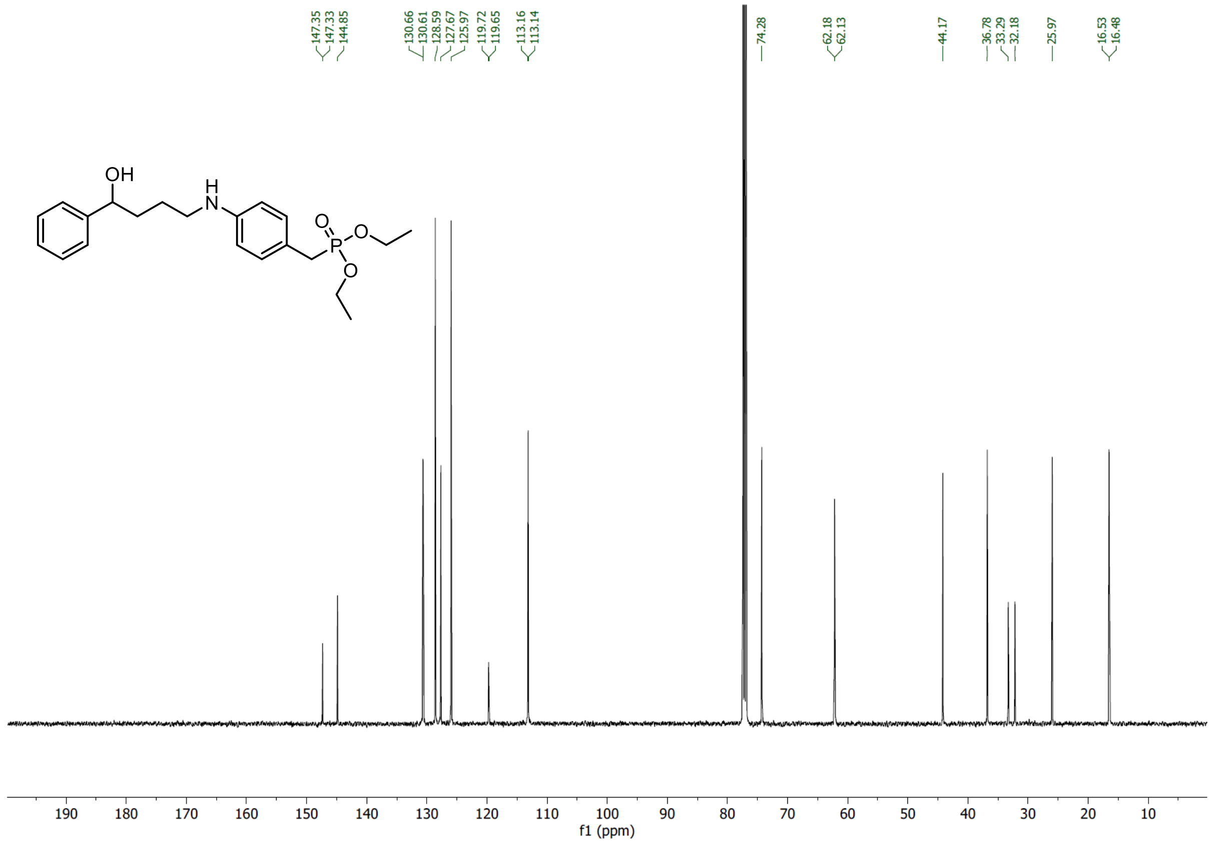

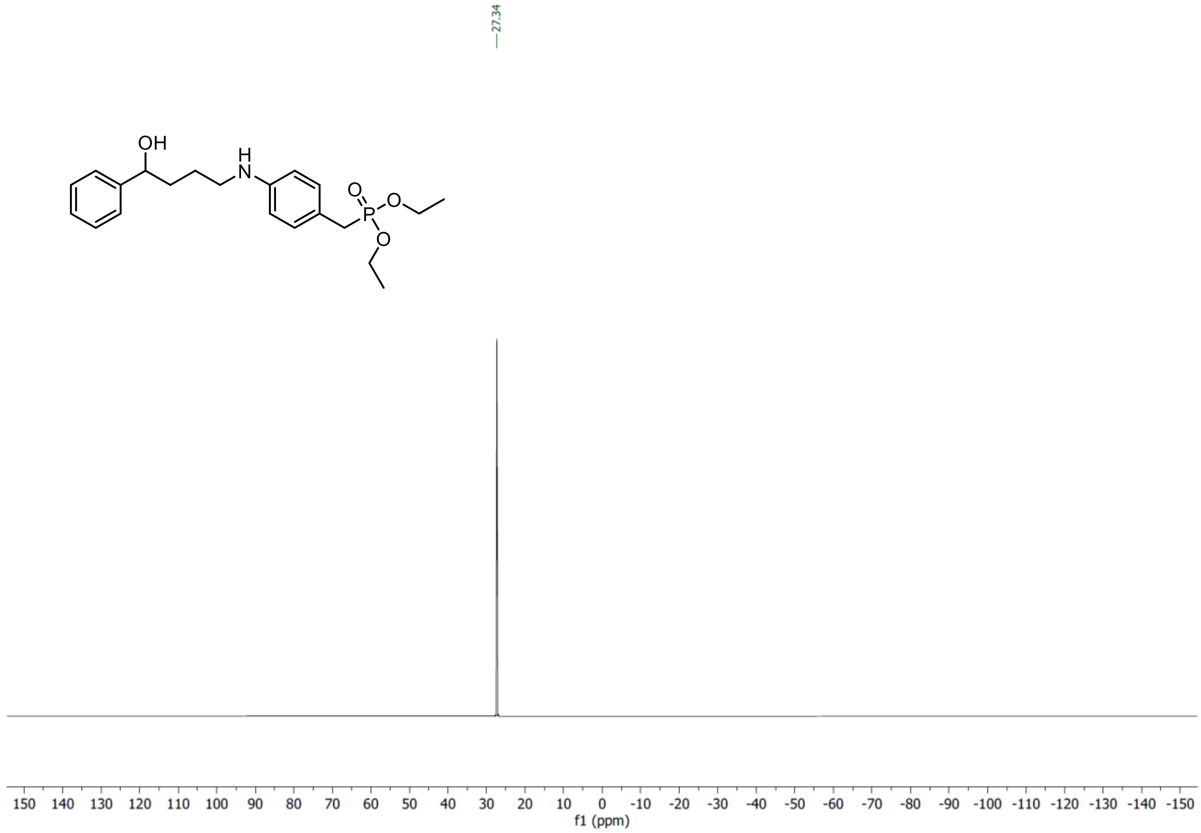

Figure S77. (Top) ^{1}H NMR (500 MHz), (centre) $^{13}C\{^{1}H\}$ NMR (126 MHz) and (bottom) ^{31}P NMR (202 MHz) spectra of **3ak** in $CDCl_3$.

4–amino–N,N–dimethylbenzenesulfonamide (3al)

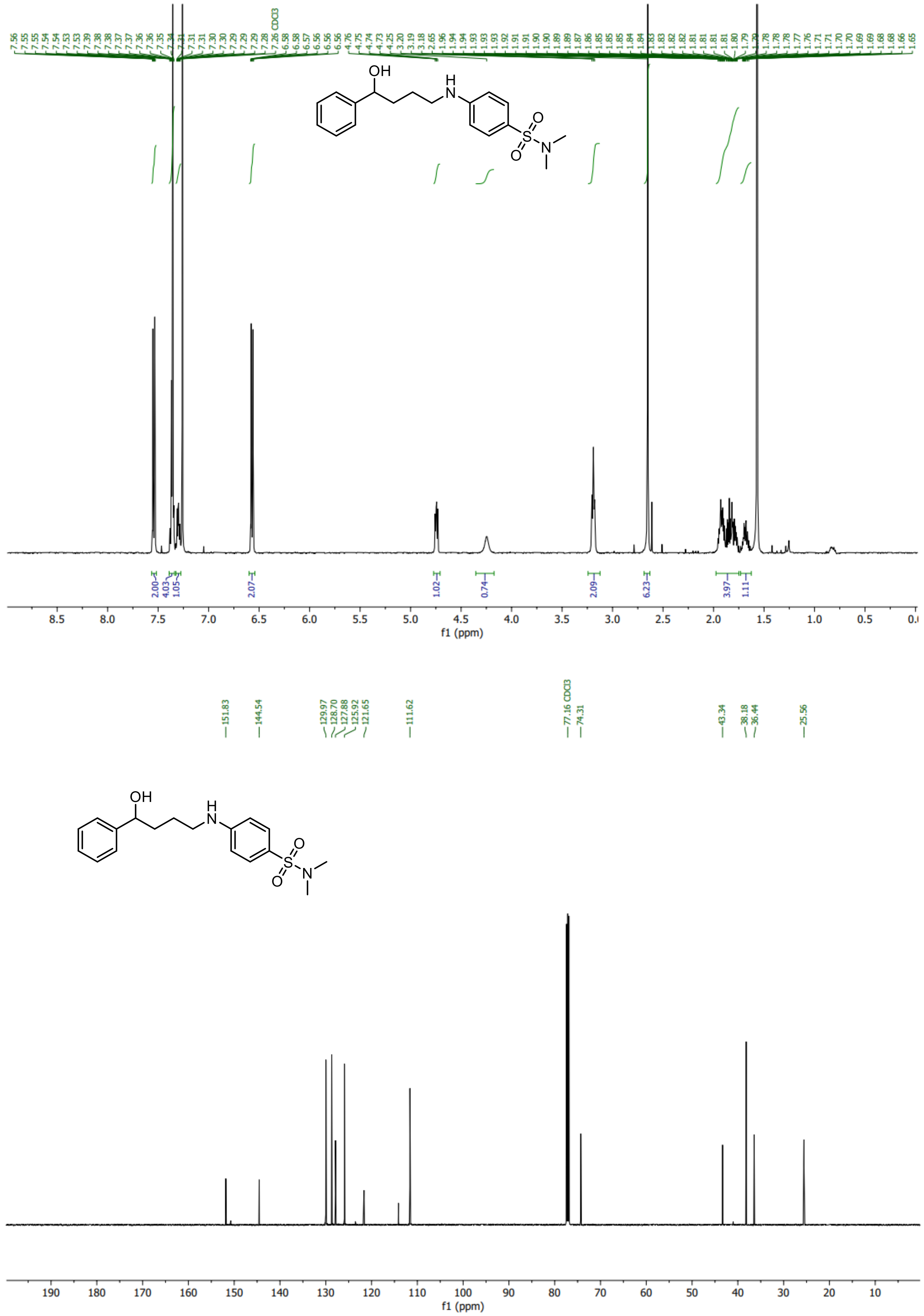

Figure S78. (Top) ^{1}H NMR (500 MHz) and (bottom) $^{13}C\{^{1}H\}$ NMR (126 MHz) spectra of **3al** in $CDCl_3$.

1.7.3 Spectra of *Late-stage functionalization* (**7aa – 7dd**)

(3R,3aS,6aR)-hexahydrofuro[2,3-b]furan-3-yl-((2S,3R)-3-hydroxy-4-((4-((4-hydroxy-4-phenylbutyl)amino)-N-isobutylphenyl)sulfonamido)-1-phenylbutan-2-yl)carbamate (7aa)

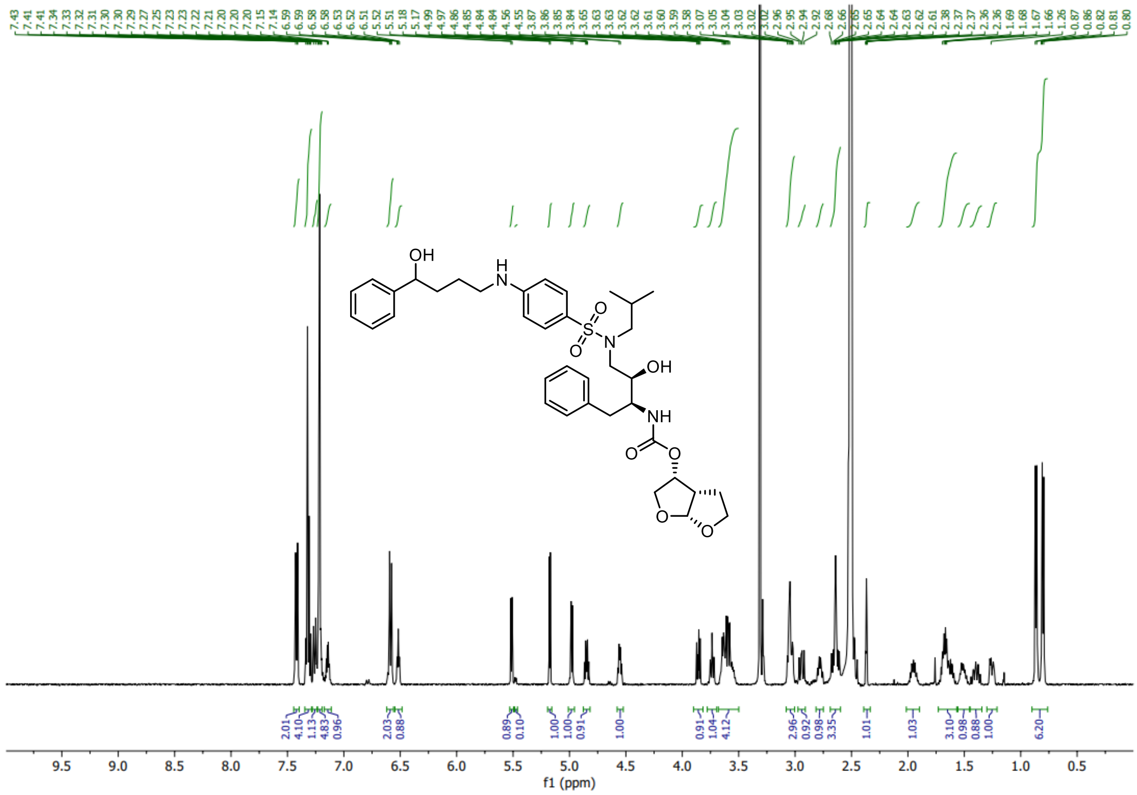

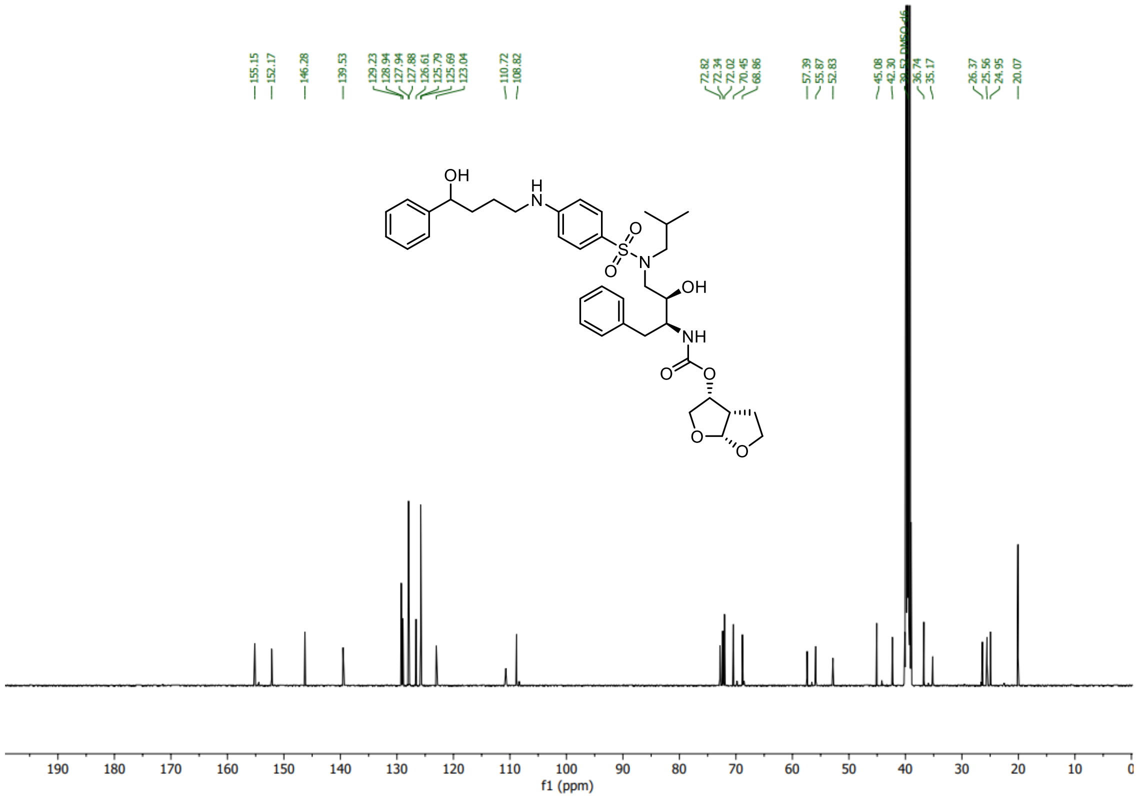

Figure S79. (Top) ^1H NMR (500 MHz) and (bottom) $^{13}C\{^1H\}$ NMR (126 MHz) spectra of **7aa** in DMSO-d_6.

3-ethyl-3-(4-((4-hydroxy-4-phenylbutyl)amino)phenyl)piperidine-2,6-dione (7ab)

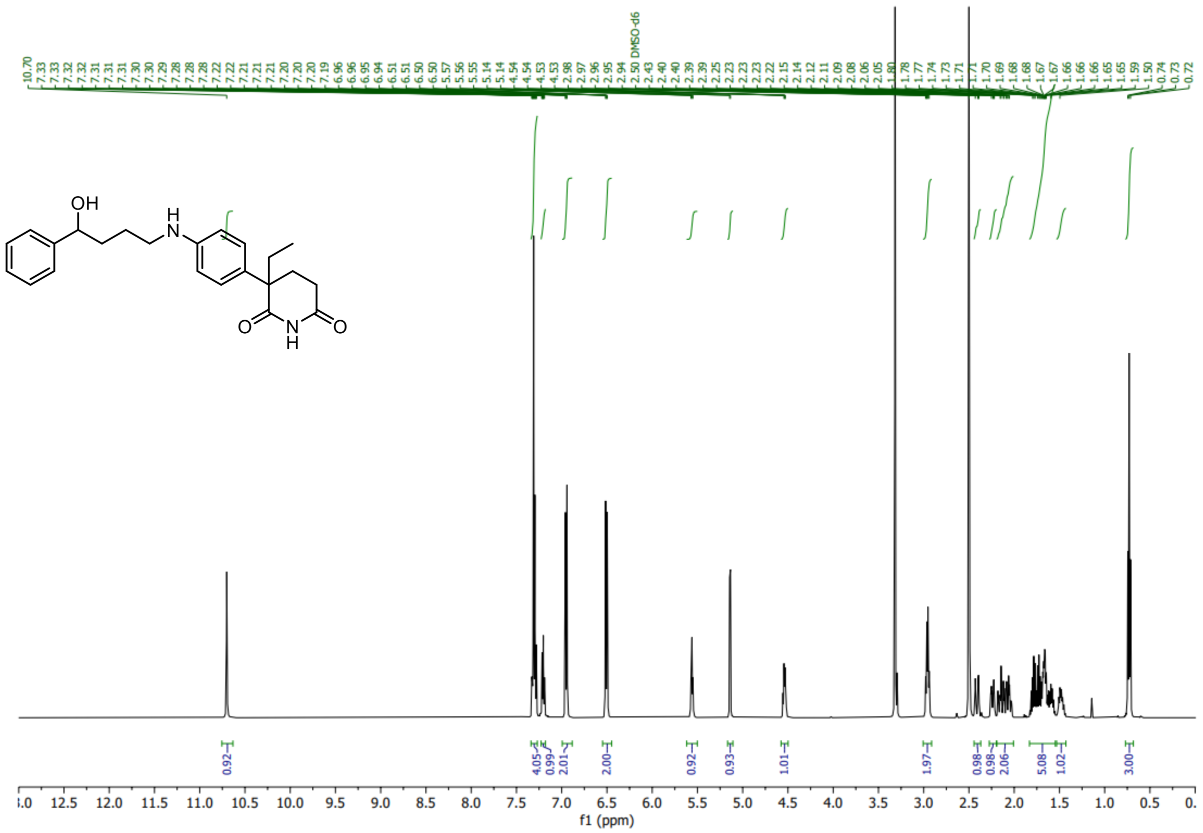

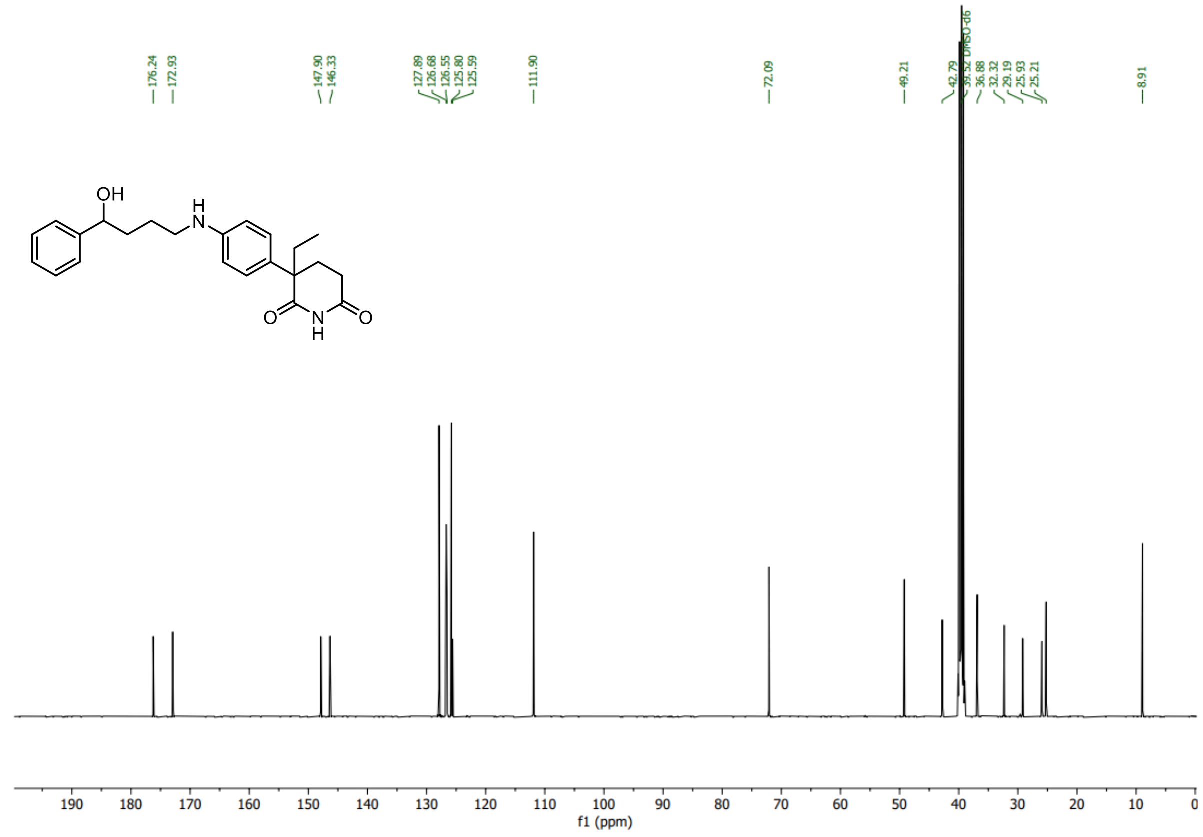

Figure S80. (Top) ^{1}H NMR (500 MHz) and (bottom) $^{13}C\{^{1}H\}$ NMR (126 MHz) spectra of **7ab** in DMSO-d_6.

3-(4-((4-hydroxy-4-phenylbutyl)amino)-1-oxoisoindolin-2-yl)piperidine-2,6-dione (7ac)

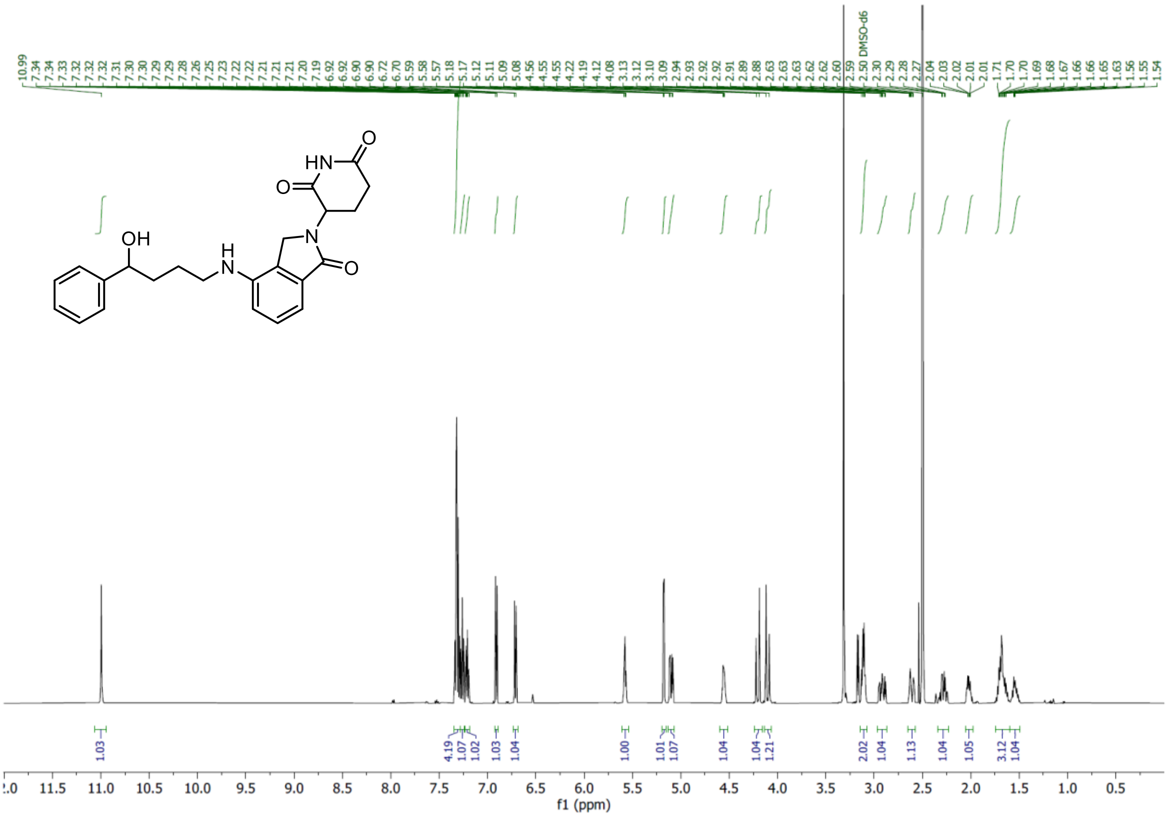

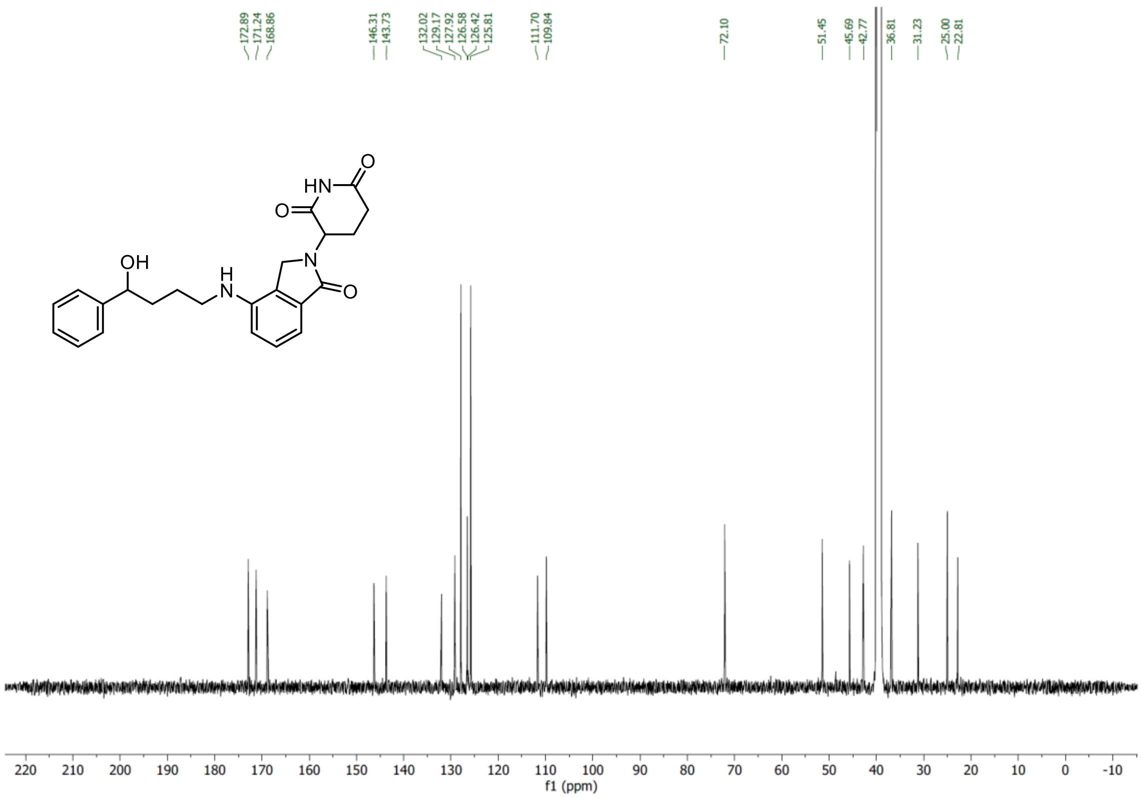

Figure S81. (Top) ^{1}H NMR (500 MHz) and (bottom) $^{13}C\{^{1}H\}$ NMR (126 MHz) spectra of **7ac** in DMSO-d_6.

3-(4-((4-(2-bromophenyl)-4-hydroxybutyl)amino)-1-oxoisoindolin-2-yl)piperidine-2,6-dione (7cc)

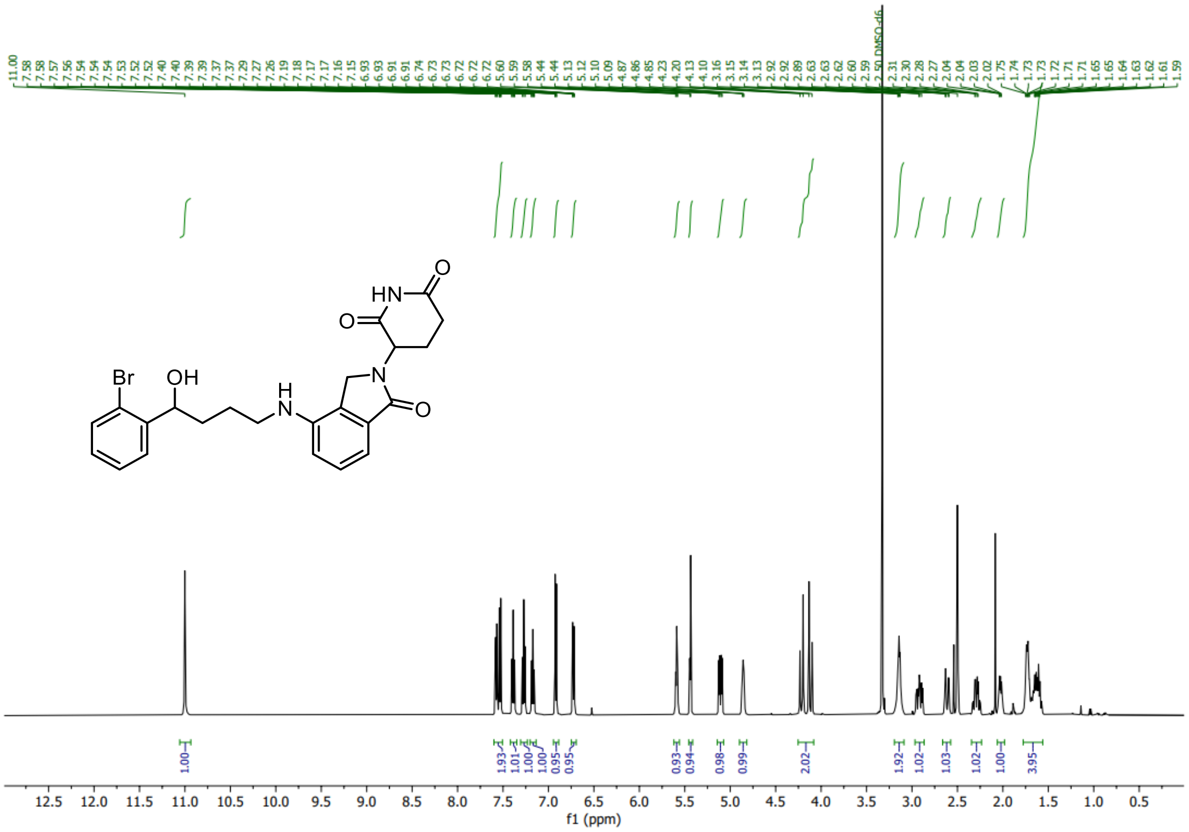

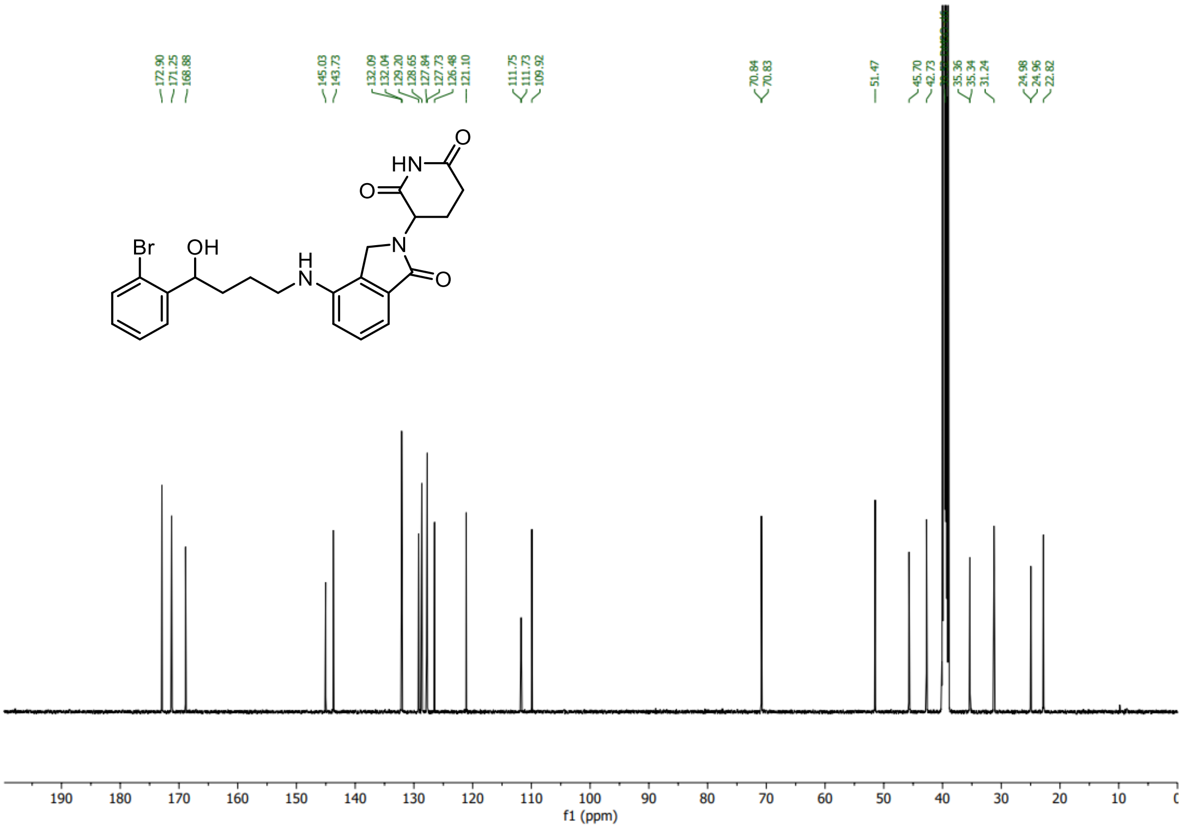

Figure S82. (Top) ^{1}H NMR (500 MHz) and (bottom) $^{13}C\{^{1}H\}$ NMR (126 MHz) spectra of **7cc** in DMSO-d_6.

3-(4-((4-(4-bromophenyl)-4-hydroxybutyl)amino)-1-oxoisoindolin-2-yl)piperidine-2,6-dione (7kc)

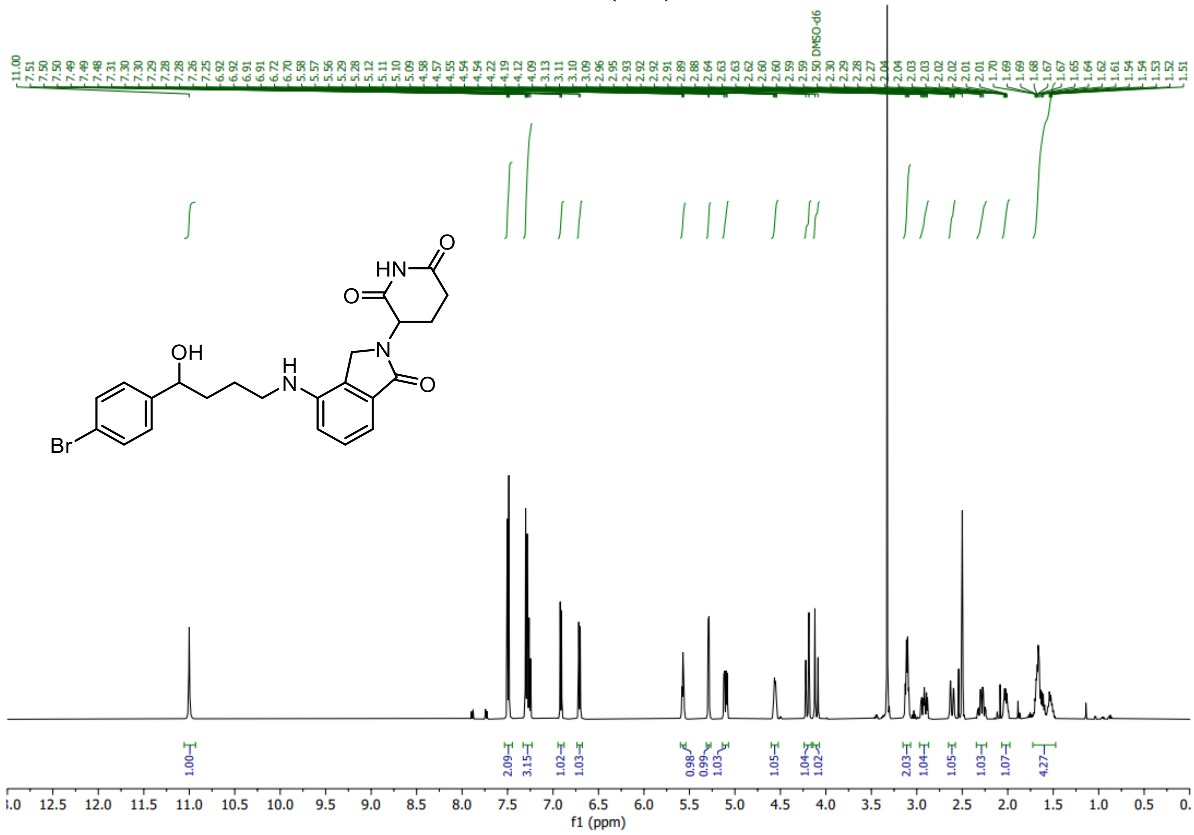

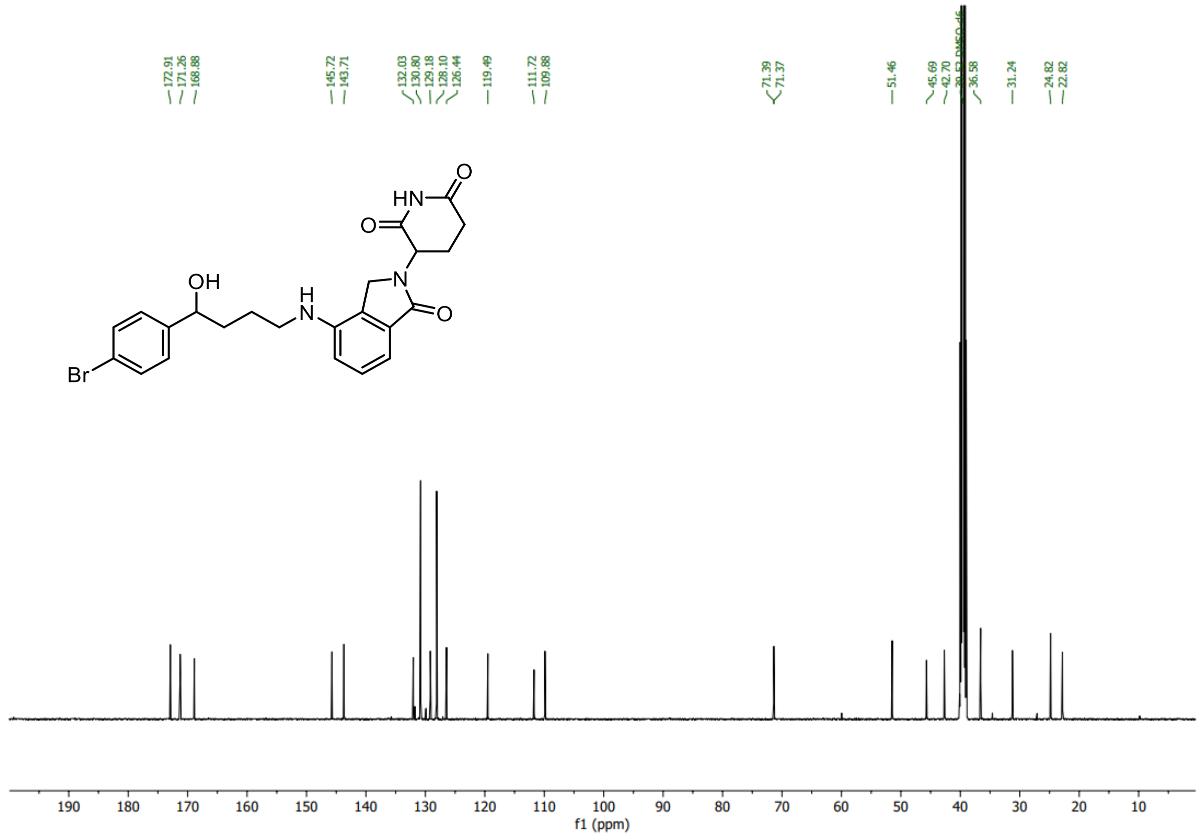

Figure S83. (Top) ^{1}H NMR (500 MHz) and (bottom) ^{13}C{^{1}H} NMR (126 MHz) spectra of **7kc** in DMSO-d_6.

3-(5-((4-hydroxy-4-phenylbutyl)amino)-1-oxoisoindolin-2-yl)piperidine-2,6-dione (5ab) oisoindolin-2-yl)piperidine-2,6-dione (7ad)

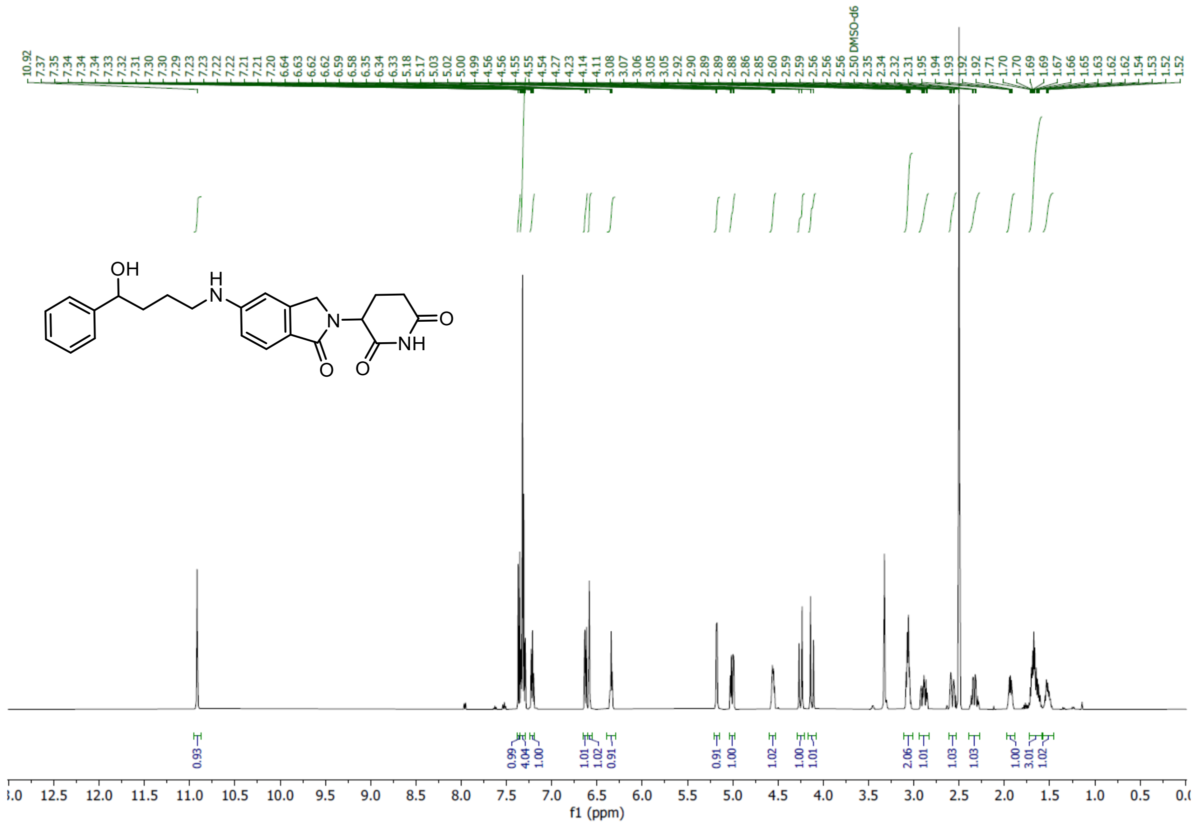

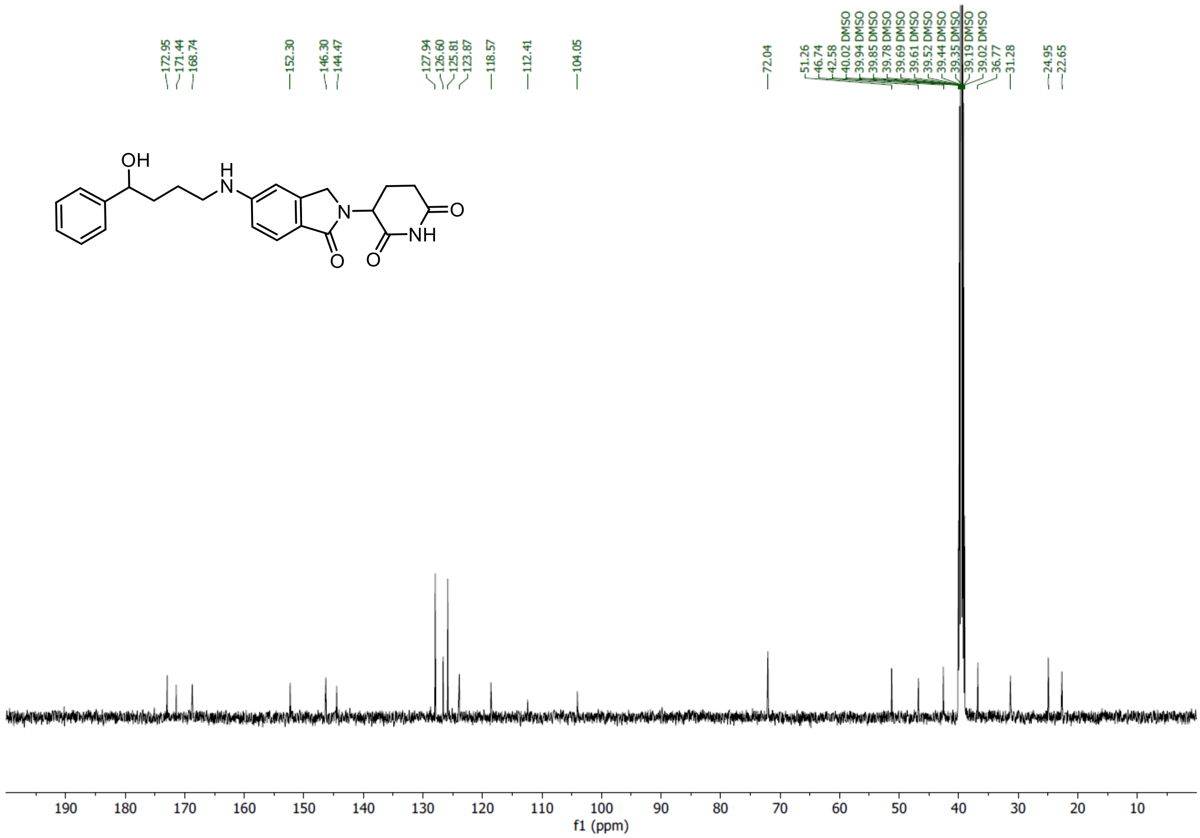

Figure S84. (Top) ^{1}H NMR (500 MHz) and (bottom) ^{13}C{^{1}H} NMR (126 MHz) spectra of **7ad** in DMSO-d_6.

3-(5-((4-(2-bromophenyl)-4-hydroxybutyl)amino)-1-oxoisoindolin-2-yl)piperidine-2,6-dione (7cd)

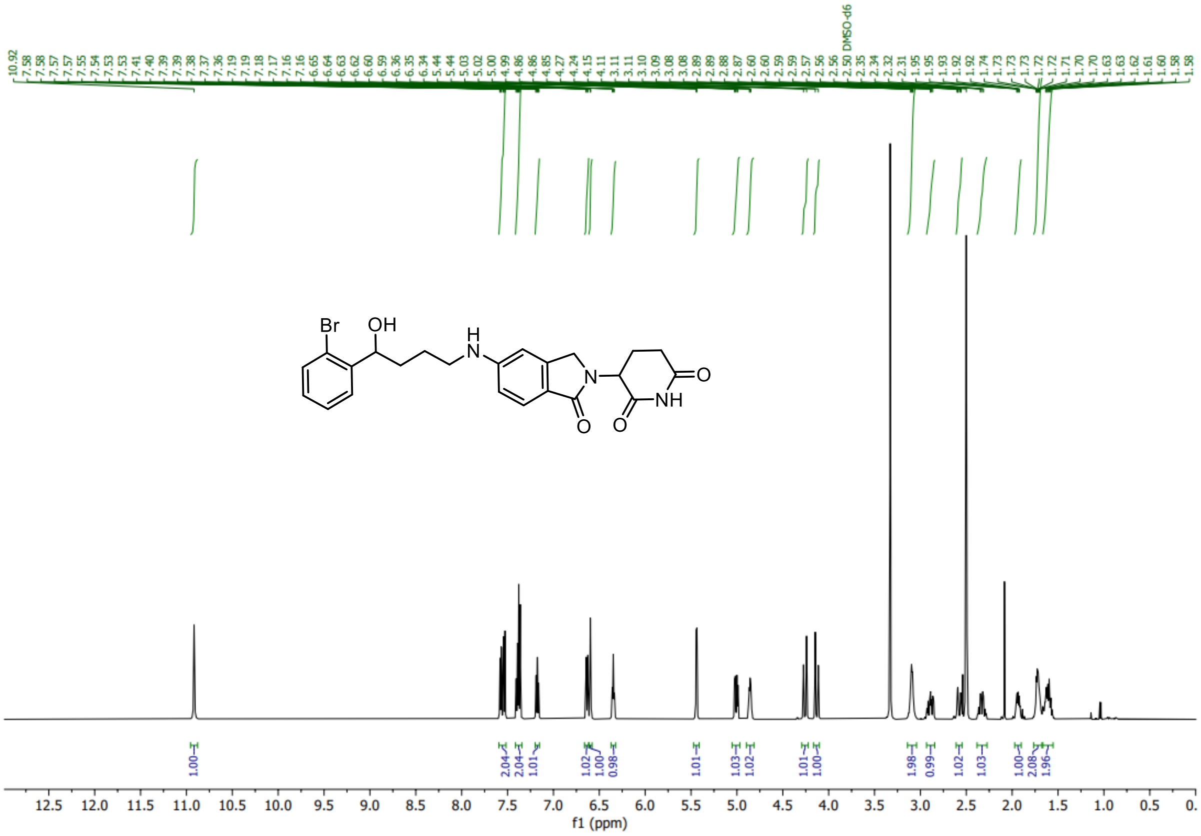

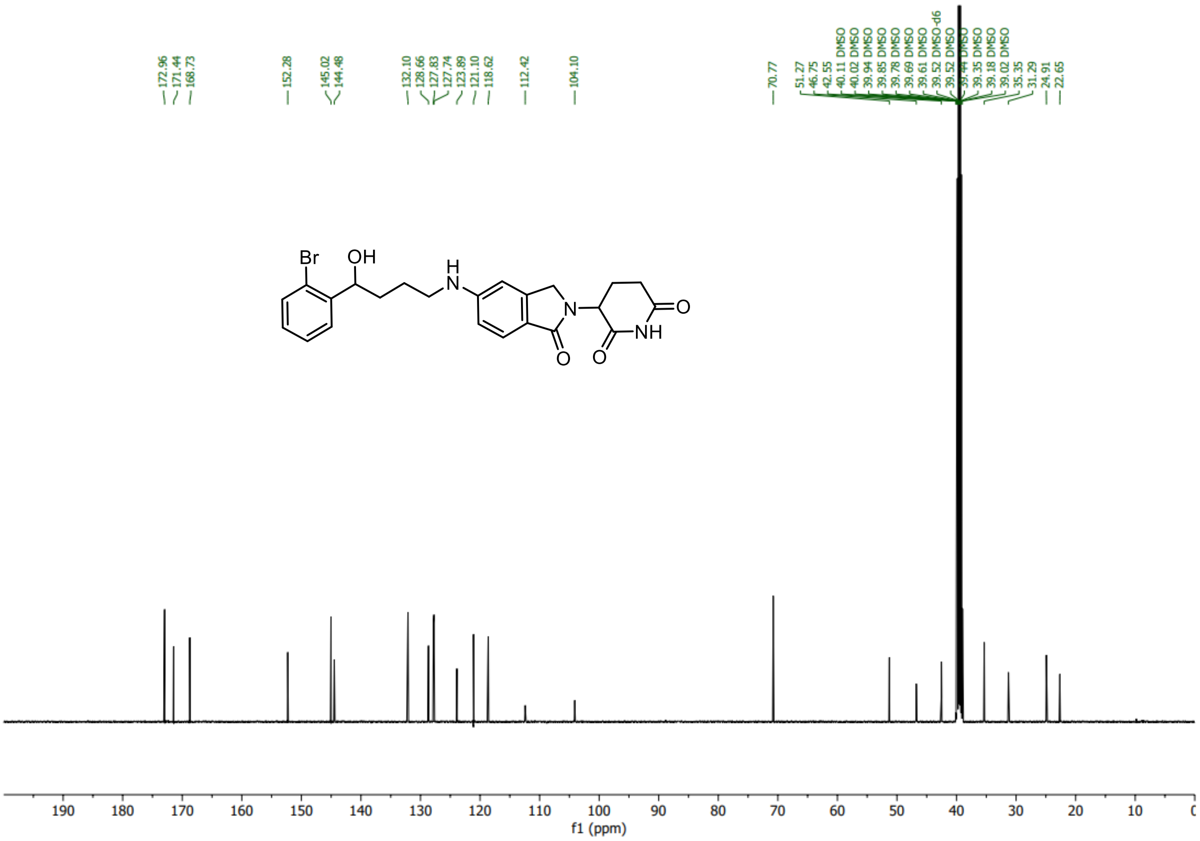

Figure S85. (Top) ^{1}H NMR (500 MHz) and (bottom) ^{13}C{^{1}H} NMR (126 MHz) spectra of **7cd** in DMSO-d_6.

3-(5-((4-(4-bromophenyl)-4-hydroxybutyl)amino)-1-oxoisoindolin-2-yl)piperidine-2,6-dione (7kd)

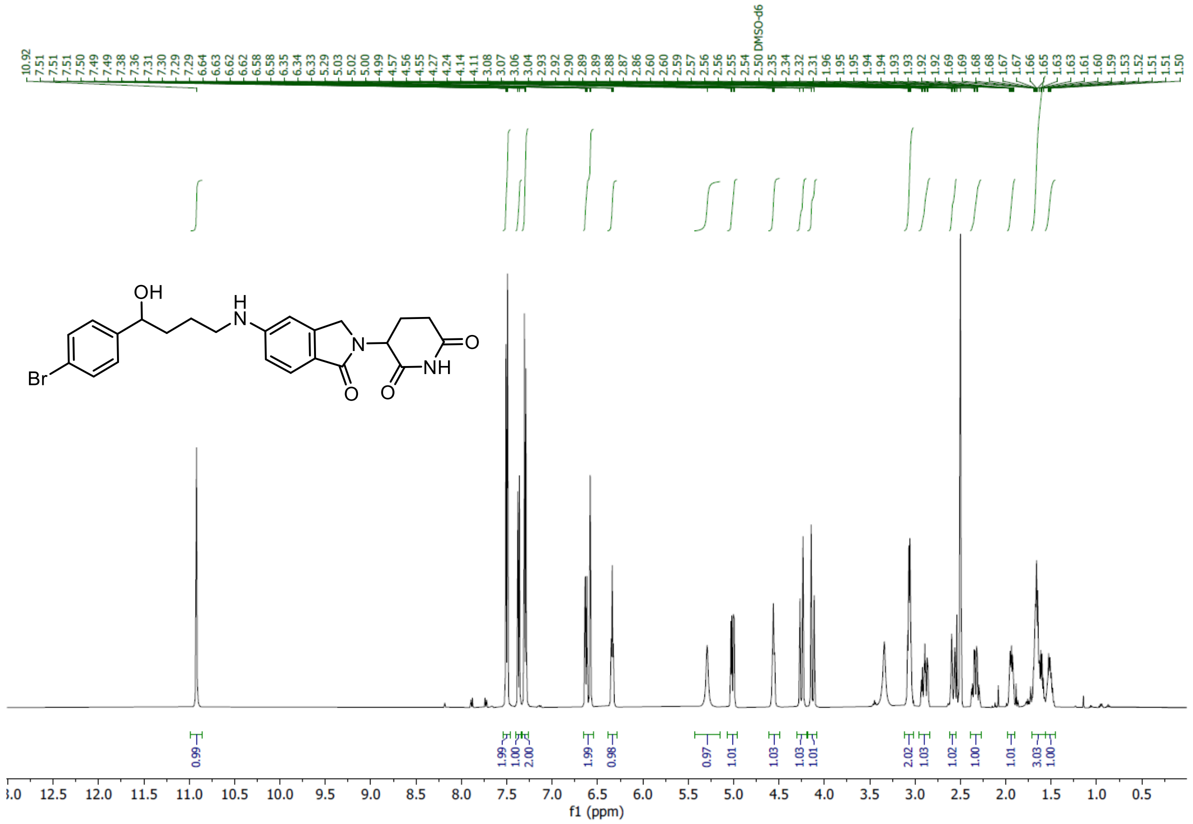

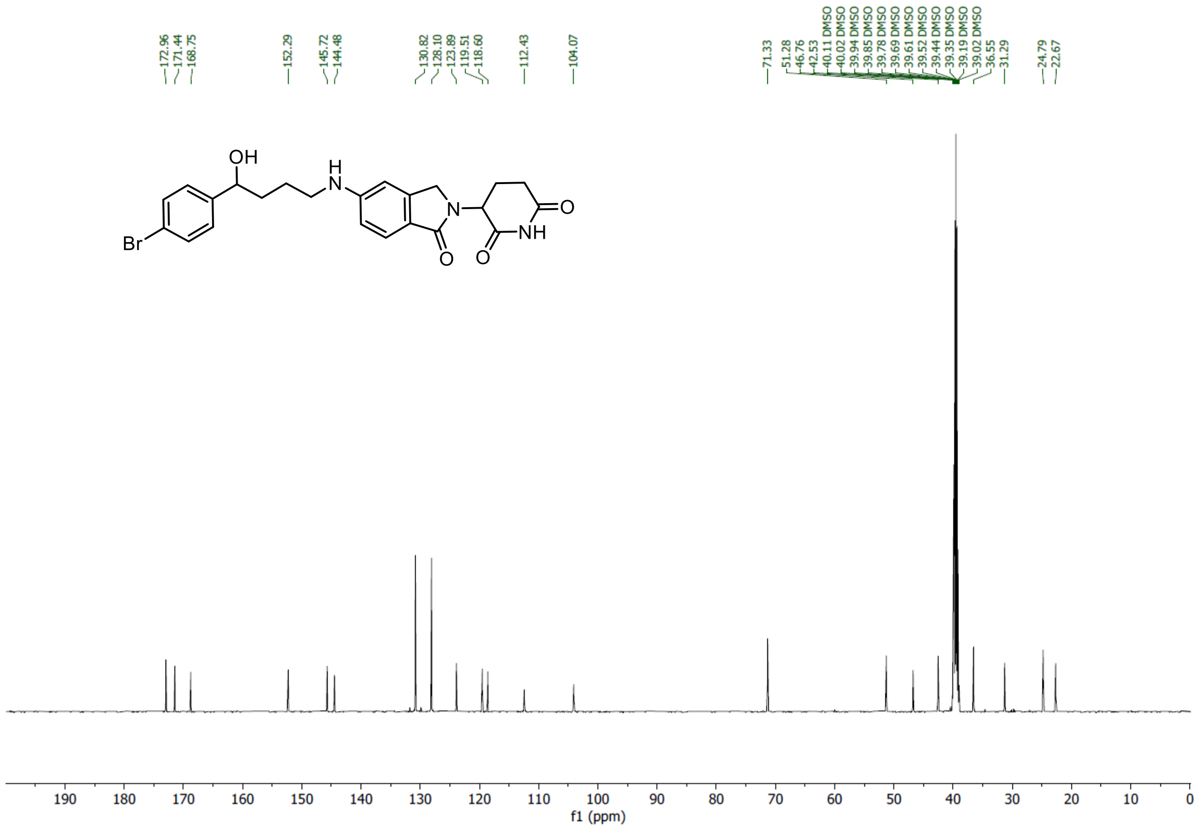

Figure S86. (Top) ^{1}H NMR (500 MHz) and (bottom) ^{13}C{^{1}H} NMR (126 MHz) spectra of **7kd** in DMSO-d_6.

3-(1-oxo-5-(2-(o-tolyl)pyrrolidin-1-yl)isoindolin-2-yl)piperidine-2,6-dione (7dd)

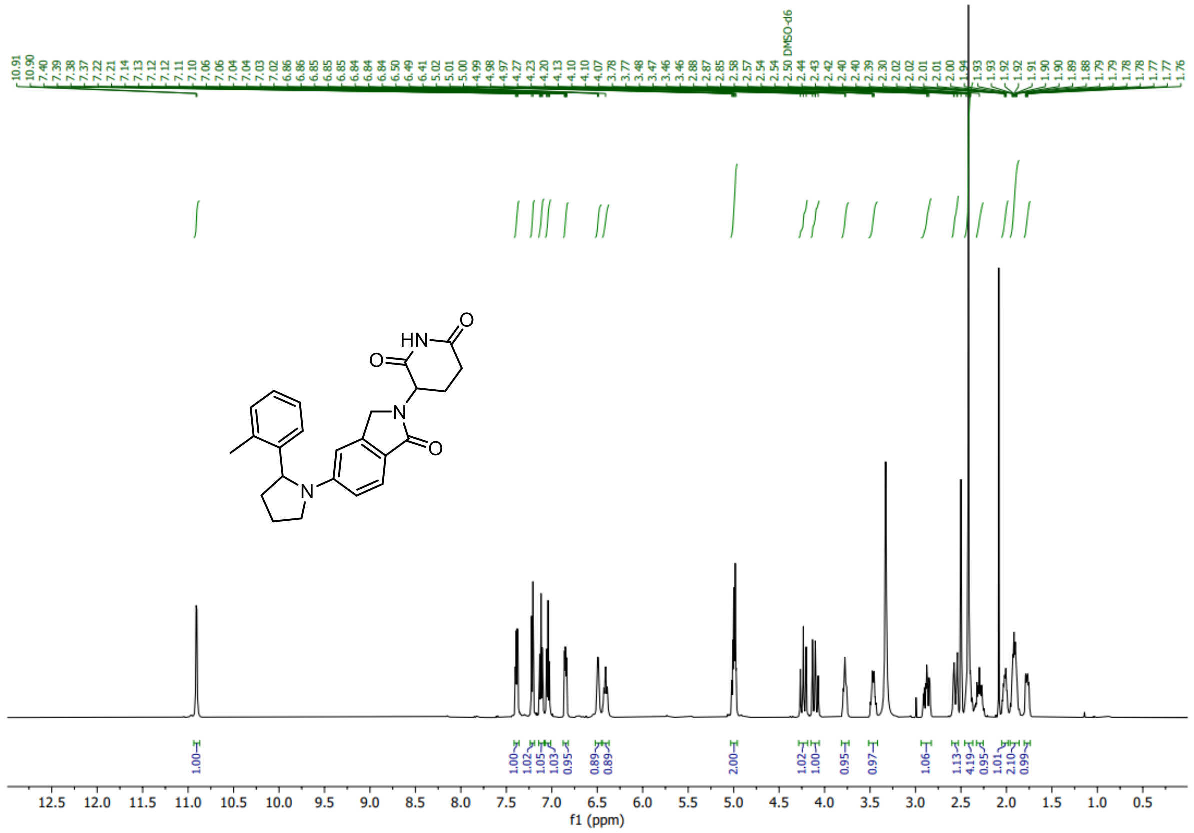

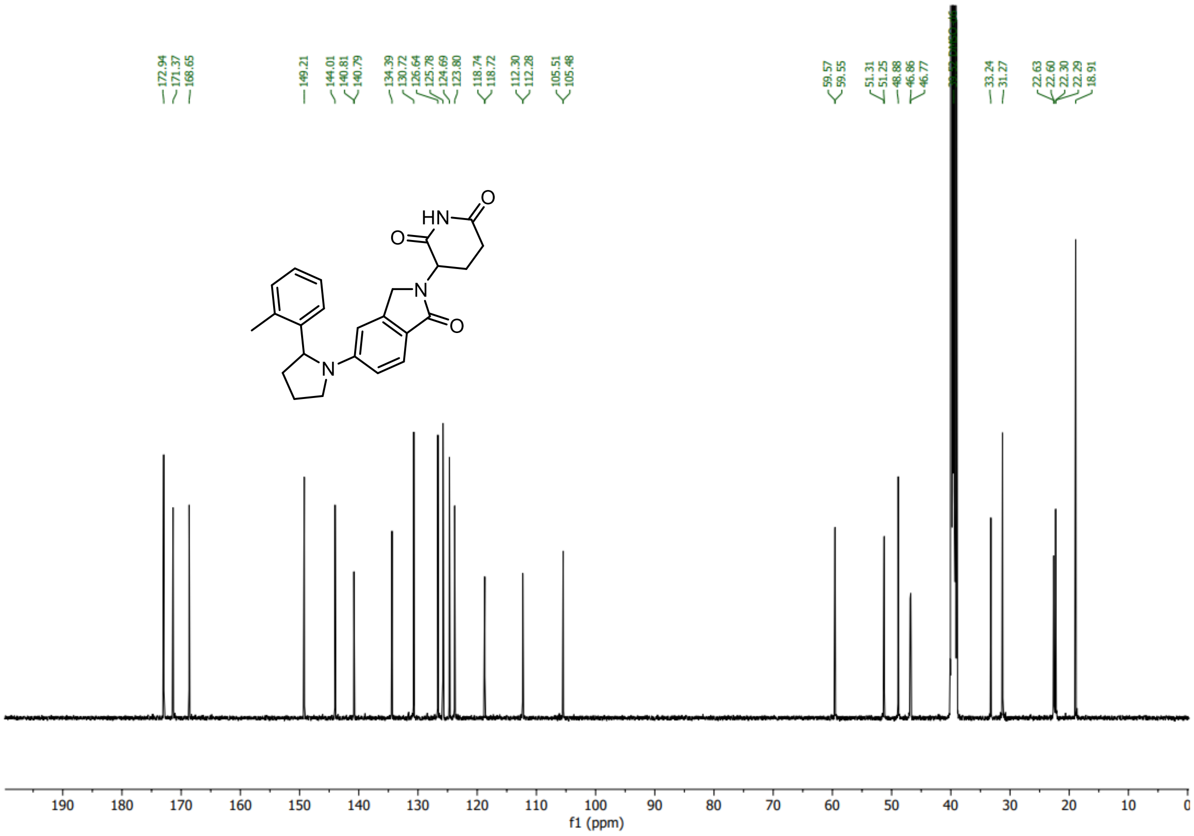

Figure S87. (Top) ^{1}H NMR (500 MHz) and (bottom) $^{13}C\{^{1}H\}$ NMR (126 MHz) spectra of **7dd** in DMSO-d_6.

1.7.4 Spectra of THF byproducts of Hammett plot (**8a – 8k**)

2-phenyltetrahydrofuran (8a)

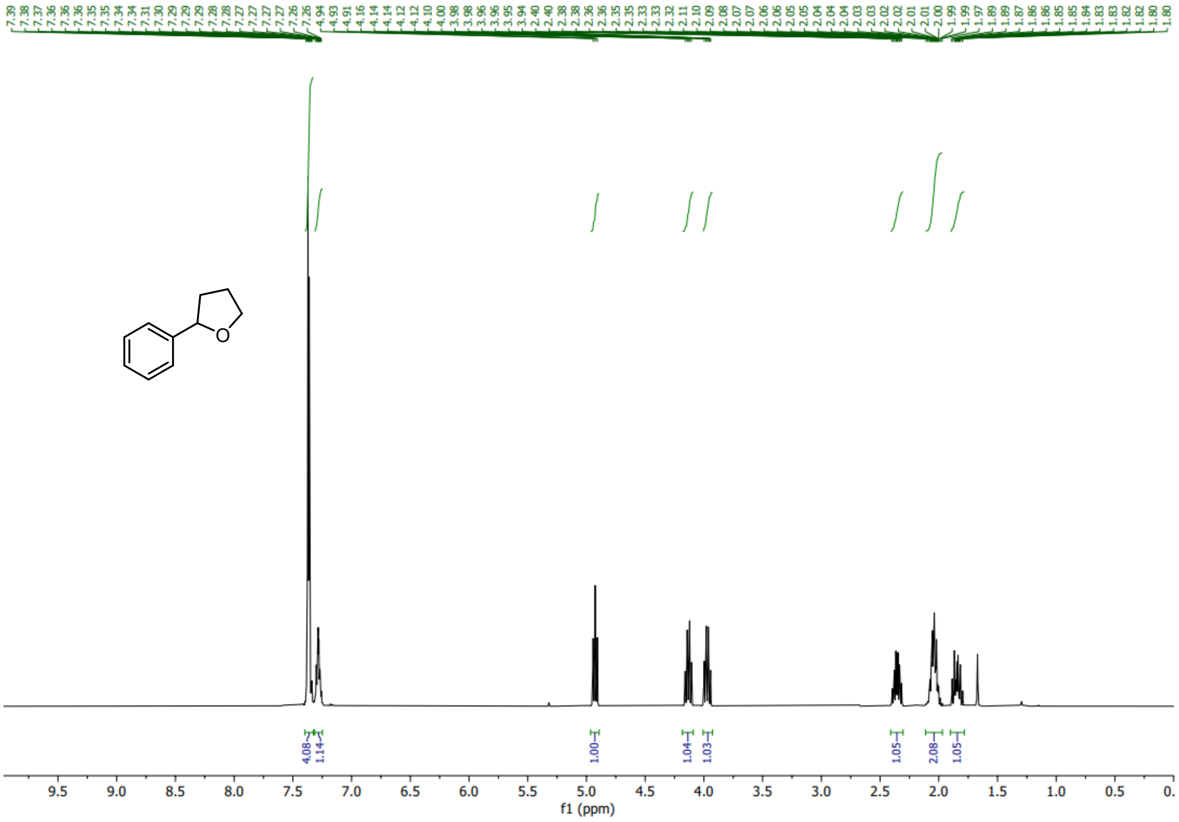

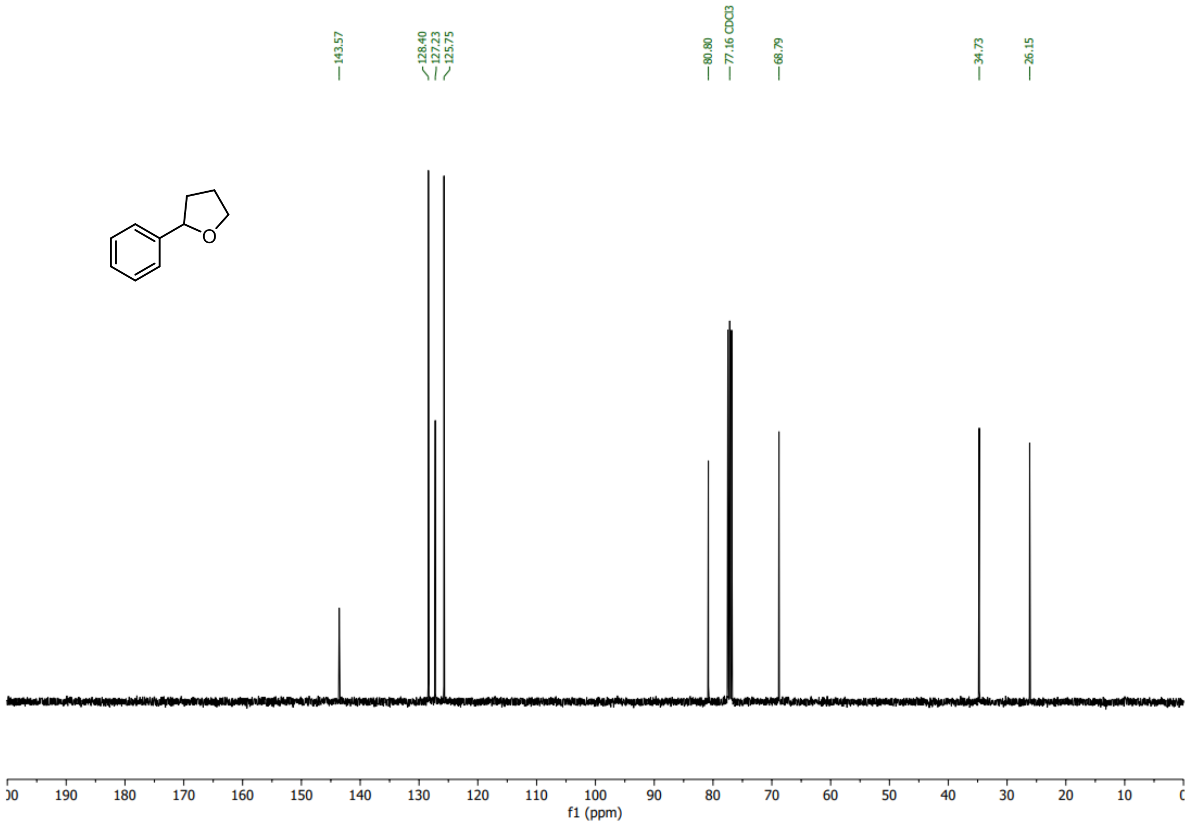

Figure S88. (Top) ^{1}H NMR (400 MHz) and (bottom) ^{13}C{^{1}H} NMR (101 MHz) spectra of **8a** in $CDCl_3$.

2-(p-tolyl)tetrahydrofuran (8l)

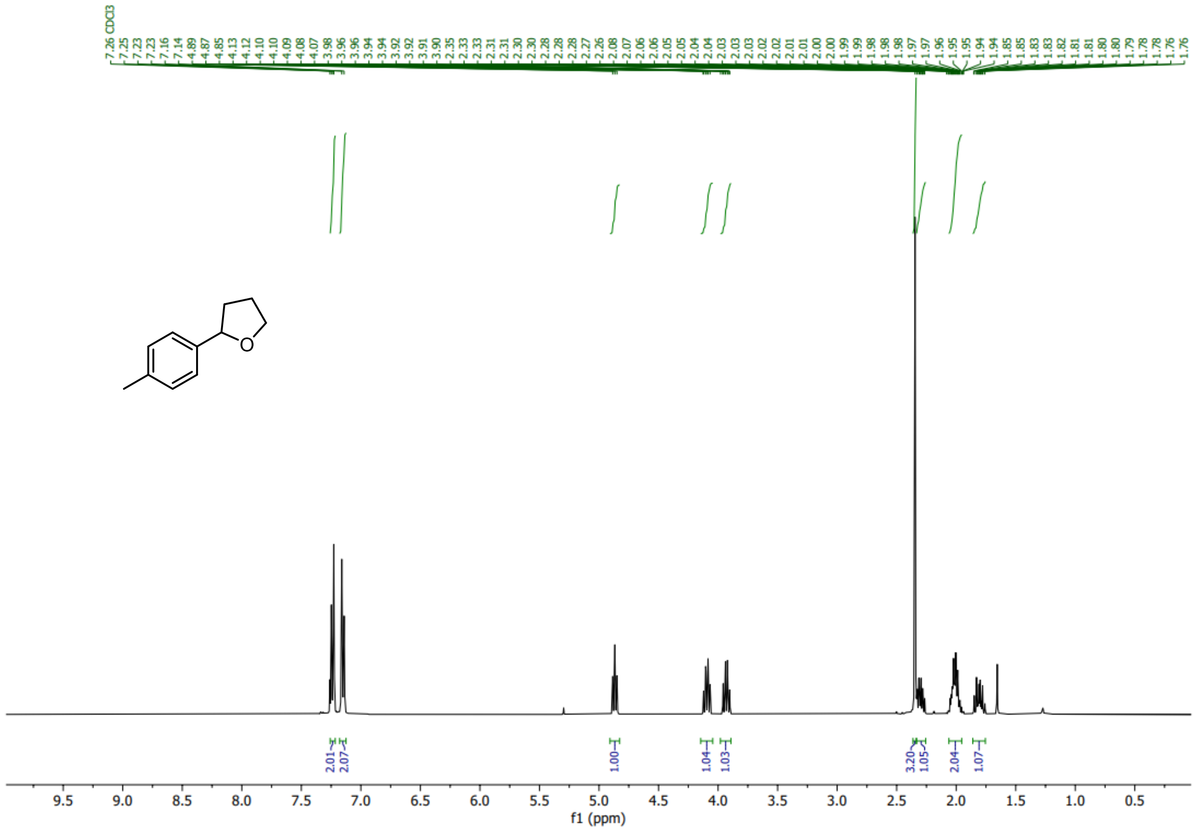

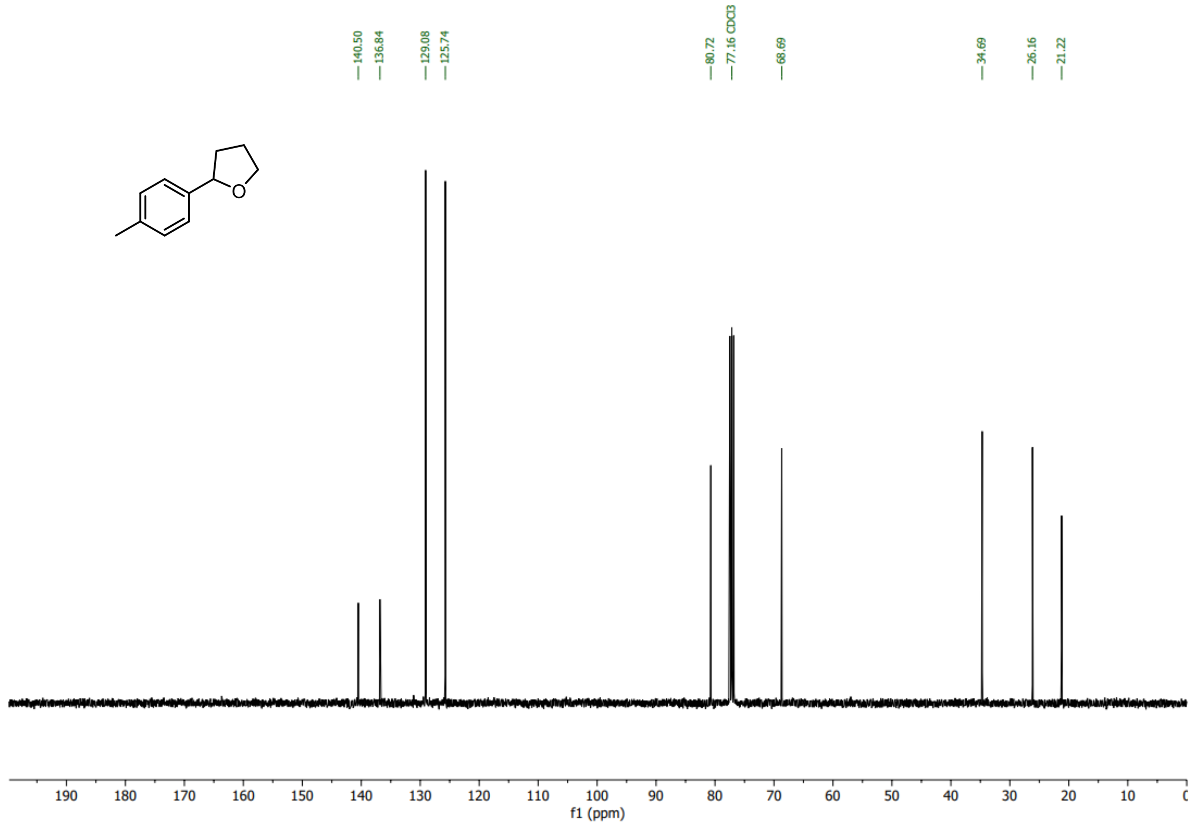

Figure S89. (Top) ^{1}H NMR (400 MHz) and (bottom) $^{13}C\{^{1}H\}$ NMR (101 MHz) spectra of **8l** in $CDCl_3$.

2-(4-fluorophenyl)tetrahydrofuran (8i)

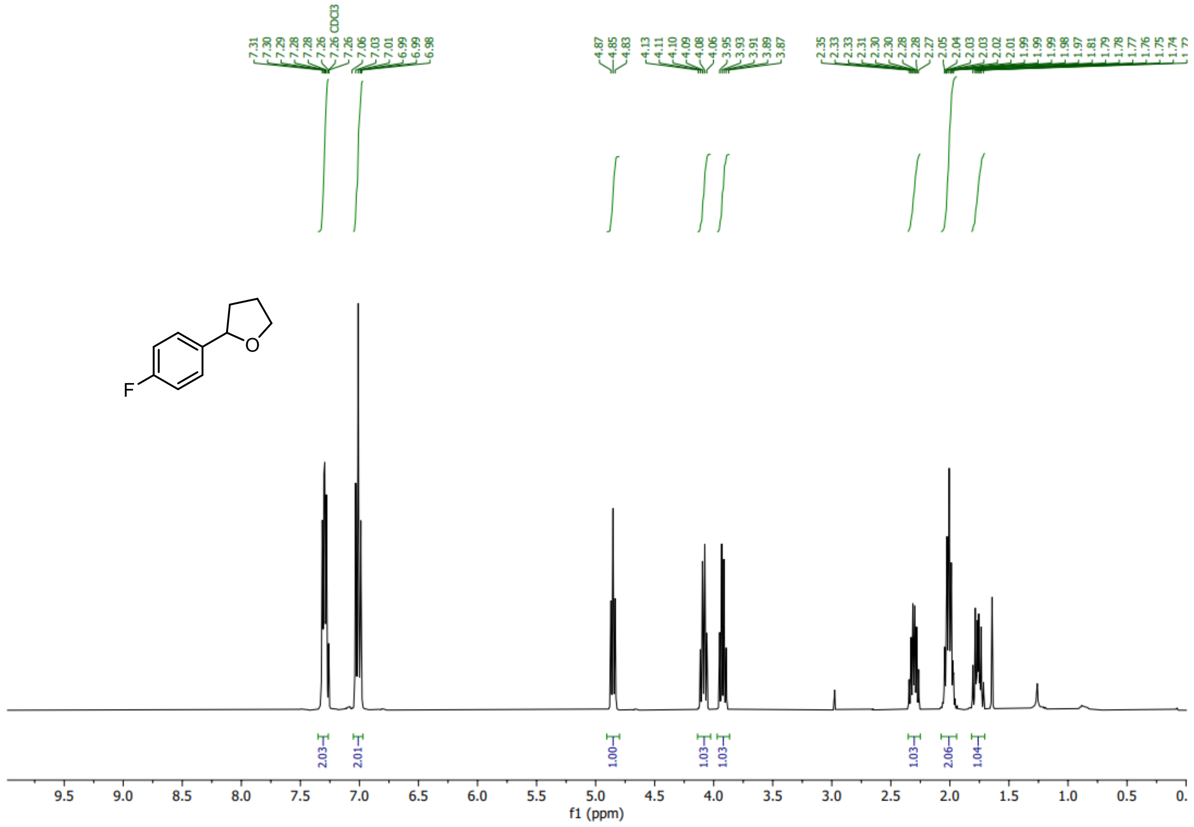

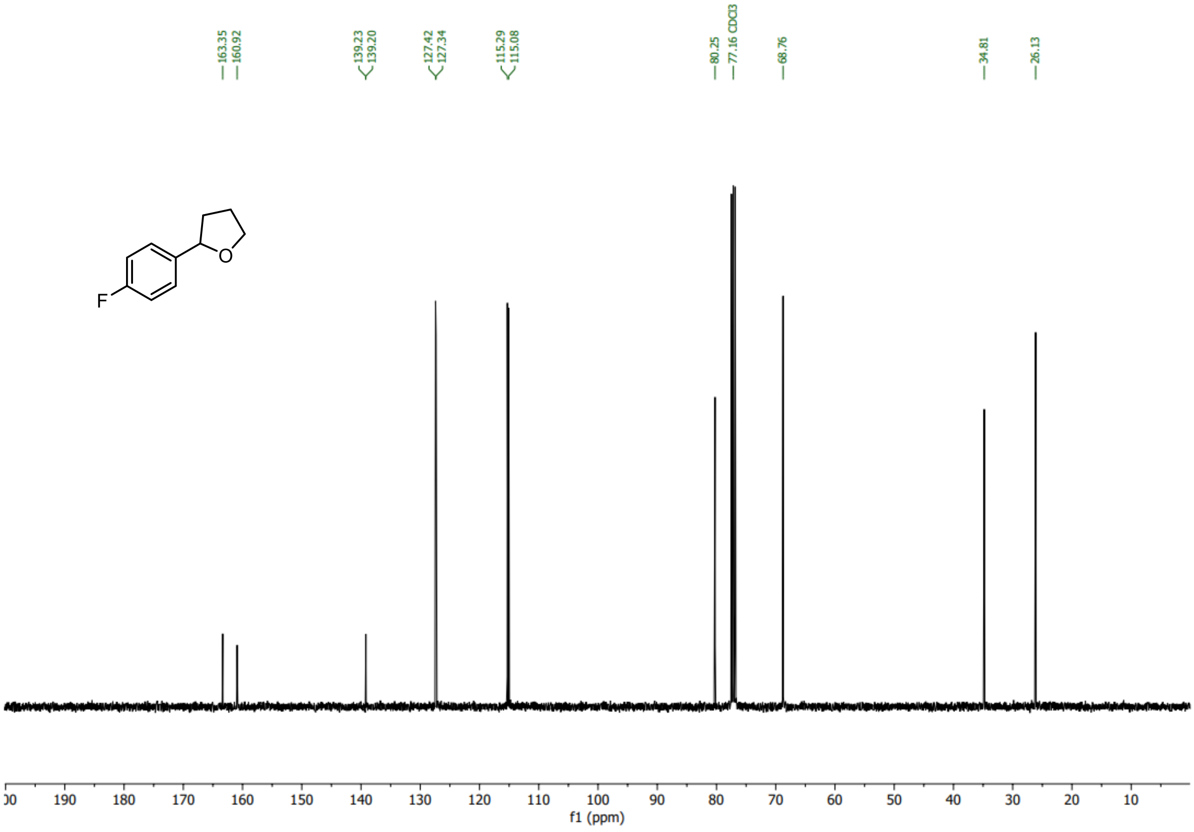

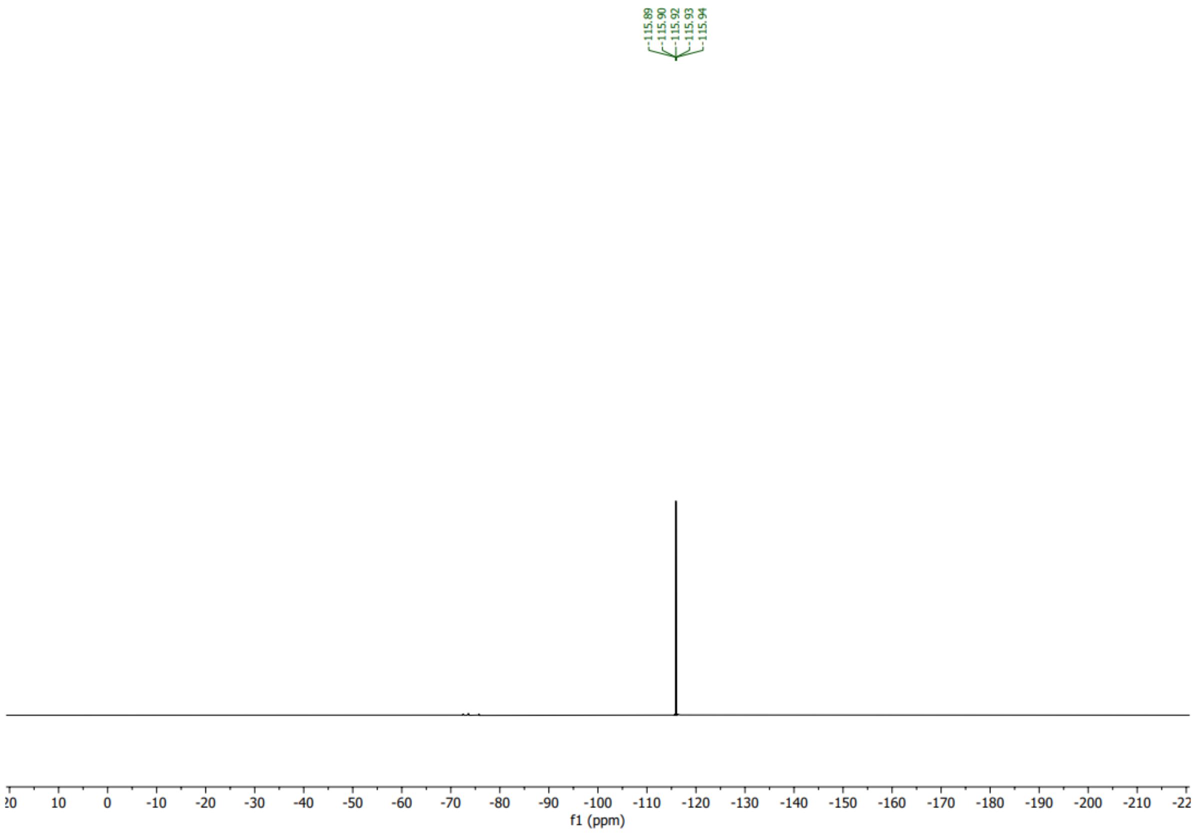

Figure S90. (Top) ^1H NMR (400 MHz), (centre) $^{13}C\{^1H\}$ NMR (101 MHz) and (bottom) ^{19}F NMR (377 MHz) spectra of **8i** in $CDCl_3$

2-(4-chlorophenyl)tetrahydrofuran (8j)

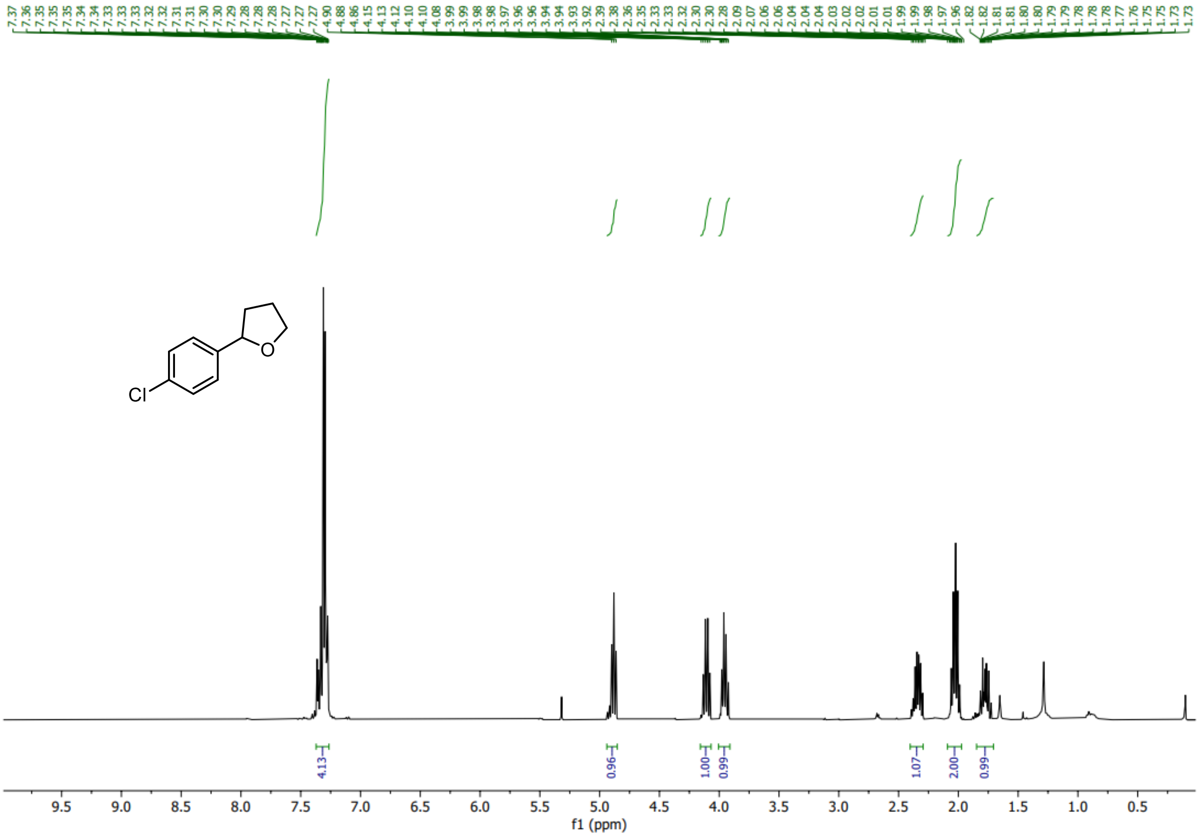

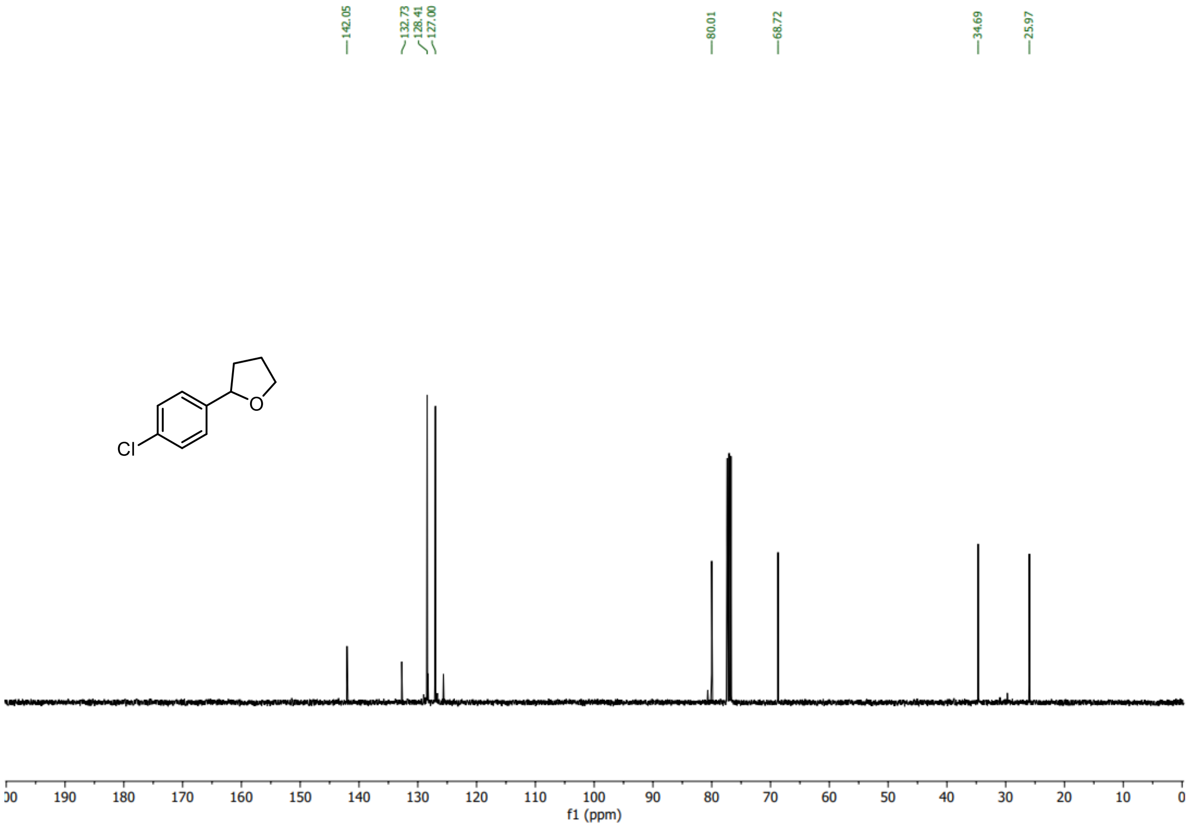

Figure S91. (Top) ^{1}H NMR (400 MHz) and (bottom) $^{13}C\{^{1}H\}$ NMR (101 MHz) spectra of **8j** in $CDCl_3$.

2-(4-chlorophenyl)tetrahydrofuran (8k)

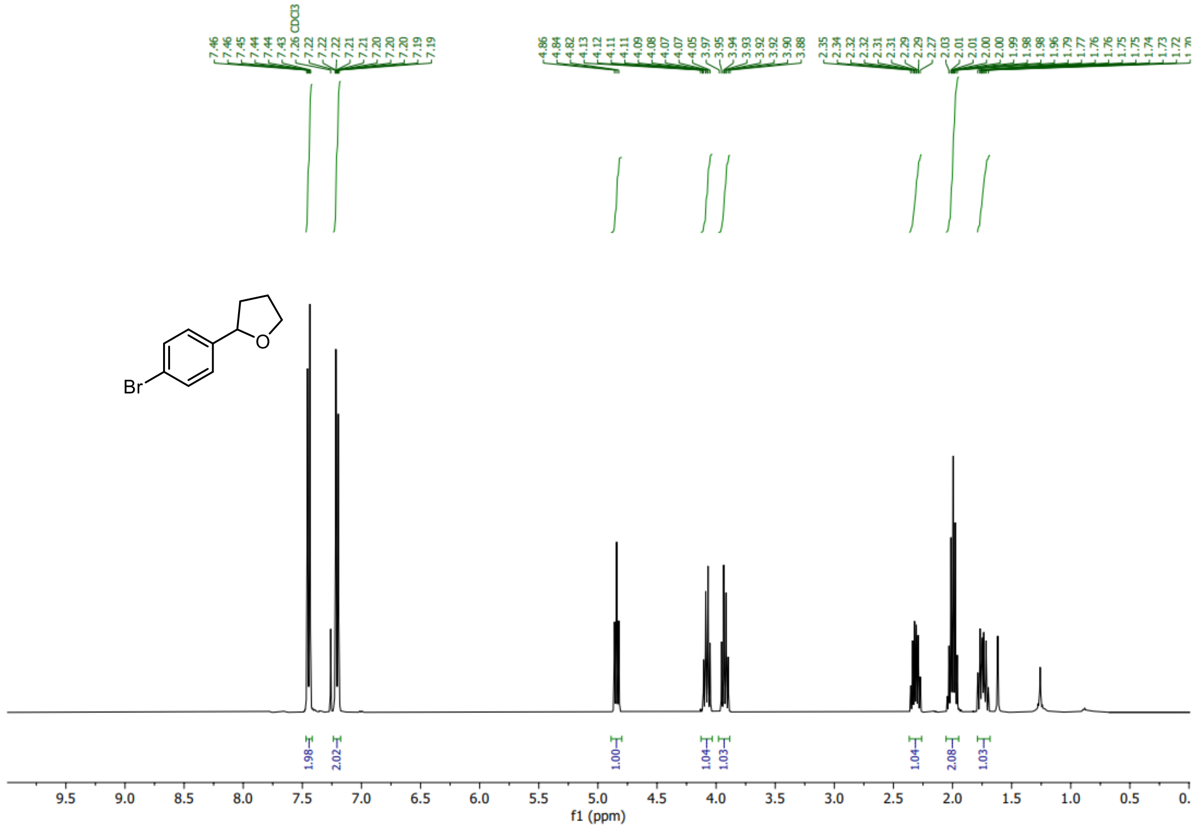

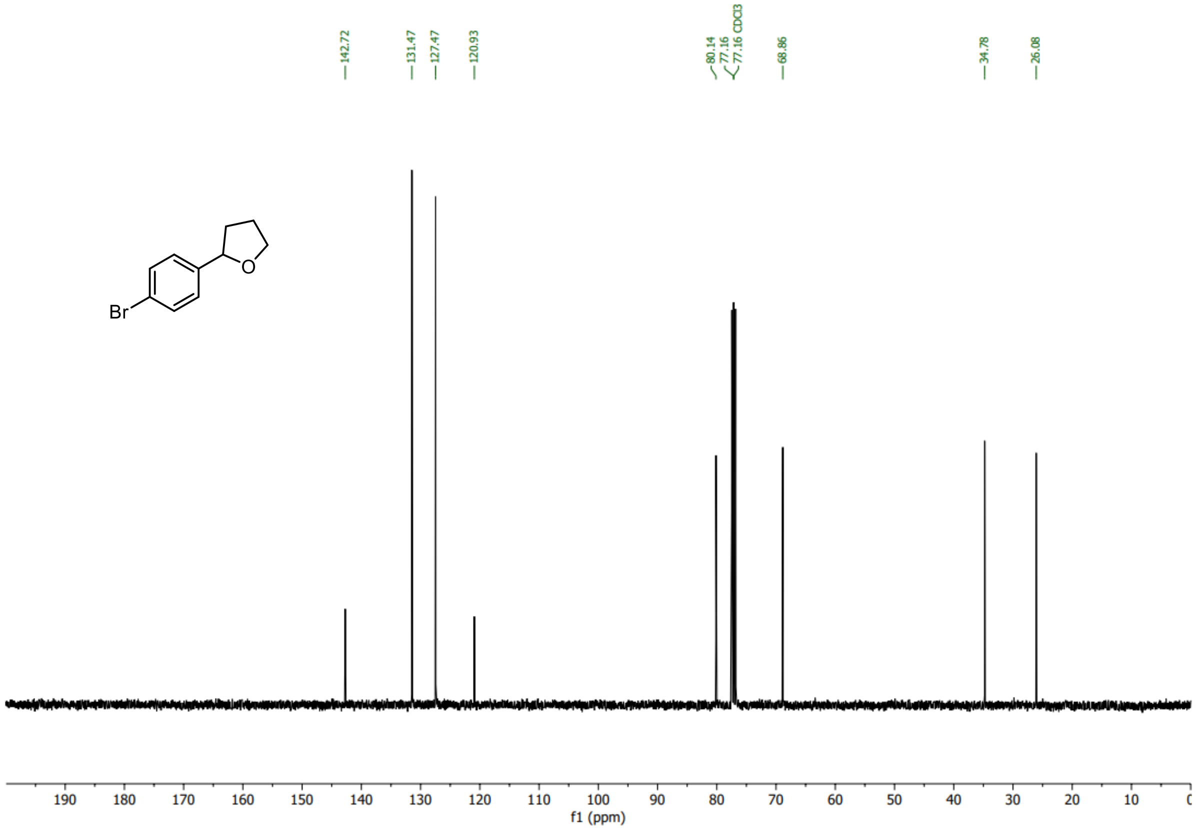

Figure S92. (Top) ^{1}H NMR (400 MHz) and (bottom) ^{13}C{^{1}H} NMR (101 MHz) spectra of **8k** in $CDCl_3$.

1.8 SFC chromatogram of chiral compound: *ee* determination

1.8.1 SFC chromatogram of starting material

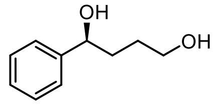

(*S*)-1-phenylbutane-1,4-diol [(*S*)-1a]

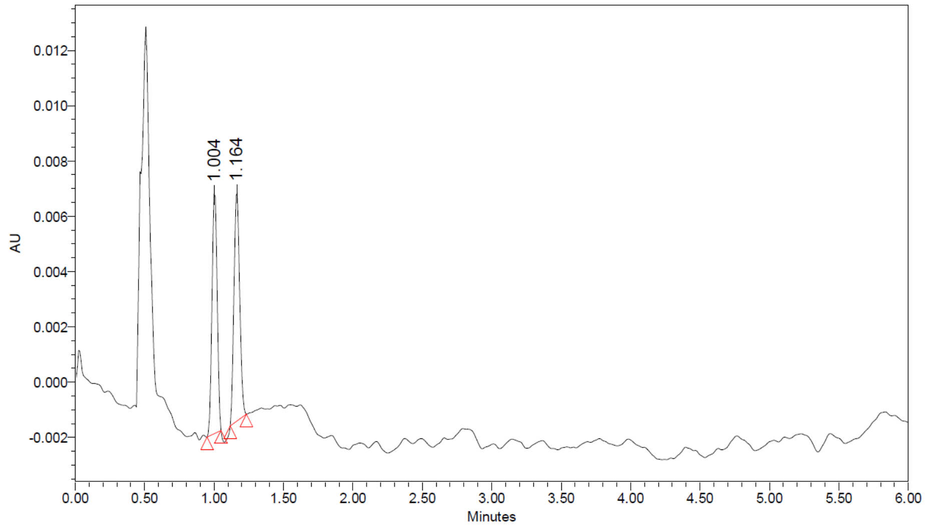

	Retention Time	Area	% Area	k'	N	USP Resolution	Width @ 50%
1	1.004	22493	48.72	0.000	3361.8		4.123167e-02
2	1.164	23675	51.28	0.159	3667.3	2.135	4.510835e-02

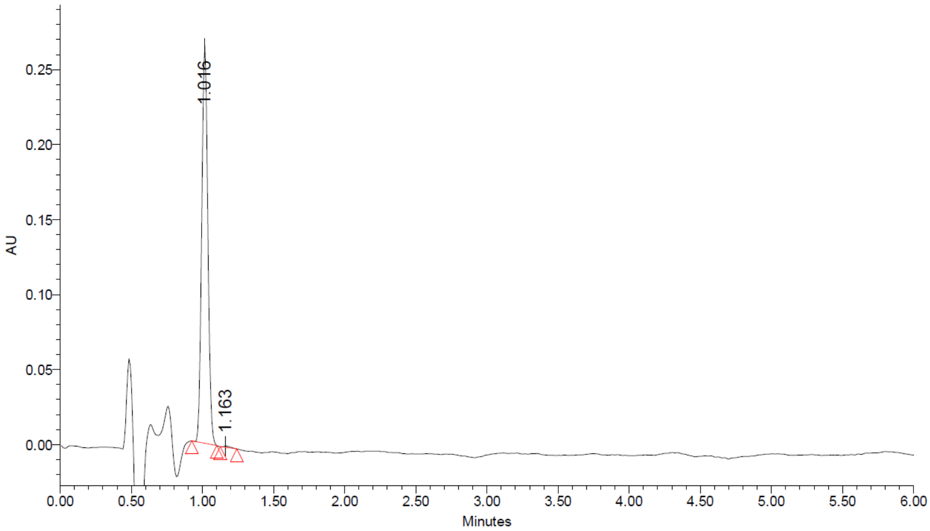

	Retention Time	Area	% Area	k'	N	USP Resolution
1	1.016	788994	99.60	0.000	2605.4	
2	1.163	3166	0.40	0.145	2635.2	1.659

ee = 99.2

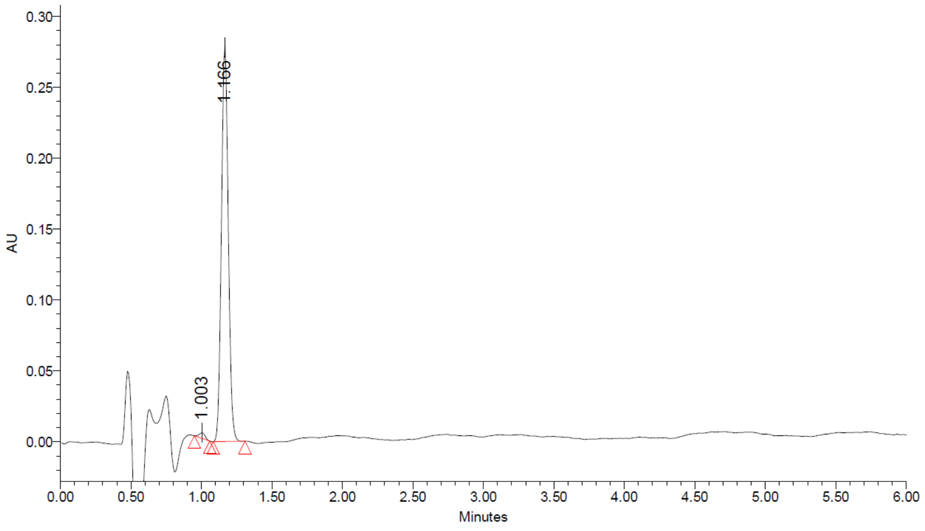

	Retention Time	Area	% Area	k'	N	USP Resolution
1	1.003	11593	1.22	0.000	2660.5	
2	1.166	942572	98.78	0.162	2701.1	1.914

ee = 97.6

Figure S93. SFC chromatogram of the racemic mixture of **1a** (up), isomer 1 (middle), isomer 2 (down).

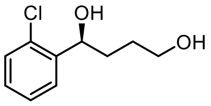

(*S*)-1-(2-chlorophenyl)butane-1,4-diol [(*S*)-1b]

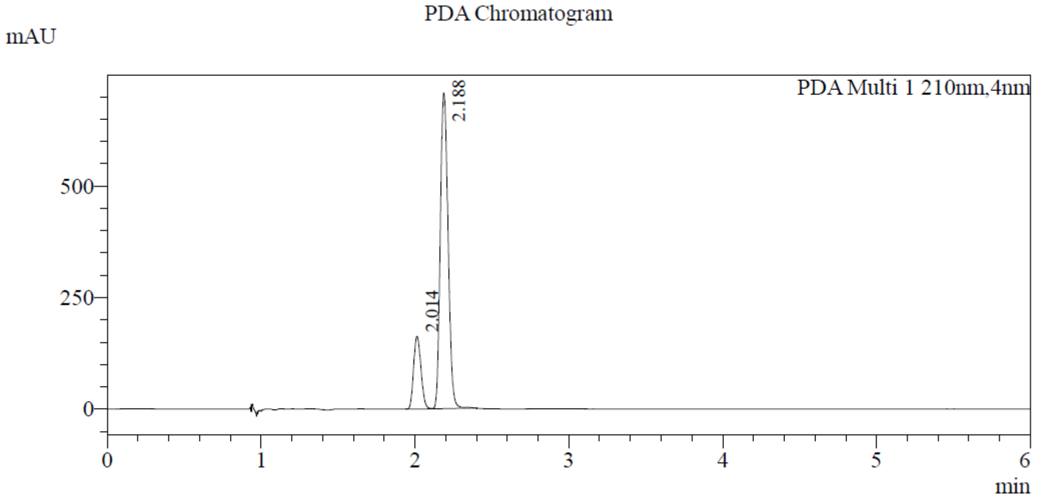

Peak Table

PDA Ch1 210nm

Peak#	Ret. Time	Area	Height	Area%
1	2.014	542748	161536	17.861
2	2.188	2495974	703678	82.139

64.3 ee

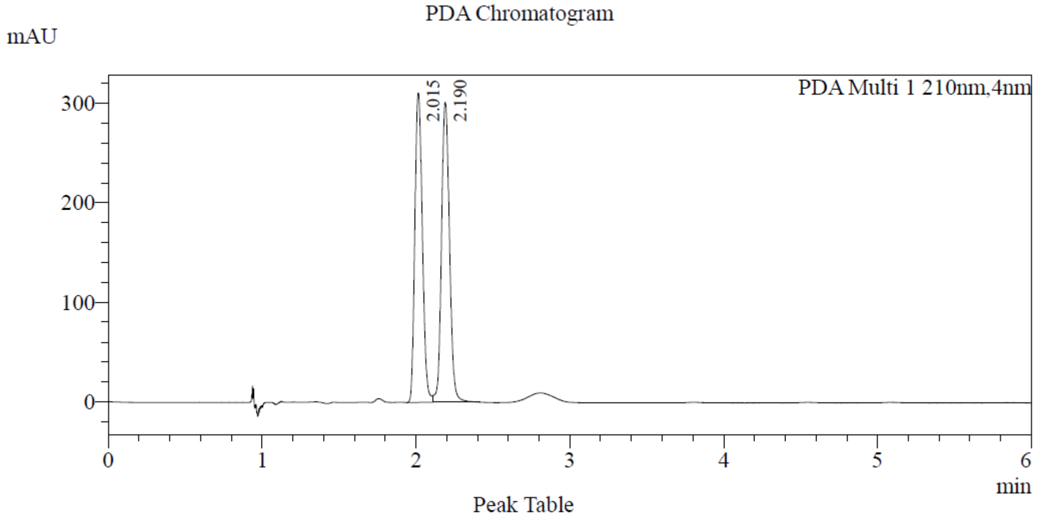

Peak Table

PDA Ch1 210nm

Peak#	Ret. Time	Area	Height	Area%
1	2.015	1056880	307359	49.087
2	2.190	1096202	298254	50.913

Figure S94. SFC chromatogram of **(*S*)-1b**, chiral (up), racemic (down).

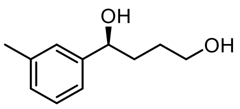

(*S*)-1-(m-tolyl)butane-1,4-diol [(*S*)-1g]

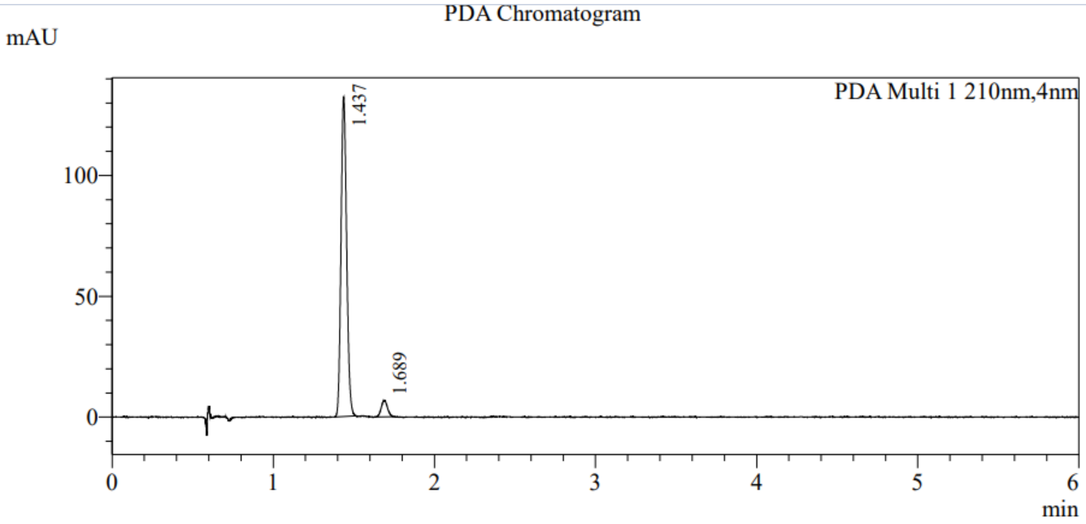

Peak Table

PDA Ch1 210nm

Peak#	Ret. Time	Height	Area	Area%
1	1.437	130744	320593	94.559
2	1.689	6752	18446	5.441

89% ee

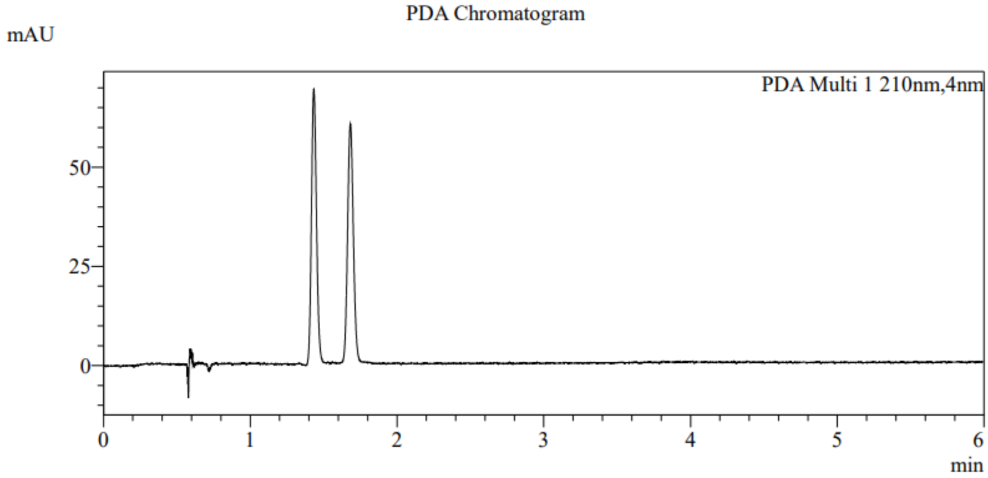

Figure S95. SFC chromatogram of **(*S*)-1g**, chiral (up), racemic (down).

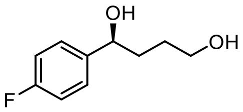

(*S*)-1-(4-fluorophenyl)butane-1,4-diol [(*S*)-1i]

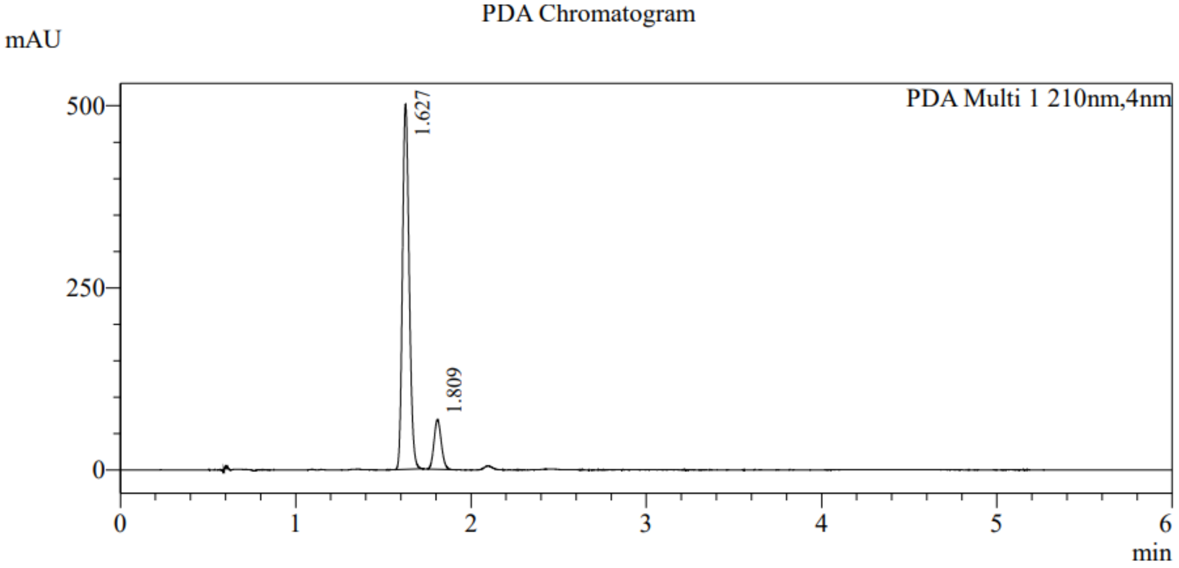

Peak Table

PDA Ch1 210nm

Peak#	Ret. Time	Height	Area	Area%
1	1.627	491721	1333348	87.121
2	1.809	66340	197116	12.879

74% ee

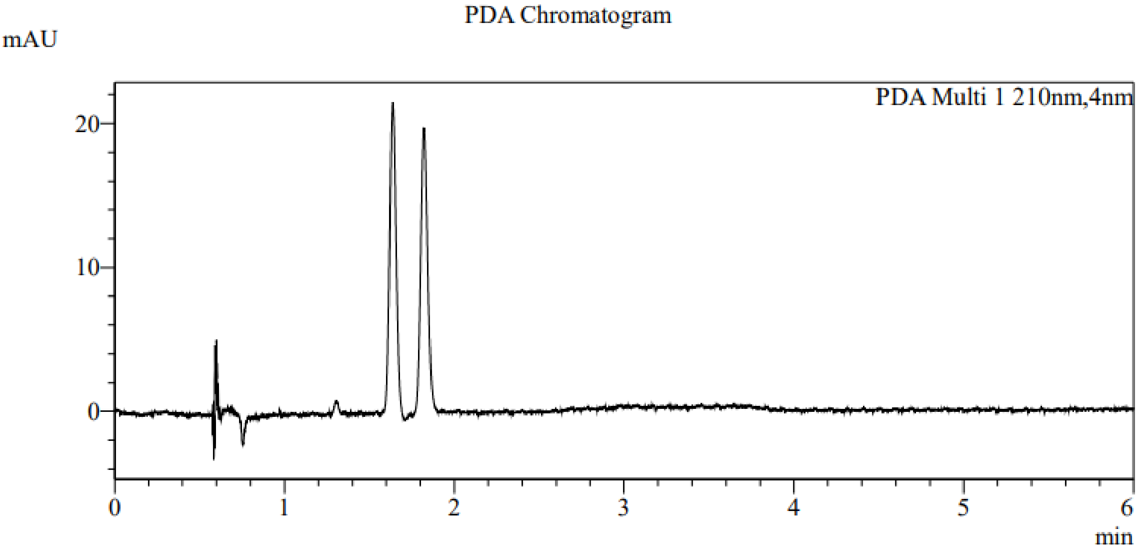

Figure S96. SFC chromatogram of **(*S*)-1i**, chiral (up), racemic (down).

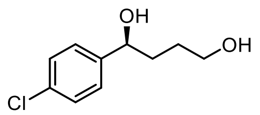

(*S*)-1-(4-chlorophenyl)butane-1,4-diol [(*S*)-1j]

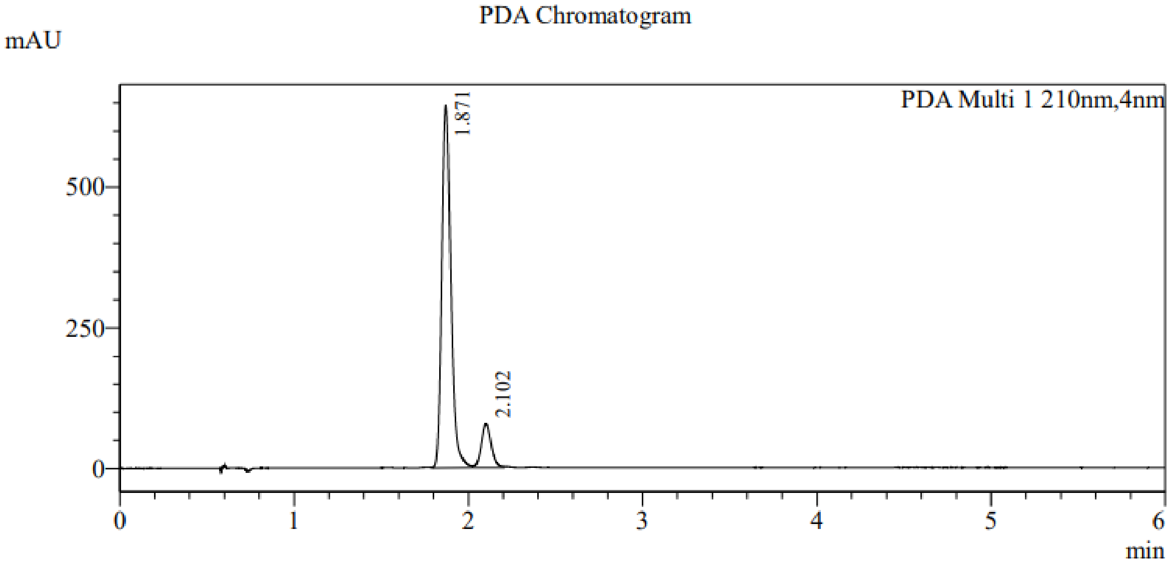

Peak Table

PDA Ch1 210nm

Peak#	Ret. Time	Height	Area	Area%
1	1.871	641296	2336940	88.154
2	2.102	77885	314031	11.846

76% ee

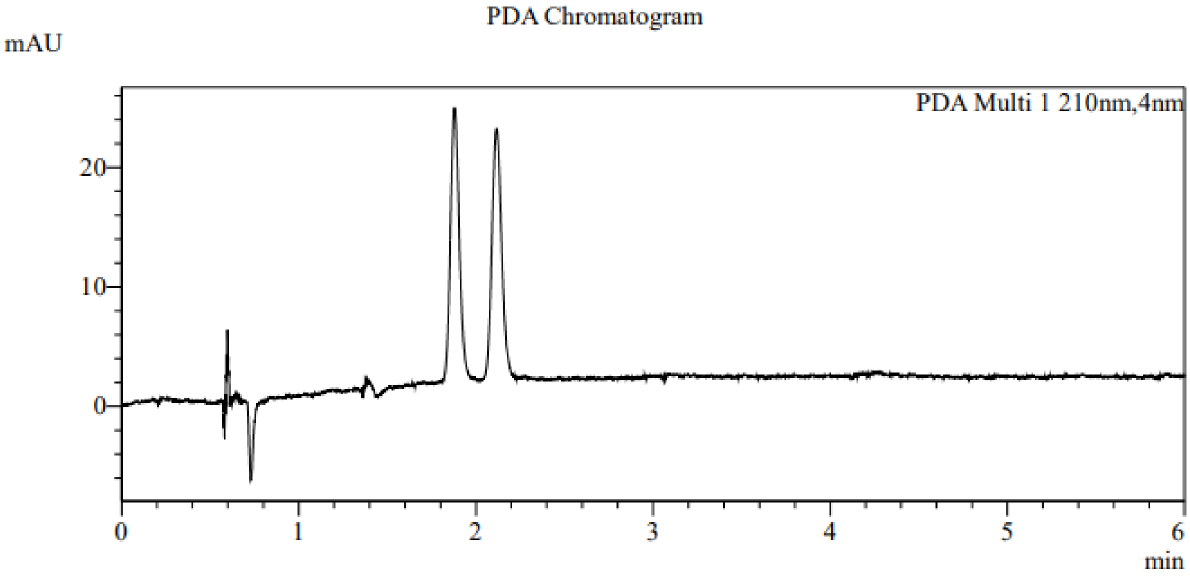

Figure S97. SFC chromatogram of **(*S*)-1j**, chiral (up), racemic (down).

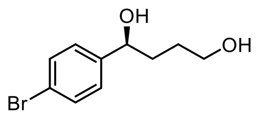

(*S*)-1-(4-bromophenyl)butane-1,4-diol [(*S*)-1k]

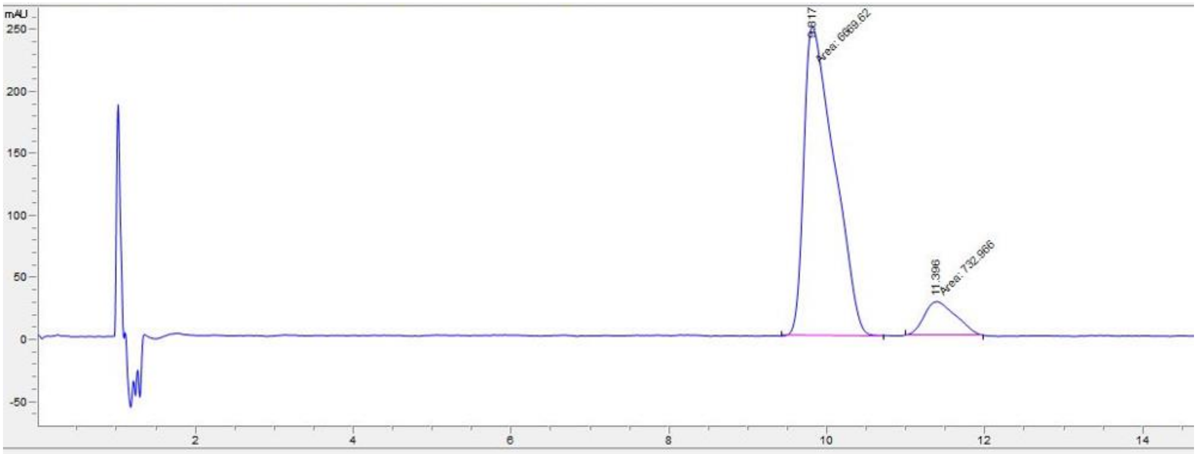

#	Time	Type	Area	Height	Width	Area%	Symmetry
1	9.817	MM	6669.6	248.9	0.4465	90.099	0.383
2	11.396	MM	733	26.6	0.4592	9.901	0.659

80% ee

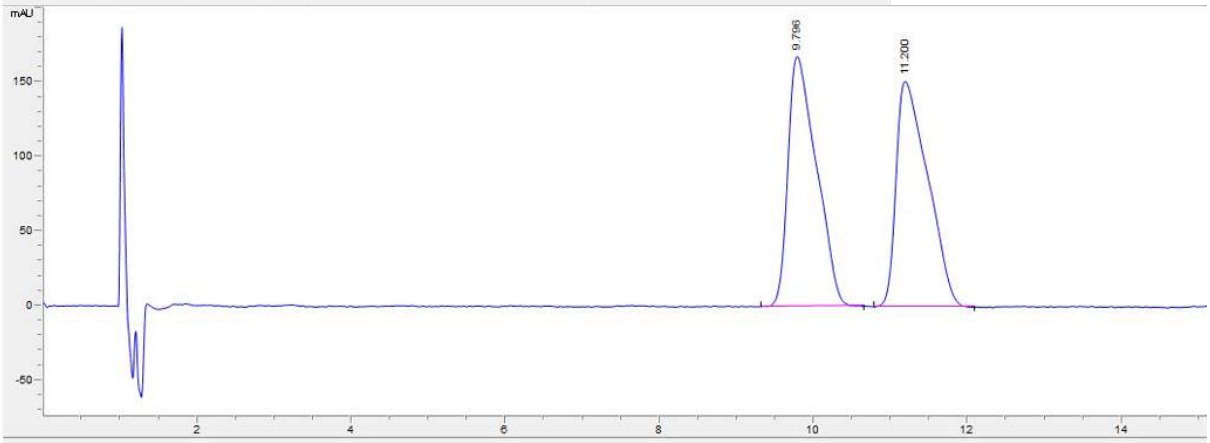

#	Time	Type	Area	Height	Width	Area%	Symmetry
1	9.796	VV R	4327.4	168.3	0.3721	49.882	0.463
2	11.2	BV R	4347.8	151.9	0.402	50.118	0.393

Figure S98. SFC chromatogram of **(*S*)-1k**, chiral (up), racemic (down).

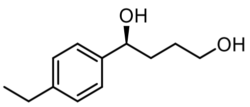

(*S*)-1-(4-ethylphenyl)butane-1,4-diol [(*S*)-1n]

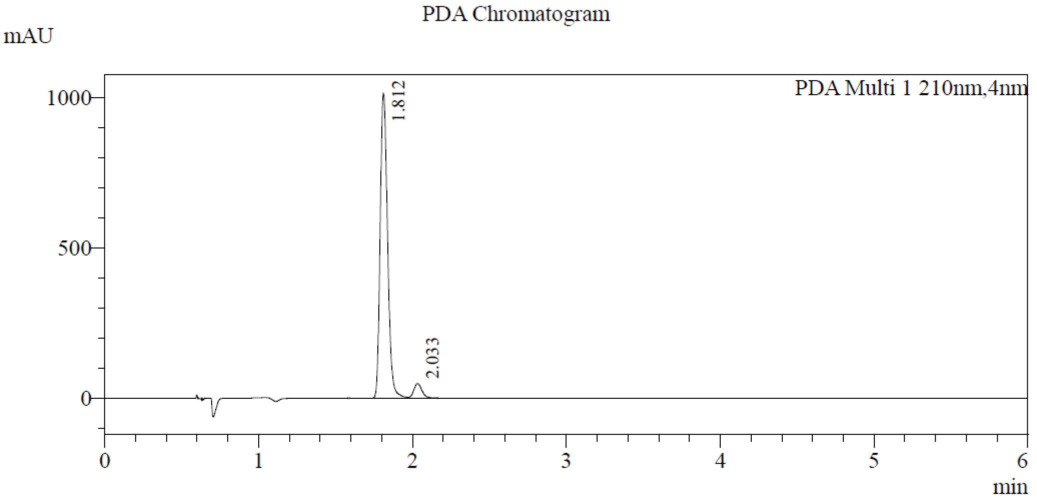

Peak Table

PDA Ch1 210nm

Peak#	Ret. Time	Area	Height	Area%
1	1.812	3386800	1008162	94.949
2	2.033	180186	47770	5.051

90% ee

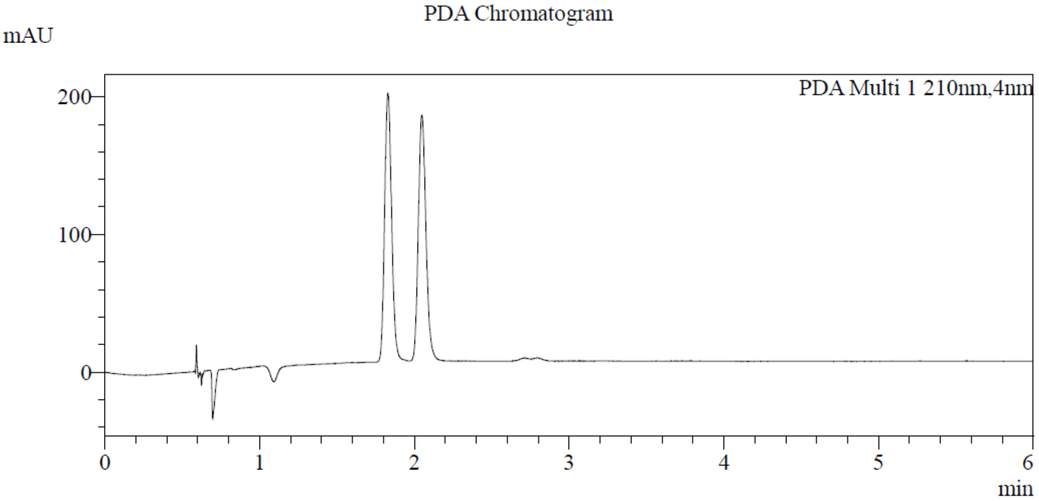

Figure S99. SFC chromatogram of **(*S*)-1n**, chiral (up), racemic (down).

1.8.2 SFC chromatogram of products

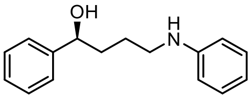

(*S*)-1-phenyl-4-(phenylamino)butan-1-ol [(*S*)-3aa]

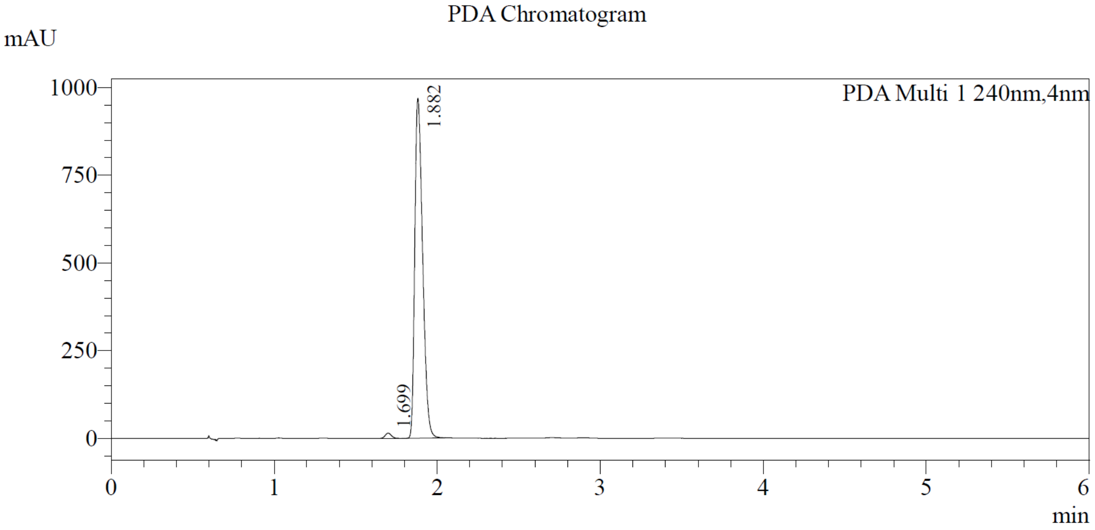

Peak Table

PDA Ch1 240nm

Peak#	Ret. Time	Area	Height	Area%
1	1.699	39453	14631	1.201
2	1.882	3246171	956784	98.799

98% ee

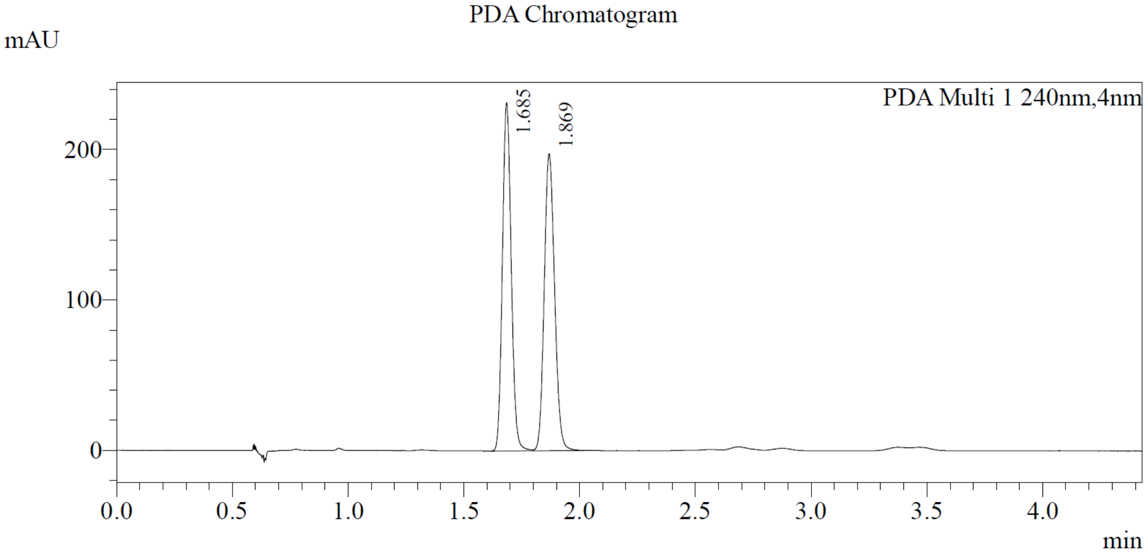

Peak Table

PDA Ch1 240nm

Peak#	Ret. Time	Area	Height	Area%
1	1.685	612420	226116	49.931
2	1.869	614118	194584	50.069

Figure S100. SFC chromatogram of **(*S*)-3aa**, chiral (up), racemic (down).

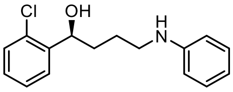

(*S*)-1-(2-chlorophenyl)-4-(phenylamino)butan-1-ol [(*S*)-3ba]

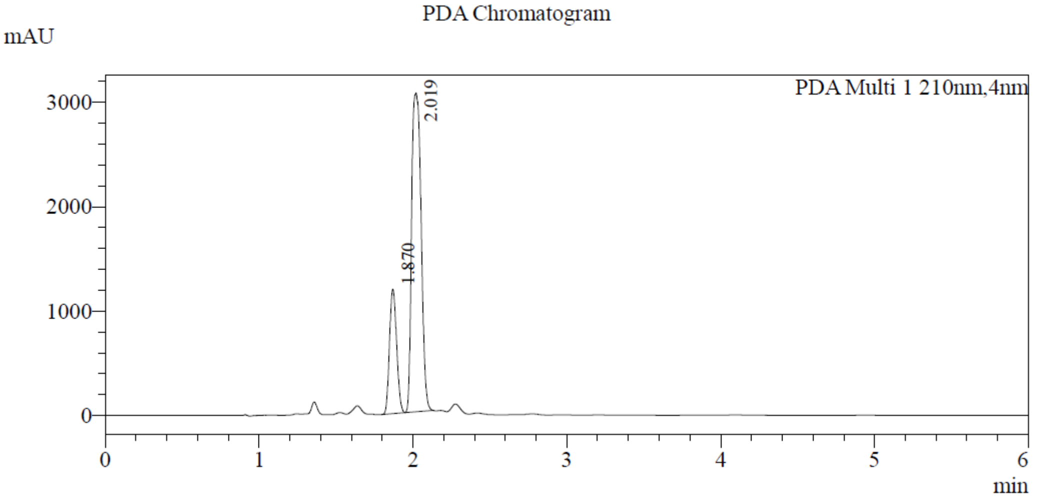

Peak Table

PDA Ch1 210nm

Peak#	Ret. Time	Area	Height	Area%
1	1.870	3853217	1181906	22.762
2	2.019	13074769	3045255	77.238

55% ee

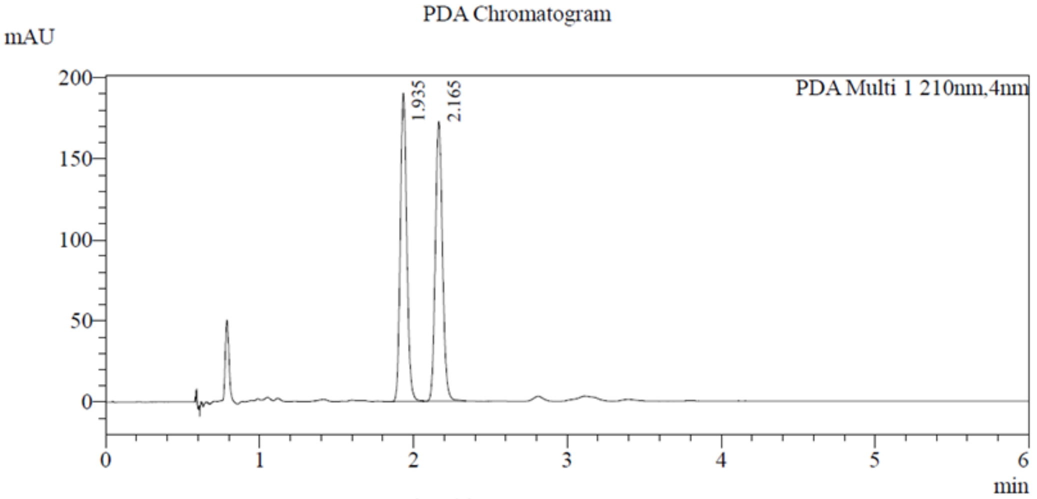

Peak Table

PDA Ch1 210nm

Peak#	Ret. Time	Area	Height	Area%
1	1.935	556574	186239	49.946
2	2.165	557769	170743	50.054

Figure S101. SFC chromatogram of **(*S*)-3ba**, chiral (up), racemic (down).

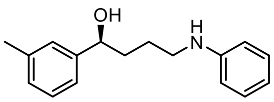

(*S*)-4-(phenylamino)-1-(m-tolyl)butan-1-ol [(*S*)-3ga]

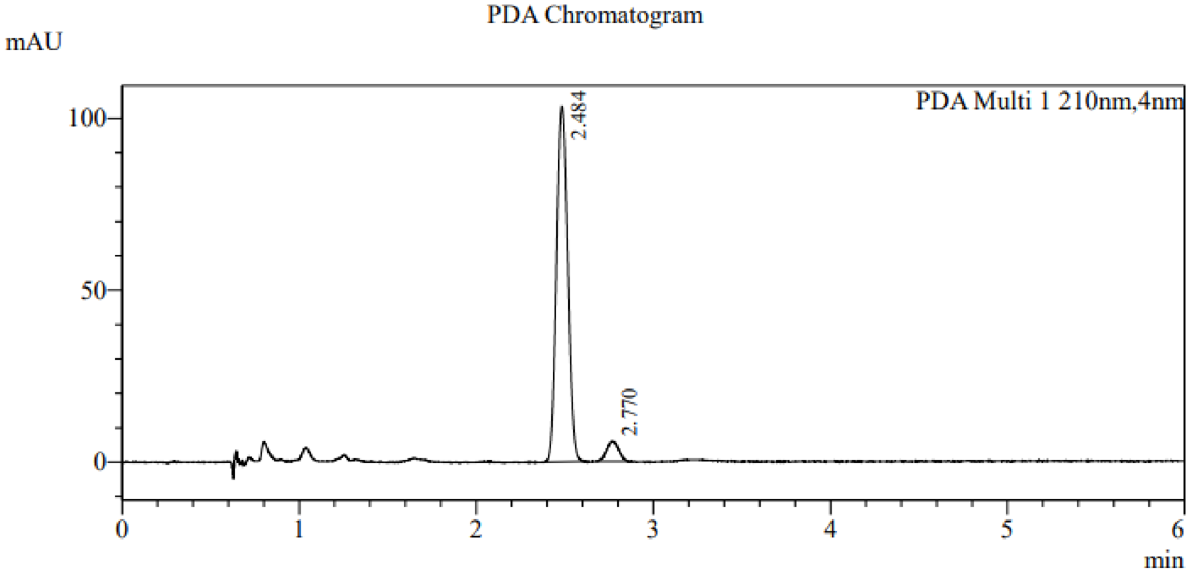

Peak Table

PDA Ch1 210nm

Peak#	Ret. Time	Height	Area	Area%
1	2.484	102081	456831	94.257
2	2.770	5769	27834	5.743

88% ee

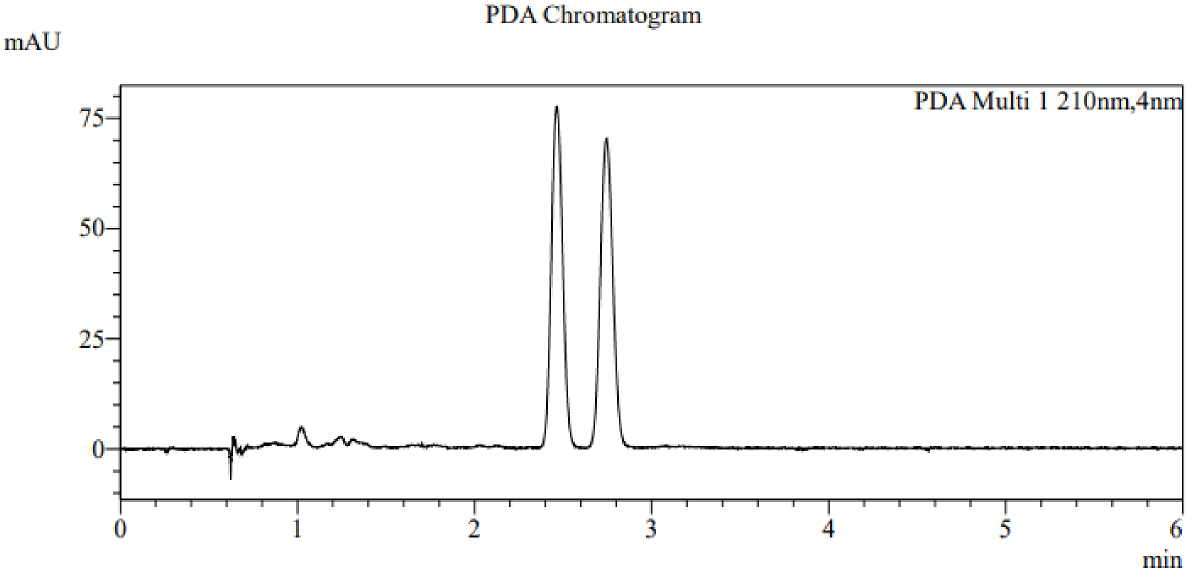

Figure S102. SFC chromatogram of **(*S*)-3ga**, chiral (up), racemic (down).

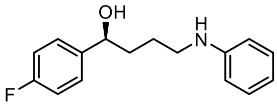

(*S*)-1-(4-fluorophenyl)-4-(phenylamino)butan-1-ol [(*S*)-3ia]

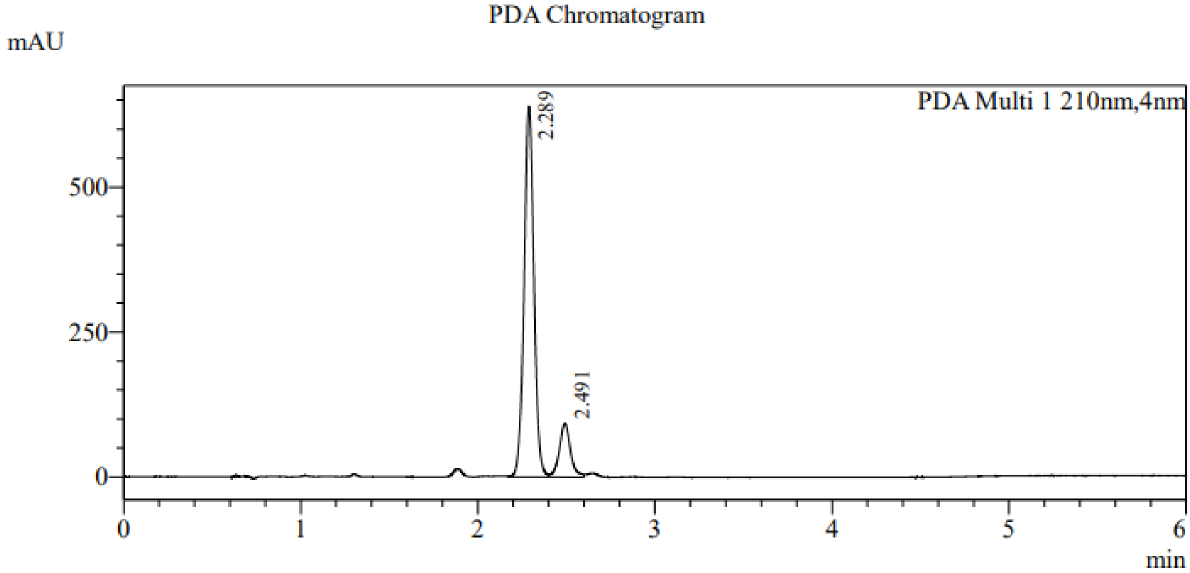

Peak Table

PDA Ch1 210nm

Peak#	Ret. Time	Height	Area	Area%
1	2.289	636314	2470422	85.863
2	2.491	91191	406757	14.137

72% ee

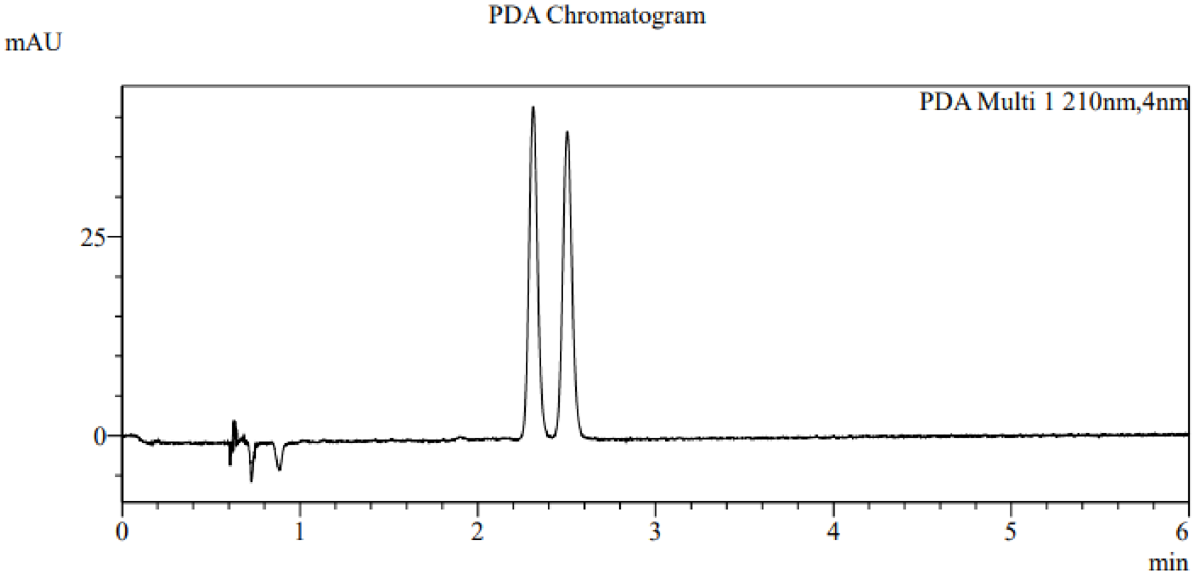

Figure S103. SFC chromatogram of **(*S*)-3ia**, chiral (up), racemic (down).

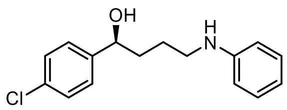

(*S*)-1-(4-chlorophenyl)-4-(phenylamino)butan-1-ol [(*S*)-3ja]

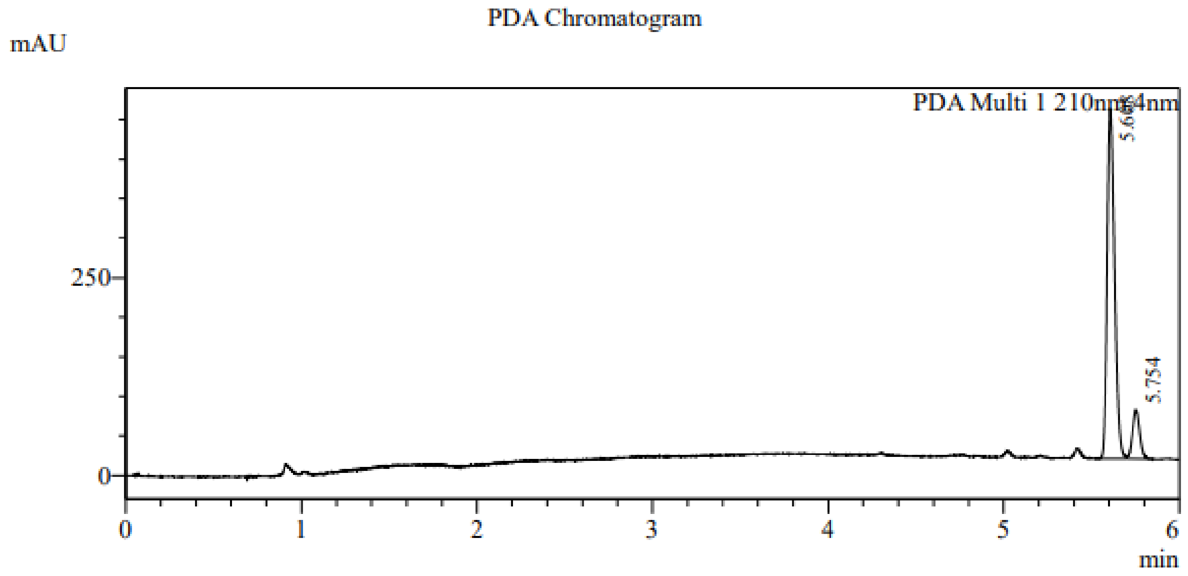

Peak Table

PDA Ch1 210nm

Peak#	Ret. Time	Height	Area	Area%
1	5.608	432016	1297036	87.816
2	5.754	61323	179963	12.184

75% ee

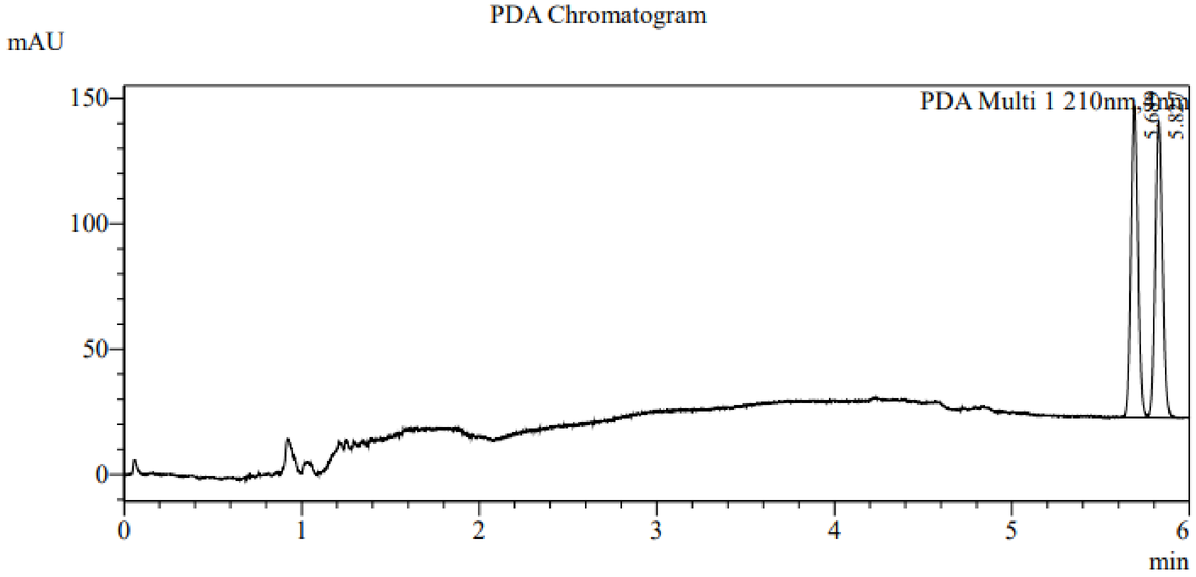

Figure S104. SFC chromatogram of **(*S*)-3ja**, chiral (up), racemic (down).

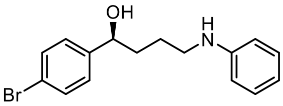

(*S*)-1-(4-bromophenyl)-4-(phenylamino)butan-1-ol [(*S*)-3ka]

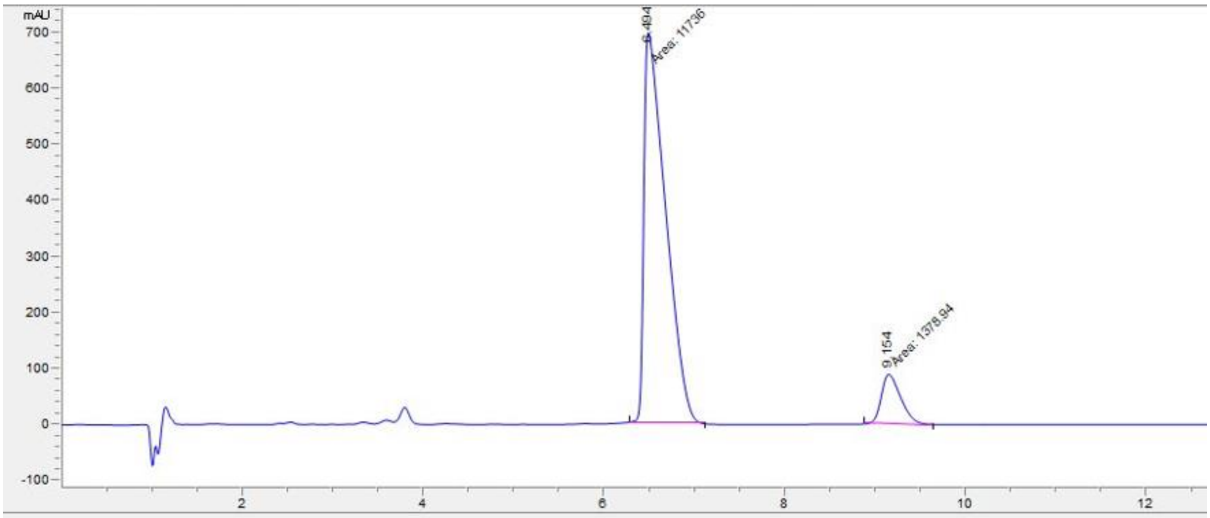

#	Time	Type	Area	Height	Width	Area%	Symmetry
1	6.494	MM	11736	702.7	0.2784	89.486	0.258
2	9.154	MM	1378.9	89.8	0.2558	10.514	0.595

79% ee

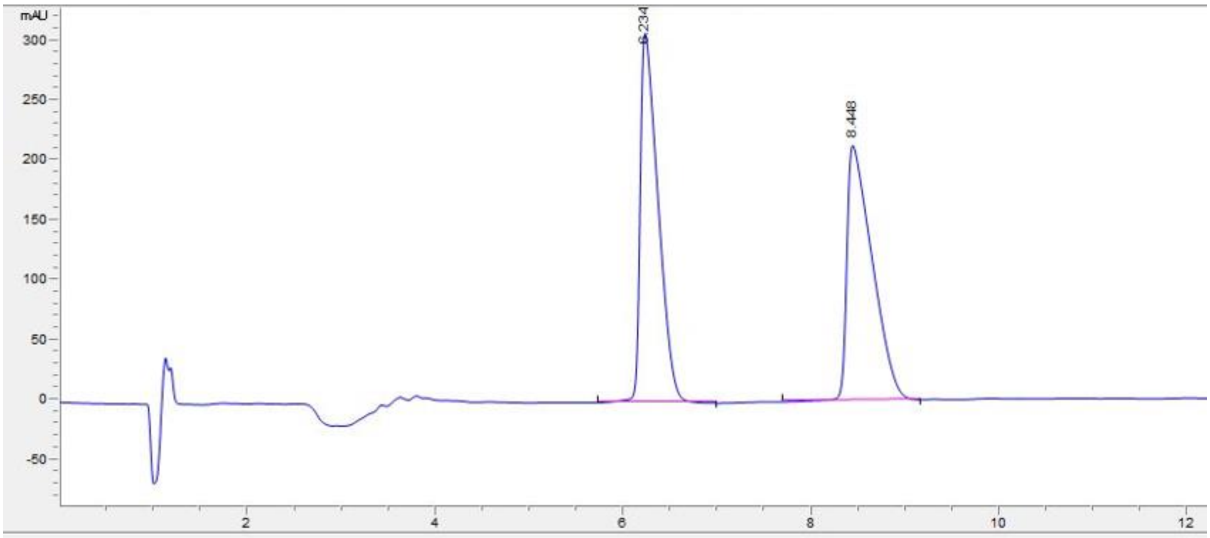

#	Time	Type	Area	Height	Width	Area%	Symmetry
1	6.234	BB	4121.2	310.3	0.2029	50.742	0.357
2	8.448	BB	4000.7	214.1	0.284	49.258	0.292

Figure S105. SFC chromatogram of **(*S*)-3ka**, chiral (up), racemic (down).

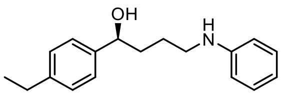

(*S*)-1-(4-ethylphenyl)-4-(phenylamino)butan-1-ol [(*S*)-3na]

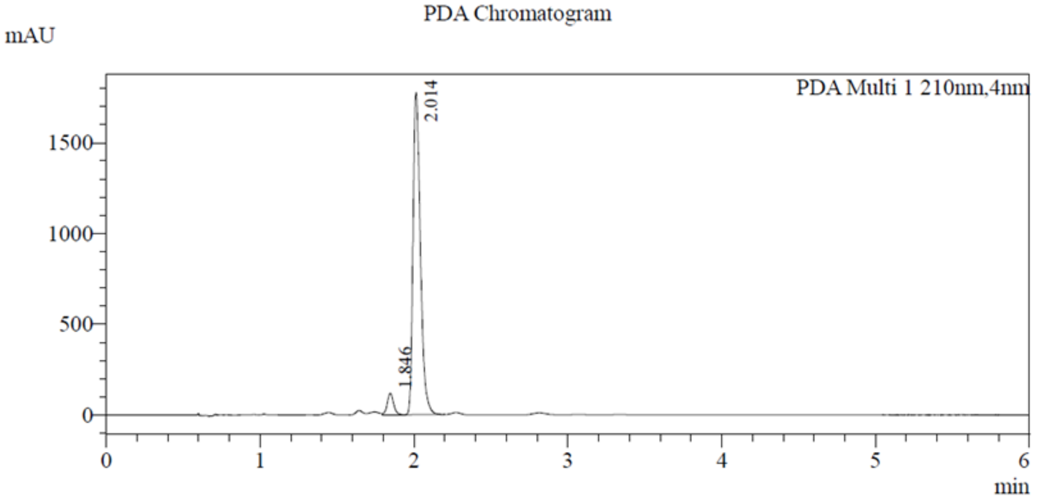

Peak Table

PDA Ch1 210nm

Peak#	Ret. Time	Area	Height	Area%
1	1.846	338621	117277	5.411
2	2.014	5918901	1761879	94.589

89% ee

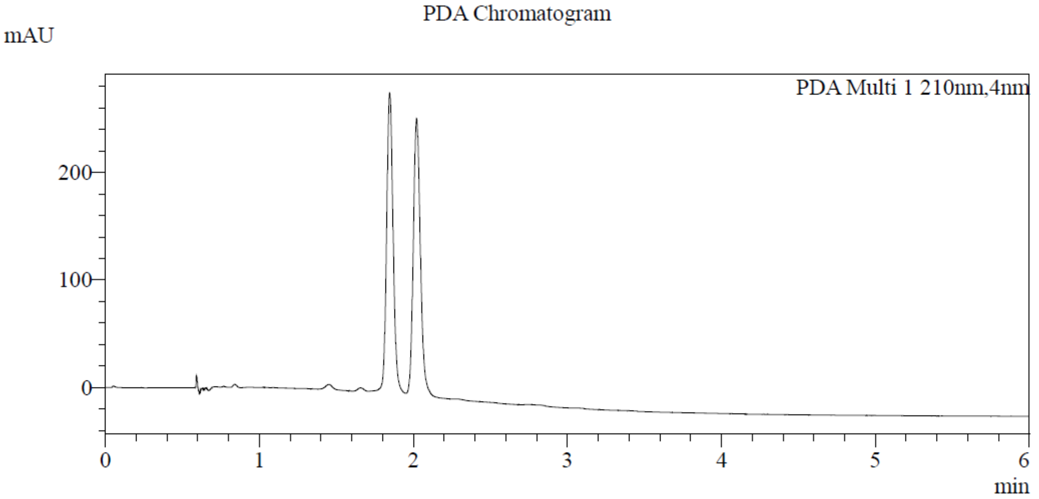

Figure S106. SFC chromatogram of **(*S*)-3na**, chiral (up), racemic (down).

1.9 References

(1) Shimkin, K. W.; Gildner, P. G.; Watson, D. A. Copper-Catalyzed Alkylation of Nitroalkanes with α-Bromonitriles: Synthesis of β-Cyanonitroalkanes. *Org. Lett.* **2016**, *18*, 988– 991.

(2) Paolillo, J. M.; Duke, A. D.; Gogarnoiu, E. S.; Wise, D. E.; Parasram, M. Anaerobic Hydroxylation of C(sp^3)-H Bonds Enabled by the Synergistic Nature of Photoexcited Nitroarenes. *J. Am. Chem. Soc.* **2023**, *145*, 2794– 2799.

(3) Huy, P. H.; Koskinen, A. M. P. Efficient, Stereodivergent Access to 3-Piperidinols by Traceless $P(OEt)_3$ Cyclodehydration. *Org. Lett.* **2013**, *15*, 5178– 5181.

(4) Arai, N.; Namba, T.; Kawaguchi, K.; Matsumoto, Y.; Ohkuma, T. Chemoselectivity Control in the Asymmetric Hydrogenation of γ- and δ-Keto Esters into Hydroxy Esters or Diols. *Angew. Chem., Int. Ed.* **2018**, *57*, 1386– 1389.

(5) Duffy, L. A.; Matsubara, H.; Procter, D. J. A Ring Size-Selective Reduction of Lactones Using SmI_2 and H_2O. *J. Am. Chem. Soc.* **2008**, *130*, 1136– 1137.

(6) Čorić, I.; Müller, S.; List, B. Kinetic resolution of homoaldols via catalytic asymmetric transacetalization. *J. Am. Chem. Soc.* **2010**, *132*, 17370–17373.

(7) Estopiñá-Durán, S.; Donnelly, L. J.; Mclean, E. B.; Hockin, B. M.; Slawin, A. M. Z.; Taylor, J. E. Aryl Boronic Acid Catalysed Dehydrative Substitution of Benzylic Alcohols for C–O Bond Formation. *Chem. - Eur. J.* **2019**, *25*, 3950– 3956.

(8) Baek, Y.; Betley, T. A. Catalytic C–H Amination Mediated by Dipyrrin Cobalt Imidos. *J. Am. Chem. Soc.* **2019**, *141*, 7797– 7806.

(9) Yang, X.-H.; Xie, J.-H.; Liu, W.-P.; Zhou, Q.-L. Catalytic Asymmetric Hydrogenation of δ-Ketoesters: Highly Efficient Approach to Chiral 1,5-Diols. *Angew. Chem., Int. Ed.* **2013**, *52*, 7833– 7836.

(10) Watile, R. A.; Bunrit, A.; Margalef, J.; Akkarasamiyo, S.; Ayub, R.; Lagerspets, E.; Biswas, S.; Repo, T.; Samec, J. S. M. Intramolecular substitutions of secondary and tertiary alcohols with chirality transfer by an iron (III) catalyst. *Nat. Commun.* **2019**, *10*, 3826.

(11) Zhou, J.; Meng, L.; Yang, Z.; Wang, J. Enantio- and Regioselective Cascade Hydroboration of Methylenecyclopropanes for Facile Access to Chiral 1,3- and 1,4-Bis(boronates). *Adv. Sci.* **2024**, *11*, 2400096– 24000104.

(12) Arai, N.; Namba, T.; Kawaguchi, K.; Matsumoto, Y.; Ohkuma, T. Chemoselectivity Control in the Asymmetric Hydrogenation of γ- and δ-Keto Esters into Hydroxy Esters or Diols. *Angew. Chem., Int. Ed.* **2018**, *57*, 1386– 1389.

(13) Nie, Z.; Chiou, M.-F.; Cui, J.; Qu, Y.; Zhu, X.; Jian, W.; Xiong, H.; Li, Y.; Bao, H. Copper-Catalyzed Radical Enantioselective Carbo-Esterification of Styrenes Enabled by a Perfluoroalkylated-PyBox Ligand. *Angew. Chem., Int. Ed.* **2022**, *61*, e202202077.

(14) Bermejo-López, A.; Raeder, M.; Martínez-Castro, E.; Martín-Matute, B. Selective and quantitative functionalization of unprotected α-amino acids using a recyclable homogeneous catalyst. *Chem* **2022**, *8*, 3302– 3323.

(15) Bermejo-López, A.; Li, M.; Dharanipragada, N. V. R. A.; Raeder, M.; Inge, A. K.; Himo, F.; Martín-Matute, B. A general catalyst for the base-free mono-N-alkylation of aromatic and aliphatic amines with alcohols. *Cell Rep. Phys. Sci.* **2024**, *5*, 101991.

(16) Marichev, K. O.; Takacs, J. M. Ruthenium-Catalyzed Amination of Secondary Alcohols Using Borrowing Hydrogen Methodology. *ACS Catal.* **2016**, *6*, 2205– 2210.

(17) Hoye, T. R.; Eklov, B. M.; Ryba, T. D.; Voloshin, M.; Yao, L. J. No–D NMR (No–Deuterium Proton NMR) Spectroscopy: A Simple Yet Powerful Method for Analyzing Reaction and Reagent Solutions. *Org. Lett.* **2004**, *6*, 953–956.

(18) Maashi, H. A.; Husayni, A. H.; Reid, K. M, M. E.; Harnedy, J.; Herneman, E. C.; Pera-Titus, M.; Morrill, L. C. Electrochemical Synthesis of C(sp^3)-Rich Heterocycles *via* Mesolytic Cleavage of Anodically Generated Aromatic Radical Cations. *Org. Lett.* **2024**, *26*, 9051–9055.
